# 2020 APHRS/HRS expert consensus statement on the investigation of decedents with sudden unexplained death and patients with sudden cardiac arrest, and of their families

**DOI:** 10.1016/j.hrthm.2020.10.010

**Published:** 2020-10-19

**Authors:** Martin K. Stiles, Arthur A. M. Wilde, Dominic J. Abrams, Michael J. Ackerman, Christine M. Albert, Elijah R. Behr, Sumeet S. Chugh, Martina C. Cornel, Karen Gardner, Jodie Ingles, Cynthia A. James, Jyh-Ming Jimmy Juang, Stefan Kääb, Elizabeth S. Kaufman, Andrew D. Krahn, Steven A. Lubitz, Heather MacLeod, Carlos A. Morillo, Koonlawee Nademanee, Vincent Probst, Elizabeth V. Saarel, Luciana Sacilotto, Christopher Semsarian, Mary N. Sheppard, Wataru Shimizu, Jonathan R. Skinner, Jacob Tfelt-Hansen, Dao Wu Wang

**Affiliations:** 1 Waikato Clinical School, Faculty of Medicine and Health Science, The University of Auckland, Hamilton, New Zealand; 2 Amsterdam University Medical Center, University of Amsterdam, Heart Center, Department of Clinical and Experimental Cardiology, Amsterdam, the Netherlands; 3 Boston Children’s Hospital, Boston, Massachusetts, USA; 4 Mayo Clinic, Rochester, Minnesota, USA; 5 Cedars-Sinai Medical Center, Los Angeles, California, USA; 6 Cardiovascular Clinical Academic Group, Molecular and Clinical Sciences Institute, St George’s, University of London, and St George’s University Hospitals NHS Foundation Trust, London, United Kingdom; 7 Amsterdam University Medical Center, Vrije Universiteit Amsterdam, Clinical Genetics, Amsterdam Public Health Research Institute, Amsterdam, the Netherlands; 8 University of New South Wales, Canberra, Australia; 9 Agnes Ginges Centre for Molecular Cardiology at Centenary Institute, The University of Sydney, Sydney, Australia; 10 Johns Hopkins University, Baltimore, Maryland, USA; 11 Cardiovascular Center and Division of Cardiology, Department of Internal Medicine, National Taiwan University Hospital and National Taiwan University College of Medicine, Taipei, Taiwan; 12 Department of Medicine I, University Hospital, LMU Munich, Munich, Germany; 13 MetroHealth Campus, Case Western Reserve University, Cleveland, Ohio, USA; 14 The University of British Columbia, Vancouver, British Columbia, Canada; 15 Massachusetts General Hospital, Boston, Massachusetts, USA; 16 Data Coordinating Center for the Sudden Death in the Young Case Registry, Okemos, Michigan, USA; 17 Libin Cardiovascular Institute, Calgary, Alberta, Canada; 18 Chulalongkorn University, Faculty of Medicine, and Pacific Rim Electrophysiology Research Institute at Bumrungrad Hospital, Bangkok, Thailand; 19 CHU de Nantes, Nantes, France; 20 Cleveland Clinic Lerner College of Cardiology at Case Western Reserve University, Cleveland, Ohio, and St Luke’s Medical Center, Boise, Idaho, USA; 21 Heart Institute, University of São Paulo Medical School, São Paulo, Brazil; 22 Department of Cardiovascular Medicine, Nippon Medical School, Tokyo, Japan; 23 Cardiac Inherited Disease Group, Starship Hospital, Auckland, New Zealand; 24 Department of Forensic Medicine, Faculty of Medical Sciences, Rigshospitalet, Copenhagen, Denmark; 25 The First Affiliated Hospital of Nanjing Medical University, Nanjing, China.

**Keywords:** Brugada syndrome, Cardiac arrest, Cardiac genetics, Catecholaminergic polymorphic ventricular tachycardia, Defibrillator, Expert consensus statement, Genetic counseling, Guidelines, Long QT syndrome, Postmortem, Resuscitation, Sudden arrhythmic death syndrome, Sudden cardiac death, Sudden unexplained death, Ventricular arrhythmia

## Abstract

This international multidisciplinary document intends to provide clinicians with evidence-based practical patient-centered recommendations for evaluating patients and decedents with (aborted) sudden cardiac arrest and their families. The document includes a framework for the investigation of the family allowing steps to be taken, should an inherited condition be found, to minimize further events in affected relatives. Integral to the process is counseling of the patients and families, not only because of the emotionally charged subject, but because finding (or not finding) the cause of the arrest may influence management of family members. The formation of multidisciplinary teams is essential to provide a complete service to the patients and their families, and the varied expertise of the writing committee was formulated to reflect this need. The document sections were divided up and drafted by the writing committee members according to their expertise. The recommendations represent the consensus opinion of the entire writing committee, graded by Class of Recommendation and Level of Evidence. The recommendations were opened for public comment and reviewed by the relevant scientific and clinical document committees of the Asia Pacific Heart Rhythm Society (APHRS) and the Heart Rhythm Society (HRS); the document underwent external review and endorsement by the partner and collaborating societies. While the recommendations are for optimal care, it is recognized that not all resources will be available to all clinicians. Nevertheless, this document articulates the evaluation that the clinician should aspire to provide for patients with sudden cardiac arrest, decedents with sudden unexplained death, and their families.

## Section 1 Introduction

### 1.1. Purpose

This expert consensus statement represents an international multidisciplinary effort led by the Asia Pacific Heart Rhythm Society (APHRS), in partnership with the Heart Rhythm Society (HRS) and in collaboration with the Association for European Cardiovascular Pathology (AECVP), the European Heart Rhythm Association (EHRA), the European Society of Human Genetics (ESHG), the Latin American Heart Rhythm Society (LAHRS), the National Society of Genetic Counselors (NSGC) (USA), the Pediatric and Congenital Electrophysiology Society (PACES), and the European Reference Network for Rare and Low Prevalence Complex Diseases of the Heart: ERN GUARD-Heart. The intent is to provide clinicians with practical patient-centered recommendations for evaluating patients with sudden cardiac arrest (SCA), decedents with sudden cardiac death (SCD), and their families, based on all available evidence. Although the recommendations are for optimal care, the writing committee recognizes that not all resources will be available to all clinicians. Nevertheless, this document articulates the evaluation that the clinician should aspire to provide.

### 1.2. Organization of the Writing Committee

The writing committee consisted of internationally recognized experts from 14 countries in the fields of cardiac electrophysiology, cardiology, pediatric cardiology, genetic counseling, community genetics and public health genomics, and cardiac pathology, representing APHRS, HRS, AECVP, EHRA, ESHG, LAHRS, NSGC, PACES, and ERN GUARD-Heart and selected according to each society’s procedures. In addition, a patient representative was chosen to provide a consumer viewpoint. Each partner society nominated a chair, who did not have relevant relationships with industry and other entities (RWIs). In accordance with the APHRS policies, disclosure of any RWIs was required from the writing committee members (Appendix 1) and from the peer reviewers (Appendix 2); of the 28 committee members, 23 (82%) had no relevant RWIs. Recommendations were drafted by the writing committee members who did not have relevant RWIs.

### 1.3. Methodology and Evidence Review

After development of a preliminary outline, committee members were given writing assignments and a schedule of conference calls. Writing committee members conducted a comprehensive evidence search using MEDLINE/PubMed, Embase, and the Cochrane Library and summarized the evidence in standardized tables (Appendix 3), with attention to the study type, size, inclusion criteria, and key findings. The writing committee reviewed evidence and established consensus to generate recommendations, which are presented in a modular knowledge chunk format, with each chunk including a table of recommendations, a brief synopsis, recommendation-specific supportive text, flow diagrams or tables as appropriate, and references. Recommendations were formulated according to the American College of Cardiology (ACC)/American Heart Association (AHA) Class of Recommendation (COR) and Level of Evidence (LOE) system^1^ (Table 1) and were subject to a period of public comment. The COR indicates the strength of a recommendation based on assessment of the estimated benefits and risks; LOE rates the quality of evidence that supports the recommendation based on type, quantity, and consistency of data from clinical trials and other sources. Case reports were not used to support recommendations. The threshold for consensus was considered as 80% or higher agreement. The 74 recommendations were balloted by the 28 writing committee members and approved by an average of 94%. A quorum of two-thirds of the writing committee was met for all votes.

### 1.4. Document Review and Approval

After review by the entire writing committee, the recommendations were opened for public comment; the draft document was reviewed by the International Scientific Document Writing Committee of the APHRS and the Scientific and Clinical Documents Committee of the HRS and was revised prior to external review. The document underwent external peer review by reviewers appointed by the APHRS and HRS and each of the collaborating societies. After subsequent revisions and endorsement by the participating societies, the document was ready for publication.

### 1.5. Scope of the Document

This document provides a framework for the investigation of 1) patients with SCA, 2) decedents with sudden unexplained death (SUD), and 3) families of both SCA survivors and SUD victims, as many conditions responsible for the cardiac arrest or unexplained death may be familial. Identifying a cause is important for preventing further events in the family, should an inherited condition be found. Integral to the process is the counseling of the patients and families, not only because of the emotionally charged subject, but because finding (or not finding) the cause of the arrest may influence the futures of the family members. The disciplines of cardiology, pediatrics, radiology, pathology, counseling, psychology, and genetics are all involved in this process. Therefore, the formation of multidisciplinary teams is essential to provide a complete service to the patients and their families.

While this document endeavors to provide clinicians with practical recommendations for evaluating patients with SCA, decedents with SUD, and their families, the best approach will vary with the situation and will be influenced by, for example, the subject’s age and results of initial testing. Although some of the recommendations do specify an age cutoff, it is recognized that this age is somewhat arbitrary and may not be always appropriate for the disease being investigated for or the demographics of the patient’s country. Nevertheless, where an age is specified in a recommendation, it has passed the consensus voting of the writing group. Referral to a center with a multidisciplinary team experienced in such evaluations is recommended because it can facilitate navigation of these complexities. A multidisciplinary team can also help organize interval follow-up evaluations for SCA survivors and their family members. Repeated interval follow-up can reveal important new clinical data and allows for integration of new knowledge into the continued evaluation and care of these patients. The writing committee members recognize that not all investigative modalities recommended will be available in all circumstances; however, this document is an attempt to outline an approach to which the clinician should aspire.

### 1.6. Relevant Clinical Practice Documents

Table 2 lists pertinent guidelines and consensus statements that the writing committee considered for this document. The included documents contain relevant information for the diagnosis of patients with SCA and SCD.

### 1.7. Definitions

The terms used in the consensus statement are defined in Table 3.

## Section 2 Epidemiology

### 2.1. Epidemiology: Sudden Death

“Sudden unexplained death” refers to an unexpected and sudden death in an individual older than 1 year. Sudden death occurring unexpectedly within the first year of life is termed “sudden unexplained death in infancy” (SUDI). Multiple definitions have been in use over the past decades, although most recent studies implement a definition that differs between witnessed and unwitnessed events; in witnessed cases, death has to occur within 1 hour of change in cardiovascular status, whereas unwitnessed cases have to be seen alive and functioning normally within 24 hours of being found dead.^12,13^

SCD constitutes the majority of SUD.^14–16^ Reported overall SCD incidence rates vary across studies and countries, in part due to large difference in SCD definitions and methods for estimation of SCD rates. Previous studies report overall SCD rates ranging from 15 to 159 SCD per 100,000 persons per annum, corresponding to 6–20% of all deaths.^17–24^ However, both incidence and causes of SCD vary markedly with age. Lowest SCD incidence is observed in children and adolescents.^15,18,25–28^ SCD incidence is low in children and the young under 35 years and increases dramatically up until the age of approximately 60–80 years.^15,19,20,29,30^

**Figure 1:**
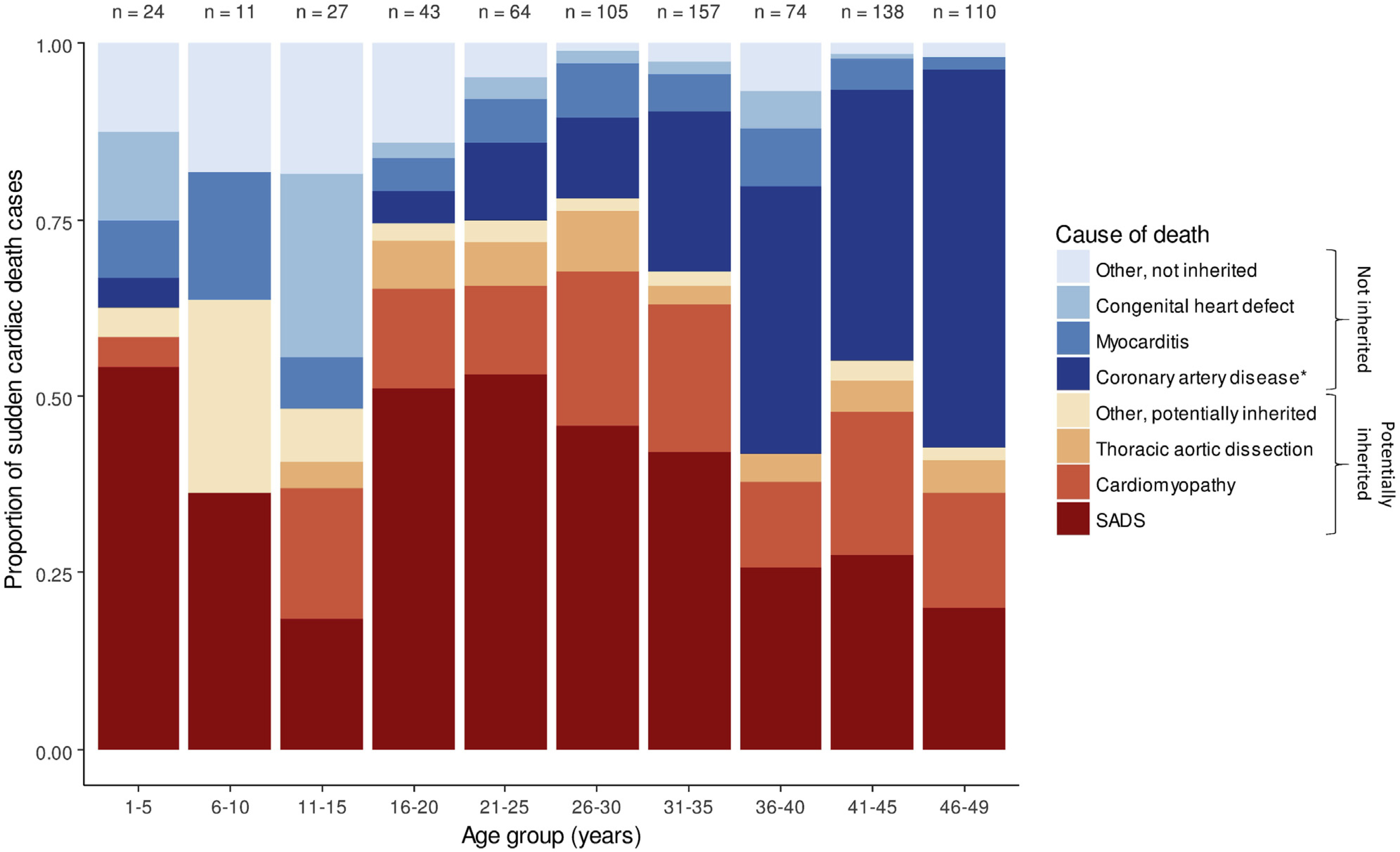
Distribution of causes of death among autopsied cases of sudden cardiac death (n = 753) according to age in persons aged 1–49 years in Denmark (J.T.-H., unpublished data). SADS = sudden arrhythmic death syndrome. *Coronary artery disease, especially in young persons, may be due to inherited disease (eg, familial hypercholesterolemia).

In young persons aged 1–35 years, most SCDs are caused by potentially inherited heart diseases, including primary arrhythmogenic disorders (eg, congenital long QT syndrome and catecholaminergic polymorphic ventricular tachycardia [CPVT]), hypertrophic cardiomyopathy, arrhythmogenic cardiomyopathy, and dilated cardiomyopathy;^15,18,25–29,31,32^ however, coronary artery disease, anomalous coronary arteries, aortic dissection, congenital heart disease, and myocarditis are also potential causes, potentially with a non-negligible genetic component (Figure 1). From the age of 35 years, coronary artery disease becomes the most common cause of SCD, although potentially inherited heart diseases remain a common cause of SCD at least until the age of 50 years.^27,29,33^ Individuals with SUD who subsequently have negative pathological and toxicological assessment may be assumed to have sudden arrhythmic death (syndrome), or SAD(S), a term synonymous with “autopsy-negative SUD.”

At any age, males have higher SCD rates compared with females, even after adjustment for risk factors of coronary heart disease.^34^ Ethnic background seems to have large effect.^35,3,6^

**Table:** Recommendations for improving outcomes from sudden death

COR	LOE	Recommendations	References
1	B-NR	1. Investigation of SUD at a young age should be made a public health priority due to the combined prevalence of inherited cardiac diseases of at least 1:500, the years of potential life lost, and the significant impact on the family and community; therefore, public funding should be allocated for relevant investigations.	^15,25,37^
1	C-EO	2. Identification of inherited cardiac conditions that predispose to SCD should be made a public health priority, as diagnosis may prevent future cardiac events in affected family members.	
1	B-NR	3. The burden of SUD and varied outcomes in relation to sex, different ethnic populations, and socioeconomic backgrounds should be investigated worldwide.	^35,36,38,39^

#### Synopsis

SUD is a tragedy and, in the case of an underlying genetic predisposition, may be preventable. The main cause of SUD is SCD. SCD in the young often occurs in people who were thought to be well, may occur without warning symptoms, and is often the first presentation of an underlying genetic heart disease. Across all ages, estimates differ from 5% to 20% of all deaths, and ethnicity-specific data on SCD incidences worldwide are sparse. Cause of death changes according to age (Figure 1). Exact estimates of the burden of SCD are crucial in order to adjudicate public health spending.

#### Recommendation-Specific Supportive Text

1. and 2. Inherited cardiac disorders are the main cause of SCD in the young. Sudden death is SCD in 60–90% depending on age, of which the majority is potentially from inherited cardiac disease.^2,15,40^ Exact estimates of the burden of SCD are crucial in order to adjudicate public health spending.^25^

3. Estimates of SCD among different ethnic backgrounds are sparse worldwide.^35,36,38,39^

### 2.2. Epidemiology: Sudden Cardiac Arrest Survivors

#### 2.2.1. Background

Out-of-hospital cardiac arrest (OHCA) is a leading cause of mortality globally^41–43^ and is defined as the loss of functional cardiac mechanical activity in association with an absence of systemic circulation, occurring outside of a hospital setting. The exact burden of OHCA remains unknown, since a considerable number of cases are not attended by emergency medical services (EMS) and regional variations are prevalent in both reporting systems and survival.^17,44,45^ Approximately 275,000 people in Europe have cardiac arrest treated by EMS per year, with only 29,000 (10.5%) surviving hospital discharge.^46^ In England, 28,729 EMS-treated OHCA cases were reported in 2014 (53 cases per 100,000 of the resident population), with only 7.9% surviving to hospital discharge.^47^ In the United States, reports from 35 communities suggested an incidence of 55 per 100,000 person-years^48^ or approximately 155,000 individuals having an EMS-treated all-rhythm OHCA per year.^48^ Globally, the weighted incidence estimates per 100,000 person-years of EMS-treated OHCA are 34.4 in Europe, 53.1 in North America, 59.4 in Asia, and 49.7 in Australia. For reported survival estimates, the percentage survival to discharge was 7.6% in Europe, 6.8% in North America, 3.0% in Asia, and 9.7% in Australia.^42^

Significant geographical variation in the incidence of OHCA associated with poor outcomes has remained unchanged in the past 3 decades.^17,41,42,44^ However, implementation of coordinated efforts targeted at improving the local chain of survival in some cities has improved regional survival to 20–40%.^49,50^ This survival benefit can be partially attributed to varying definitions of OHCA,^42^ but it is primarily due to a coordinated effort to optimize the effectiveness of the local chain of survival.^51^ Identifying and improving weak links in the local chain of survival, paired with targeted approaches to improve the effectiveness, has resulted in positive outcomes achieved in several geographic regions.^49,52–54^

#### 2.2.2. Causes of Out-of-Hospital Cardiac Arrest

OHCA causes are classified into cardiac and noncardiac causes.^47,55,56^ Approximately 80% of individuals presenting with OHCA reached by EMS, and in whom resuscitation is considered possible, have a cardiac cause.^56^

OHCA can affect seemingly fit and healthy athletes, young adults, or children. The incidence of SCD in athletes can range from 1 in 23,000 to 1 in 200,000 athletes per year, depending on a number of factors including populations studied.^57,58^ In a retrospective analysis of the Rescu Epistry database of consecutive OHCA attended by EMS in a specific area of Ontario, Canada, the incidence of SCD during participation in competitive sports was reported to be 0.76 cases per 100,000 athlete-years.^59^ The main causes of SCD were stratified by age. In those younger than 35 years, structural heart and primary arrhythmic causes were most common. In those aged between 35 and 45 years, coronary artery disease was the most frequent underlying pathology.^59^ In a prospective study of children and young adults aged 1–35 years, 490 cases of SCD were identified from centers in Australia and New Zealand.^25^ The cause of death was unexplained in 40% of these cases at autopsy, in whom a structurally normal heart was reported.^25^ In this study, the annual incidence of SCD was calculated to be 1.3 cases per 100,000 people. When stratified according to age group, the highest incidence (3.2 cases per 100,000 people per year) was observed in those aged 31–35 years. Coronary artery disease was the most common cause ascribed. Younger age and SCD occurring at night were independently associated with unexplained SCD, probably due to congenital channelopathies. Less common causes were inherited cardiomyopathies (eg, dilated, hypertrophic, and arrhythmogenic right ventricular), myocarditis, and aortic dissection.

**Figure 2:**
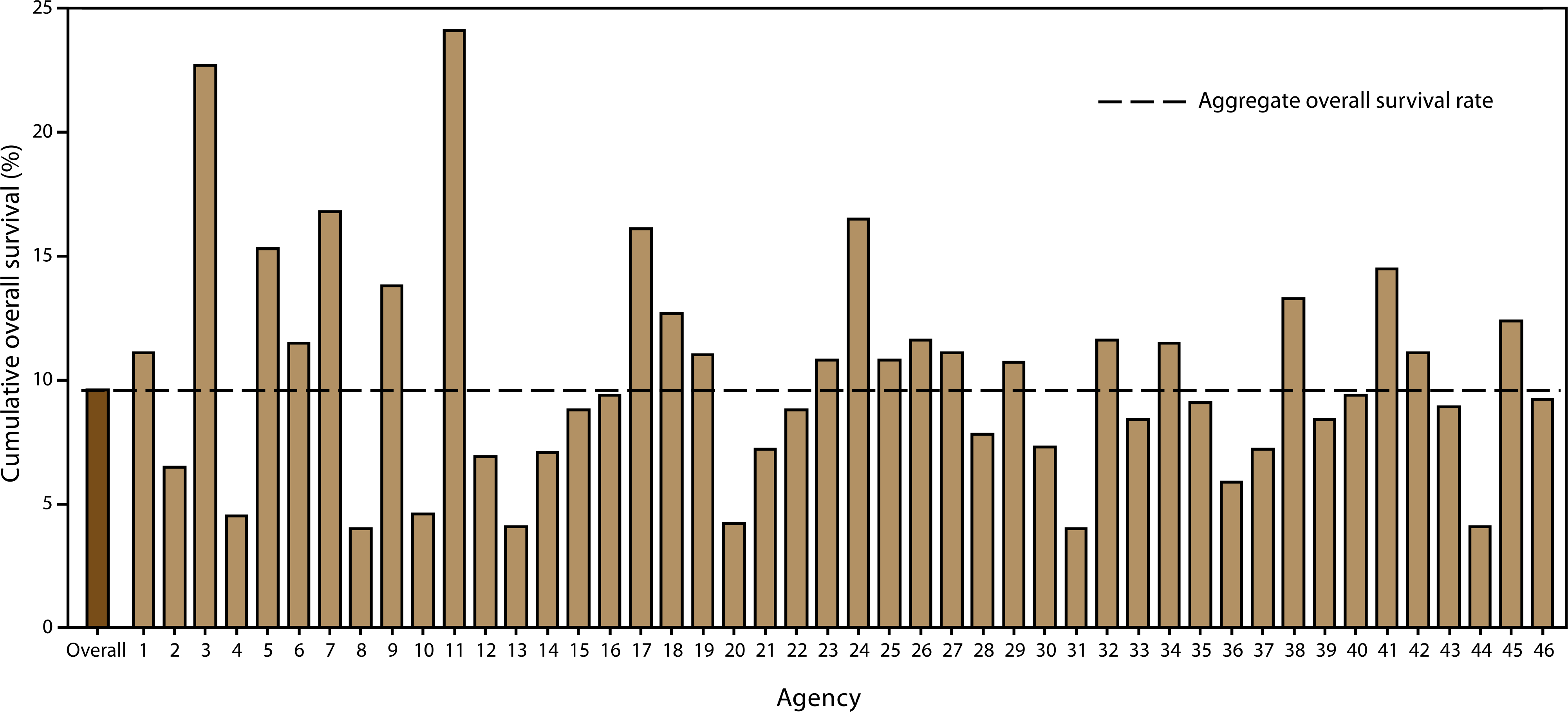
Cumulative overall survival rates, by participating emergency medical services agency—Cardiac Arrest Registry to Enhance Survival (CARES), United States, October 1, 2005–December 31, 2010. Agencies sorted by total number of out-of-hospital cardiac arrest events in CARES (from low to high; range: 18–5,434).^60^

The Cardiac Arrest Registry to Enhance Survival (CARES), established by the Centers for Disease Control and Prevention (CDC),^60^ evaluated OHCA events of presumed cardiac etiology that involve persons who received resuscitative effort. OHCA is defined in CARES as a cardiac arrest that occurred in the prehospital setting, had a presumed cardiac etiology, and involved a person who received resuscitative efforts, including cardiopulmonary resuscitation (CPR) or defibrillation. The registry includes 40,274 OHCA records, of which 31,689 OHCA events were presumed to be of cardiac etiology (eg, myocardial infarction or arrhythmia) that received resuscitation efforts in the prehospital setting (mean age 64.0 years [SD 18.2]; 61.1% male). The survival rate to hospital admission was 26.3%, and the overall survival rate from cardiac arrest to hospital discharge was 9.6% (Figure 2). Approximately 36.7% of OHCA events were witnessed by a bystander. Only 33.3% of all patients received bystander CPR, and only 3.7% were treated by bystanders with an automated external defibrillator (AED) before the arrival of EMS providers.

**Figure 3:**
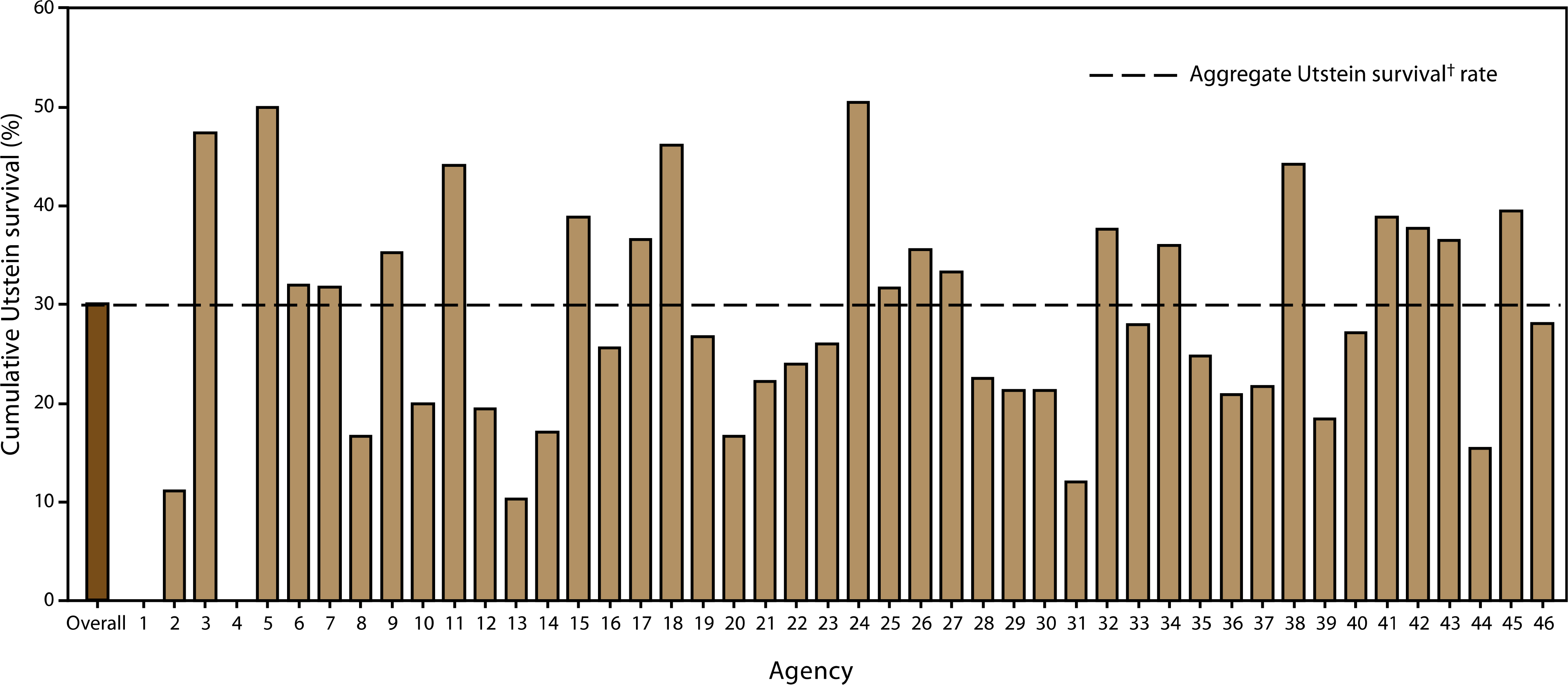
Cumulative Utstein survival rates (patients alive when arriving to hospital) by participating emergency medical services agency—Cardiac Arrest Registry to Enhance Survival (CARES), United States, October 1, 2005–December 31, 2010. Agencies sorted by total number of out-of-hospital cardiac arrest events in CARES (from low to high).^60^ †Utstein survival refers to survival to hospital discharge of persons whose cardiac arrest events were witnessed by a bystander and had an initial rhythm of ventricular fibrillation or pulseless ventricular tachycardia (range: 0–598).

The group most likely to survive an OHCA is persons who are witnessed to collapse by a bystander and found in a shockable rhythm (ie, arrhythmias leading to ventricular fibrillation or pulseless ventricular tachycardia).^60^ Among this group, survival to discharge was 30.1% (Figure 3). A subgroup analysis, performed among persons who experienced OHCA events unwitnessed by EMS, revealed that whites were significantly more likely to receive CPR than blacks, Hispanics, or members of other racial/ethnic populations (*p* < 0.001). Overall survival to hospital discharge of patients whose events were not witnessed by EMS personnel was 8.5%. Of these, patients who received bystander CPR had a significantly higher rate of overall survival (11.2%) than those who did not (7.0%) (*p* < 0.001).

**Figure 4:**
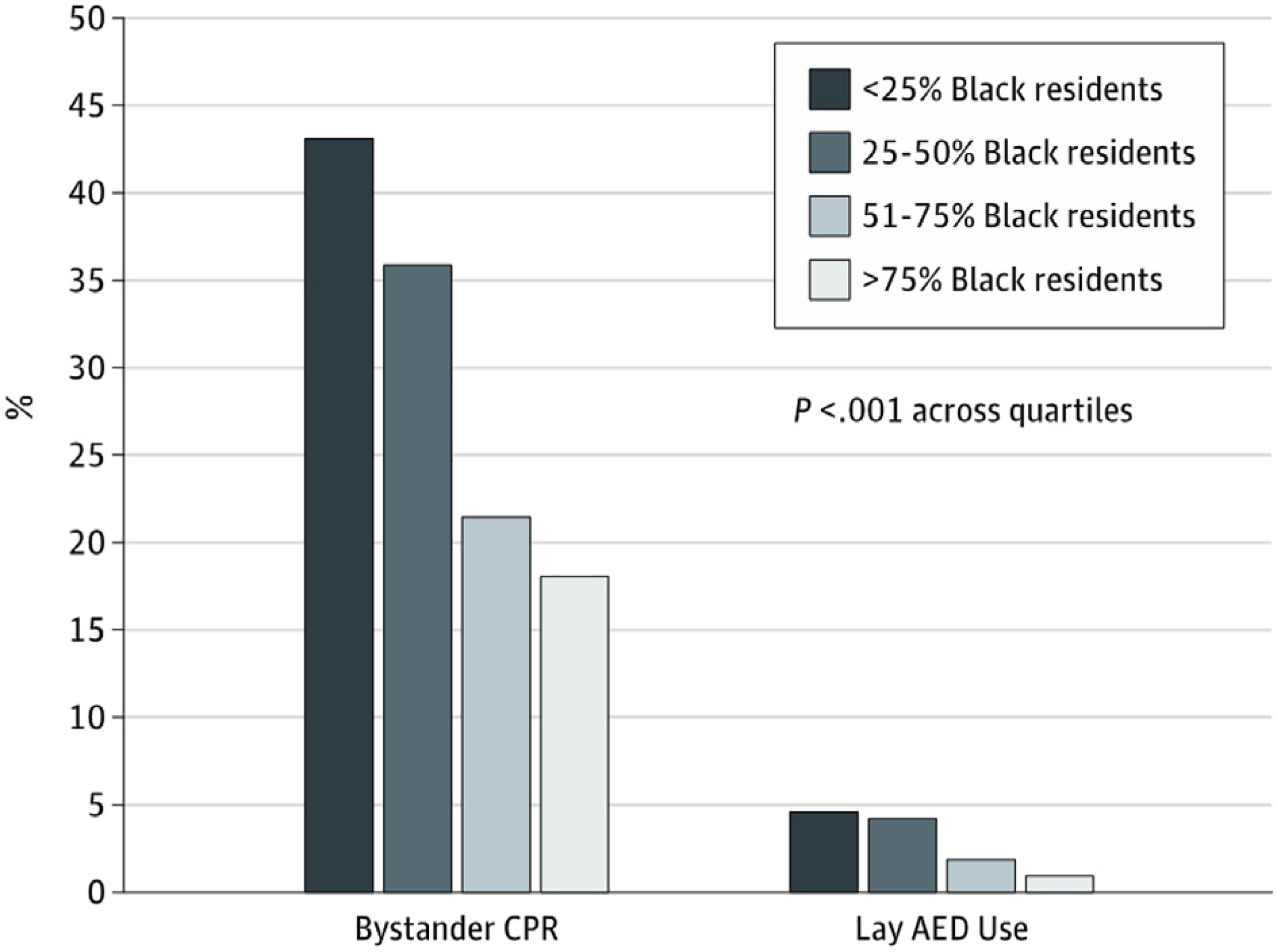
Bystander treatments of patients with out-of-hospital cardiac arrest before emergency medical services arrival among neighborhoods by percentage of black residents. Reprinted with permission from the American Medical Association.^39^ AED = automated external defibrillator; CPR = cardiopulmonary resuscitation.

Figure 4 shows bystander CPR and lay AED use by percentage of black residents in the area. Directing attention toward improving education, availability of AEDs, and treatment of cardiac arrest in predominantly black neighborhoods may save lives.^39^

Bystander AED use in OHCA in pediatric populations is variable and uncommon, with important variations based on neighborhood characteristics leading to marked disparities in survival and outcomes. Griffis et al.^38^ reported that AED use (likely due to availability) was more common in neighborhoods with a median household income of >$50,000 per year (12.3%; *p* = 0.016), <10% unemployment (12.1%; *p* = 0.002), and >80% high school education (11.8%; *p* = 0.002). Greater survival to hospital discharge and neurologically favorable survival were among arrests with bystander AED use, varying by neighborhood characteristics.

#### 2.2.3. Public Health Implications

**Figure 5:**
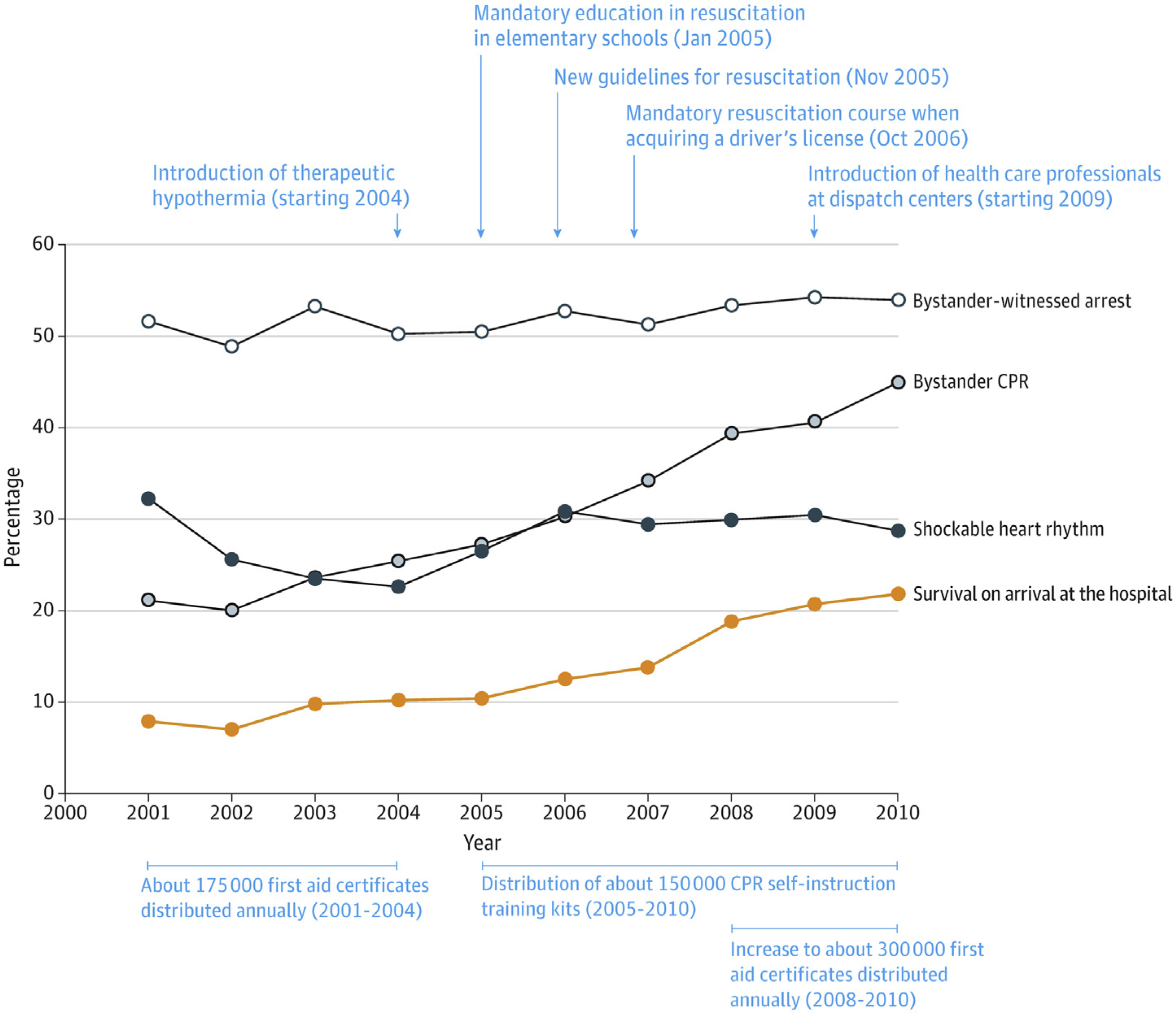
Bystander-witnessed arrest, bystander cardiopulmonary resuscitation (CPR), shockable heart rhythm as first recorded rhythm, and survival on arrival at the hospital, Denmark, 2001–2010. Reprinted with permission from the American Medical Association.^54^

The majority of persons who experience an OHCA event, irrespective of etiology, do not receive bystander CPR or other timely interventions that are known to improve the likelihood of survival to hospital discharge (eg, defibrillation).^54^ Because nearly half of cardiac arrest events are witnessed, efforts to increase survival rates should focus on timely and effective delivery of interventions by bystanders and EMS personnel (Figure 5).

**Figure 6:**
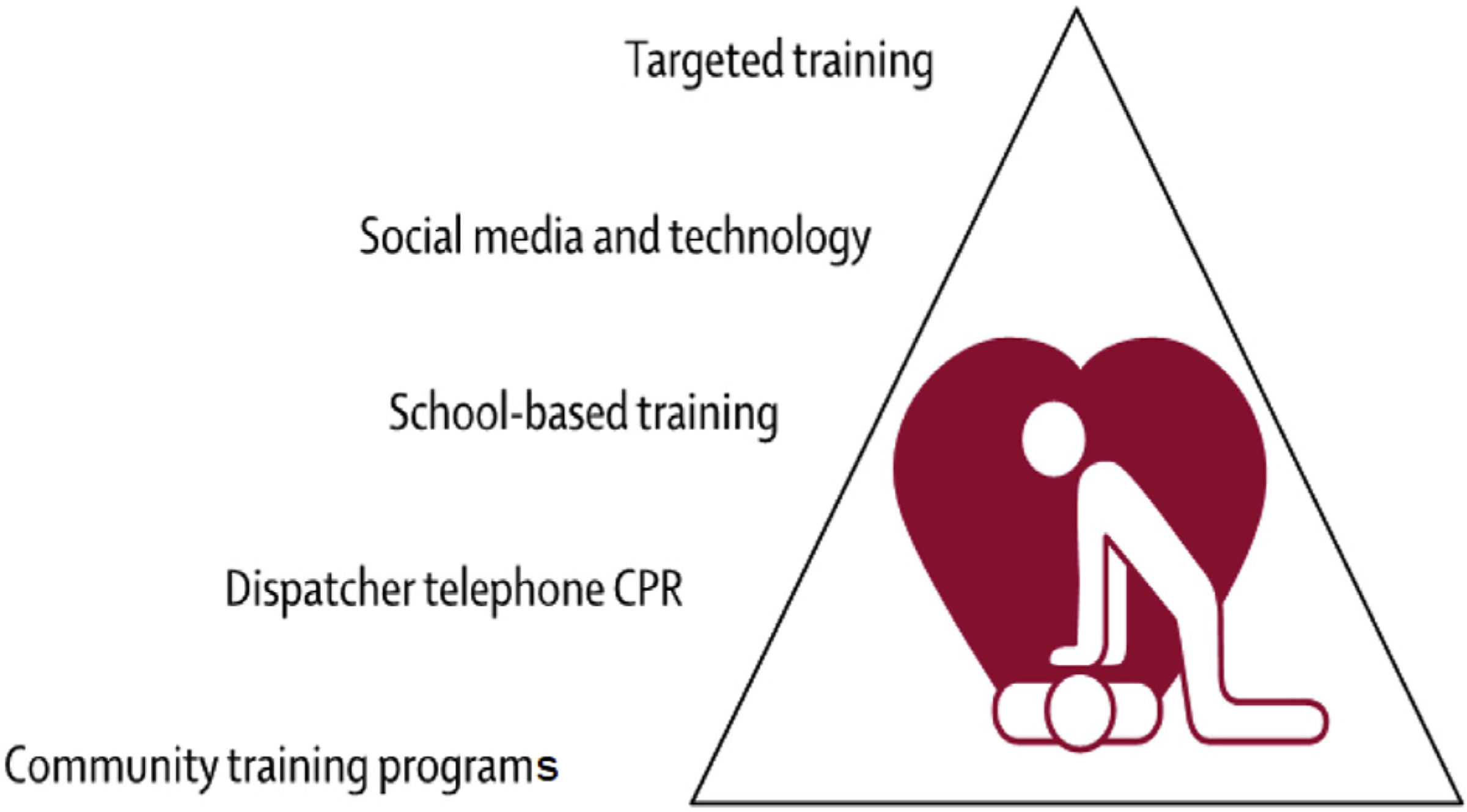
Examples of complimentary bystander cardiopulmonary resuscitation (CPR) programs. Reprinted with permission from Elsevier.^64^

Education of public officials and community members regarding the importance of increasing rates of bystander CPR and promoting the use of early defibrillation by lay and professional rescuers is critical to increasing survival rates. Reporting at local and national levels can enable local and national public health and EMS agencies to coordinate their efforts to target improving emergency response for OHCA events, regardless of etiology, which can lead to improvement in OHCA survival rates (Figure 6).

**Table:** Recommendations for improving outcomes in SCA survivors

COR	LOE	Recommendations	References
1	B-NR	1. Targeted CPR training should be widely implemented with particular emphasis on low-income communities, ethnic minorities, and middle- to low-income countries.	^38,60^
1	B-NR	2. The burden of out-of-hospital SCA and varied outcomes in different ethnic populations and socioeconomic backgrounds should be investigated worldwide.	^35,38,39^
1	B-NR	3. Appropriately maintained AEDs should be readily available at schools, stadiums, public transport stations, casinos, etc, as well as venues where no other access to AEDs is available (eg, trains, ships, planes), with appropriate training of users.	^61,62^

#### Synopsis

OHCA remains a significant cause of mortality globally. Despite implementation of cardiac arrest protocols including CPR training and AEDs, only 33% of witnessed OHCA cases receive bystander CPR and less than 4% are defibrillated onsite. OHCA hospital discharge survival remains dismal at around 10% and has remained stagnant for the past 3 decades. Significant geographic variation in OHCA incidence and the role of social disparities merit further research. Public health campaigns promoting CPR training in at-risk communities and greater availability of AEDs are needed.

#### Recommendation-Specific Supportive Text

1. Coordinated efforts targeted at improving the local chain of survival have improved regional survival.^49,50^ Targeted approaches to improve the effectiveness of CPR have resulted in positive outcomes.^52–54^ The group most likely to survive an OHCA is persons who are witnessed to collapse by a bystander and found in a shockable rhythm, so widespread CPR training is recommended. Subgroup analysis has revealed that whites were significantly more likely to receive CPR than other racial/ethnic populations.^60^ AED use was more common in neighborhoods with high median household income, <10% unemployment, and >80% high school education.^38^ Therefore, maximum benefit will be gained from targeting CPR training to groups of high socioeconomic need and ethnic minorities.
2. The burden of OHCA and the response of bystanders appears to vary according to ethnicity and socioeconomic status.^35,38,39,60^ Further investigation of these findings may result in targeted approaches to maximize outcome from investment when aimed at these communities.
3. Availability of AEDs has been shown to improve survival.^61–63^ Therefore, as the majority of cardiac arrests are witnessed, AEDs at schools, stadiums, stations, etc, may be expected to increase survival. Venues where delivery of AEDs by emergency services is unlikely (eg, trains, ships, planes) are of particular importance. Appropriately maintained equipment and appropriate training of potential AED users are an essential component of this strategy.

## Section 3 Multidisciplinary Team

### 3.1. Introduction

The investigation of SCD and resuscitated SCA requires input from a variety of different disciplines. The coordination and the communication between them mandate the formation of a multidisciplinary team. Numerous consensus statements agree on the importance of a dedicated combined cardiac genetic service in this setting.^2,11,65–67^

### 3.2. Key Features of an Effective Multidisciplinary Team

Certain key features can be identified in well-functioning multidisciplinary teams across specialties. Nancarrow et al.^68^ propose 10 key attributes including positive leadership and management, communication strategies and structures, appropriate resources, appropriate skill mix, and a supportive team climate with a focus on education of each other. There should be open communication and shared decision-making.

**Figure 7:**
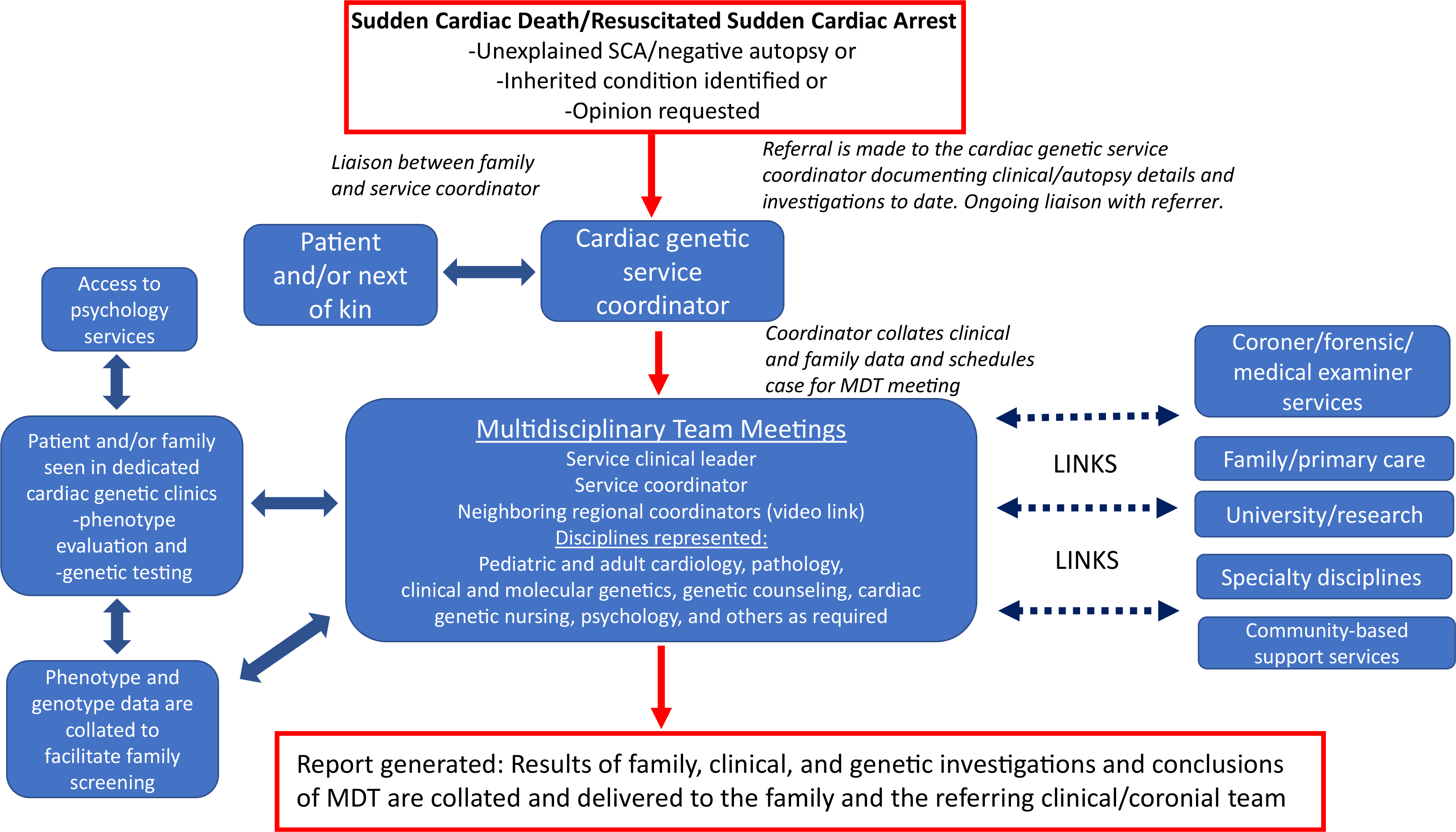
Referral flow for cardiac genetic investigation of sudden cardiac death (SCD) or resuscitated sudden cardiac arrest (SCA). MDT = multidisciplinary team.

**Figure 8:**
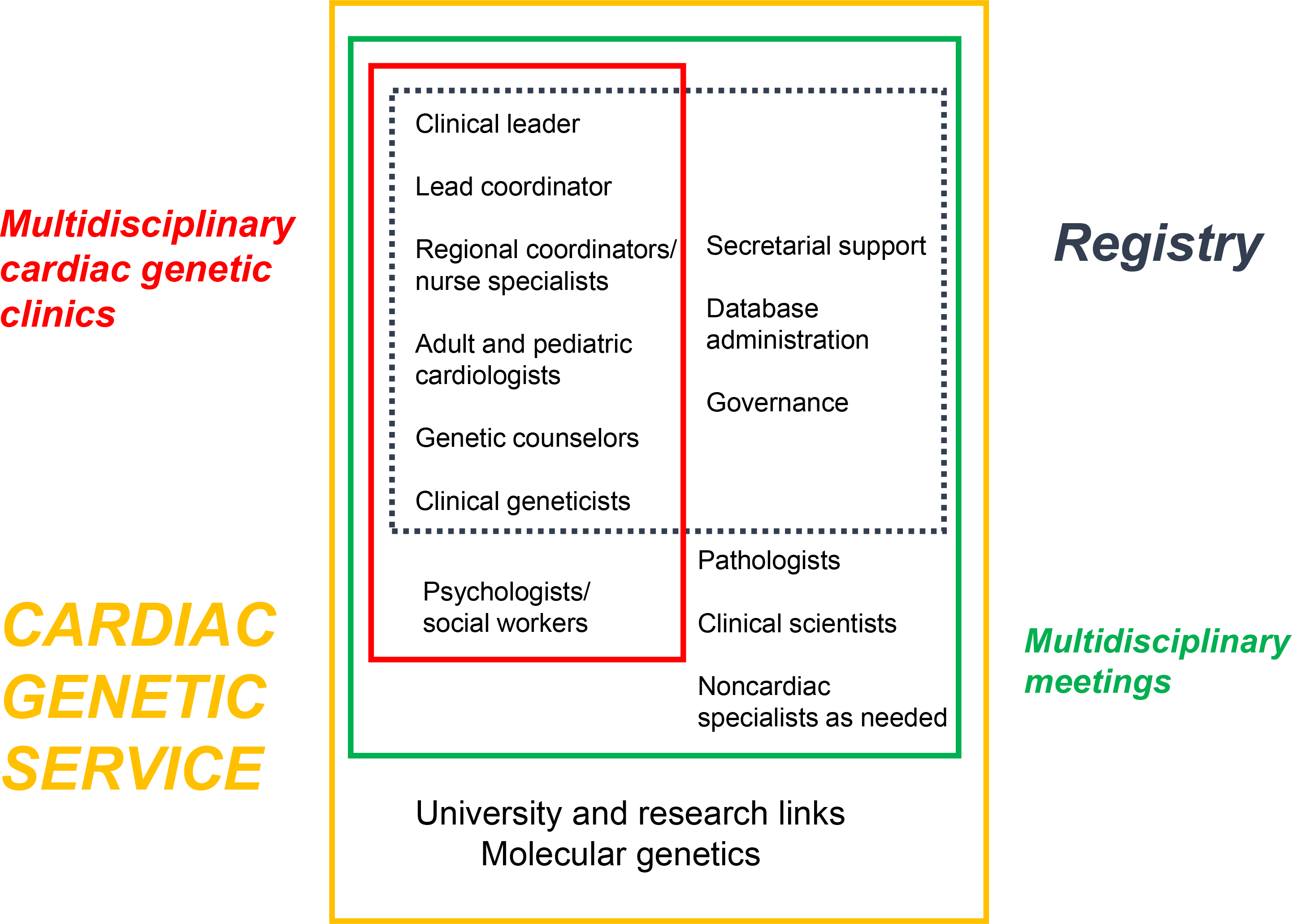
Participants in a cardiac genetic service. “Pathologists” includes forensic pathologists. Modified with permission from Elsevier.^69^

The detection of inherited heart conditions by pathologists and by hospital clinicians requires heightened awareness of their existence and a simple referral pathway to a multidisciplinary service with cardiac genetic expertise. Clinical experience shows that the appointment of a coordinator, as well as an enthusiastic team leader, is essential to facilitate this process, and regular meetings increase relevance and improve attendance^69^ (Figures 7 and 8).

### 3.3. Defining Which Disciplines Should Be Represented

The investigation of SUD is led by (forensic) pathology and the investigation of resuscitated SCA by pediatric or adult cardiology, with cardiac heart rhythm specialists and genetic cardiologists often being central. Clinical and molecular genetic specialists and genetic counselors are needed because of the significant role of molecular genetics in achieving a diagnosis and cascade screening, the consideration of multisystem genetic syndromes, and the high prevalence of genetic variants of uncertain significance.^70,71^ The high levels of psychological morbidity among SCA survivors and family members of both SCA survivors and decedents mandates access to psychology expertise,^72–74^ and input into the multidisciplinary team helps keep this in focus.^75^ The presence of a specialist cardiac genetic nurse in the cardiology inpatient setting increases detection of inherited cardiac conditions following SCA.^76^ Other clinical specialists can be helpful and be drafted in for certain cases—neurologists, pediatricians, metabolic specialists, and intensivists, for example.

### 3.4. Coordination Across Disciplines and Other Boundaries

The fact that the many disciplines may not be co-located highlights the importance of a coordinator to a multidisciplinary service. Co-location is not critical for effective collaboration, and non-co-location should not be an excuse for failed collaboration. A regional or institutional coordinator could be a nurse specialist, genetic counselor, or other allied professional and is vital to facilitate team meetings and communication between specialists and between centers, with primary care and across regions or between states and countries where necessary to facilitate family screening^69,77^ (Figure 7).

### 3.5. Links to Other Services

Links to other services as proposed in a recent scientific statement^66^ and practiced by some centers already^69^ include connections to molecular genetic expertise, researchers, primary health providers, between regions, and to a cardiac genetic clinical registry to facilitate family screening and follow-up across traditional boundaries (Figure 8).

Clinical and genetic registries are generally voluntary and consent-based and have a research element. We do not consider that they are compulsory. However, in this setting they do have particular relevance because many cases remain unresolved after the initial investigation and families may find comfort in knowing that efforts to find a diagnosis continue. The multidisciplinary team also provides a mechanism to revisit family members if new findings appear in the wider family or if the pathogenicity of a genetic variant is redefined.

**Table:** Recommendations for the role of a multidisciplinary team for investigation of SUD and SCA

COR	LOE	Recommendations	References
1	B-NR	1. The investigation of SUD and SCD due to a potentially heritable condition should be overseen by a multidisciplinary team with, as a minimum, appropriate expertise in pediatric and/or adult cardiology, genetics, genetic counseling, and pathology.	^78–82^
1	B-NR	2. The investigation of a sudden cardiac arrest survivor where a heritable condition is possible should be overseen by a multidisciplinary team with, as a minimum, appropriate expertise in pediatric and/or adult cardiology, genetics, and genetic counseling.	^79,82–85^

#### Synopsis

The cardiac and genetic investigation of SUD and resuscitated SCA should be overseen by a multidisciplinary team with appropriate expertise in this area. Recommendations include adequate resourcing, a dedicated coordinator, strong leadership, and a mutually supportive team that meets regularly.

#### Recommendation-Specific Supportive Text

1. For regions where coordinated cardiac genetic services exist that include the investigation of SUD, detection of inherited heart conditions is higher than in regions where they are not.^78^ Families prefer specialized clinics that combine co-located cardiac and genetic expertise and genetic counseling.^79^ Many such dedicated clinics internationally have led to the detection of inherited heart conditions following SCD and resuscitated SCA.^80–82^ It is therefore logical that multidisciplinary teams should have links to such clinics. Continued productive dialogue among pathology, coronial, police, and cardiac genetic services is recommended to improve the quality and relevance of forensic pathologists’ reports.^86^
2. Genetic testing in this context leads to a significant proportion of both pathogenic and unclassified variants, and precise evaluation of clinical phenotype is imperative for the correct assignation of such variants, so that a service that combines specialist cardiology and genetic expertise is essential.^83,84^ Specialized clinics that combine co-located cardiac and genetic expertise and genetic counseling are preferred,^79^ and such combined clinics have a high detection of inherited cardiac conditions following resuscitated SCA.^82,85^

## Section 4 Counseling Families, the Bereaved and the Nearly Bereaved

Genetic counseling is a process that aims to assist patients and their families to understand and adapt to the medical, psychosocial, and familial impact of inherited diseases.^87,88^ Genetic counseling goes beyond the discussion of genetic testing and is important for all patients with a genetic condition, at all stages of management.^89^ Although genetic counseling may be performed by any number of health professionals, genetic counselors are specifically trained in this role and have grown to a large allied health workforce worldwide.^90,91^ In some institutions, this role may be performed by a clinical/medical geneticist, genetic nurse, or other appropriately trained specialist.

In the setting of SCD or resuscitated SCA where a genetic cause is suspected, the inclusion of genetic counselors in the multidisciplinary team is widely advocated. The role of the cardiac genetic counselor includes taking a detailed family history, investigating and confirming details such as postmortem reports, providing education and awareness, assisting in coordinating family clinical screening, and providing psychosocial support.^74,92–95^ Throughout the process of genetic testing, genetic counselors provide important pre- and post-test genetic counseling, assist with interpretation of the results, help communicate this information to relatives, and assist with cascade genetic testing^75,93,96,97^ (Table 4).

Where there are significant emotional difficulties (see Section 5), the process of effectively conveying genetic information can be challenging.^98^ For families who have experienced a young SCD where a genetic cause is suspected, learning the potential inheritance risk to family members and need for clinical screening can add an additional stressor at a time of intense grief. Furthermore, with the increasing availability of postmortem genetic testing (see Sections 6.4 and 6.5), the need for complex genetic discussions with families is more commonplace.^99^ Genetic counseling prior to and after genetic testing is important, particularly where genetic test results are not straightforward such as identification of variants of uncertain significance or in the event of a variant reclassification.^100,101^

There is wide acknowledgment that genetic counseling as a process should go beyond just provision of information.^10,2,103^ The psychosocial aspects of genetic counseling include psychological support, empathic listening, crisis intervention skills, knowledge of family dynamics, coping models, processes of grief, and adjustment to disease diagnoses, all of which align with the core competencies of genetic counseling accreditation.^10,4,105^ Attending to the psychosocial needs, in addition to provision of education and information, has been demonstrated to positively impact patient outcomes, largely based around knowledge and recall, but healthy adjustment, empowerment, behavioral change, and satisfaction with decision-making also reduce anxiety and worry.^103,104,106,10,7^

**Table:** Recommendations for counseling families affected by SUD and SCA

COR	LOE	Recommendations	References
1	B-NR	1. Genetic counseling is strongly recommended for all families where there has been an SUD or resuscitated SCA and a heritable cause is suspected, and should include antemortem and postmortem data collection and evaluation, so that risks, benefits, results, and the clinical significance of genetic testing can be discussed.	^80–82,84,85,103,106,107,109^
1	C-EO	2. It is recommended that genetic testing in families where an SUD or resuscitated SCA due to a heritable cause is suspected is performed only with appropriate genetic counseling.	

### Synopsis

Genetic counseling of patients and their families with genetic conditions is recommended, including those with SCD or resuscitated SCA where a genetic cause is suspected. Key aspects of the process include discussion of inheritance risks, education and awareness, pre- and post-test genetic counseling, interpretation of genetic results, taking a family history, coordination of clinical screening, and psychosocial support. Genetic counseling is focused on both information provision and psychosocial support and together has been shown to improve knowledge and recall; promote healthy adjustment, empowerment, and behavioral change; increase satisfaction with decision-making; and reduce anxiety and worry. While genetic counseling is a process often performed by a variety of health professionals, ideally a specifically trained genetic counselor or genetic nurse with appropriate skills in information provision and psychosocial support would perform this role.

#### Recommendation-Specific Supportive Text

1. Genetic counseling includes both information provision and psychosocial support. It is ideally performed by health professionals with specific training and experience; this includes genetic counselors, genetic nurses, or other qualified health professionals. ^80–82,84,85,103,106,107,109^
2. In the context of genetic testing, pre- and post-test genetic counseling must be performed.^75,95,96,99^ In cases where there is uncertainty in the findings, such as a variant of uncertain significance or a variant reclassification, this is of particular importance.^100,101^

## Section 5 Psychological Care

SCD where a genetic cause is suspected has a profound psychological impact on the surviving members of the family. Grief is a normal emotional response to the loss of a loved one. Individuals will grieve differently, and while there is no single trajectory, many will experience disbelief, yearning, anger, sadness, and acceptance.^117^ After a death, an individual will not return to normal, but rather create a revised meaningful life without the deceased. In a small proportion of bereaved individuals, the initial grief response does not resolve and may result in prolonged grief, or persistent complex bereavement disorder according to the Diagnostic and Statistical Manual of Mental Disorders, 5th Edition (DSM-5).^118,119^ This occurs in approximately 7% of the general bereaved population,^120^ and in 21% of first-degree relatives following SCD in the young.^121^ Posttraumatic stress symptoms can also be experienced by family members. Posttraumatic stress occurs in response to a specific trigger, typically one that threatens one’s own or a loved one’s well-being. It is characterized by avoidance with hyperarousal and intrusive thoughts, including persistent and extreme fear and panic similar to that experienced by family members at the time of the event.^122^ Posttraumatic stress has been shown in 44% of first-degree relatives following a young SCD.^121^ Individuals with prolonged grief and/or posttraumatic stress symptoms can benefit from intervention with a clinical psychologist or other appropriately trained clinicians, and there is extensive evidence to support the efficacy of psychological treatments for these conditions in other settings.^120^ Further, there is greater risk of other psychiatric comorbidities,^118^ suicide,^123^ and development of chronic medical conditions.^124^

**Figure 9:**
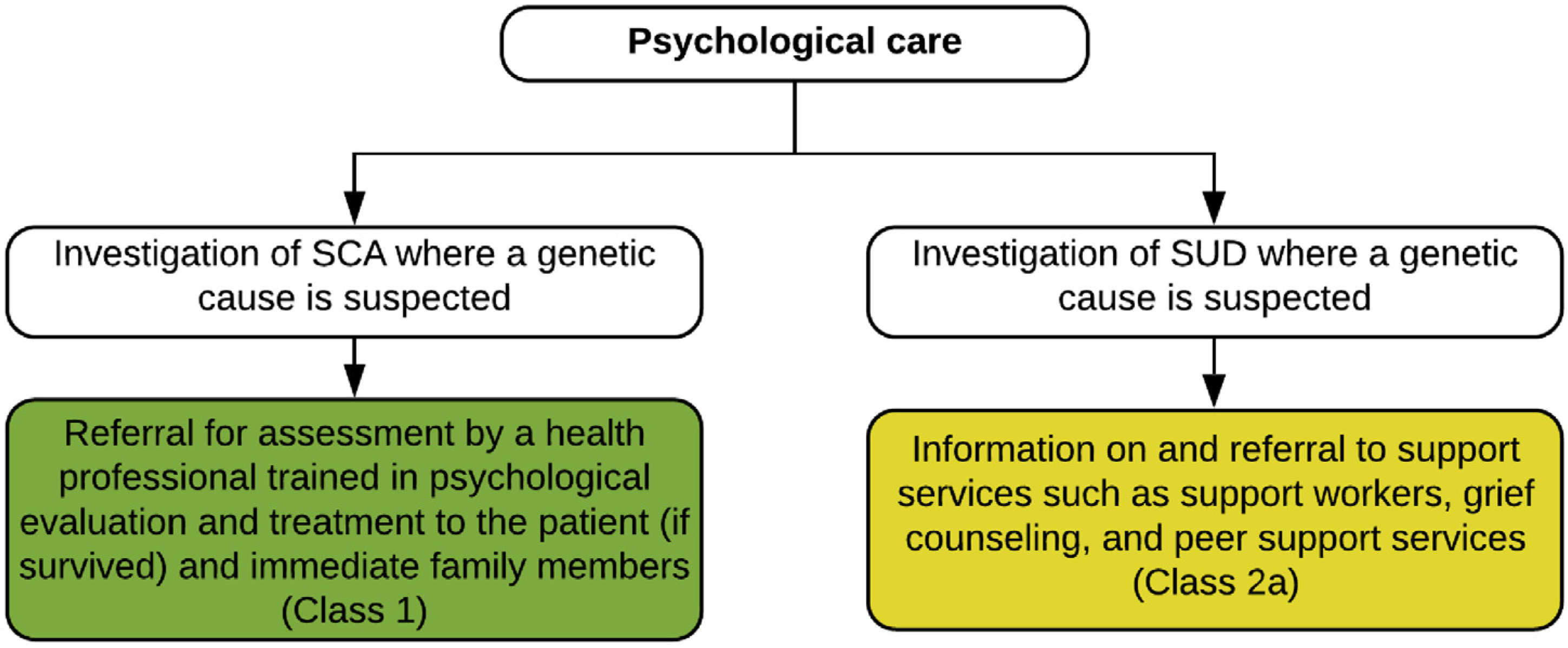
Psychological care following a sudden cardiac arrest (SCA) or a sudden unexplained death (SUD) where a genetic cause is suspected. Colors correspond to the Class of Recommendation in Table 1.

Factors associated with poor psychological outcomes have been investigated. One study showed that mothers of the deceased were more likely to report anxiety and depression symptoms.^72^ In total, 53% of the mothers surveyed reported probable anxiety disorder on average 4 years after the death. In a larger study, after adjusting for factors including relationship to the decedent, those family members who witnessed the death or discovered the decedent’s body had a 3-fold risk of posttraumatic stress symptoms (OR 3.3, 95% CI 1.2–8.7, *p* = 0.02) and a 4-fold risk of prolonged grief (OR 4.0, 95% CI 1.3–12.5, *p* = 0.02).^121^ Given that half report symptoms indicating psychological difficulties, all first-degree relatives should be offered psychological evaluation and treatment. Although the evidence for psychological support is derived from studies investigating SCD where a genetic cause is suspected, it may logically apply to those individuals who have survived SCA and their families (Figure 9).

There may be initial reluctance to seek psychological support given community stigma around mental health. Indeed, a recent study investigating families who had experienced a young SCD found that only 12% had sought psychological support, with most of those being self-referrals.^125^ In discussing options for ongoing psychological support with patients and families, normalizing their response to a significant psychological stressor and describing common symptoms of prolonged grief and posttraumatic stress may reduce any perceived sense of stigma and increase interest in seeking support.

A recent needs analysis of parents who had experienced the SCD of their child (including adult children) found that while medical information and support were the most important need, psychological information and support were the most unmet need.^126^ Nearly three-quarters reported wanting access to professional counseling or psychological services. Further, many indicated access to genetic testing or understanding the genetic cause to be an important need, highlighting the importance of maintaining realistic expectations regarding the diagnostic yield of postmortem genetic testing with families.^101^ At present, this is likely not greater than 15%, and there is a high likelihood of uncertain genetic findings especially with increasing gene panel sizes.^25,70^ A Swedish study of parents whose children died suddenly between 15 and 35 years of age likewise showed a critical lack of information and support in the acute grief stage.^127^ This included a need for better communication of the postmortem examination process (how long it would take, when they would get results), time with a health professional to discuss the death, and information about the cause of death. There was a lack of psychological support in the immediate aftermath, with many family members seeking their own care, including grief counselors and support groups. The need for support in the early aftermath has been shown to be important in other studies examining suddenly bereaved parents.^128,129^

Community or peer-based bereavement support groups can also enhance social support.^120,130^ Peer support programs come in many different forms but always involve people with similar backgrounds providing emotional, social, or practical support to each other.^131^ Peer supporters draw on their shared experiences to provide empathic understanding, information, and advice to those they are helping. A key aim is to promote hope, recovery from illness or trauma, improved life skills, psychological well-being, and social integration.^132^ A recent systematic review of peer support services for bereaved survivors of the sudden death of a loved one in multiple settings found evidence of reductions in grief and increased well-being and personal growth among participants, and improved personal growth and positive meaning in life among peer providers.^133^ There is a current gap in care in addressing psychological support needs of families after the SCD of a young relative.

**Table:** Recommendations for psychological care

COR	LOE	Recommendations	References
1	B-NR	1. In the investigation of SCA where a genetic cause is suspected, it is recommended that referral be offered for assessment by a health professional trained in psychological evaluation and treatment to the patient (if survived) and immediate family members.	^72,121,126,134^
2a	C-LD	2. In the investigation of SUD where a genetic cause is suspected, provision of information and referral to support services such as support workers, grief counseling, and peer support services can be useful.	^127,131–133^

### Synopsis

The psychological impact to the family following an SCD where a genetic cause is suspected can be significant. Although many family members will navigate their way through this traumatic experience, up to 44% may require additional psychological support from an appropriately trained health professional such as a clinical psychologist. Addressing community stigma around mental health needs to be considered and discussed with families. In addition, support services such as social workers, grief counselors, psychosocial teams, and peer support groups may be useful to many families. Whereas the evidence for psychological support is derived from studies investigating SCD where a genetic cause is suspected, it may logically apply in those families where there has been an SCA.

### Recommendation-Specific Supportive Text

1. and 2. A clinical psychologist or appropriately trained health professional includes those equipped to assess and treat trauma; for example, those experienced in delivering cognitive behavioral therapies. While the evidence to date supports a need for psychological support in family members following a young SCD where a genetic cause is suspected, this may likewise be important for relatives of a patient who suffers an SCA.^72,121,134^ There is a need to train personnel in psychological care for SUD and SCA, as this is an area where the need is not currently met.

## Section 6 Investigation of Sudden Death

### 6.1. Investigation of Sudden Death: History—Personal and Family

**Figure 10:**
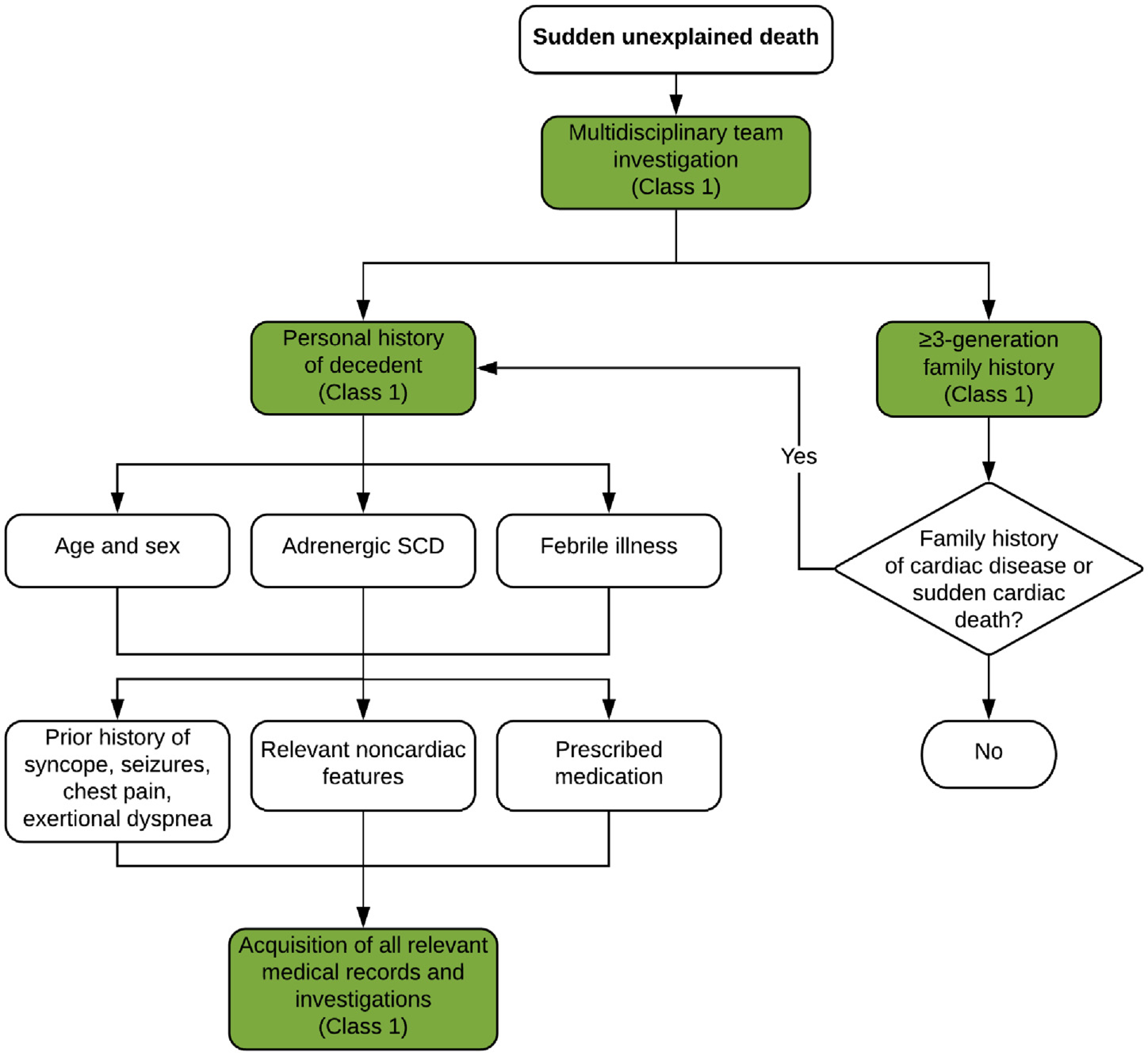
Investigation of sudden unexplained death: personal and family history. Colors correspond to the Class of Recommendation in Table 1. SCD = sudden cardiac death.

Despite being “low-tech” and inexpensive, the history, as a tool for clinical phenotyping, is the essential and fundamental basis of approaching a patient with SCA because it can guide appropriate use and interpretation of other diagnostic modalities. The history should be focused toward both the decedent proband and also the wider family for evidence of other potentially affected members prior to investigations. Surviving family members should be investigated by a multidisciplinary team within a specialist program for cardiovascular genetic disorders with the all appropriate medical, genetic, and psychological personnel and ability for comprehensive investigations^2,10^ (Figure 10).

The proband age may help define potential etiologies; CPVT and long QT syndrome are typically diseases of the young, whereas coronary artery disease and cardiomyopathies become more common with age (Figure 1). Although most deaths occur at rest or during sleep,^25^ death during exertion may point to specific etiologies such as CPVT, long QT syndrome type 1, or arrhythmogenic cardiomyopathy. In addition to a detailed prior medical and medication history (including potential drugs of abuse), the decedent’s health in the 24–48 hours preceding death including the presence of any viral prodrome or fever, as well as any prescribed medication, may be relevant. Myocarditis secondary to viral infection may be associated with viral and gastrointestinal symptoms, and both Brugada syndrome and long QT syndrome may be exacerbated by specific pharmacological agents through further inhibition of ion channel function.^135^ Fever is a well-recognized trigger of ECG changes and arrhythmia in Brugada syndrome^136^ and in some long QT syndrome subtypes,^137^ and in young children may be misdiagnosed as febrile seizures.^138^

Between 18% and 45% of sudden death cases may have experienced prior relevant symptoms, typically palpitations, chest pain, pre-syncope, or syncope, and may have undergone relevant investigations.^29,70,139^ All medical records relevant to the sudden death etiology should be sought.

Relevant information from the family history should be collected by a health professional with specific experience in cardiovascular genetic disease (preferably a genetic counselor) and by an appropriately trained cardiologist. Symptoms and diagnoses in other family members as well as prior cardiovascular investigations should be sought. Noncardiac findings may be highly pertinent including unexplained epilepsy unresponsive to conventional therapy; skeletal muscle weakness; curled hair and subtle palmoplantar hyperkeratosis/keratoderma (arrhythmogenic cardiomyopathy)^140^; attention deficit disorder and intellectual disability (CPVT)^141^; and history of pneumothoraces, vascular disease, and gastrointestinal and uterine rupture (vascular Ehlers-Danlos syndrome).^142^ Any other deaths or major cardiac events in the family should be recorded including those related to drowning in good swimmers, unexplained motor vehicle accidents, and sudden infant death or late fetal demise. If the SUD was observed, it is useful to collect witness accounts about the events occurring immediately prior to the collapse and during any resuscitation attempts.

**Table:** Recommendations for investigation of sudden death: personal and family history

COR	LOE	Recommendations	References
1	B-NR	1. In the investigation of SUD, an effort should be taken to obtain detailed personal and three-generation family history (as a minimum) with the assistance of a multidisciplinary team, including witness accounts.	^25,29,70,77,81,135,139,143,144^
1	B-NR	2. In the investigation of SUD, prior medical records and relevant investigations from the decedent proband and family members should be retrieved.	^25,29,70,77,81,135,139,143–145^

#### Synopsis

The personal medical and three-generation family history provides the initial information on which subsequent investigations will be based. Specific features within the wider family may suggest diagnoses and help direct subsequent investigation. The history should be recorded by cardiologists, specialist nurses, and geneticists or genetic counselors experienced in cardiovascular genetic diseases, ideally within the confines of a multidisciplinary program that can address the medical, genetic, and psychological needs of the family (see Section 3).

#### Recommendation-Specific Supportive Text

1. The personal and three-generation family history may provide critical information relevant to the etiology of SCD and provide a starting point for further investigations in both the decedent proband and surviving family members.^77^ Multiple studies show a significant proportion of children and adults experience relevant cardiac symptoms prior to sudden death,^10,25,29,70,135,138,139^ and some may have sought medical attention and undergone investigations. Available ECGs and cardiac imaging, together with autopsy findings, may allow a diagnosis to be made (or excluded) in the proband who, until family investigations have been performed, is the one definitively affected member of the family. Noncardiac features and symptoms may also provide important diagnostic information.^140–142^
2. Further investigation is necessary where sudden death occurs in specific circumstances such as when a cardiac event may have triggered an apparently environmental death. Examples include road traffic accidents with no apparent cause and drowning in competent swimmers.

The presence and associated investigations for other noncardiac conditions should also be evaluated, specifically epilepsy. Failure to identify a neurological etiology or abnormality would suggest seizures may have had a cardiac etiology. Overlap syndromes exist between true neurological epilepsy and long QT syndrome type 2.^146,147^

### 6.2. Investigation of Sudden Death: Examination of Premorbid Investigations

Individuals who have succumbed to SUD may have had pertinent investigations prior to their death that aid in the diagnosis of the cause of their SUD. Twelve-lead electrocardiogram (ECG) is the most useful pre-SUD investigation. Although long or short QT interval, spontaneous type 1 Brugada pattern, and early repolarization pattern are associated with fatal arrhythmias due to congenital long QT syndrome, short QT syndrome, Brugada syndrome, or early repolarization syndrome,^148–152^ many patients with SUD without structural heart disease have a normal or near-normal ECG,^145,153^ particularly women.^154^ Additional ECG findings suggestive of arrhythmic syncope include bifascicular block; intraventricular conduction abnormalities (QRS duration >0.12 s); Mobitz I second-degree atrioventricular block and first-degree atrioventricular block with markedly prolonged PR interval; sinus bradycardia (<40 bpm) or slow atrial fibrillation (<40 bpm); nonsustained ventricular tachycardia; pre-excited QRS complexes; negative T waves in right precordial leads or epsilon waves; and left ventricular hypertrophy,^5^ any of which may indicate potential diagnoses of inherited arrhythmia syndromes such as progressive cardiac conduction defect, familial pre-excitation, arrhythmogenic cardiomyopathy, or hypertrophic cardiomyopathy.^4,151^ In the general population, premature ventricular complexes (PVCs) are mostly benign; however, some frequent or complex PVCs significantly increase the risk of SCD.^155–157^ If an ECG is recorded by the AED or EMS just before SCD, features such as J-wave or ST segment elevation (especially if augmented after a long pause) may help in the diagnosis of coronary spasm, early repolarization syndrome, or Brugada syndrome.^158,159^ Interpretation of ECGs obtained immediately after resuscitation/defibrillation should be performed with great caution (see Section 7.4).

Syncope is a sentinel clinical symptom before SUD and may prompt investigations subsequently useful in making a retrospective diagnosis of the cause of SUD. In particular, the trigger for the syncopal event bears useful information. Ambulatory ECG monitoring during life may provide clues to the cause of SUD and should be sought.

If transthoracic echocardiography, cardiac computed tomography (CT), or cardiac magnetic resonance imaging (CMR) are performed during the patient’s life, detailed review may indicate features of dilated cardiomyopathy, hypertrophic cardiomyopathy, or arrhythmogenic cardiomyopathy.^160^ If blood or other tissue sample has been taken before SUD, this may be a source of DNA for genetic testing, should there not be a postmortem collection of tissue.^8,11,67,15,1^ Neurological findings such as developmental delay or seizures thought to be suspicious for epilepsy during life may contribute to a diagnosis of a cardiac channelopathy, such as CPVT or long QT syndrome.^141,161,16,2^ If a patient with SUD has a cardiovascular implantable electronic device (CIED) implanted, postmortem interrogation of the CIED is useful to determine the cause and timing of SCD.^163^

**Table:** Recommendations for investigation of sudden death: examination of premorbid investigations

COR	LOE	Recommendations	References
1	B-NR	1. All relevant cardiac investigations, including 12-lead ECGs, echocardiography, CT, CMR, genetic analyses, and ambulatory monitoring recorded before SUD, should be reviewed and analyzed.	^145,148–150,153–158,164^
1	B-NR	2. Any blood or DNA sample (eg, blood in EDTA, blood on filter paper card) taken before SUD should be stored for future genetic analysis.	^84,165–167^
1	B-NR	3. Neurological events such as seizures suspicious for epilepsy before SUD should be reviewed and studied for a potential cardiac etiology.	^70,146,147,161,162,168,169^
2b	C-LD	4. ECG information from the AED or ECG monitor recorded around the time of SCD may be useful for review and analysis.	^158,170^
1	B-NR	5. Any implanted cardiac electronic device in an individual with SCD should be reviewed and analyzed.	^163,171,172^

#### Synopsis

During the investigation of SUD, pertinent investigations performed prior to death can aid in establishing the cause. Although ECG features such as QT interval, type 1 Brugada pattern, and early repolarization may be critical for diagnosis, many ECGs taken during life will be normal. Ambulatory ECG monitoring and cardiac imaging should be sought to provide clues to the diagnosis of SCD. Symptoms attributed to a neurological cause may be re-evaluated, in collaboration with neurologists. Any potential DNA sample before SUD should be stored if tissue is not gained at autopsy. ECG information from the AED, emergency services, or CIEDs may also be useful to determine the cause of SCD.

#### Recommendation-Specific Supportive Text

1. Investigations during life may provide clues to the cause of SUD and should be sought to aid in diagnosis.^173^ These include resting, exercise and ambulatory ECG tracings, and cardiac imaging studies (echocardiography, CT, and CMR).^145,148–150,153–158,164^
2. Samples for potential DNA testing taken during life may subsequently prove invaluable should they be the only source of DNA. Although it is a Class 1 recommendation that patients with SUD have an autopsy and material for DNA testing collected (see Section 6.3), it is recognized that sometimes this is not done, making blood or tissue samples taken during life the only remaining source for molecular autopsy.^84,165–167^ Using such samples requires appropriate consent from family, unless ordered by the coroner. It should be recognized that success varies depending on the storage method, but attempts to gather useful DNA may be worthwhile even from suboptimal sources. Future extraction methods may improve the yield so continued storage is advisable.
3. Symptoms such as seizures thought to be suspicious for epilepsy during life may in fact be attributable to a cardiac channelopathy when further investigation is done. Other neurological findings such as developmental delay hold significance for diagnoses such as CPVT. Thus, meticulous recording of neurological events during life may lead to a diagnosis in SUD.^70,146,147,161,162,168,169^
4. Recordings from emergency services continuous ECG monitoring or from interrogation of AEDs, when available, may provide clues to the etiology of SUD. However, it should be acknowledged that a finding of ventricular fibrillation is often due to this rhythm being a final common rhythm in arrhythmic death, regardless of the initial rhythm causing hemodynamic collapse. Nevertheless, at points such as reinitiation of arrhythmia and glimpses of normal rhythm in between arrhythmia may suggest a specific diagnosis.^158,170^
5. The memory function of CIEDs may reveal the initiation pattern of cardiac arrhythmia and aid in the diagnosis of SCD. Therefore, if an SUD victim has a CIED implanted in life, interrogation of this device can provide useful clues in the diagnosis of SCD.^163,171,172^

### 6.3. Investigation of Sudden Death: The Postmortem Examination and Imaging

**Figure 11:**
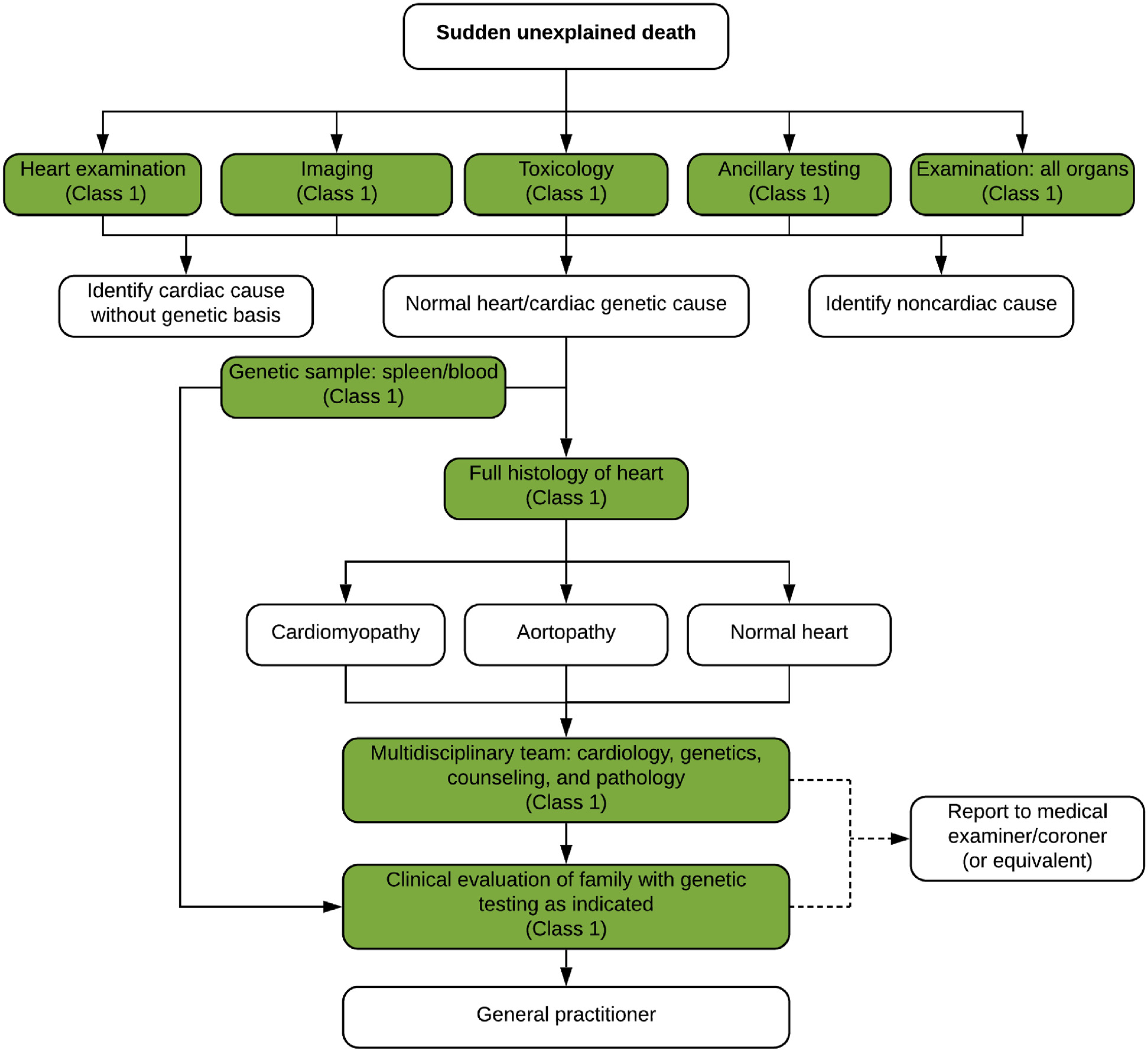
Investigation of sudden unexplained death: the postmortem examination and imaging. Colors correspond to the Class of Recommendation in Table 1.

The critical components to the investigation of SUD include examining the circumstances of the death and the autopsy (Figure 11). Identification of SUD relies on the reporting of EMS, police, hospitals, and witnesses. Investigation of a death is determined by the jurisdiction in which the death occurs.^14,174,175^ Unexpected or unexplained deaths, when the individual was in apparent good health, should be carried out by a trained pathologist who has a thorough knowledge of cardiac pathology.^176^ Autopsies vary not only by country but also by individual jurisdictions within countries. The autopsy should be comprehensive, examining all organs and conducted in a systematic and objective method with a focus on standardized reporting.^2,67,177,178^ Cases should be referred to a cardiac pathologist when a cardiac cause is suspected.^2,176^

Imaging includes X-rays and photography. Photography is useful in providing documentation of syndromic features and highlighting individual organ pathology. Postmortem CT and magnetic resonance imaging (MRI) have been shown to be useful^179^ but are not universally available. Noncardiac causes should be looked for including infection, thromboembolism, tumors, intracerebral lesions, respiratory disease, and abdominal causes such as ruptured abdominal aneurysm. Body mass index should be recorded along with waist circumference.

Ancillary testing should be performed including microbiology/cultures for infectious disease, metabolic screening (particularly in younger children), toxicology, vitreous testing for biochemistry, genetic testing (see Sections 6.4 and 6.5), and other testing as indicated by the autopsy findings. Taking a sample for toxicology is recommended in all sudden unexpected deaths.^180^

Samples for genetic testing should be saved at the time of autopsy from every sudden death case.^2,181^ Ideally, two of the following three should be saved: a small piece of fresh frozen heart, a small piece of fresh frozen spleen/liver/thymus, and EDTA blood. If RNAlater (ThermoFisher Scientific, Waltham, MA, USA) or similar reagent to preserve DNA at room temperature is available, fresh tissue can be transported in this to the referral genetic center without need for freezing.

The heart should be examined thoroughly^67^ and at least 7–10 samples taken for histology. Cardiovascular disease is the leading cause of sudden death in the young and is divided into two major groups: morphologically positive (eg, congenital heart disease, coronary artery disease, and cardiomyopathy) and morphologically normal hearts. Combined with negative toxicology, those with morphologically normal hearts have been labeled as having “autopsy-negative sudden unexplained death” or “sudden arrhythmic death (syndrome) or SAD(S).”^182^ Samples should always be taken, even from a macroscopically normal heart, as histology may reveal inflammation and cardiomyopathies. Always consider sudden unexpected death in epilepsy (SUDEP) and sudden death in alcohol misuse (SUDAM)^183^ where clinical history and circumstances are important. Pathologists and clinicians should not overinterpret findings in the heart at autopsy such as nonsignificant coronary artery disease, etc.^184^

**Table:** Recommendations for investigation of sudden death: the postmortem examination and imaging

COR	LOE	Recommendations	References
1	B-NR	1. An autopsy is strongly recommended in individuals with an SUD.	^14,25,37,175–177^
1	B-NR	2. Autopsies for SUD should be comprehensive, including photography, imaging, toxicology, gross examination of all organs, and detailed examination of the brain, heart, and thorax, with histology being essential.	^14,175–177,180^
1	B-NR	3. EDTA blood and/or one type of fresh tissue (heart, liver, spleen, skeletal muscle) should be saved at autopsy for SUD and banked at −20°C or −80°C for potential genetic analysis; two sources are ideal, if possible.	^25,70,166,185^
2b	C-LD	4. Storing frozen myocardial tissue may be considered at autopsy for SUD, as it may aid in assessing the significance of future genetic findings.	^186,187^
1	C-EO	5. Findings of an autopsy for SUD should be communicated to the family in a timely fashion in accordance with local legal requirements.	
1	B-NR	6. Cases with likely cardiac causes for SUD should be referred to a pathologist with expertise in cardiac disease, as the finding of an abnormal or normal heart is important for family screening.	^176,177,184^
1	C-LD	7. When an autopsy for SUD reveals a possible genetic cause, or the heart is normal, then referral for clinical and genetic investigation of the family is recommended.	^80,81,143,188,189^

#### Synopsis

A comprehensive autopsy is an essential part of the investigation of SUD and should include collection and storage of tissue suitable for genetic analysis. When the autopsy suggests a possible genetic cause, or no cause and the heart is normal, referral to a multidisciplinary team for further investigation is indicated (see Section 3).

#### Recommendation-Specific Supportive Text

1. SUD should have an autopsy done by a trained pathologist.^174–176^ Studies have shown that autopsies performed by pathologists who have a thorough knowledge of cardiac pathology have a superior diagnostic yield.^174–176^ In cases where autopsy is not possible (eg, for religious reasons), a full body MRI or CT scan is recommended.^179^
2. Samples should be taken for infection and toxicology. Histological sampling of all important organs especially the heart (from multiple sites) is essential even when macroscopically normal.^174,175^
3. Blood or tissue suitable for DNA extraction and postmortem genetic testing should be obtained at all autopsies.^176^ Following the initial investigation, DNA should be extracted and banked if genetic disease is suspected or if the cause remains unknown.^2,176^ Ideally, lack of cost coverage should not be a reason not to comply with these recommendations.
4. Frozen myocardial tissue may be useful for subsequent RNA analysis or expression studies of aberrant proteins.^2,67^
5. Cause of death should be discussed in a multidisciplinary meeting (see Section 3) and provided by a pathologist to the medical examiner/coroner. The findings and follow-up recommendations should be communicated to the family.^178,190,191^
6. Finding of an abnormal or normal heart is important for family screening and directs much of the subsequent investigation (see Sections 6.4 and 6.5).
7. Autopsy phenotype should be established at a multidisciplinary meeting of pathologist, medical examiner, cardiologist, and clinical geneticist.

### 6.4. Investigation of Sudden Death: Genetic Evaluation Where the Phenotype Is Known

Genetic evaluation may be appropriate following SCD in two scenarios: most commonly, where the deceased individual is the proband with no prior medical history, or alternatively the deceased is part of a family where diagnosis is established but he/she has not yet undergone genetic evaluation. Initially, pathological examination should be performed by an experienced pathologist to ensure that all cardiac and extracardiac features relevant to the potential diagnosis are recognized (see Section 6.3).

**Figure 12:**
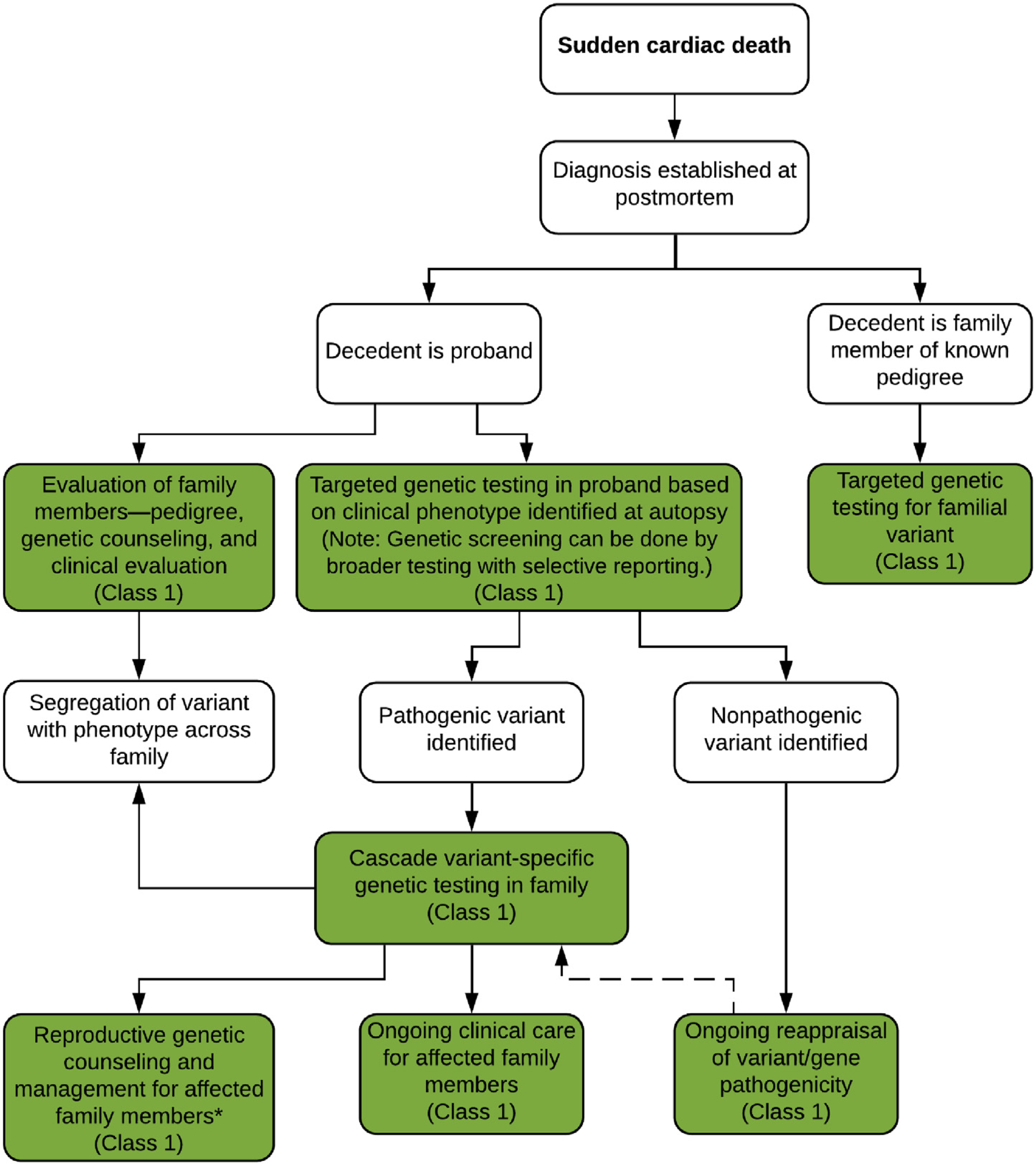
Clinical and genetic evaluation after sudden death where a phenotype is known. Colors correspond to the Class of Recommendation in Table 1. *See Section 8.2.

In cases where the deceased is the proband and a postmortem diagnosis is established, identification of a pathological variant may facilitate genetic testing in the wider family evaluation. Genetic testing of DNA from the deceased proband may be performed directly after autopsy or deferred until first-degree family members have been clinically evaluated (Figure 12). As part of familial evaluation, a three-generation pedigree (at a minimum) performed by a practitioner knowledgeable in the genetics of cardiovascular disease (eg, a genetic counselor or specialist nurse) is mandatory and should cover all potentially relevant cardiac and extracardiac features within the family (see Section 6.1). Genetic testing of deceased individuals may not be covered by health insurance in certain countries; in this instance, using a clinically affected family member as the testing proband with confirmatory testing in the deceased may be a more feasible strategy. Clinical and genetic testing in the proband and multiple family members will define segregation of the identified genetic variant(s), adding to the validity of the genetic findings. The yield of genetic testing in cases where a diagnosis of cardiomyopathy is established postmortem is significantly greater than where structural changes are uncertain.^192^

In cases where no other family members are clinically affected and the deceased proband is an apparently isolated case, genetic testing can be used as evaluation of the single definitely affected individual within the family. Families should be counseled about the expected benefits and potential outcomes of genetic investigations prior to testing. If identified variants are considered likely pathogenic or pathogenic, cascade testing across the family can be considered to identify at-risk individuals with no current clinical features.

In cases where the deceased is part of a family with a prior diagnosis of cardiovascular disease and known pathological variant, confirmatory genetic testing may be performed.

**Table:** Recommendations for investigation of sudden death: genetic evaluation where the phenotype is known

COR	LOE	Recommendations	References
1	B-NR	1. For SCD where the phenotype is suspected to be heritable, genetic testing is recommended to attempt to elucidate the genetic basis and to facilitate the identification of first-degree family members at risk for developing the same disease (cascade testing).	^25,80–82,84,85,143^
1	C-LD	2. Genetic testing in the deceased proband with SCD and known phenotype should include only genes with robust evidence of gene–disease association.	^192^
1	B-NR	3. In first-degree relatives of a proband with SCD from a suspected heritable cause, phenotype-guided clinical screening is recommended and, where a genetic diagnosis is available, cascade genetic testing should be offered.	^25,80–82,84,85,143,189,193^
1	B-NR	4. In families affected by SCD who have undergone genetic testing, periodic re-evaluation of the genetic test results is recommended.	^115,194–201^
1	C-LD	5. A genetic diagnosis made in a relative of a proband with SCD should be considered together with the clinical findings.	^70,143,189^

#### Synopsis

A postmortem diagnosis following SCD significantly facilitates further clinical evaluation of family members and may provide an explanation for the family as to the underlying etiology. Genetic testing targeted toward the clinical diagnosis and phenotype is an important component of the overall evaluation of both the proband and family and provides additional support to the clinical diagnosis. Further investigations can be performed as clinically indicated. In cases where no other individuals within the family are clinically affected, identification of a definitively pathogenic variant in the proband facilitates cascade genetic testing in family members.

#### Recommendation-Specific Supportive Text

1. Genetic testing in the decedent proband is recommended to support the clinical diagnosis and facilitate cascade genetic testing within the family.^10,11^ The yield of genetic testing is significantly higher when associated with a specific postmortem diagnosis.^84,192^
2. If a cardiac phenotype has been identified in the deceased proband, genetic testing should be targeted toward that specific phenotype to maximize the chances of a clinically actionable result and minimize the risk of ambiguous secondary findings. Targeted panels for cardiomyopathy, channelopathies, familial thoracic aortic aneurysm, and familial hyperlipidemia are the preferred option. However, given the limited availability of proband DNA and potential financial implications, broader genetic testing including whole exome and genome with selective reporting based on the phenotype may be considered. Robust evidence for gene–disease association is currently probably only available in the Clinical Genome Resource (ClinGen) expert panels for long QT syndrome, Brugada syndrome, hypertrophic cardiomyopathy, arrhythmogenic cardiomyopathy.^195,196,202^ Wider panels and whole-exome sequencing/whole-genome sequencing share the potential disadvantage of a high burden of variants of uncertain/unknown significance, but in experienced hands (ie, expert centers), this will not lead to unintended follow-up.^203^
3. Clinical evaluation for family members of a proband with an evident or likely phenotype can be targeted based on that phenotype, although due to varied expressivity and overlap syndromes should be sufficiently broad to provide a comprehensive cardiovascular evaluation. If a pathogenic or likely pathogenic variant has been identified in the deceased proband in a gene consistent with the clinical diagnosis, genetic counseling and testing should be offered to family members.^25,80–82,84,85,143,189,193^ Cascade testing for likely pathogenic variants should be done at the discretion of an experienced provider after reviewing the data. In cases with limited supporting data from the family, a more conservative approach may be appropriate.
4. Serial re-evaluation of variants should be performed based on new phenotype data in the family pedigree, or new data regarding both specific variants and whole genes, the pathogenicity of which may have been up- or downgraded based on contemporaneous evidence.^115,194–196,204^ Responsibility for re-evaluation is unclear, but the re-evaluation is best carried out in a center of expertise.
5. Genetic variants identified in deceased probands and subsequently in family members should be correlated with clinical findings to determine segregation patterns within the family.

### 6.5. Investigation of Sudden Death: Genetic Evaluation Where the Phenotype Is Unknown

SCD may occur in an individual without any prior medical history or medical data. As indicated above, a three-generation pedigree including examination of all potentially relevant cardiac and extracardiac features within the family should be performed by a cardiologist experienced in genetic heart disease or clinical geneticist experienced in cardiology in an effort to optimize the identification of subtle clinical features before defining the cause of death as unknown. Specific triggers (eg, competitive athlete, emotional or physical stress, drug use, swimming, acoustic triggers, seizure) leading to the SCD event may help focus clinical and genetic investigation. Collection of blood and/or suitable tissue for molecular autopsy/postmortem genetic testing is recommended in all victims of SUD.^2,10^ Local protocols are recommended to ensure proper handling and extended storage of biosamples, as well as processes and consent allowing for contact of families for genetic testing in the future.

Family members of sudden death victims where the phenotype remains unknown should be instructed to request re-evaluation of the index event in case of future developments in the family. Clinical signs or symptoms in first-degree family members leading to a suspected phenotype of a sudden death victim may occur over many years and sometimes skip generations, so an unknown phenotype might prompt more extensive questioning of older family members.

In an SCD case where the phenotype remains unknown after expert evaluation, re-evaluation of family members to assess for new information that may impact diagnosis should be performed periodically, although the yield is low.^205^ We suggest every 3 to 5 years, but shorter intervals should be considered if there is more than one SCD event in the family. Periodic re-evaluation should be stopped for individuals after age 45 years, unless the decedent died in this age range or new findings emerge.

**Figure 13:**
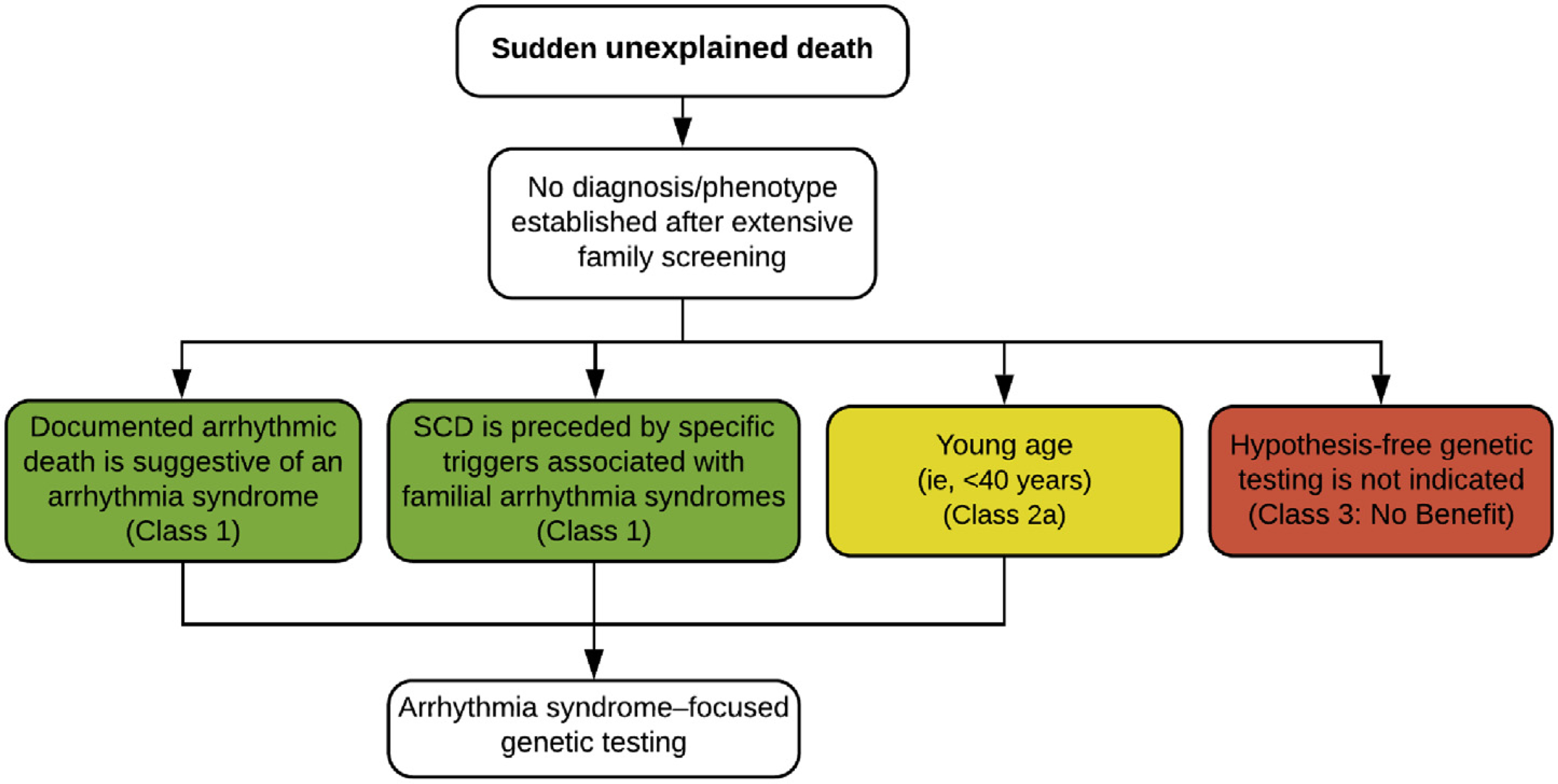
Investigation of sudden death: genetic evaluation where the phenotype is unknown. Colors correspond to the Class of Recommendation in Table 1. SCD = sudden cardiac death.

Three scenarios may trigger arrhythmia syndrome–focused genetic evaluation of SCD even if the phenotype remains unknown: 1) documented arrhythmic death suggestive of an arrhythmia syndrome; 2) specific triggers associated with familial arrhythmia syndromes; 3) young age (<40 years) (Figure 13).

Families should be counseled about the expected benefits and potential outcomes of genetic investigations prior to testing. When genotyping is performed, the identification of a genetic variant as causal remains challenging and requires reassessing the correct classification or potential reclassification periodically. With rapidly advancing genotyping technologies and the availability of large gene panels, the identification of genetic variants of uncertain/unknown significance becomes more frequent. Medical uncertainty in general elicits a variety of responses from patients. It is important to consider patients’ responses to the ambiguous nature of genetic testing. Medical professionals ordering genetic testing should be prepared for the possibility of their patients’ misinterpretation of such results. Pre-test counseling should include a discussion of the possibility of a variant of uncertain significance and what it would mean for the patient’s care and its potential psychosocial impacts. When a variant of uncertain significance is found, post-test counseling should include additional education and a discussion of the variant’s implications and medical management recommendations based on the results. If identified variants are considered likely pathogenic or pathogenic, cascade testing across the family should be offered to identify at-risk individuals with no current clinical features. Cascade testing should not be performed with variants of uncertain significance; however, careful investigation within a multidisciplinary team (including genetic counseling) may allow eventual reclassification of the variant so that it may then be used for cascade testing (see Sections 3 and 4).

**Table:** Recommendations for investigation of sudden death: genetic evaluation where the phenotype is unknown

COR	LOE	Recommendations	References
1	B-NR	1. In an SUD case at a young age where the phenotype remains unknown after expert evaluation, re-evaluation of first-degree relatives to assess for new information that may achieve diagnosis should be performed every 3 to 5 years (shorter intervals should be considered if there is more than one SCD event in the family) until at least age 45 years.	^25,70,80,85,205,206^
1	B-NR	2. In an SUD case where the phenotype is unknown, arrhythmia syndrome–focused genetic testing is recommended if 1) documented arrhythmic death is suggestive of an arrhythmia syndrome, and 2) SCD is preceded by specific triggers associated with familial arrhythmia syndromes.	^25,70,80,84,85,185,205–208^
2a	B-NR	3. In an SUD case occurring in a patient younger than 40 years where the phenotype is unknown, arrhythmia syndrome–focused genetic testing can be useful.	^25,70,80,84,85,205,206,208,209^
3: No Benefit	B-NR	4. In an SUD case where the phenotype is unknown, hypothesis-free genetic testing using exome or genome sequencing is not indicated in routine patient care, as this may lead to misinterpretation of genetic variants (specifically variants of uncertain significance).	^210,211^

#### Synopsis

Collection and storage of blood and/or suitable tissue for postmortem genetic testing is recommended in all victims of SUD. In a large number of cases, the phenotype underlying SCD remains unknown despite comprehensive evaluation of the victim and their family. In SCD cases where the phenotype is determined as unknown after expert evaluation, re-evaluation should be performed periodically to assess for new information that may impact diagnosis. While hypothesis-free genetic testing is not indicated in cases of SCD where the phenotype remains unknown, arrhythmia syndrome–focused genetic evaluation of SCD is advised if 1) an arrhythmic death is documented suggestive of an inherited arrhythmia syndrome, 2) specific triggers associated with familial arrhythmia preceded the SCD, and/or 3) SCD occurred at young age.

#### Recommendation-Specific Supportive Text

1. Genetic diseases may express with reduced penetrance. Hence, a negative clinical screening does not exclude the (silent) presence of a genetic disorder. It is, therefore, reasonable to suggest repeated screening with a time interval between 3 to 5 years until at least age 45 years. Studies demonstrating the yield of clinical screening after the sudden death of a close relative usually report only the result of the first screening.^25,70,80,85,205,206^ Responsibility for the repeated (infrequent) screening lies in the hands of the individual, but the local team should make sure that he/she is appropriately informed.
2. In an SCD case where the phenotype is unknown, arrhythmia syndrome–focused genetic testing of the proband should be considered if 1) documented arrhythmic death (such as torsades de pointes arrhythmias leading to ventricular fibrillation) is suggestive of an arrhythmia syndrome, and/or 2) SCD is preceded by specific triggers (eg, competitive athlete, emotional or physical stress, swimming, drug use, acoustic triggers, seizure) associated with familial arrhythmia syndromes.^10,25,70,80,85,205,206,212^ Collection and storage of blood and/or suitable tissue for postmortem genetic testing is recommended in all victims of SUD irrespective of an identified phenotype at the time of death. Long-term storage of biosamples of SCD victims is recommended in expert centers to allow for genetic testing if indicated at present or in the future.^10,25,70,80,85,205,206,212^
3. In an SCD case where the phenotype is unknown, arrhythmia syndrome–focused genetic testing of the proband can be considered if SCD occurred at young age. Testing for cardiomyopathy genes (such as *LMNA*) has been studied and can increase the diagnostic rate, although it should be recognized that the yield is lower.^10,25,70,80,85,205,206,212^
4. Hypothesis-free genetic testing is not indicated in cases of SCD where the phenotype remains unknown. Genetic testing using any range from large unfocused gene panels to whole-exome or whole-genome sequencing in the absence of a clinical phenotype or diagnosis may be considered in the context of a scientific effort but is not recommended for routine patient care and counseling.^213,214^ The aim of discouraging hypothesis-free testing in clinical settings is to reduce the misinterpretation of genetic variants and their causality, specifically, variants of uncertain significance. A specific problem in this field is the nonuniformity in calling variants across different laboratories.^215^

**Figure 14:**
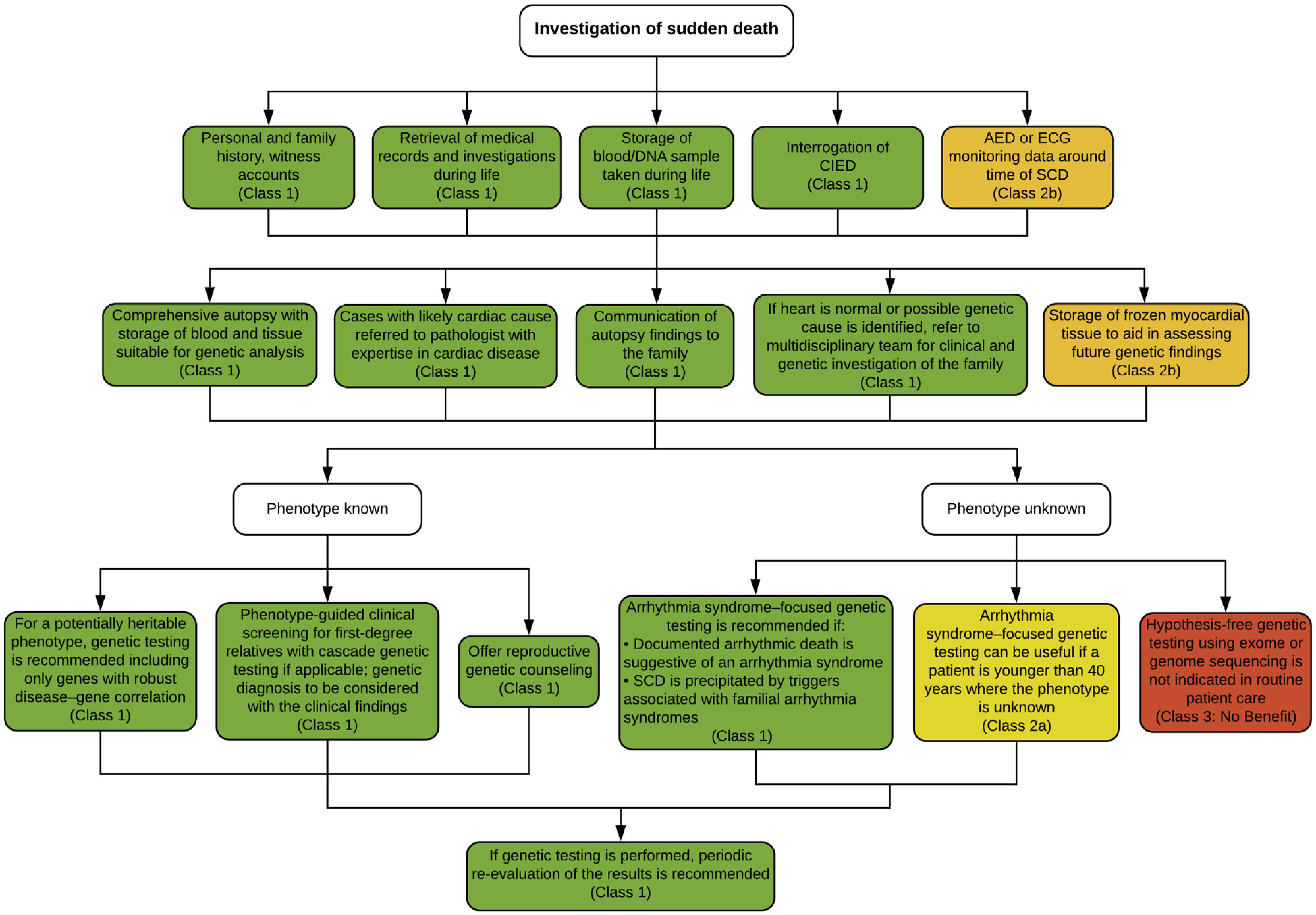
Investigation of sudden death. Colors correspond to the Class of Recommendation in Table 1. AED = automated external defibrillator; CIED = cardiovascular implantable electronic device; ECG = electrocardiogram; SCD = sudden cardiac death.

Figure 14 summarizes the recommendations from Section 6.

## Section 7 Investigation of Sudden Cardiac Arrest Survivors

### 7.1. Investigation of Sudden Cardiac Arrest Survivors: History—Personal and Family

When an individual has been resuscitated after SCA, the clinician must try to define the likely underlying cause, similar to that discussed in Section 6.1 focused on SCD. This should include information on age, sex, past medical history, recent symptoms, activity or emotional status at the time of SCA (eg, sleeping, exercising, or emotion), time of onset (eg, morning or night), and environment (eg, public or private location), exposure to medicinal or recreational drugs (particularly those that block potassium or sodium channels) or alcohol, and a detailed family history of three generations at least.

To focus the history taking, one must consider the differential diagnosis of SCA, which is similar to that discussed in Section 6.1. If the SCA was observed, a description of the event by the observer can add useful information. All records of the primary event, including initial rhythm recordings and details of the resuscitation, should be collected in addition to any prior ECGs and imaging studies. A family history of heart disease, syncope, or sudden death may point to a genetic cause. A study by Waddell-Smith et al.^77^ showed that while inpatient cardiology teams identified a familial condition in only about 8% of cases, nurses trained in taking a family history detected a familial condition in 32% of cases. Thus, although most patients with SCA will be in a nonspecialty environment at first, ideally specially trained members of the cardiology and genetic counselor team with experience in genetic heart disorders should be utilized to elicit relevant details of the family history. Practical educational assistance can be found at https://www.primarycaregenetics.org.

**Table:** Recommendations for investigation of SCA survivors: personal and family history

COR	LOE	Recommendations	References
1	B-NR	1. In the investigation of an SCA survivor, detailed personal and three-generation family history should be taken with the assistance of a multidisciplinary team, including witness accounts.	^77^
1	B-NR	2. All possible details surrounding an SCA event should be sought, including patient’s recollection, witness accounts, and medical records.	^216–220^

#### Synopsis

Observational studies have demonstrated that the cause of SCA can be determined in a substantial proportion of patients. Historical features, especially age, coronary risk factors, symptoms, activity at the time of SCA, exposure to drugs, and family history frequently provide important clues to the diagnosis and point the way to further investigation.

#### Recommendation-Specific Supportive Text

1. In a study of 37 patients with history of SCD, cardiomyopathy, or ventricular tachycardia, a family history obtained by specially trained personnel was far more likely to elicit a history of inherited cardiac disease than one obtained by an inpatient cardiac team.^77^
2. A detailed history is a crucial component of diagnosing the cause of SCA. Studies utilizing a comprehensive, systematic approach including history, physical examination, ECGs (eg, 12-lead ECG, treadmill, 24-hour Holter, signal-averaged ECG, if needed), cardiac imaging (eg, coronary angiography, echocardiogram, CMR, CT), provocative testing, electrophysiological study, cardiac biopsy, and genetic testing have shown that a diagnosis can be established in a substantial proportion of survivors of SCA.^216–219^

### 7.2. Investigation of Sudden Cardiac Arrest Survivors: Examination

The next step in cardiac evaluation of SCA survivors is physical examination. The main purpose is to identify signs of syndromic and nonsyndromic diseases that can be associated with SCA. For example, obesity and/or the presence of xanthomata may indicate an increased likelihood of premature coronary atherosclerosis.^221,222^ Syndromic features that may be relevant to genetic disorders include woolly hair and palmoplantar keratoderma (arrhythmogenic right ventricular cardiomyopathy),^140^ joint contractures (Emery-Dreifuss muscular dystrophy),^223^ muscle weakness and atrophy (lamin A/C and desmin cardiomyopathies, Triadin knockout syndrome),^224^ micrognathia, syndactyly, clinodactyly (Andersen-Tawil and Timothy syndromes),^224^ chest and limb deformities, and tall stature (Marfan syndrome).^225,226^

Fever, hypothermia, dehydration, and signs of drug abuse may be detected on physical examination, although these signs may be confounded by neurological impairment after cardiac arrest. These factors can trigger life-threatening arrhythmia in genetic heart disease. For example, fever has been associated with malignant arrhythmias in 6% of patients with cardiac arrest and Brugada syndrome.^136^

Cardiac murmurs can raise the suspicion of left ventricular outflow tract obstruction, mitral mid-systolic click (valve prolapse) with or without regurgitation, and Ebstein anomaly. Signs of pulmonary edema and hepatosplenomegaly can be detected in patients with severe systolic myocardial dysfunction. Importantly, examination findings may be affected by the SCA event and evolve during a hospital stay, requiring repeated physical examination to determine whether findings are related to the cause of SCA or the effect of SCA.

**Table:** Recommendation for investigation of SCA survivors: examination

COR	LOE	Recommendation	References
1	B-NR	1. Detailed physical examination is recommended after resuscitation from SCA.	^136,140,222,224,225^

#### Synopsis

There are no data describing the usefulness of physical examination in resuscitated SCD. However, it is a first basic step in the diagnostic process that will focus subsequent complementary investigations.

#### Recommendation-Specific Supportive Text

1. Protocols for clinics investigating SCA survivors support the use of physical examination as the first step of clinical overview.^10,216,227^

### 7.3. Investigation of Sudden Cardiac Arrest Survivors: Baseline Investigations

In most emergency settings, a patient resuscitated from cardiac arrest in whom myocardial infarction is suspected will have undergone a coronary reperfusion strategy to treat acute occlusion. It is critical to obtain blood tests (cardiac enzymes, inflammatory markers, glucose, serum electrolytes, and white blood cell count) and pertinent toxicological analysis at presentation. The latter may include testing for drugs of abuse such as ethanol, opiates, and stimulants, as well as levels of prescribed medication that may prolong QT interval/QRS duration or cause respiratory depression.^228^ While assisting in the acute management of a resuscitated patient, these results will also help to differentiate acute myocardial injury as a cause of cardiac arrest (eg, ischemia without clear evidence of coronary occlusion or myocarditis) and pick up other reversible causes such as drug overdose, electrolyte imbalance, or endocrine and metabolic disorders. Retention and storage of suitable blood samples on patient arrival in the emergency department will allow subsequent diagnostic evaluation including DNA extraction and analysis in a patient who dies prior to diagnosis or for later clinical and family review. In some cases, this may be the only opportunity to obtain genetic material for analysis.

In an OHCA, the use of AEDs is ever more widespread and increases survival.^229^ Sensitivity for the diagnosis of cardiac rhythm at the time of arrest is about 99%.^230^ Therefore, routine inspection of data from AED recordings may improve the quality of diagnosis (see Section 6.2). The underlying rhythm of cardiac arrest may provide information on the arrhythmogenic mechanism, assist in diagnosis, and eventually indicate any misdiagnosis of rhythm.^231^ Any ECG tracings from emergency services, as well as recordings from interrogation of CIEDs or wearables can also contribute to diagnosis.^163^

The 12-lead ECG in sinus rhythm or during arrhythmia recurrence is fundamental to the diagnostic investigation and should be repeated daily during recovery.^216^ It may support diagnoses of primary electrical disorders, pre-excitation, and heart muscle diseases. However, abnormalities of cardiac conduction and repolarization may result from myocardial injury during the cardiac arrest and patients undergoing post-arrest hypothermia protocols may have transient ECG changes including QT prolongation and J point elevation that should be interpreted with caution. Information about electrolyte levels, drug prescription, and body temperature should be added to the ECG to prevent misinterpretation of such ECG abnormalities. A high precordial lead ECG is an inexpensive tool to increase detection of Brugada syndrome pattern.^184,232–235^

In addition to standard ECG, a signal-averaged ECG may demonstrate late potentials. Two or more abnormalities in the absence of a prolonged QRS duration (≥110 ms) on the standard ECG is a minor diagnostic criterion for arrhythmogenic cardiomyopathy and suggests ventricular depolarization abnormality.^4^

Continuous heart rhythm monitoring is recommended during hospitalization due to the transient nature of some arrhythmias. Recording the onset (including pause-dependent or tachycardia-associated initiation) and late and short-coupled ventricular ectopics as triggers for torsade de pointes, polymorphic ventricular tachycardia, or ventricular fibrillation will elucidate cardiac arrest mechanism and likely diagnosis.^236^ Evidence of dynamic ST elevation associated with chest pain may also indicate likelihood of coronary vasospasm.^216^

Echocardiography is the screening tool of choice for structural heart disease, although early myocardial dysfunction may be present after cardiac arrest and, if present early, the test should be repeated later during the patient’s convalescence. CMR allows detection of inflammatory diseases, such as myocarditis and sarcoidosis, through recognition of subepicardial edema. Identification of an inflammatory etiology is important, as it may be self-limiting or treatable. If sarcoidosis is suspected, then positron emission tomography–CT scanning may be indicated.^237^ The presence of subendocardial edema would suggest ischemic injury.^238^ Late gadolinium enhancement indicates chronic fibrosis, permitting detection of a cardiomyopathic etiology, and can also contribute to the diagnosis of mitral valve prolapse associated with a risk of SCD.^239,240^ Coronary imaging (at any age) will be important to exclude coronary artery disease not investigated at presentation as an emergency and ensure that an anomalous coronary circulation or coronary dissection is not missed.^241^ This may be by cardiac catheterization or by CT coronary angiography.^242^ Coronary angiography will only be required in select pediatric and young survivors.

Patients in whom the cause of their SCA remains undiagnosed may require periodic re-evaluation of the above investigations, as features may develop later that point to a cause of their SCA (similar to Section 6.5, Recommendation 1).

**Table:** Recommendations for investigation of SCA survivors: baseline investigations

COR	LOE	Recommendations	References
1	B-NR	1. Blood samples for electrolytes, toxicology, and EDTA blood stored for future genetic testing are recommended for all SCA survivors on admission to hospital.	^228^
1	B-NR	2. Retrieval of recordings from CIEDs and wearable monitors is recommended for all SCA survivors.	^243–245^
2b	C-LD	3. Retrieval of recordings from AEDs and ambulance services may be useful for all SCA survivors.	^158,170^
1	B-NR	4. Recording of 12-lead ECGs during sinus rhythm and, if possible, during arrhythmia, is recommended for all SCA survivors.	^85,150,153,216,246^
1	C-LD	5. A high precordial lead ECG is recommended in all undiagnosed SCA survivors to increase detection of a type 1 Brugada ECG pattern.	^189,232–235^
1	C-LD	6. Continuous ECG monitoring is recommended for all SCA survivors during the initial hospital stay.	^85,216,246^
2b	C-LD	7. A signal-averaged ECG may be useful in SCA survivors to aid in the diagnosis of arrhythmogenic cardiomyopathy.	^247^
1	B-NR	8. Echocardiography is recommended for evaluation of cardiac structure and function in all SCA survivors.	^85,216^
1	B-NR	9. CMR with late gadolinium enhancement is recommended for evaluation of acute or chronic myocardial disease in SCA survivors without a clear underlying cause.	^238,248^
2a	B-NR	10. CMR can be useful for evaluation of acute or chronic myocardial disease in SCA survivors, when the etiology is primary electrical or there is evidence for acute cardiac ischemia.	^238,248^
1	B-NR	11. Coronary imaging is recommended in all adult SCA survivors, to exclude coronary artery disease, dissection, or anomalies not considered fully at first presentation, and in select younger cases.	^241^

#### Synopsis

Systematic clinical testing is paramount in SCA survivors. This includes blood testing, toxicology, ECG, signal-averaged ECG, high precordial lead ECG, continuous ECG monitoring, echocardiography, and coronary imaging. If the diagnosis remains elusive and cardiac arrest is deemed unexplained, then CMR is important to identify subtle forms of cardiomyopathy or acquired structural disease.

#### Recommendation-Specific Supportive Text

The usefulness of blood testing and toxicology is by consensus, and a diagnostic role is unquestionable.^2,10,228^ Viral studies may be useful, but no systematic evidence is available as yet.^249^ A potential role for biomarkers specific for one of the arrhythmia syndromes is anticipated.^250,251^
Results of a forensic study indicate the value of postmortem CIED interrogation to define the cause and timing of death more accurately and to detect potential CIED-related safety issues. CIED interrogation in unexplained deaths clarified the manner of death in 60.8% of the cases including cardiac and nonarrhythmic death and device concerns.^163^
Sensitivity for the diagnosis of cardiac rhythm via AED at the time of arrest is about 99%.^230^ However, the AED seldom catches the initial rhythm of cardiac arrest and therefore may not contribute to the etiology of SCA.
Primary electrical disorders and specific cardiomyopathies may be detected by conventional ECG.^2,10,216,252^ ECG findings in the immediate aftermath of a cardiac arrest, other than ST-segment elevation indicative of an acute coronary syndrome, may, however, have poor diagnostic accuracy.^253,254^ These could be caused by abnormal repolarization following electrical cardioversion,^255^ metabolic and electrolyte abnormalities, or even subarachnoid hemorrhage.^256^ Therapeutic hypothermia may lead to misleading ECG changes such as prolongation in PR, QRS and QT intervals, and J point elevation.^257,258^ Interpretation of ECGs obtained immediately after resuscitation/defibrillation should be performed with great caution.
Although there are no data describing directly the value of high precordial lead ECGs in SCD survivors, there is ample evidence of an increased yield of the type 1 Brugada ECG pattern.^189^
Cardiac monitoring during short-term follow-up demonstrates an arrhythmogenic mechanism of SCD in some registries.^216,236,246^
Signal-averaged ECG is part of the Task Force Criteria for arrhythmogenic right ventricular cardiomyopathy.^4^ Signal-averaged ECG has been proposed as useful in other conditions (ie, Brugada syndrome), although systematic evaluation has not been performed.
Echocardiography is a valuable screening tool for detection of arrhythmogenic cardiomyopathy and other structural abnormalities useful in elucidating the cause of SCA.^85^ Patients with functional abnormalities on initial echocardiogram should have this test repeated after recovery, to allow for the effects of the SCA itself and drugs used around the time of the arrest to wear off.
9. and 10. The utility of CMR has been evaluated in a series of studies involving survivors of unexplained arrest and has repeatedly been shown to provide significant incremental diagnostic value. A study of 137 individuals with unexplained aborted cardiac arrest found that CMR provided a diagnosis or identified an arrhythmic substrate in 76% of individuals, including an infarct pattern suggestive of occult myocardial infarction in 44%. Notably, the presence of late gadolinium enhancement, reflective of myocardial fibrosis, was associated with a 6.7 hazard ratio (*p* < 0.001) of recurrent arrhythmic events on multivariate analysis.^248^ The presence of subendocardial edema would suggest ischemic injury even when initial coronary imaging excludes significant obstruction. Coronary vasospasm and dissection might be misdiagnosed, and coronary re-evaluation may then be reconsidered.^238^ The frequency of occult infarcts is not insignificant,^248^ although the risk of SCD in patients with myocardial infarction and nonobstructive coronary arteries is low.^259^ For Recommendation 10, primary electrical disease is not referring to an established diagnosis of long QT syndrome or CPVT, where MRI is unlikely to be of use.
Coronary artery disease is the leading cause of SCD in adults and might be treatable. Furthermore, coronary dissection and anomalies may also be relevant in this age group as well as in younger patients.

### 7.4. Investigation of Sudden Cardiac Arrest Survivors: Provocative Testing

Once a cardiac arrest survivor has undergone initial thorough baseline evaluation, most overt acquired or genetic etiologies will have been diagnosed. However, concealed disorders may be uncovered by provocative maneuvers such as lying to standing ECGs, exercise ECG testing, epinephrine challenge, sodium channel blocker challenge, or ergonovine and acetylcholine testing. Some may even be employed in a resuscitated cardiac arrest survivor who is unlikely to survive due to neurological injury.

Exercise testing may uncover ventricular arrhythmia relevant to the cause of cardiac arrest. For example, evidence of monomorphic ventricular tachycardia arising from the right ventricle is part of Task Force Criteria for diagnosis of arrhythmogenic right ventricular cardiomyopathy (arrhythmogenic cardiomyopathy).^260–262^ Exercise may also uncover concealed epsilon waves or even a type 1 Brugada ECG pattern.^263,264^ The generation of bidirectional ventricular ectopy or tachycardia and/or polymorphic ventricular tachycardia in the absence of ischemia, structural disease, or digoxin toxicity is typical of CPVT^265^ and has been evaluated in the Cardiac Arrest Survivors with Preserved Ejection Fraction Registry (CASPER) protocol.^216^ Abnormal dynamics of repolarization in response to challenges may also inform the likelihood of underlying long QT syndrome. To this end, maximum QT prolongation (QT stretch) and T-wave morphology changes during lying to standing and then return to baseline heart rate (QT stunning) may be useful markers, although they are less specific in children.^266–268^ These discriminate well in genotyped families and may add to diagnostic utility, but evaluation in unexplained cardiac arrest survivors has not been undertaken.^269^ The recovery phase of exercise may also reveal readily measurable QT prolongation and T-wave abnormalities and has been validated in families with long QT syndrome for prediction of genotype^270,271^ and in cardiac arrest survivors^216^ such that a QTc >480 ms at 4 min of recovery forms part of the long QT syndrome risk score.^272^

Epinephrine challenge with ECG monitoring has been advocated as an alternative to exercise testing for the diagnosis of long QT syndrome and CPVT, particularly where the patient is unable to exercise. In long QT syndrome, QT prolongation and secondary T-wave changes have been able to discriminate LQT1 and LQT2 patients from unaffected family members even though they have normal baseline QTc intervals.^273,274^ The test has been assessed in unexplained cardiac arrest survivors and has suggested a low specificity for long QT syndrome.^275^ The finding would be unlikely to provide a secure diagnosis in isolation and was proposed as useful in association with exercise testing and genetic testing. Indeed, in normal subjects, pharmacological sympathetic stimulation does produce significant prolongation of QTc.^276^ Epinephrine testing has also shown some diagnostic utility for CPVT in cardiac arrest survivors by inducing ventricular ectopic activity, bidirectional couplets, and ventricular tachycardia.^275^ However, there is uncertainty as to the ideal cutoff for epinephrine-induced arrhythmia, and the diagnostic sensitivity compared to exercise testing in CPVT families is low.^277^ Isoproterenol challenge for the diagnosis of arrhythmogenic right ventricular cardiomyopathy has also been advocated but has not been tested by other groups or in the unexplained cardiac arrest survivor without overt phenotype.^278^

Sodium channel blocker challenge (ajmaline, procainamide, flecainide, and pilsicainide) has been used extensively for investigating the possibility of Brugada syndrome in cardiac arrest survivors, although these are mainly reported in series of patients with a strong suspicion of Brugada syndrome^279–281^ rather than in unexplained cardiac arrest survivors.^282^ The use of leads V1 and V2 in the second and third intercostal space or high right precordial ECG leads during provocation increases the diagnostic yield.^233,283^ In the CASPER registry, there was a yield from procainamide challenge,^284^ although this may underestimate the true burden, as different sodium channel blockers have different potencies for inducing the type 1 Brugada ECG pattern. For example, ajmaline is associated with an odds ratio of 8 for inducing the type 1 Brugada ECG pattern compared with procainamide^285^ and a 4% yield in a small group of “healthy” controls.^286^ Furthermore, while recent consensus guidelines would give a definite diagnosis to a cardiac arrest survivor with a type 2 or 3 pattern converting to a type 1, the implication of a drug-induced type 1 pattern without a baseline type 2 or 3 is not addressed.^152^ Yet cardiac arrest survivors from CASPER without a type 2 or 3 pattern had positive procainamide challenges.^284^ Other tests such as the full stomach test have been proposed but have not been taken up in general.^287^

Different approaches for provocation of an underlying repolarization abnormality have been employed including drug challenge with quinidine and sotalol and mental stress tests.^288–291^ These may offer utility in the future but have not been tested in the cardiac arrest survivor.

Coronary vasospasm, while a recognized cause of cardiac arrest, may not be picked up clinically at presentation.^292^ Ergonovine or acetylcholine challenge has been proposed as a Class 1 indication by recent guidelines^293^ and was employed selectively in the CASPER experience.^216^ Hyperventilation has also been used as a diagnostic test.^294^ Recent experience from the Paris Sudden Death Expertise Center investigators suggests that pharmacological challenge is useful for diagnosis and could be better employed.^295^ Nonetheless, there are few centers with extensive experience in the use of the test, and there is a possibility of false-positive findings in the cardiac arrest survivor population.

Adenosine challenge has been used to unmask pre-excitation that may otherwise be missed.^296,29,7^ In the absence of other causes, it will indicate the need for electrophysiological study to evaluate the risk of the accessory pathway (rapidity of antegrade conduction) followed by ablation therapy. However, electrophysiological study is not routinely included in the workup of unexplained cardiac arrest.^8^ Indeed, previous consensus guidelines proposed a Class 3 indication when assessing a suspected primary electrical disorder.^8^ It does not add additional diagnostic or prognostic value unless there is evidence to indicate otherwise. For example, pre-excited atrial fibrillation, bundle branch re-entrant ventricular tachycardia, and rapid supraventricular tachycardias that degenerate into ventricular fibrillation have all previously been described as culprits requiring an invasive approach to diagnosis and curative ablation therapy.^298,29,9^ Electroanatomic voltage mapping of the right ventricle is a discretionary tool that may be considered to detect evidence of subclinical arrhythmogenic right ventricular cardiomyopathy.^300^ More recently, extensive endocardial and epicardial mapping of unexplained cardiac arrest cases has been employed to identify cases with either Purkinje triggers and/or subtle depolarization abnormalities that may be suitable for ablation therapy.^301^

**Table:** Recommendations for investigation of SCA survivors: provocative testing

COR	LOE	Recommendations	References
1	B-NR	1. Exercise testing is recommended in all undiagnosed SCA survivors to induce arrhythmias that may support the diagnoses of arrhythmogenic cardiomyopathy and CPVT and to evaluate dynamic depolarization or repolarization features that may support the diagnoses of Brugada syndrome, arrhythmogenic cardiomyopathy, and long QT syndrome.	^216,220,263–265,270,271,302,303^
2a	B-NR	2. Lying to standing ECGs can be useful in SCA survivors for the diagnosis of long QT syndrome, but must be interpreted with caution in children.	^266–270^
2b	B-NR	3. Epinephrine challenge may be considered for the diagnosis of long QT syndrome and CPVT, in those unable to exercise.	^273–275,277,304^
1	B-NR	4. Sodium channel blocker challenge with standard and high precordial ECG leads is recommended for the diagnosis of Brugada syndrome in undiagnosed SCA survivors with suggestive clinical characteristics, including a type 2 or 3 Brugada ECG pattern.	^233,273–275,277,286^
2a	B-NR	5. Sodium channel blocker challenge with standard and high precordial ECG leads can be useful for the diagnosis of Brugada syndrome in SCA survivors where no other disorder has been identified.	^233,279–282^
2b	B-NR	6. Ergonovine, acetylcholine, or hyperventilation testing when performed in experienced centers may be considered for the diagnosis of coronary vasospasm as the cause of SCA in a survivor where no other disorder has been identified.	^216,294,295^
2b	C-LD	7. Adenosine challenge may be useful for the unmasking of ventricular pre-excitation and therefore the diagnosis of rapidly conducted atrial arrhythmia as the likely cause of SCA in a survivor where no other disorder has been identified.	^297^
2a	C-LD	8. An electrophysiological study can be considered if bundle branch re-entrant ventricular tachycardia, pre-excited atrial fibrillation, or supraventricular tachycardia are suspected in an SCA survivor.	^298,299^
2b	C-LD	9. Electroanatomic right ventricular voltage mapping may be considered for detection of subclinical arrhythmogenic cardiomyopathy in an SCA survivor where no other disorder has been identified.	^300^
2b	C-LD	10. An electrophysiological study may be considered in an SCA survivor where no other disorder has been identified to evaluate potential underlying substrate.	^301^

#### Synopsis

Concealed Brugada syndrome, long QT syndrome, CPVT, arrhythmogenic cardiomyopathy, pre-excitation, and coronary vasospasm may be uncovered by provocative maneuvers in the cardiac arrest survivor whose cause of cardiac arrest remains unknown after baseline clinical, ECG, and imaging investigations. Exercise ECG testing and sodium channel blocker challenge appear to offer most potential utility, whereas lying to standing ECGs; epinephrine, isoproterenol, and adenosine challenge; and hyperventilation, ergonovine, and acetylcholine testing may be considered in specific patients. Electrophysiological study and electroanatomic mapping may be useful to provide patient-specific insights into the mechanism of cardiac arrest and offer therapeutic options but should be avoided in the routine investigation of channelopathy. However, data in general are limited to case reports or case series, some with validation cohorts, and there are no randomized studies.

#### Recommendation-Specific Supportive Text

1. Exercise testing is a versatile, straightforward, and readily available test that may yield diagnoses due to the arrhythmic challenge or effects on depolarization and repolarization. As such, it should be a standard part of the investigative armamentarium.^216,220,263–265,270–272,302,303^
2. Lying to standing ECGs may offer some insights into the likelihood of long QT syndrome, but in isolation a positive result may not fulfill diagnostic criteria and therefore may only complement the evaluation.^266–270^
3. Similarly, epinephrine challenge has low specificity for long QT syndrome and may be insensitive for CPVT. In isolation, a positive result may not fulfill diagnostic criteria and therefore may only complement the evaluation.^273–275,277,304^ There are risks to this test, both in inducing ventricular arrhythmia and in false diagnosis.
4. Sodium channel blocker testing has a clear role to play in evaluating the cardiac arrest survivor, although the implication of a positive result in patients with or without a high prior likelihood of Brugada syndrome is unclear, eg, male vs. female, cardiac arrest during sleep vs. exercise, type 2 or 3 ECG pattern vs. no Brugada pattern at baseline. This reflects the lack of a gold standard for the diagnosis of Brugada syndrome, and therefore the utility of sodium channel blocker challenge in patients without a clear prior likelihood of Brugada syndrome is less certain and merits a lower utility in the evaluation cascade.^233,273–275,277,286^ Furthermore, sodium channel blocking agents differ in their sensitivity and specificity for inducing ECG changes and availability varies worldwide (see Section 7.4 text).^285^
5. See 4.^233,279–282^
6. The utility of ergonovine or acetylcholine challenge or hyperventilation testing in all cardiac arrest survivors is unclear, as there are no studies of systematic testing in all cardiac arrest survivors. In particular, the specificity of the test is unknown, especially in the cardiac arrest survivor population. It can, however, lead to diagnoses when employed in a protocol and therefore should be considered as part of the armamentarium until more evidence is available.^216,293–295^
7. Adenosine challenge has not been tested systematically in cardiac arrest survivors, but there are limited data suggesting that it will uncover concealed pre-excitation that may cause cardiac arrest in the setting of rapidly conducted pre-excited atrial tachyarrhythmias.^296,297^
8. There are limited case series describing the use of electrophysiological study in diagnosis and treatment of pre-excited atrial arrhythmias and bundle branch re-entrant ventricular tachycardia.^298,299^
9. Studies of endocardial mapping report a higher sensitivity for detection of arrhythmogenic cardiomyopathy, but this has not been explored in SCA survivors.^300^ Sensitivity and specificity are unknown, particularly in patients where no other tests are abnormal.
10. Extensive endocardial and epicardial mapping of unexplained cardiac arrest cases has been employed to identify cases with either Purkinje triggers and/or subtle depolarization abnormalities that may be suitable for ablation therapy.^301^

### 7.5. Investigation of Sudden Cardiac Arrest Survivors: Genetic Evaluation

**Figure 15:**
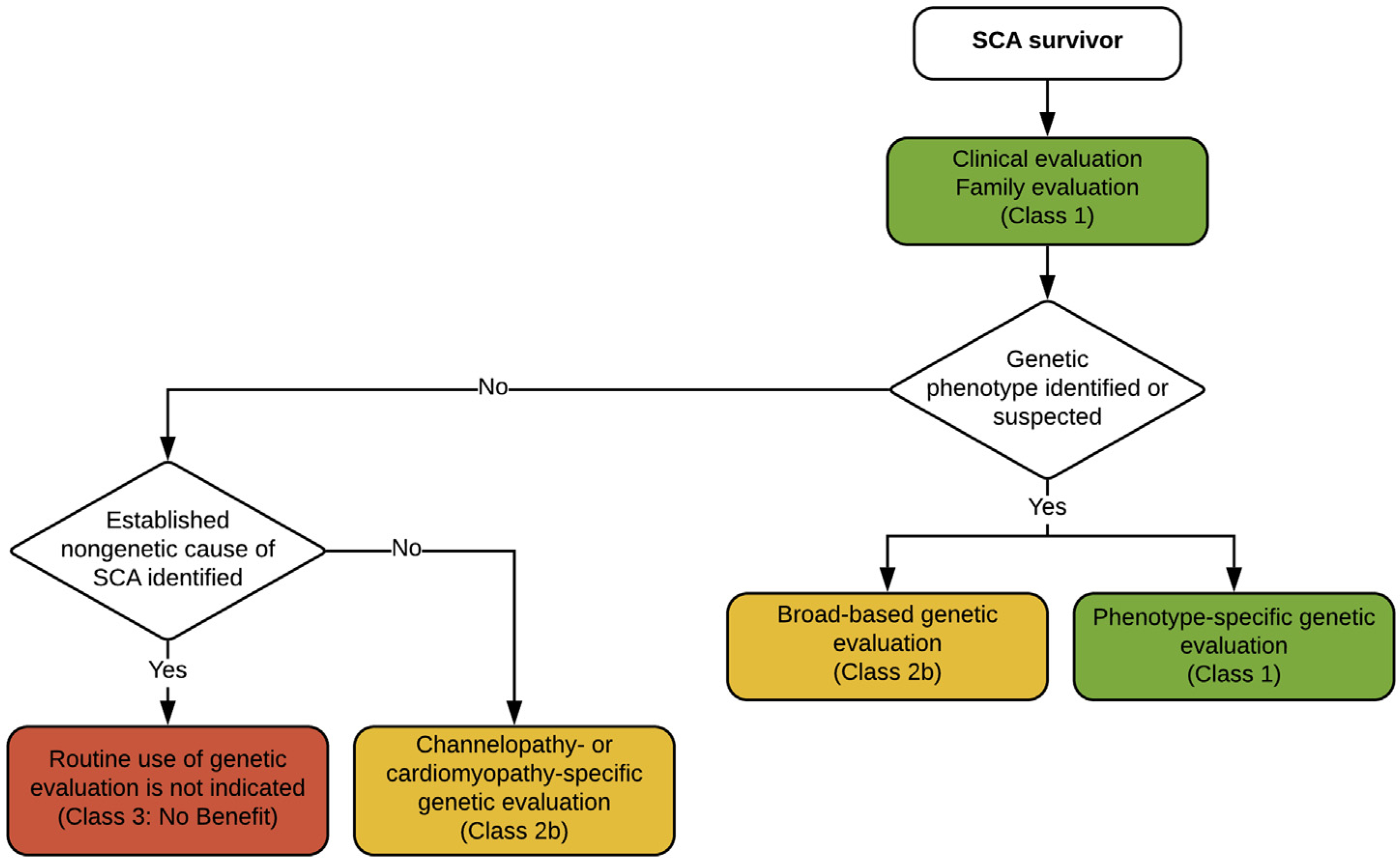
Investigation of sudden cardiac arrest (SCA) survivors: genetic evaluation. Colors correspond to the Class of Recommendation in Table 1.

Although most SCA survivors will have an indication for an implantable defibrillator for secondary prevention of a cardiac arrest,^6^ genetic evaluation may influence final diagnosis, treatment recommendations, and family screening (Figure 15). In some cases, genetic evaluation may enable therapy specific to the disease mechanism. Recent technological advances in genetic evaluations, establishment of reference databases of genetic variants, systematic annotation of causal genes,^305^ and standardization of variant interpretation^306^ have enabled efficient and comprehensive genetic assessment. However, the pace of genetic discovery and variant interpretation is evolving rapidly, creating a complex landscape surrounding genetic evaluation of SCA survivors.

The decision to pursue genetic evaluation is an individualized one in which the patient, with proper (clinical and genetic) counseling, must weigh the benefits, limitations, and personal and familial implications. The genetic basis of SCA and most individual conditions that predispose to SCA remain incompletely understood. As such, caution is advised when interpreting negative genetic test results or when examining genes with low likelihood of causing SCA. Prior clinical practice guidelines have specifically addressed the diagnostic considerations regarding specific genetic cardiovascular conditions.^4,10,307^ The ClinGen represents a systematic effort to enumerate causal genes and variation related to human disease, including SCA-related conditions.^305^ Previous reports have been developed for Brugada syndrome,^195^ long QT syndrome,^196^ and hypertrophic cardiomyopathy.^202^

The yield of genetic testing varies substantially by condition.^188^ Variant interpretation may differ by laboratory^215^ despite recent efforts to standardize variant interpretation.^306^ Given the rapid changes in available technology to evaluate the genome, complexities in variant interpretation, and nuances in the ethical and legal framework surrounding genetic testing in some settings,^66,115,215,308–310^ it is recommended that SCA survivors undergoing genetic assessment have evaluations performed at centers with multidisciplinary experience in counseling, variant interpretation, and management of genetic heart disease (see Section 3). For SCA survivors who have undergone genetic testing, an offer of periodic re-evaluation of the genetic test results is advocated (similar to Section 6.4, Recommendation 4).

**Table:** Recommendations for genetic evaluation of SCA survivors

COR	LOE	Recommendations	References
1	B-NR	1. Genetic evaluation of SCA survivors is recommended for those with a diagnosed or suspected genetic cardiac disease phenotype when the results are likely to influence diagnosis, management, or family screening.	^84,311–322^
1	B-NR	2. When genetic evaluation is performed in an SCA survivor with a suspected or diagnosed genetic cardiac disease phenotype, it is recommended that evaluations include only genes where there is robust gene–disease association.	^84,308^
2b	B-NR	3. When genetic evaluation is performed in an SCA survivor with a suspected or diagnosed genetic cardiac disease phenotype, assessment of genes or genomic regions that are not known to be causally related may be considered in select circumstances.	^84^
2b	B-NR	4. Genetic evaluation of SCA survivors without a distinct genetic cardiac disease phenotype may be considered in select circumstances.	^82,84,209,246,323–330^
3: No Benefit	C-EO	5. Genetic testing in SCA survivors with a well-established nongenetic cause of SCA is not recommended.	

#### Synopsis

SCA can be caused by diverse etiologies, some of which may be predominantly or partially influenced by genetic predisposition. Whereas a thorough clinical evaluation leads to a diagnosis of the cause of SCA for most individuals, the cause of SCA may remain uncertain in others.^216^ In some cases, genetic evaluation of SCA survivors can confirm a molecular etiology that predisposed to the SCA event, support the diagnosis of a specific phenotype, influence management, and facilitate screening in family members at risk via cascade genetic testing.

#### Recommendation-Specific Supportive Text

**Figure 16:**
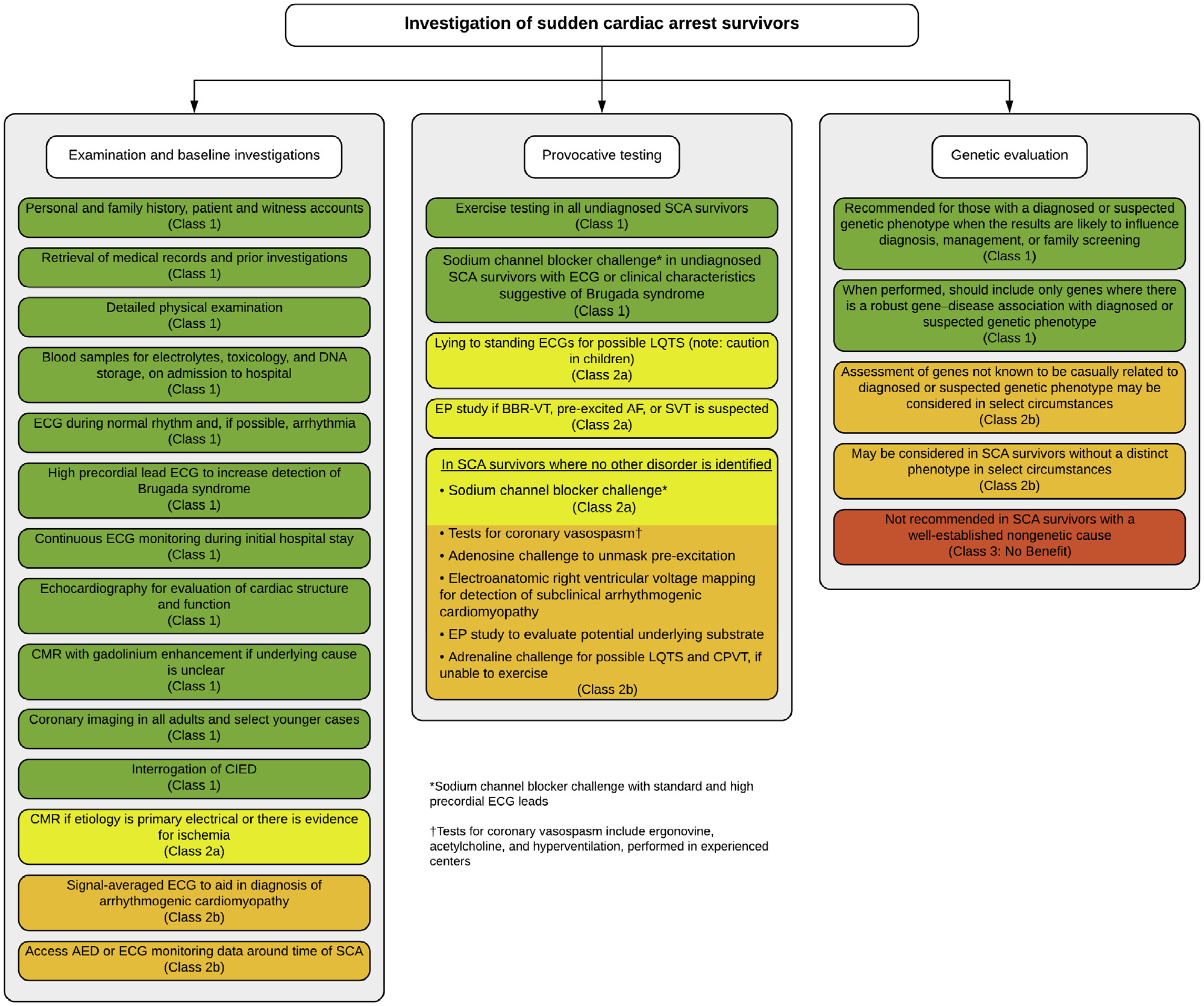
Investigation of sudden cardiac arrest survivors. Colors correspond to the Class of Recommendation in Table 1. AED = automated external defibrillator; AF = atrial fibrillation; BBR-VT = bundle branch re-entry ventricular tachycardia; CIED = cardiovascular implantable electronic device; CMR = cardiac magnetic resonance imaging; CPVT = catecholaminergic polymorphic ventricular tachycardia; ECG = electrocardiogram; EP = electrophysiological; LQTS = long QT syndrome; SCA = sudden cardiac arrest; SVT = supraventricular tachycardia.

Genetic testing, after appropriate genetic counseling and informed consent, may facilitate identification of a molecular cause of SCA by identifying pathogenic variants in genes associated with specific phenotypes and fulfilling formal disease-based diagnostic criteria.^4,10^ Examples of scenarios in which discovery of a genetic cause of SCA may influence management recommendations include administration of beta blockade for patients with long QT syndrome,^311^ sodium channel inhibition in long QT syndrome type 3,^312–314^ flecainide administration for patients with CPVT,^315,331^ or exercise restriction recommendations in patients with arrhythmogenic cardiomyopathy.^316,317^ Genetic evaluation may also influence family screening by facilitating cascade genetic testing and clinical surveillance in relatives at greatest risk for disease.
2. and 3. Genetic tests have variable yield and may result in discovery of variants of uncertain clinical significance, which can be frequent and challenging to interpret. Using genetic tests that comprise well-established genes related to a suspected or diagnosed genetic phenotype is most likely to result in discovery of disease-causing variants in an individual or family, while minimizing the probability of discovering a variant of uncertain clinical significance. In contrast, genetic tests with more comprehensive genomic coverage may lead to moderately increased diagnostic yield but at the expense of increased rates of discovery of variants of uncertain clinical significance.^84,308^ Nevertheless, given a rapidly evolving understanding of the molecular causes of specific phenotypes and increased yield of broader genetic assessment,^82,84,246,323,324^ tests that include broader coverage may be considered in select circumstances, such as when a heritable phenotype is being mapped within a family or assessment for de novo variation is sought through sequencing of multiple family members. The latter examples would only be pertinent when it becomes apparent that a familial trait is likely or there has been exome sequencing in a trio of confirmed parents and the index case.
Genetic evaluation appears to be highest for individuals with a phenotype consistent with a genetic cause^84,323,324^ and is lower among SCA survivors without a clearly identifiable genetic phenotype.^82,84,246,323–326^ Nevertheless, individuals with an idiopathic cause of SCA may eventually develop a diagnosis of a genetic etiology during long-term follow-up.^327^ Genetic testing can identify variants during the concealed phase of a genetic disease such as arrhythmogenic cardiomyopathy,^328^ or in individuals with conditions otherwise regarded as nongenetic such as in drug-induced long QT syndrome.^329^ As such, a low but non-negligible yield for genetic testing appears to be present among individuals with idiopathic SCA.
In individuals with a well-established nongenetic cause of SCA, the routine use of genetic evaluation is not recommended owing to the potential for discovery of variants of uncertain significance and misdiagnosis.
Figure 16 summarizes the recommendations from Section 7.

## Section 8 Investigation of the Family

### 8.1. Background

The sudden death of a young, apparently healthy individual raises many questions for family members. Apart from mourning and the question “could we have done something to avoid this,” a very relevant question is whether family members could be affected as well. Roles for health care providers include providing psychological support for the family, identifying a cause for the sudden death, and understanding the implications for family members (see Sections 4 and 5). For this chapter, it is especially relevant that families need support to organize clinical and genetic testing for family members and sometimes postmortem genetic testing of the deceased.^2,101,332^

Currently, many cases and even familial forms of unexplained cardiac disease remain insufficiently investigated.^333^ When clinical symptoms indicate that a cardiomyopathy or arrhythmia may have contributed to the death, further steps are needed to specify the diagnosis. If blood or tissue of the deceased is available, DNA testing can be done for a range of arrhythmia syndromes and cardiomyopathies, nowadays often using gene panels. Without a specific diagnosis in the deceased, clinical investigation of the first-degree relatives (parents, siblings, and children) can identify a person with similar symptoms or signs, although sometimes mild. This relative of the deceased can be the proband for DNA testing and thus provide the key to a diagnosis for the family.

Efforts are needed to increase the proportion of postmortem examinations (either forensic or medical autopsy) to clarify whether or not an underlying cause can be suspected or proven. The postmortem result should be communicated to the family as per local protocols. Without a postmortem diagnosis, efforts are needed to evaluate eventual clinical symptoms of the parents and other first-degree family members.^80,81,143,144,334^ This is outlined in Section 6. If DNA of the deceased person can be used for testing, or a relative who has similar symptoms can be tested, a monogenic form of cardiomyopathy or arrhythmia may be recognized that may also be present in family members. Typically, first-degree relatives are at 50% risk of carrying the same pathogenic mutation, since many of these conditions follow an autosomal dominant pattern of inheritance.

**Figure 17:**
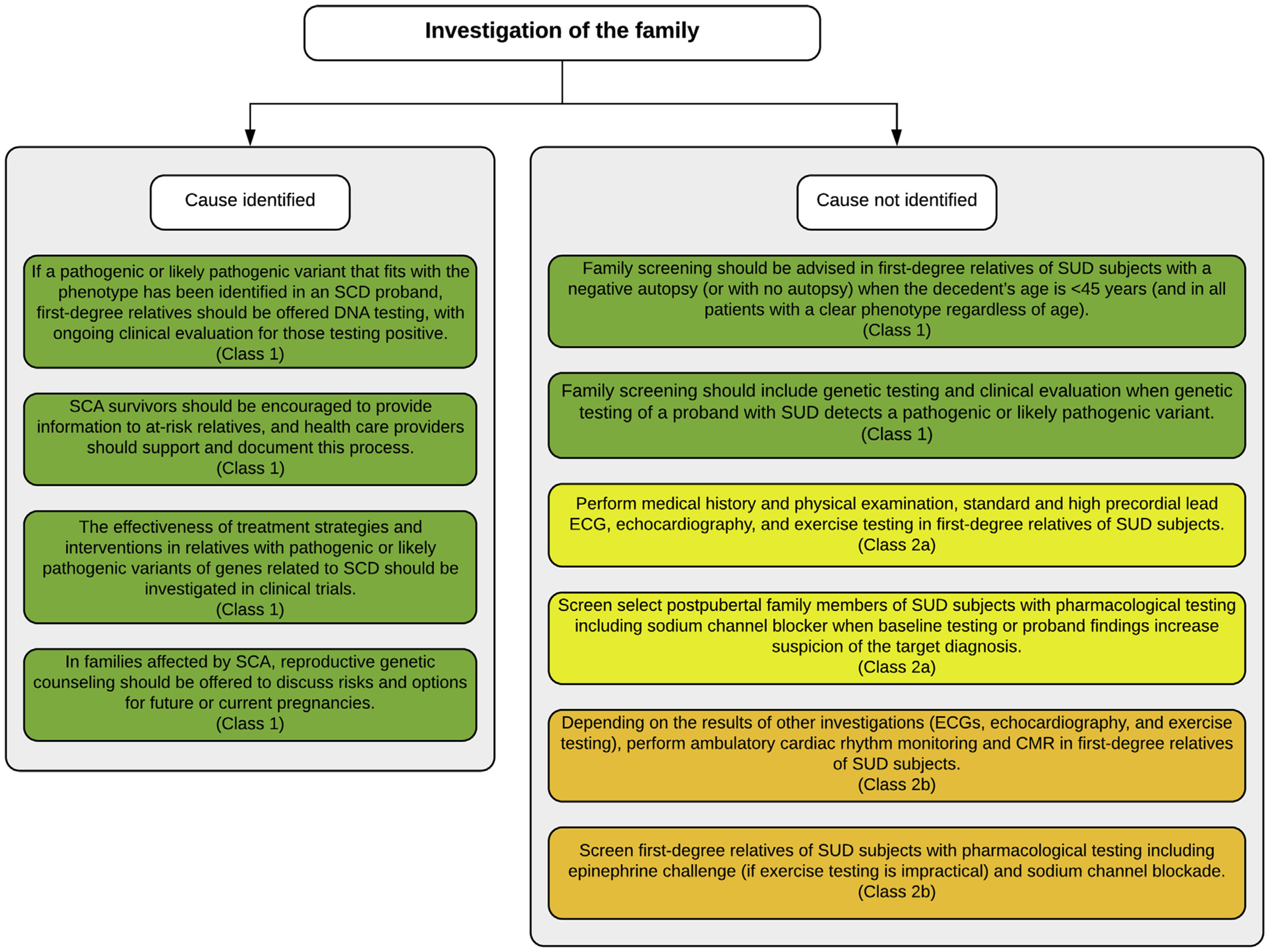
Investigation of the family affected by sudden cardiac arrest and/or sudden unexplained death when cause is identified or not identified. Colors correspond to the Class of Recommendation in Table 1. CMR = cardiac magnetic resonance imaging; ECG = electrocardiogram; SCA = sudden cardiac arrest; SCD = sudden cardiac death; SUD = sudden unexplained death.

Depending on whether or not autopsy has been performed, whether DNA is available, whether a relative is already diagnosed with a cardiogenetic condition, and whether symptoms were noticed during life, there are different situations possible: 1) sudden death, with clinical observations or DNA testing suggesting a specific diagnosis, and 2) sudden death, with no cause identified. The first situation will be discussed in Section 8.2, the second in Section 8.3 (Figure 17).

### 8.2. Investigation of the Family: Cause Identified—Cascade Testing, Clinical and Genetic Investigations

In cascade testing, after the postmortem diagnosis in an index patient by a DNA test, an invitation can be sent to parents, brothers and sisters, and children.^335^ If any of the relatives is also diagnosed with the condition, a next circle of first-degree relatives is invited. If one of the first-degree relatives is not available (either deceased or not wanting to participate), second-degree relatives are invited, for instance, children of a deceased sibling. This systematic approach is very effective in autosomal dominant conditions, since first-degree relatives are at 50% a priori risk to carry the same pathogenic mutation and second-degree relatives at 25% risk. For minors, the age at which treatment starts determines the age before which DNA testing is advised. This may differ between countries and conditions. DNA testing of minors is not advised if the result would have no consequences in childhood.

Presymptomatic DNA testing makes it possible to organize preventive measures, such as regular cardiological follow-up, use of medication (eg, beta blockers), lifestyle advice (eg, avoid intensive sports), implantable cardioverter-defibrillators, or reproductive planning.^80,193,336–342^ Presymptomatic DNA testing also puts an end to uncertainty and fear for those family members who test negative.^339^ For them, follow-up investigations are no longer indicated.

In studies describing cascade screening, often the participation of relatives is limited.^343–348^ This implies that many persons carrying pathogenic variants remain undiagnosed and are at continued risk of sudden death. Increasingly, geneticists and other stakeholders plea for an active approach to cascade testing for conditions where interventions are available.^349–351^ Stakeholders agree on the importance of early diagnosis and informing the family.^2,340,352^ Barriers to cascade screening include out-of-pocket expenses for the patient, limited resources for informing relatives, and privacy regulations.^339,351,353,354^ To benefit from predictive, personalized, and preventive medicine, the roles and responsibilities of stakeholders in genetic testing as a preventive strategy need to be carefully aligned.

If clinical signs and symptoms suggest an inherited condition but no DNA test has been performed or no pathogenic variant has been identified, then history, examination, and clinical investigations of first-degree relatives are required to identify those at risk for SCD.

**Table:** Recommendations for investigation of the family: cause identified—cascade testing, clinical and genetic investigations

COR	LOE	Recommendations	References
1	C-LD	1. If a pathogenic or likely pathogenic variant that fits with the phenotype has been identified in an SCD proband, first-degree relatives should be offered DNA testing, with ongoing clinical evaluation for those testing positive.	^70,84,346,355,356^
1	C-LD	2. SCA survivors should be encouraged to provide information to at-risk relatives, and health care providers should support and document this process.	^350,357^
1	C-LD	3. The effectiveness of treatment strategies and interventions in relatives with pathogenic or likely pathogenic variants of genes related to SCD should be investigated in clinical trials.	^333,358^
1	B-NR	4. In families affected by SCA, reproductive genetic counseling should be offered to discuss risks and options for future or current pregnancies.	^336–338^

#### Synopsis

A postmortem diagnosis in a victim of SCD implies the possibility of avoiding sudden death in relatives. This requires an active approach to inform parents, siblings, and children of the index case and offer clinical evaluation and potential genetic testing. A multidisciplinary service should support all elements of recognizing an inherited cardiac disorder in a victim (pathologist), identifying pathogenic mutations (geneticist), clinical evaluation and surveillance of carriers of the mutation (cardiologist), and supporting the patients and relatives (psychologist).

#### Recommendation-Specific Supportive Text

1. Since undiagnosed cardiogenetic conditions can be life-threatening and since interventions are available to reduce the risk of sudden death, first-degree relatives who are at 50% risk of carrying the pathogenic variant need to be informed about the possibilities of clinical investigations and genetic testing.^70,84,346,355,356^ Second-degree relatives whose intervening first-degree relative refuses genetic testing (or is not available) should be offered testing due to a 25% risk.
2. Patients often can inform their relatives at risk, but medical professionals increasingly feel ethically responsible to support this process.^349,350,357^ Supplying a letter for the patient to share with relatives is advised. Occasionally, directly contacting relatives may be possible in some jurisdictions. Studies have shown that there is substantial room for improvement of the uptake of family screening. Barriers include reluctance to consent to postmortem investigations, lack of information, and lack of funding for the services. While the services needed should involve many disciplines (pathologist, cardiologist, geneticist), the systematic approaching of family members fits the specialty of clinical genetics well. To achieve a high uptake, a systematic approach is needed.
3. The identification of at-risk individuals leads to the question of whether all require active treatment. Prospective clinical studies are needed to answer this question. To balance pros and cons for survival and for quality of life on treatment, long-term follow-up of persons following the suggested surveillance and interventions is needed. Precision prevention advice should build on this evidence.
4. In families with genetically affected individuals (with or without clinical findings), detailed prenatal counseling and guidance regarding inheritance patterns, variant penetrance, and risk should be offered, and other options including preimplantation genetic diagnosis should be explored.^336–338^

### 8.3. Investigation of the Family: Cause Not Identified—Clinical and Genetic Investigations

Sudden death in the young is always a tragedy for those lost and the remaining family. When the cause of sudden death is not identified, either because there was no postmortem examination or because the autopsy was negative, significant anxiety in the family focuses on two major questions: why did the subject die suddenly and what risks apply to the remaining family members? An autopsy is typically requested, and in some jurisdictions mandated, but may not be completed because of cultural, family, or logistical limitations. When a postmortem examination establishes a cause of death, diagnosis transitions from SUD to death attributed to autopsy-related findings (see Section 8.2). When no underlying anatomic or toxicologic cause of death is identified with forensic autopsy, the description of the death goes from SUD to sudden arrhythmic death (syndrome)/SAD(S), since death is attributed to a presumed arrhythmia, or autopsy-negative SCD. In the case of nonspecific findings, follow-up of families should be similar to that with a negative autopsy.^184,359^

When sudden death is classified as SAD or autopsy-negative SCD (ie, SUD), the differential diagnosis includes a breadth of inherited conditions that are predominantly ion channelopathies, with latent cardiomyopathy a consideration based on subtle autopsy findings. Causes include long QT syndrome, CPVT, short QT syndrome, Brugada syndrome, and arrhythmogenic cardiomyopathy. Careful review by a trained cardiac pathologist is recommended to ensure that assigned causes or absent causes are accurate (see Section 6.3).^2^

Recent studies have shown that a genetic evaluation of the deceased subject’s DNA associated with a clinical evaluation of first-degree relatives of the deceased subject retrospectively identified the cause of death in 20–40% of cases.^70,80,81,143,144,189,3,3,4^ In this situation, the identification of the cause of sudden death provides an explanation to the family and facilitates further cascade screening. In a second step, it will enable prevention measures in the family to limit the risk of a second death.

**Table:** Recommendations for investigation of the family: cause not identified—clinical and genetic investigations

COR	LOE	Recommendations	References
1	B-NR	1. Family screening should be advised in first-degree relatives of SUD subjects with a negative autopsy (or with no autopsy) when the decedent’s age is <45 years (and in all patients with a clear phenotype regardless of age).	^80,81,143,144,334^
1	B-NR	2. Family screening should include genetic testing and clinical evaluation when genetic testing of a proband with SUD detects a pathogenic or likely pathogenic variant.	^70,80,81,84,143,144,189,334^
2a	B-NR	3. It is reasonable to take a medical history and perform physical examination, standard and high precordial lead ECG, echocardiography, and exercise testing in first-degree relatives of SUD subjects.	^80,81,143,144,334^
2b	C-LD	4. Depending on the results of other investigations (ECGs, echocardiography, and exercise testing), it may be reasonable to perform ambulatory cardiac rhythm monitoring and CMR in first-degree relatives of SUD subjects.	^70,81,143,189,360^
2a	B-NR	5. It is reasonable to screen select postpubertal family members of SUD subjects with pharmacological testing including sodium channel blocker when baseline testing or proband findings increase suspicion of the target diagnosis.	^143,189,205,275,288,334^
2b	B-NR	6. It may be reasonable to screen first-degree relatives of SUD subjects with pharmacological testing including epinephrine challenge (if exercise testing is impractical) and sodium channel blockade.	^189,205,275,288,334^

#### Synopsis

Screening of first-degree relatives of the SCD victim is informed by findings from the forensic investigation. Though the yield of genetic testing is relatively low, results should be applied to all first-degree relatives in conjunction with clinical assessment. In the absence of genetic results, screening tests should include a medical history, standard and high precordial lead ECG, 24-hour ambulatory monitoring, echocardiography, and exercise test, with select use of pharmacological provocation and advanced imaging.

#### Recommendation-Specific Supportive Text

Although autopsy is recommended, family screening after sudden death in young patients is effective even when an autopsy is not conducted. Broad screening of first-degree relatives with systematic testing is warranted.^80,81,143,144,334^ Combining molecular autopsy with clinical evaluation in surviving families increases diagnostic yield.^70^ The value of surveillance testing after negative evaluation is uncertain, though commonly undertaken until age 45 years (range: 40–50 years), with decreasing frequency with age.^85^ The context of SCD, the family history, and existing findings should inform the potential merits of ongoing surveillance, including frequency and duration. The age at which and from which onward surveillance is warranted depends on the (suspected) underlying condition.
When decedent genetic testing detects a pathogenic or likely pathogenic variant, the result enables identification of all family members at risk of SCD.^80,81,143,144,334^ It is important to combine genetic and clinical evaluation, especially when the pathogenicity of the detected variant is uncertain, to evaluate the correlation between the genetic finding and clinical diagnosis for each family.^83^ If DNA is not available from the decedent and no clinical phenotype is present in the family, genetic testing of family members should be strongly discouraged.
Several studies have demonstrated that family screening should include at least a medical history, standard and high precordial lead ECG (to improve the detection of the Brugada syndrome), echocardiography, exercise test,^80,81,143,144,334^ and Holter monitoring on a case-by-case basis.
CMR and 24-hour ambulatory monitoring of family members can inform diagnosis.^4,5^
5. and 6. A careful assessment of the circumstances of sudden death may point to a specific diagnosis. Sudden death in a young or middle-aged male occurring during a febrile illness or sleep suggests the diagnosis of Brugada syndrome, whereas sudden death occurring in a subject during physical activity suggests the diagnosis of long QT syndrome or CPVT. As Brugada syndrome may be masked or intermittent in some patients, sodium channel blocker challenge may unmask the type 1 pattern and increase the effectiveness of family screening. It should be recognized that there is a potential high rate of false positives, as data on the specificity and sensitivity of the test are not available, and a positive ECG may be induced in 4–5% of normal subjects.^286,361^ In the pediatric age group, a negative test may convert to a positive test after puberty.^362^ Long QT syndrome may also be unmasked by standing, exercise test, epinephrine test, or mental stress test.^266,269,270,275,288^ In first-degree relatives of young SUD victims with no manifest abnormalities during the initial examination, the risk of developing manifest inherited cardiac disease or cardiac events during follow-up is low.^205^

## Section 9 Future Directions

Many of the recommendations in this document seem intuitive, obvious, and straightforward; however, much of what is being recommended within this document is seldom routinely performed even in well-resourced countries. Many decedents of SUD never receive an autopsy, and the evaluation of first-degree relatives of an SUD victim or an SCA survivor ranges widely from no evaluation to ordering a multitude of tests that are then repeated regularly and indefinitely. The extensive variability in practice indicates that developing common sense processes and multidisciplinary teams remains a considerable challenge in many areas and guidance is required. Developing these processes and teams involves leaders challenging many medico-political barriers that obstruct and delay best medical practice. We hope that this document will empower those who wish to achieve such changes for the better, including efforts for continuous improvement of practice. So, assuming the recommendations in this document are embraced and implemented, what are the next steps and future directions in this field?

Firstly, unlike cancer statistics and even SCD in the elderly due to coronary artery disease, the precise prevalence, epidemiology, and etiologies of either SUD or SCA in the young remain obscure in most countries. Only if these conditions become a notifiable event will the true scale and scope ever be captured.

Secondly, communities, states/provinces, and countries must advocate for and expect that a true comprehensive autopsy, including postmortem genetic testing (ie, molecular autopsy), occurs whenever an SUD occurs in a young person. The current dismal rate of autopsy must be reversed. Only when this becomes the standard of care will the true epidemiology/etiology of SCD be determined.

Thirdly, the basic occurrence and subsequent extent of an evaluation of the living, whether she/he is an SCA survivor or the first-degree relative of an SUD victim or an SCA survivor, must become standard of care. After initial cardiological evaluation (examination, ECGs, stress test, and echocardiogram) has been completed, the true contribution of SCA-predisposing genetic heart disease will be exposed. Furthermore, the composition and contribution of advanced investigations ranging from sodium channel drug provocation studies, to MRI, to genetic testing requires further study, as does the recommended interval for a repeat cardiological evaluation of the first-degree relatives when their first evaluation is either normal or inconclusive.

Finally, when such investigations are commenced, future studies to minimize the collateral damage from uncertain clinical findings and genetic “variants of uncertain significance” will be needed. While it is recognized that the correct necropsy diagnosis of the SUD decedent and the correct diagnosis of the SCA survivor may give patients and their families some answers and resolution to the event, the premature and erroneous diagnosis due to excessive confidence in or overinterpretation of clinical or genetic findings of uncertain significance can cause remarkable harm. If we are to “first do no harm,” the model of multidisciplinary teams with the expertise to correctly evaluate all the investigations and potential (mis)diagnoses should be made accessible to all.

## Supplementary Material

supplementary Appendix 3

## Figures and Tables

**Table 1:** ACC/AHA recommendation system: Applying Class of Recommendation and Level of Evidence to clinical strategies, interventions, treatments, and diagnostic testing in patient care*

**CLASS (STRENGTH) OF RECOMMENDATION**	
**CLASS 1 (STRONG)**	**Benefit >>> Risk**
**Suggested phrases for writing recommendations:**	
• Is recommended	
• Is indicated/useful/effective/beneficial	
• Should be performed/administered/other	
• Comparative-Effectiveness Phrases†:	
– Treatment/strategy A is recommended/indicated in preference to treatment B	
– Treatment A should be chosen over treatment B	
**CLASS 2a (MODERATE)**	**Benefit >> Risk**
**Suggested phrases for writing recommendations:**	
• Is reasonable	
• Can be useful/effective/beneficial	
• Comparative-Effectiveness Phrases†:	
– Treatment/strategy A is probably recommended/indicated in preference to treatment B	
– It is reasonable to choose treatment A over treatment B	
**CLASS 2b (WEAK)**	**Benefit ≥ Risk**
**Suggested phrases for writing recommendations:**	
• May/might be reasonable	
• May/might be considered	
• Usefulness/effectiveness is unknown/undear/uncertain or not well-established	
**CLASS 3: No Benefit (MODERATE) (Generally, LOE A or B use only)**	**Benefit = Risk**
**Suggested phrases for writing recommendations:**	
• Is not recommended	
• Is not indicated/useful/effective/beneficial	
• Should not be performed/administered/other	
**Class 3: Harm (STRONG)**	**Risk > Benefit**
**Suggested phrases for writing recommendations:**	
• Potentially harmful	
• Causes harm	
• Associated with excess morbidity/mortality	
• Should not be performed/administered/other	
**LEVEL (QUALITY) OF EVIDENCE**‡	
**LEVEL A**	
• High-quality evidence‡ from more than 1 RCT	
• Meta-analyses of high-quality RCTs	
• One or more RCTs corroborated by high-quality registry studies	
**LEVEL B-R**	**(Randomized)**
• Moderate-quality evidence‡ from 1 or more RCTs	
• Meta-analyses of moderate-quality RCTs	
**LEVEL B-NR**	**(Nonrandomized)**
• Moderate-quality evidence‡ from 1 or more well-designed, well-executed nonrandomized studies, observational studies, or registry studies	
• Meta-analyses of such studies	
**LEVEL C-LD**	**(Limited Data)**
• Randomized or nonrandomized observational or registry studies with limitations of design or execution	
• Meta-analyses of such studies	
• Physiological or mechanistic studies in human subjects	
**LEVEL C-EO**	**(Expert Opinion)**
• Consensus of expert opinion based on clinical experience	

COR and LOE are determined independently (any COR may be paired with any LOE).

A recommendation with LOE C does not imply that the recommendation is weak. Many important clinical questions addressed in guidelines do not lend themselves to clinical trials. Although RCTs are unavailable, there may be a very clear clinical consensus that a particular test or therapy is useful or effective.

* The outcome or result of the intervention should be specified (an improved clinical outcome or increased diagnostic accuracy or incremental prognostic information).

† For comparative-effectiveness recommendations (COR 1 and 2a; LOE A and B only), studies that support the use of comparator verbs should involve direct comparisons of the treatments or strategies being evaluated.

‡ The method of assessing quality is evolving, including the application of standardized, widely-used, and preferably validated evidence grading tools; and for systematic reviews, the incorporation of an Evidence Review Committee.

COR indicates Class of Recommendation; EO, expert opinion; LD, limited data; LOE, Level of Evidence; NR, nonrandomized; R, randomized; and RCT, randomized controlled trial.

**Table 2:** Relevant clinical practice documents

Title	Publication year

European Recommendations Integrating Genetic Testing into Multidisciplinary Management of Sudden Cardiac Death^2^	2019
2019 HRS/EHRA/APHRS/LAHRS Expert Consensus Statement on Catheter Ablation of Ventricular Arrhythmias^3^	2019
2019 HRS Expert Consensus Statement on Evaluation, Risk Stratification, and Management of Arrhythmogenic Cardiomyopathy^4^	2019
2018 ESC Guidelines for the Diagnosis and Management of Syncope^5^	2018
2017 AHA/ACC/HRS Guideline for Management of Patients with Ventricular Arrhythmias and the Prevention of Sudden Cardiac Death^6^	2017
Pre-participation Cardiovascular Evaluation for Athletic Participants to Prevent Sudden Death: Position Paper from the EHRA and the EACPR, Branches of the ESC^7^	2016
2015 ESC Guidelines for the Management of Patients with Ventricular Arrhythmias and the Prevention of Sudden Cardiac Death^8^	2015
EHRA/HRS/APHRS Expert Consensus on Ventricular Arrhythmias^9^	2014
HRS/EHRA/APHRS Expert Consensus Statement on the Diagnosis and Management of Patients with Inherited Primary Arrhythmia Syndromes^10^	2013
HRS/EHRA Expert Consensus Statement on the State of Genetic Testing for the Channelopathies and Cardiomyopathies^11^	2011

**Table 3:** Definitions

Term	Definition

Sudden cardiac arrest (SCA)	Sudden cessation of cardiac activity with hemodynamic collapse, typically due to sustained ventricular arrhythmia
Sudden cardiac death (SCD)	Death that occurs within 1 hour of onset of symptoms in witnessed cases, and within 24 hours of last being seen alive when it is unwitnessed
Sudden unexplained death (syndrome) (SUD[S])	Unexplained sudden death occurring in an individual older than 1 year
Sudden unexplained death in infancy (SUDI)	Unexplained sudden death occurring in an individual younger than 1 year with negative pathological and toxicological assessment Note: Synonymous with “sudden unexplained infant death” (SUID)
Sudden arrhythmic death (syndrome) (SAD[S])	Unexplained sudden death occurring in an individual older than 1 year with negative pathological and toxicological assessment Note: Synonymous with “autopsy-negative sudden unexplained death”
Sudden unexplained death in epilepsy (SUDEP)	Sudden and unexpected, nontraumatic and nondrowning death of a person with epilepsy, without a toxicological or anatomical cause of death detected during the postmortem examination

**Table 4:** Key goals of genetic counseling following sudden cardiac death/resuscitated sudden cardiac arrest

Goal	Description

Genetic counseling about inheritance risks	Provide information tailored specifically to the family about their inheritance risks.
Provide education and awareness	Educate about inheritance risks, the need for clinical surveillance, and options for genetic testing to allow the family to make subsequent important medical decisions. Conveying information is not straightforward, given varying health literacy and competing health concerns;however, genetic counseling can support effective communication.^108^ Genetic counseling can also include connection of families with advocacy organizations and relevant research studies.
Pre- and post-test genetic counseling	Explain the process and discuss the options of genetic testing, all possible outcomes of testing, implications for patients and/or their family members, and worries and fears about testing;ensure consideration of all possible results and implications.^109^ Care should be taken in conveying test results of uncertain significance,^101,110^ specifically ensuring adequate understanding and confidence to communicate key risk information to family members.
Pre- and post-test genetic counseling for cascade testing of asymptomatic relatives	There are ethical, legal, and social implications when considering cascade genetic testing of asymptomatic at-risk relatives. Careful pre-test genetic counseling should explore the individual’s feelings toward their risk, how they might feel if they are gene positive or gene negative, and implications for their own health and clinical management based on their genetic result. Discussion about the potential for reclassification of the genetic result is also important.^100,111^
Provide input regarding classification of genetic variants	Knowledge of variant and gene curation processes will enable review of any genetic test findings at all stages of family management.^112^ Clinicians involved in family management (including genetic counselors) are more likely to provide conservative variant classifications compared to clinical laboratories,^113^ and processes to guarantee regular review of variants will ensure appropriate reclassifications are made.^114,115^
Obtain detailed three-generation family history and confirm details	Record family history information in a pedigree and interpret the information and the risk posed to family members. Taking a detailed family history can allow development of rapport, elucidate family relationships and social circumstances, and inform clinical care.^116^
Assist with coordination of family clinical screening	Ensure adequate understanding of the clinical screening recommendations for family members and provide assistance with communicating this to relatives as needed. Provide support in organizing cardiology appointments with appropriate tests.^92^
Provide psychosocial support and identify when referral to clinical psychologist is required	Although genetic counseling is unlikely to resolve any significant psychopathologies, the process of providing information and a big picture perspective allowing a patient to normalize their experience and emotional response can have a positive impact, including patient empowerment.^103,106,107^

## Top 10 Take-Home Messages

1. Sudden cardiac death (SCD) is an important public health issue and warrants further study to better quantify its occurrence, its impact on society, and the opportunities for improving outcomes through public education and provision of automated external defibrillators and cardiopulmonary resuscitation (CPR) training.
2. For survivors of sudden cardiac arrest (SCA), victims of sudden unexplained death (SUD), and their relatives, a multidisciplinary team is central to thorough investigation, so as to maximize the opportunity to make a diagnosis. Where there has been an SCD or resuscitated SCA and a genetic cause is suspected, genetic testing and counseling is essential for families, to ensure that risks, benefits, results, and the clinical significance of genetic testing can be discussed.
3. The psychological care of families affected by SUD and survivors of SCA (and their families) should run in parallel with the investigation process. Assessment by professionals trained in psychological care should be offered, as well as grief counseling and peer support, where appropriate.
4. For the investigation of SUD, a detailed personal and family history is essential, with attention to sentinel symptoms during life such as syncope or seizures, witness accounts, premorbid investigations, and inspection of any cardiac rhythm monitoring around the time of death.
5. A comprehensive autopsy is an essential part of the investigation of SUD and should include collection and storage of tissue suitable for genetic analysis. When the autopsy suggests a possible genetic cause, or no cause and the heart is normal, referral to a multidisciplinary team for further investigation is indicated.
6. For victims of SCD or survivors of cardiac arrest where the phenotype is known, genetic testing of the proband focused on likely candidate genes, along with clinical evaluation of family members, aids in identifying family members with, or at risk of developing, the same condition.
7. For victims of SCD or survivors of cardiac arrest where the phenotype is not known, arrhythmia syndrome–focused genetic testing may help arrive at a secure diagnosis, whereas wider testing without careful consideration of the implications of indeterminate results by experienced clinicians may only serve to add uncertainty and lead to misinterpretation of results.
8. For the investigation of SCA survivors, essential inquiry includes detailed personal and family history, witness accounts, physical examination, multiple electrocardiograms (ECGs), and cardiac imaging. Ambulatory monitoring and/or provocative testing (exercise, pharmacological, and invasive electrophysiological) may provide additional useful information. A sample suitable for future DNA testing should be taken early in the patient’s course and stored.
9. Genetic investigation of SCA survivors is best undertaken at a center with multidisciplinary care infrastructure and should focus on likely candidate genes known to be causally related to the suspected phenotype. In some cases, genetic evaluation without a suspected phenotype may be undertaken with appropriate genetic counseling, although genetic evaluation of patients with a known nongenetic cause of cardiac arrest is discouraged.
10. The investigation of the families of victims of SUD and survivors of SCA should include clinical and, if known, genetic cascade testing. If the cause of SUD (or rarely, SCA) is unknown, then clinical investigation of first-degree relatives may include physical examination, ECGs, cardiac imaging, ambulatory monitoring, and provocative testing (exercise, pharmacological, and rarely invasive electrophysiological) with multidisciplinary team supervision. Follow-up and periodic re-evaluation are important and are directed by initial findings.

## Appendix 1 Author Disclosure Table

**Table:**

Writing group member	Employment	Honoraria/Speaking/Consulting	Speakers’ bureau	Research*	Fellowship support*	Ownership/Partnership/Principal/Majority stockholder	Stock or stock options	Intellectual property/Royalties	Other

Martin K. Stiles, MBChB, PhD, FHRS (APHRS Chair)	Waikato Clinical School, Faculty of Medicine and Health Science, The University of Auckland, Hamilton, New Zealand	None	None	None	None	None	None	None	None
Arthur A. M. Wilde, MD, PhD (HRS Chair)	Amsterdam University Medical Center, University of Amsterdam, Heart Center, Department of Clinical and Experimental Cardiology, Amsterdam, the Netherlands	None	None	None	None	None	None	None	None
Dominic J. Abrams, MBBS, MD, MBA	Boston Children’s Hospital, Boston, Massachusetts, USA	None	None	None	None	None	None	None	None
Michael J. Ackerman, MD, PhD	Mayo Clinic, Rochester, Minnesota, USA	0: Abbott; 0: Audentes Therapeutics; 0: Boston Scientific; 0: Gilead Sciences; 0: MyoKardia; 1: InVitae; 1: Medtronic	None	5: NIH	None	None	None	0: AliveCor; 0: Blue Ox Healthcare; 0: StemoniX	None
Christine M. Albert, MD, MPH, FHRS	Cedars-Sinai Medical Center, Los Angeles, California, USA	1: Roche Diagnostics	None	5: Abbott; 5: NIH; 5: Roche Diagnostics	None	None	None	None	None
Elijah R. Behr, MA, MBBS, MD	Cardiovascular Clinical Academic Group, Molecular and Clinical Sciences Institute, St George’s, University of London, and St George’s University Hospitals NHS Foundation Trust, London, United Kingdom	None	None	None	None	None	None	None	None
Sumeet S. Chugh, MD, FHRS	Cedars-Sinai Medical Center, Los Angeles, California, USA	None	None	5: NIH	4: Abbott; 4: BIOTRONIK; 4: Boston Scientific; 4: Medtronic	None	None	None	President, Cardiac Electrophysiology Society (2020–2022)
Martina C. Cornel, MD, PhD	Amsterdam University Medical Center, Vrije Universiteit Amsterdam, Clinical Genetics, Amsterdam Public Health Research Institute, Amsterdam, the Netherlands	None	None	None	None	None	None	None	0: European Society of Human Genetics (Vice Chair of Public and Professional Policy Committee); 0: VSOP (Dutch Genetic Alliance) (Board Membership)
Karen Gardner, MPH, PhD	University of New South Wales, Canberra, Australia	None	None	None	None	None	None	None	None
Jodie Ingles, GradDipGenCouns, PhD, MPH, FHRS	Agnes Ginges Centre for Molecular Cardiology at Centenary Institute, The University of Sydney, Sydney, Australia	None	None	3: MyoKardia	None	None	None	None	None
Cynthia A. James, ScM, PhD, CGC	Johns Hopkins University, Baltimore, Maryland, USA	None	None	1: NSGC; 2: Boston Scientific	None	None	None	None	0: NSGC (Chair of the Practice Guidelines Committee)
Jyh-Ming Jimmy Juang, MD, PhD	Cardiovascular Center and Division of Cardiology, Department of Internal Medicine, National Taiwan University Hospital and National Taiwan University College of Medicine, Taipei, Taiwan	None	None	None	None	None	None	None	None
Stefan Kääb, MD, PhD	Department of Medicine I, University Hospital, LMU Munich, Munich, Germany	1: AstraZeneca; 1: Bayer	None	1: BIOTRONIK; 1: Boston Scientific; 1: Medtronic	None	None	None	None	None
Elizabeth S. Kaufman, MD, FHRS	MetroHealth Campus, Case Western Reserve University, Cleveland, Ohio, USA	None	None	None	None	None	None	None	None
Andrew D. Krahn, MD, FHRS	The University of British Columbia, Vancouver, British Columbia, Canada	1: Medtronic	None	None	None	None	None	None	None
Steven A. Lubitz, MD, MPH	Massachusetts General Hospital, Boston, Massachusetts, USA	1: Bayer; 1: Bristol-Myers Squibb	None	5: AHA; 5: Bayer; 5: Boehringer Ingelheim; 5: Bristol-Myers Squibb; 5: Fitbit; 5: IBM; 5: NIH	None	None	None	None	None
Heather MacLeod, MS, CGC	Data Coordinating Center for the Sudden Death in the Young Case Registry, Okemos, Michigan, USA	None	None	None	None	None	None	None	None
Carlos A. Morillo, MD, FHRS	Libin Cardiovascular Institute, Calgary, Alberta, Canada	1: Abbott; 1: Medtronic; 1: Servier	None	None	None	None	None	None	None
Koonlawee Nademanee, MD, FHRS, CCDS	Chulalongkorn University, Faculty of Medicine, and Pacific Rim Electrophysiology Research Institute at Bumrungrad Hospital, Bangkok, Thailand	1: Biosense Webster	None	4: Medtronic	None	None	None	3: Biosense Webster	None
Vincent Probst, MD, PhD	CHU de Nantes, Nantes, France	1: BIOTRONIK; 1: Boston Scientific; 1: Medtronic	None	5: Boston Scientific	None	None	None	None	None
Elizabeth V. Saarel, MD, FHRS, CEPS-P	Cleveland Clinic Lerner College of Cardiology at Case Western Reserve University, Cleveland, Ohio, and St Luke’s Medical Center, Boise, Idaho, USA	None	None	None	None	None	None	None	None
Luciana Sacilotto, MD, PhD	Heart Institute, University of São Paulo Medical School, São Paulo, Brazil	None	None	None	None	None	None	None	None
Christopher Semsarian, MBBS, MPH, PhD, FHRS	Agnes Ginges Centre for Molecular Cardiology at Centenary Institute, The University of Sydney, Sydney, Australia	None	None	None	None	None	None	None	None
Mary N. Sheppard, MB, BCH, BAO, MD, FRCPath	Cardiovascular Clinical Academic Group, Molecular and Clinical Sciences Institute, St George’s, University of London, and St George’s University Hospitals NHS Foundation Trust, London, United Kingdom	None	None	None	None	None	None	None	AECVP (Past President)
Wataru Shimizu, MD, PhD	Department of Cardiovascular Medicine, Nippon Medical School, Tokyo, Japan	None	None	None	None	None	None	None	None
Jonathan R. Skinner, MBChB, MD, FHRS	Cardiac Inherited Disease Group, Starship Hospital, Auckland, New Zealand	None	None	None	None	None	None	None	None
Jacob Tfelt-Hansen, MD, DMSc	Department of Forensic Medicine, Faculty of Medical Sciences, Rigshospitalet, Copenhagen, Denmark	None	None	None	None	None	None	None	None
Dao Wu Wang, MD, PhD	The First Affiliated Hospital of Nanjing Medical University, Nanjing, China	None	None	None	None	None	None	None	None

Number value: **0** = $0; **1** = ≤ $10,000; **2** = > $10,000 to ≤ $25,000; **3** = > $25,000 to ≤ $50,000; **4** = > $50,000 to ≤ $100,000; **5** = > $100,000.

* Research and fellowship support are classed as programmatic support. Sources of programmatic support are disclosed but are not regarded as a relevant relationship with industry for writing group members or reviewers. AHA = American Heart Association; AECVP = Association for European Cardiovascular Pathology; NIH = National Institutes of Health; NSGC = National Society of Genetic Counselors.

## Appendix 2 Reviewer Disclosure Table

**Table:**

Peer reviewer	Employment	Honoraria/Speaking/Consulting	Speakers’ bureau	Research*	Fellowship support*	Ownership/Partnership/Principal/Majority stockholder	Stock or stock options	Intellectual property/Royalties	Other

Takeshi Aiba, MD, PhD	National Cerebral and Cardiovascular Center, Osaka, Japan	None	None	None	None	None	None	None	None
Allison L. Cirino, MS, CGC	Brigham and Women’s Hospital, Boston, Massachusetts, USA	None	None	None	None	None	None	None	None
Florence Fellmann, MD, PhD	The Collaboratory, University of Lausanne, Lausanne, Switzerland	None	None	None	None	None	None	None	None
Michael H. Gollob, MD	Toronto General Hospital, University of Toronto, Toronto, Canada	None	None	None	None	None	None	None	None
Yung-Kuo Lin, MD, PhD	Taipei Municipal Wanfang Hospital, Taipei Medical University, Taipei, Taiwan	None	None	None	None	None	None	None	None
Joaquín S. Lucena, MD, PhD	Institute of Legal Medicine and Forensic Sciences, Seville, Spain	None	None	None	None	None	None	None	Association for European Cardiovascular Pathology President (2018–2021)
Ciorsti MacIntyre, MD	QEII Health Science Centre, Halifax, Canada	1: Abbott; 1: Medtronic	None	0: Abbott	None	None	None	None	None
Jens Cosedis Nielsen, MD, DMSc, PhD, FESC, FEHRA	Aarhus University Hospital, Aarhus, Denmark	None	None	5: Novo Nordisk Foundation	None	None	None	None	None
Juan David Ramirez, MD	Clínica Cardio VID, Medellín, Colombia	None	None	None	None	None	None	None	0: Medtronic
Gregory Webster, MD, MPH, CCDS	Ann & Robert H. Lurie Children’s Hospital of Chicago, Chicago, Illinois, USA	None	None	4: AHA; 5: NIH	None	None	None	None	None

Number value: **0** = $0; **1** = ≤ $10,000; **2** = > $10,000 to ≤ $25,000; **3** = > $25,000 to ≤ $50,000; **4** = > $50,000 to ≤ $100,000; **5** = > $100,000.

* Research and fellowship support are classed as programmatic support. Sources of programmatic support are disclosed but are not regarded as a relevant relationship with industry for writing group members or reviewers. AHA = American Heart Association; NIH = National Institutes of Health.

## ABBREVIATIONS

AED: automated external defibrillator
CIED: cardiovascular implantable electronic device
CMR: cardiac magnetic resonance imaging
COR: Class of Recommendation
CPR: cardiopulmonary resuscitation
CPVT: catecholaminergic polymorphic ventricular tachycardia
CT: computed tomography
ECG: electrocardiogram
EMS: emergency medical services
LOE: Level of Evidence
MRI: magnetic resonance imaging
OHCA: out-of-hospital cardiac arrest
PVC: premature ventricular complex
RWI: relationship with industry and other entities
SAD(S): sudden arrhythmic death (syndrome)
SCA: sudden cardiac arrest
SCD: sudden cardiac death
SUD: sudden unexplained death
