# Supplementary material for: 2020 APHRS/HRS expert consensus statement on the investigation of decedents with sudden unexplained death and patients with sudden cardiac arrest, and of their families
Source: Heart Rhythm. Author manuscript; Available in PMC 2021 Jun 11. (PMC8194370; doi:10.1016/j.hrthm.2020.10.010)
Supplement: supplementary Appendix 3 [file NIHMS1704401-supplement-supplementary_Appendix_3.pdf]

Appendix 3 Evidence Table

Study name or acronym; Author; Year published; PMID	Aim of study; Endpoints; Study type (RCT, observational — multicenter or single center; case series or other [if RCT include intervention and comparator]); Study size (N)	Patient population with inclusion and exclusion criteria	Results (absolute event rates, *p* values; OR or RR; 95% CI)	Other relevant findings or adverse events	Limitations; Other comments; Conclusions
**Epidemiology: Sudden Death**					
Winkel et al. Nationwide study of sudden cardiac death in persons aged 1-35 years.<br>• Year published: 2011<br>• PMID: 21131293	Aim: To study the incidence of SCD in persons aged 1-35 years in a nationwide setting (5.38 million people) by systematic evaluation of all deaths.<br>Size: 625	Inclusion criteria: All deaths in persons aged 1-35 years in Denmark in 2000-2006 were included. 625 cases of sudden unexpected death were identified (10% of all deaths), of which 156 (25%) were not autopsied. Of the 469 autopsied cases, 314 (67%) were SCD.	The most common cardiac cause of death was ischemic heart disease (13%); 29% of autopsied sudden unexpected death cases were unexplained. In 45% of SCD cases, the death was witnessed; 34% died during sleep; 89% were out-of-hospital deaths.	Highest possible incidence rate of SCD in the young was 2.8 per 100,000 person-years including nonautopsied cases of sudden unexpected death. Excluding those, the incidence rate declined to 1.9 per 100,000 person-years.	A total of 7% of all deaths in the young can be attributed to SCD, when including nonautopsied cases (autopsy ratio 75%). The incidence rate of SCD in the young of 2.8 per 100,000 person-years is higher than previously reported.
Bagnall et al. A prospective study of sudden cardiac death among children and young adults.<br>• Year published: 2016<br>• PMID: 27332903	Aim: To define etiology of SCD in Australia and New Zealand 2010-2012<br>Endpoints: Clinical/genetic diagnosis in proband and family<br>Study type: Prospective population study<br>Size: 490 SCD cases	Inclusion criteria:<br>All SCD cases aged 1-35 years	SCD incidence in Australia/New Zealand was 1.3 per 100,000<br>Unexplained SCD more common in females, when occurring at night, and children aged 1-5 years.<br>54/490 cases were exertional or post-exercise.	Unexplained death the most common etiology; statistically associated with female sex and nocturnal SCD	Not enough evidence to include all cases. The study did not include resuscitated cardiac arrest cases.
Behr et al. Sudden arrhythmic death syndrome: a national survey of sudden unexplained cardiac death.	Aim: To describe the characteristics of SADS and compare its incidence with official national mortality statistics for unascertained deaths.<br>Size: 115	Inclusion criteria: Consecutive cases meeting the following criteria: white Caucasian, aged 4-64 years, no history of cardiac disease, last seen alive within 12 h of death, normal	56 (49%) SADS victims were identified: mean age 32 years, range 7-64 years and 35 (63%) male. 7 of 39 cases (18%) had a family history of other premature sudden deaths (<45). The estimated mortality from SADS was 0.16/100,000 per annum (95% CI 0.12 to 0.21), compared with an official mortality of 0.10/100,000 per annum	Deaths from SADS occur predominantly in young males.	When compared with official mortality, the incidence of SADS may be up to eight times higher than estimated: more than 500 potential SADS cases per annum in England. Families with SADS carry genetic cardiac disease, placing them at risk of further sudden deaths. SADS

**Study name or acronym; Author; Year published; PMID**	**Aim of study; Endpoints; Study type (RCT, observational — multicenter or single center; case series or other [if RCT include intervention and comparator]); Study size (N)**	**Patient population with inclusion and exclusion criteria**	**Results (absolute event rates, *p* values; OR or RR; 95% CI)**	**Other relevant findings or adverse events**	**Limitations; Other comments; Conclusions**
• Year published: 2007<br>• PMID: 17237131		coroner's autopsy, cardiac pathologist's confirmation of a normal heart and negative toxicology.	for International Classification of Diseases 798.1 (sudden death, cause unknown-instantaneous death) or 1.34/100,000 per annum for unascertained causes of death.		should therefore be a certifiable cause of death prompting specialized cardiological evaluation of families.
Becker et al. Racial differences in the incidence of cardiac arrest and subsequent survival. The CPR Chicago Project.<br>• Year published: 1993<br>• PMID: 8341333	Aim: To examine racial differences in the incidence of cardiac arrest in a large urban population and in subsequent survival.<br>Size: 6,451 patients	Inclusion criteria: Nontraumatic, OHCA in Chicago from January 1, 1987, through December 31, 1988. The study population comprised 6,451 patients: 3,207 whites, 2,910 blacks, and 334 persons of other races.	The incidence of cardiac arrest was significantly higher for blacks than for whites in every age group. The survival rate after cardiac arrest was 2.6% in whites, as compared with 0.8% in blacks ($p < .001$).	The association between race and survival persisted even when other recognized risk factors were taken into account. Important differences between blacks and whites in the response times of the EMS were not found.	The black community in the study was at higher risk for cardiac arrest and subsequent death than the white community, even after controlling for other variables.
Griffis et al. Characteristics and outcomes of AED use in pediatric cardiac arrest in public settings: the influence of neighborhood characteristics.<br>• Year published: 2020<br>• PMID: 31785372	Aim: To describe the association between bystander AED use, neighborhood characteristics and survival outcomes following public pediatric OHCA.<br>Size: 971 pediatric OHCA	Inclusion criteria: Nontraumatic OHCA among children less than 18 years of age in a public setting between from January 1, 2013 through December 31, 2017, were identified in the CARES database.	AEDs were used by bystanders in 10.3% of OHCA. AEDs were used on 2.3% of children ≤1 year (infants), 8.3% of 2-5 year-olds, 12.4% of 6-11 year-olds, and 18.2% of 12-18 year-olds ($p < .001$). AED use was more common in neighborhoods with a median household income of >$50,000 per year (12.3%; $p = .016$), <10% unemployment (12.1%; $p = .002$), and >80% high school education (11.8%; $p = .002$).	Greater survival to hospital discharge and neurologically favorable survival were among arrests with bystander AED use, varying by neighborhood characteristics.	Bystander AED use is uncommon in pediatric OHCA, particularly in high-risk neighborhoods, but improves survival. Further study is needed to understand disparities in AED use and outcomes.
Starks et al. Association of neighborhood demographics	Aim: To evaluate the association between bystander treatments (CPR and AED) and timing of EMS	Inclusion criteria: Patients with OHCA from January 1, 2008, to December 31, 2011,	The median age of patients with OHCA: 64 years (IQR 51-78). The percentage of patients with OHCA receiving bystander CPR or a lay	When the primary model included geographic site, there was an	Those with OHCA in predominantly black neighborhoods had the lowest rates of bystander CPR and AED

**Study name or acronym; Author; Year published; PMID**	**Aim of study; Endpoints; Study type (RCT, observational — multicenter or single center; case series or other [if RCT include intervention and comparator]); Study size (N)**	**Patient population with inclusion and exclusion criteria**	**Results (absolute event rates, *p* values; OR or RR; 95% CI)**	**Other relevant findings or adverse events**	**Limitations; Other comments; Conclusions**
with out-of-hospital cardiac arrest treatment and outcomes: where you live may matter.<br>• Year published: 2017<br>• PMID: 28854308	personnel on OHCA outcomes according to the racial composition of the neighborhood where the OHCA event occurred.<br>Main outcomes and measures: Survival to discharge, return of spontaneous circulation on ED arrival, and favorable neurologic status at discharge.<br>Study type: Retrospective observational cohort study<br>Size: 22,816 adult patients with nontraumatic OHCA	using data from the Resuscitation Outcomes Consortium. Neighborhoods where OHCA occurred were classified by census tract, based on percentage of black residents: less than 25%, 25% to 50%, 51% to 75%, or more than 75%.	automatic external defibrillation was inversely associated with the percentage of black residents in neighborhoods. Compared with OHCA in predominantly white neighborhoods (<25% black), those with OHCA in mixed to majority black neighborhoods had lower adjusted survival rates to hospital discharge (25%-50% black: odds ratio, 0.76; 95% CI, 0.61-0.93; 51%-75% black: odds ratio, 0.67; 95% CI, 0.49-0.90; >75% black: odds ratio, 0.63; 95% CI, 0.50-0.79; *p* < .001).	attenuated nonsignificant association between racial composition in a neighborhood and survival.	use and significantly lower likelihood for survival compared with predominantly white neighborhoods.
Zhao et al. Racial differences in sudden cardiac death.<br>• Year published: 2019<br>• PMID: 30712378	Aim: To compare the lifetime cumulative risk of SCD among blacks and whites, and to evaluate the risk factors that may explain racial differences in SCD risk in the general population.<br>Study type: Cohort<br>Size: 3,832 blacks and 11,237 whites	Inclusion criteria: 3,832 blacks and 11,237 whites participating in the Atherosclerosis Risk in Communities Study (ARIC).	The mean (SD) age was 53.6 (5.8) years for blacks and 54.4 (5.7) years for whites. During 27.4 years of follow-up, 215 blacks and 332 whites experienced SCD. The lifetime cumulative incidence of SCD at age 85 years was 9.6, 6.6, 6.5, and 2.3% for black men, black women, white men, and white women, respectively. The sex-adjusted hazard ratio for SCD comparing blacks with whites was 2.12 (95% CI, 1.79-2.51). The association was attenuated but still statistically significant in fully adjusted models (hazard ratio, 1.38; 95% CI, 1.11-1.71). In mediation analysis, known factors explained 65.3% (95% CI 37.9-92.8%) of the excess risk of SCD in blacks in comparison with whites.	The single most important factor explaining this difference was income (50.5%), followed by education (19.1%), hypertension (22.1%), and diabetes mellitus (19.6%). Racial differences were evident in both genders but stronger in women than in men.	Blacks had a much higher risk for SCD in comparison with whites, particularly among women. Income, education, and traditional risk factors explained ~65% of the race difference in SCD. The high burden of SCD and the racial-gender disparities observed in our study represent a major public health and clinical problem.

**Study name or acronym; Author; Year published; PMID**	**Aim of study; Endpoints; Study type (RCT, observational — multicenter or single center; case series or other [if RCT include intervention and comparator]); Study size (N)**	**Patient population with inclusion and exclusion criteria**	**Results (absolute event rates, *p* values; OR or RR; 95% CI)**	**Other relevant findings or adverse events**	**Limitations; Other comments; Conclusions**
**Epidemiology: Sudden Cardiac Arrest Survivors**					
McNally et al. Out-of-hospital cardiac arrest surveillance—Cardiac Arrest Registry to Enhance Survival (CARES), United States, October 1, 2005-December 31, 2010.<br>• Year published: 2011<br>• PMID: 21796098	Aim: To provide summary data from an OHCA surveillance registry in the United States.<br>Size: 31,689 OHCA events of presumed cardiac etiology	Inclusion criteria: During October 1, 2005-December 31, 2010, a total of 40,274 OHCA records were submitted to the CARES registry. 31,689 OHCA events of presumed cardiac etiology (e.g., myocardial infarction or arrhythmia) that received resuscitation efforts in the prehospital setting were analyzed.<br>Exclusion criteria: 8,585 noncardiac etiology arrests and missing hospital outcomes were excluded from the analysis.	The mean age at cardiac arrest was 64.0 years (SD 18.2); 61.1% of persons who experienced OHCA were male (n = 19,360). According to local EMS agency protocols, 21.6% of patients were pronounced dead after resuscitation efforts were terminated in the prehospital setting. The survival rate to hospital admission was 26.3%, and the overall survival rate to hospital discharge was 9.6%. Approximately 36.7% of OHCA events were witnessed by a bystander. Only 33.3% of all patients received bystander CPR, and only 3.7% were treated by bystanders with an AED before the arrival of EMS providers. The group most likely to survive an OHCA are persons who are witnessed to collapse by a bystander and found in a shockable rhythm (e.g., VF or pulseless VT). Among this group, survival to discharge was 30.1%.	A subgroup analysis was performed among persons who experienced OHCA events that were not witnessed by EMS personnel to evaluate rates of bystander CPR for these persons. In this group, whites were significantly more likely to receive CPR than blacks, Hispanics, or members of other racial/ethnic populations (*p* < .001).	CARES data confirm that patients who receive CPR from bystanders have a greater chance of surviving OHCA than those who do not.<br>Education of public officials and community members about the importance of increasing rates of bystander CPR and promoting the use of early defibrillation by lay and professional rescuers is critical to increasing survival rates. Reporting at the state and local levels can enable state and local public health and EMS agencies to coordinate their efforts to target improving emergency response for OHCA events, regardless of etiology, which can lead to improvement in OHCA survival rates.
Griffis et al. Characteristics and outcomes of AED use in pediatric cardiac arrest in public settings: the influence of neighborhood characteristics.	Aim: To describe the association between bystander AED use, neighborhood characteristics and survival outcomes following public pediatric OHCA.<br>Size: 971 pediatric OHCA	Inclusion criteria: Nontraumatic OHCA among children less than 18 years of age in a public setting between from January 1, 2013, through December 31, 2017, were identified in the CARES database.	AEDs were used by bystanders in 10.3% of OHCA. AEDs were used on 2.3% of children ≤1 year (infants), 8.3% of 2-5-year-olds, 12.4% of 6-11-year-olds, and 18.2% of 12-18-year-olds (*p* < .001). AED use was more common in neighborhoods with a median household income of >$50,000 per year (12.3%; *p* = .016), <10% unemployment (12.1%; *p* =	Greater survival to hospital discharge and neurologically favorable survival were among arrests with bystander AED use, varying by neighborhood characteristics.	Bystander AED use is uncommon in pediatric OHCA, particularly in high-risk neighborhoods, but improves survival. Further study is needed to understand disparities in AED use and outcomes.

Study name or acronym; Author; Year published; PMID	Aim of study; Endpoints; Study type (RCT, observational — multicenter or single center; case series or other [if RCT include intervention and comparator]); Study size (N)	Patient population with inclusion and exclusion criteria	Results (absolute event rates, *p* values; OR or RR; 95% CI)	Other relevant findings or adverse events	Limitations; Other comments; Conclusions
• Year published: 2020<br>• PMID: 31785372			.002), and >80% high school education (11.8%; *p* = .002).		
Starks et al. Association of neighborhood demographics with out-of-hospital cardiac arrest treatment and outcomes: where you live may matter.<br>• Year published: 2017<br>• PMID: 28854308	Aim: To evaluate the association between bystander treatments (CPR and AED) and timing of EMS personnel on OHCA outcomes according to the racial composition of the neighborhood where the OHCA event occurred.<br>Main outcomes and measures: Survival to discharge, return of spontaneous circulation on ED arrival, and favorable neurologic status at discharge.<br>Study type: Retrospective observational cohort study<br>Size: 22,816 adult patients with nontraumatic OHCA	Inclusion criteria: Patients with OHCA from January 1, 2008, to December 31, 2011, using data from the Resuscitation Outcomes Consortium. Neighborhoods where OHCA occurred were classified by census tract, based on percentage of black residents: less than 25%, 25% to 50%, 51% to 75%, or more than 75%.	The median age of patients with OHCA: 64 years (IQR 51-78). The percentage of patients with OHCA receiving bystander CPR or a lay automatic external defibrillation was inversely associated with the percentage of black residents in neighborhoods. Compared with OHCA in predominantly white neighborhoods (<25% black), those with OHCA in mixed to majority black neighborhoods had lower adjusted survival rates to hospital discharge (25%-50% black: odds ratio, 0.76; 95% CI, 0.61-0.93; 51%-75% black: odds ratio, 0.67; 95% CI, 0.49-0.90; >75% black: odds ratio, 0.63; 95% CI, 0.50-0.79; *p* < .001).	When the primary model included geographic site, there was an attenuated nonsignificant association between racial composition in a neighborhood and survival.	Those with OHCA in predominantly black neighborhoods had the lowest rates of bystander CPR and AED use and significantly lower likelihood for survival compared with predominantly white neighborhoods.
Becker et al. Racial differences in the incidence of cardiac arrest and subsequent survival. The CPR Chicago Project.<br>• Year published: 1993<br>• PMID: 8341333	Aim: To examine racial differences in the incidence of cardiac arrest in a large urban population and in subsequent survival.<br>Size: 6,451 patients	Inclusion criteria: Nontraumatic, OHCA in Chicago from January 1, 1987, through December 31, 1988. The study population comprised 6,451 patients: 3,207 whites, 2,910 blacks, and 334 persons of other races.	The incidence of cardiac arrest was significantly higher for blacks than for whites in every age group. The survival rate after cardiac arrest was 2.6% in whites, as compared with 0.8% in blacks (*p* < .001). The association between race and survival persisted even when other recognized risk factors were taken into account.	Important differences between blacks and whites in the response times of the EMS were not found.	The black community in the study was at higher risk for cardiac arrest and subsequent death than the white community, even after controlling for other variables.

**Study name or acronym; Author; Year published; PMID**	**Aim of study; Endpoints; Study type (RCT, observational — multicenter or single center; case series or other [if RCT include intervention and comparator]); Study size (N)**	**Patient population with inclusion and exclusion criteria**	**Results (absolute event rates, *p* values; OR or RR; 95% CI)**	**Other relevant findings or adverse events**	**Limitations; Other comments; Conclusions**
Hallstrom et al. Public-access defibrillation and survival after out-of-hospital cardiac arrest.<br>• Year published: 2004<br>• PMID: 15306665	Aim: The rate of survival after OHCA is low. It is not known whether this rate will increase if laypersons are trained to attempt defibrillation with the use of AEDs.<br>Endpoints: Survival to hospital discharge<br>Study type: Prospective, community-based, multicenter clinical trial<br>Size: More than 19,000 volunteer responders	Inclusion criteria: More than 19,000 volunteer responders from 993 community units in 24 North American regions participated, trained in CPR alone or in CPR and the use of AEDs.	Patients with treated OHCA in the two groups were similar in age (mean, 69.8 years), proportion of men (67%), rate of cardiac arrest in a public location (70%), and rate of witnessed cardiac arrest (72%). No inappropriate shocks were delivered. There were more survivors to hospital discharge in the units assigned to have volunteers trained in CPR plus the use of AEDs (30 survivors among 128 arrests) than there were in the units assigned to have volunteers trained only in CPR (15 among 107; *p* = .03; relative risk, 2.0; 95% CI, 1.07 to 3.77); there were only 2 survivors in residential complexes.	Functional status at hospital discharge did not differ between the two groups.	Training and equipping volunteers to attempt early defibrillation within a structured response system can increase the number of survivors to hospital discharge after OHCA in public locations. Trained laypersons can use AEDs safely and effectively.
Capucci et al. Tripling survival from sudden cardiac arrest via early defibrillation without traditional education in cardiopulmonary resuscitation.<br>• Year published: 2002<br>• PMID: 12196330	Aim: To improve public access to early defibrillation, we established Piacenza Progetto Vita (PPV), the first system of out-of-hospital early defibrillation by first-responder volunteers.<br>Size: 1,285	Inclusion criteria: 1,285 lay volunteers trained in use of the AED, without traditional education in cardiac pulmonary resuscitation, responded to all cases of suspected sudden cardiac arrest (SCA), in coordination with the EMS.	During the first 22 months, 354 SCA occurred (72 ± 12 years, 73% witnessed). The PPV volunteers treated 143 SCA cases (40.4%), with an EMS call-to-arrival time of 4.8 ± 1.2 min (versus 6.2 ± 2.3 min for EMS, *p* = .05). Overall survival rate to hospital discharge was tripled from 3.3% (7 of 211) for EMS intervention to 10.5% (15 of 143) for PPV intervention (*p* = .006). The survival rate for witnessed SCA was tripled by PPV: 15.5% versus 4.3% in the EMS-treated group (*p* = .002). A “shockable” rhythm was present in 23.8% (34 of 143) of the PPV patients versus 15.6% (33 of 211) of the EMS patients (*p* = .055). The survival rate from shockable dysrhythmias was higher for PPV versus EMS: 44.1% (15 of 34) versus 21.2% (7 of 33), *p* = .046.	The neurologically intact survival rate was higher in PPV-treated versus EMS-treated patients: 8.4% (12 of 143) versus 2.4% (5 of 211), *p* = .009.	Broad dissemination of AEDs for use by nonmedical volunteers enabled early defibrillation and tripled the survival rate for out-of-hospital SCA.

Study name or acronym; Author; Year published; PMID	Aim of study; Endpoints; Study type (RCT, observational — multicenter or single center; case series or other [if RCT include intervention and comparator]); Study size (N)	Patient population with inclusion and exclusion criteria	Results (absolute event rates, *p* values; OR or RR; 95% CI)	Other relevant findings or adverse events	Limitations; Other comments; Conclusions
**Multidisciplinary Team**					
Earle et al. Detection of sudden death syndromes in New Zealand. • Year published: 2016 • PMID: 27857240	Aim: To investigate regional variations in the detection of sudden death syndromes across New Zealand by assessing registrations in the national Cardiac Inherited Diseases Registry New Zealand (CIDRNZ). Size: 1,940	Registry data were extracted from the CIDRNZ in October 2015 and results are expressed as registrations per 100,000 people by district health board area.	The CIDRNZ has 1,940 registrants from 712 families, 46% of whom are definitely or probably affected by cardiac inherited disease. There are clear regional differences in registration frequencies between regions and between the North and South Islands, both for overall registrations (56/100,000 and 14/100,000, respectively; $p < .001$) and for LQTS registrations (15/100,000 and 6/100,000, respectively; $p < .001$). Regions with local coordinators have the highest number of registrations.		The detection of sudden death syndromes in New Zealand through a cardiac genetic registry is possible, but much work is needed to improve regional variation in the detection/reporting of these conditions across the country.
Ingles et al. Psychosocial impact of specialized cardiac genetic clinics for hypertrophic cardiomyopathy. • Year published: 2008 • PMID: 18281919	Aim: To describe the psychosocial factors associated with attending a specialty cardiac genetic clinic, and to determine whether these may be predictors of comorbid anxiety and depression in this population. Size: 109	Inclusion criteria: Questionnaires were sent to 184 individuals attending the Royal Prince Alfred Hospital Hypertrophic Cardiomyopathy Clinic.	Completed questionnaires were returned by 109 participants (59.2% response rate), of which 76.9% had a diagnosis of HCM, while 23.1% were at-risk relatives attending for clinical screening. Patient satisfaction scores were generally high to very high across all groups, though only 24% of HCM patients showed good adjustment to HCM and 10% had low worry about HCM scores.	Within the disease group, logistic regression analysis adjusting for age, gender, and education revealed adjustment to HCM and worry about HCM scores to be significantly associated with anxiety, while adjustment scores and location of patient follow-up (i.e., Hypertrophic Cardiomyopathy Clinic or another cardiologist) to be	HCM patients who attend specialized cardiac genetic clinics are better adjusted and worry less, than those who do not attend. An integrated approach, including a genetic counselor, is important in the management of HCM families.

**Study name or acronym; Author; Year published; PMID**	**Aim of study; Endpoints; Study type (RCT, observational — multicenter or single center; case series or other [if RCT include intervention and comparator]); Study size (N)**	**Patient population with inclusion and exclusion criteria**	**Results (absolute event rates, *p* values; OR or RR; 95% CI)**	**Other relevant findings or adverse events**	**Limitations; Other comments; Conclusions**
				significantly associated with depression scores.	
Tan et al. Sudden unexplained death: heritability and diagnostic yield of cardiological and genetic examination in surviving relatives.<br>• Year published: 2005<br>• PMID: 15998675	Aim: Because SUDs may have heritable causes, cardiological and genetic assessment of surviving relatives of SUD victims may reveal the underlying disease and unmask presymptomatic carriers. The study aimed to establish the diagnostic yield of such assessments.<br>Size: 43 families	Inclusion criteria: 43 consecutive families with > or =1 SUD victim who died at < or =40 years of age.	An inherited disease and likely cause of death in 17 of 43 families (40%) were identified; 12 families had primary electrical disease: CPVT (5 families), LQTS (4 families), BrS (2 families), and long-QT/BrS (1 family). ARVC (3 families), HCM (1 family), and FH (1 family) were also found. Molecular genetic analysis provided confirmation in 10 families.	Finding the diagnosis was more likely when more relatives were examined and in families with > or =2 SUD victims < or =40 years of age. The resting/ exercise ECG had a high diagnostic yield. These efforts unmasked 151 presymptomatic disease carriers (8.9 per family).	Examination of relatives of young SUD victims has a high diagnostic yield, with identification of the disease in 40% of families and 8.9 presymptomatic carriers per family. Simple procedures (examining many relatives) and routine tests (resting/exercise ECG) constitute excellent diagnostic strategies. Molecular genetics provide strong supportive information.
Behr et al. Cardiological assessment of first-degree relatives in sudden arrhythmic death syndrome.<br>• Year published: 2003<br>• PMID: 14602442	Aim: To search for evidence of inherited cardiac disease. in cases of SADS.<br>Size: 147	Inclusion criteria: 147 first-degree relatives of 32 people who died of SADS	Of 147 first-degree relatives of 32 people who died of SADS, 109 (74%) underwent cardiological assessment. Seven (22%) of the 32 families were diagnosed with inherited cardiac disease: 4 with LQTS; 1 with nonstructural cardiac electrophysiological disease; 1 with myotonic dystrophy; and 1 with HCM.		Families of people who die of SADS should be offered assessment in centers with experience of inherited cardiac disease.

**Study name or acronym; Author; Year published; PMID**	**Aim of study; Endpoints; Study type (RCT, observational — multicenter or single center; case series or other [if RCT include intervention and comparator]); Study size (N)**	**Patient population with inclusion and exclusion criteria**	**Results (absolute event rates, *p* values; OR or RR; 95% CI)**	**Other relevant findings or adverse events**	**Limitations; Other comments; Conclusions**
Kumar et al. Familial cardiological and targeted genetic evaluation: low yield in sudden unexplained death and high yield in unexplained cardiac arrest syndromes.<br>• Year published: 2013<br>• PMID: 23973953	Aim: To assess value of a clinical and genetic protocol for evaluating SCA patients.<br>Endpoints: Diagnosis<br>Study type: Case series<br>Size: 52 probands with UCA	Inclusion criteria: Unexplained SCA<br>Exclusion criteria: An identifiable noncardiac etiology; any evidence of coronary artery disease on electrocardiography (ECG) or coronary angiography; and abnormal ventricular function or valvular heart disease	Clinical diagnosis made in 32 (62%); genetic testing performed in 25/32 families (26 genes); pathogenic variant identified in 12/25 tested (yield 48%)	Mean age 32 years	In contrast to previously published series, a comprehensive strategy of cardiological evaluation and targeted genetic testing in more than 100 families with SADS was found to have a lower diagnostic yield (18%). Diagnostic yield in families with UCA was approximately 4 times higher (62%), which is consistent with the published literature.
Giudicessi et al. Assessment and validation of a phenotype-enhanced variant classification framework to promote or demote RYR2 missense variants of uncertain significance.<br>• Year published: 2019<br>• PMID: 31112425	Aim: To determine if a phenotype-enhanced variant classification approach could reduce the burden of RYR2 VUS encountered during clinical genetic testing.<br>Study type: Retrospective<br>Size: 84 RYR2-positive individuals from the Mayo Clinic and 149 RYR2-positive individuals from Amsterdam University Medical Center	Inclusion criteria: Conducted in 84 RYR2-positive individuals from the Mayo Clinic (Rochester, MN) and validated in 149 RYR2-positive individuals from Amsterdam University Medical Center.	Overall, 72 distinct RYR2 variants were identified among the 84 Mayo Clinic (39 unique) and 149 Amsterdam University Medical Center (30 unique) cases. Three variants were present in both cohorts. ACMG guidelines classified 47% of all RYR2 variants as VUS. In the Mayo Clinic cohort, readjudication using amended phenotype-enhanced ACMG standards dropped the VUS rate significantly (20/42 [48%] versus 3/42 [7%]; $p < .001$) with 13/20 (65%) RYR2 VUS promoted to likely pathogenic and 4/20 (20%) demoted to likely benign.	A similar drop in VUS rate (14/33 [42%] versus 3/33 [9%]; $p = .001$) was observed in the Amsterdam University Medical Center validation cohort with 10/14 (71%) RYR2 VUS promoted to likely pathogenic and 1/14 (7%) demoted to likely benign.	This multicenter study illustrates the potential utility of phenotype-enhanced variant classification in CPVT.

**Study name or acronym; Author; Year published; PMID**	**Aim of study; Endpoints; Study type (RCT, observational — multicenter or single center; case series or other [if RCT include intervention and comparator]); Study size (N)**	**Patient population with inclusion and exclusion criteria**	**Results (absolute event rates, *p* values; OR or RR; 95% CI)**	**Other relevant findings or adverse events**	**Limitations; Other comments; Conclusions**
Mellor et al. Genetic testing in the evaluation of unexplained cardiac arrest: from the CASPER (Cardiac Arrest Survivors with Preserved Ejection Fraction Registry).<br>• Year published: 2017<br>• PMID: 28600387	Aim: To assess role of genetic testing in individuals without a clinical phenotype.<br>Endpoints: Mutation carrier<br>Study type: Cross-sectional<br>Size: 174 genetic testing/ 375 cardiac arrest survivors in CASPER; genetic testing of cardiac genes at the discretion of the treating provider	Inclusion criteria: UCA with documented VT or VF requiring direct current cardioversion or defibrillation, and subsequent testing demonstrated normal left ventricular function (left ventricular ejection fraction ≥50%) and normal coronary arteries (no coronary stenosis >50%)<br>Exclusion criteria: Known causes for cardiac arrest, including an ECG diagnostic of LQTS (persistent resting QTc >460 ms for males and 480 ms for females) or BrS, HCM, marked hypokalemia, drug overdose, or commotio cordis, recognized forms of idiopathic VT	29 individuals with a pathogenic variant (17% yield); pathogenic variants identified in 18/72 (25%) of phenotype positive patients; pathogenic variants identified in 11/102 (11%) of phenotype negative patients	Mean age 39 years; 18% with ≥VUS; prior syncope (OR 4) and fam hx of SCA (OR 3.2) were associated with pathogenic variant carrier status	Nonstandardized genetic testing panels; no controls; no segregation data.<br>Prior syncope and family history of sudden death are predictors of a positive genetic test. Both arrhythmia and cardiomyopathy genes are implicated. Broad, multiphenotype testing revealed the highest frequency of pathogenic variants in phenotype-negative patients.
van der Werf et al. Diagnostic yield in sudden unexplained death and aborted cardiac arrest in the young: the experience of a tertiary referral	Aim: To find diagnosis in families with SUD victim (140 families) and in SCA survivors (69).<br>Study type: Cross-sectional	Inclusion criteria: SUD victim or SCA survivor aged 1-50 years	Diagnosis found in 33% of SUD families and in 61% of SCA survivors. Comprehensive cardiologic and genetic examination was performed in both populations.	ACA victims, 31 (74%) of the 42 diagnoses made were inherited cardiac diseases.	Inherited cardiac diseases are predominantly causative in both groups.

**Study name or acronym; Author; Year published; PMID**	**Aim of study; Endpoints; Study type (RCT, observational — multicenter or single center; case series or other [if RCT include intervention and comparator]); Study size (N)**	**Patient population with inclusion and exclusion criteria**	**Results (absolute event rates, *p* values; OR or RR; 95% CI)**	**Other relevant findings or adverse events**	**Limitations; Other comments; Conclusions**
center in the Netherlands.<br>• Year published: 2010<br>• PMID: 20646679					
**Counseling Families, the Bereaved and the Nearly Bereaved**					
Edwards et al. Interventions to improve risk communication in clinical genetics: systematic review.<br>• Year published: 2008<br>• PMID: 18207694	Aim: To undertake a systematic review of existing literature on risk communication in genetics and its effects on key outcomes for clients.<br>Study type: Review<br>Size: 28 studies	Inclusion criteria: Twenty-eight studies were included, principally from cancer genetics.	16 communication interventions have been evaluated, generally showing improvements in cognitive outcomes for users, such as knowledge, understanding and risk perception, and without adverse effects on anxiety, cancer-related worry and depression. However, often it was the supportive or emotional elements of counseling that provided benefits to users, rather than the informational or educational elements. Similar results were found in 12 further studies of decision aids which also appear to achieve shorter consultations that can focus more on the supportive elements of counseling.	For both communication models and decision aids, the supportive or emotional elements of counseling provided more benefits to users than the informational or educational elements.	Debate is required on how to strike a balance between the medical model, its agenda and perceived requirements to disclose or discuss a range of issues and the sometimes competing goals of addressing users' concerns, needs for support, issues of loss and relationship problems.
Michie et al. Genetic counselling: the psychological impact of meeting patients' expectations.<br>• Year published: 1997<br>• PMID: 9132497	Aim: To describe 131 consultations of patients referred to a regional genetics center, and documents their expectations, the extent to which these are met, and the predictors and consequences of expectations being met.	Inclusion criteria: Patients referred to a regional genetics center. The outcomes assessed were state anxiety, concern about the problem for which the patient was referred, and satisfaction with information given.	Patients came to GC expecting information (79%), explanation (63%), reassurance (50%), advice (50%), and help in making decisions (30%). The majority got what they were expecting: 74% had their expectation for information met, 56% had their expectation for explanation met, 60% had their expectation for reassurance met, 61% had their expectation for advice met, and 73% had their	Patient expectations, and whether or not these were met, were not predicted by any of the patient or counselor variables measured. When patients' expectations for reassurance and	Meeting patients' expectations for information, explanation, or help with decision making were not associated with better outcomes.

**Study name or acronym; Author; Year published; PMID**	**Aim of study; Endpoints; Study type (RCT, observational — multicenter or single center; case series or other [if RCT include intervention and comparator]); Study size (N)**	**Patient population with inclusion and exclusion criteria**	**Results (absolute event rates, *p* values; OR or RR; 95% CI)**	**Other relevant findings or adverse events**	**Limitations; Other comments; Conclusions**
	Size: 131 consultations		expectation for help with making decisions met.	advice were met, patients were less concerned, and their anxiety level was more reduced than when such expectations were not met.	
Ison et al. The impact of cardiovascular GC on patient empowerment.<br>• Year published: 2019<br>• PMID: 30680842	Aim: To investigate the effect of CVGC.<br>Size: 42	Inclusion criteria: 42 participants who completed pre- and post-GC surveys	The mean difference between pre- and post-GC empowerment scores was 17.5 points (mean pre-GC score = 118.5, mean post-GC score = 136, $p < .0001$; effect size, d = 0.94). Forty percent of individuals (17/42) were aware of surveillance recommendations for at-risk family members prior to GC; this increased to 76% of individuals (32/42) post-GC ($p < .01$).	This is the first study to explore patient empowerment before and after GC in a cardiology setting.	The results demonstrate a significant increase in empowerment and awareness of recommendations for at-risk relatives as a result of CVGC. This study demonstrates the utility of CVGC in patient care.
Liu et al. Genetic counselors' approach to postmortem genetic testing after sudden death: an exploratory study.<br>• Year published: 2018<br>• PMID: 31240068	Aim: To learn more about the experiences of genetic counselors who had considered or ordered postmortem genetic testing via a survey sent to the National Society of Genetic Counselors.	Survey sent to the National Society of Genetic Counselors	Cardiovascular genetic counselors were significantly more willing to recommend genetic testing in younger age decedents (ages 10, 18, 30, 40, and 50) compared to other specialty genetic counselors ($p < .05$, chi-square). Thirty-seven percent (7 of 19) of GCs reported insurance covering some portion of genetic testing.	Participants also reported highest success for DNA extractions with fresh and frozen blood, reinforcing NAME recommendations for appropriate sample collection for postmortem genetic testing.	Participating genetic counselors demonstrated a very good understanding for the appropriate use of postmortem genetic testing and did identify suspected barriers of cost and lack of insurance coverage as deterrents. With the rapid decrease in costs for diagnostic genetic testing, ME/C awareness of NAME recommendations for sample collection and storage remain important to facilitate postmortem genetic testing.

**Study name or acronym; Author; Year published; PMID**	**Aim of study; Endpoints; Study type (RCT, observational — multicenter or single center; case series or other [if RCT include intervention and comparator]); Study size (N)**	**Patient population with inclusion and exclusion criteria**	**Results (absolute event rates, *p* values; OR or RR; 95% CI)**	**Other relevant findings or adverse events**	**Limitations; Other comments; Conclusions**
Tan et al. Sudden unexplained death: heritability and diagnostic yield of cardiological and genetic examination in surviving relatives.<br>• Year published: 2005<br>• PMID: 15998675	Aim: Because SUDs may have heritable causes, cardiological and genetic assessment of surviving relatives of SUD victims may reveal the underlying disease and unmask presymptomatic carriers. The study aimed to establish the diagnostic yield of such assessments.<br>Size: 43 families	Inclusion criteria: 43 consecutive families with > or =1 SUD victim who died at < or =40 years of age.	An inherited disease and likely cause of death in 17 of 43 families (40%) were identified; 12 families had primary electrical disease: CPVT (5 families), LQTS (4 families), BrS (2 families), and long-QT/BrS (1 family). ARVC (3 families), HCM (1 family), and FH (1 family) were also found. Molecular genetic analysis provided confirmation in 10 families.	Finding the diagnosis was more likely when more relatives were examined and in families with > or =2 SUD victims < or =40 years of age. The resting/ exercise ECG had a high diagnostic yield. These efforts unmasked 151 presymptomatic disease carriers (8.9 per family).	Examination of relatives of young SUD victims has a high diagnostic yield, with identification of the disease in 40% of families and 8.9 presymptomatic carriers per family. Simple procedures (examining many relatives) and routine tests (resting/exercise ECG) constitute excellent diagnostic strategies. Molecular genetics provide strong supportive information.
Behr et al. Cardiological assessment of first-degree relatives in sudden arrhythmic death syndrome.<br>• Year published: 2003<br>• PMID: 14602442	Aim: To search for evidence of inherited cardiac disease in cases of SADS.<br>Size: 147	Inclusion criteria: 147 first-degree relatives of 32 people who died of SADS	Of 147 first-degree relatives of 32 people who died of SADS, 109 (74%) underwent cardiological assessment. Seven (22%) of the 32 families were diagnosed with inherited cardiac disease: 4 with LQTS; 1 with nonstructural cardiac electrophysiological disease; 1 with myotonic dystrophy; and 1 with HCM.		Families of people who die of SADS should be offered assessment in centers with experience of inherited cardiac disease.
Kumar et al. Familial cardiological and targeted genetic evaluation: low yield in sudden unexplained death and high yield in unexplained cardiac arrest syndromes.	Aim: To assess value of a clinical and genetic protocol for evaluating SCA patients.<br>Endpoints: Diagnosis<br>Study type: Case series<br>Size: 52 probands with UCA	Inclusion criteria: Unexplained SCA<br>Exclusion criteria: An identifiable noncardiac etiology, (2) any evidence of CAD on electrocardiography (ECG) or coronary angiography, and (3) abnormal ventricular	Clinical diagnosis made in 32 (62%); genetic testing performed in 25/32 families (26 genes); pathogenic variant identified in 12/25 tested (yield 48%)	Mean age 32 years	In contrast to previously published series, a comprehensive strategy of cardiological evaluation and targeted genetic testing in more than 100 families with SADS was found to have a lower diagnostic yield (18%). Diagnostic yield in families with UCA was approximately 4 times higher

Study name or acronym; Author; Year published; PMID	Aim of study; Endpoints; Study type (RCT, observational — multicenter or single center; case series or other [if RCT include intervention and comparator]); Study size (N)	Patient population with inclusion and exclusion criteria	Results (absolute event rates, *p* values; OR or RR; 95% CI)	Other relevant findings or adverse events	Limitations; Other comments; Conclusions
• Year published: 2013<br>• PMID: 23973953		function or valvular heart disease.			(62%), which is consistent with the published literature.
Mellor et al. Genetic testing in the evaluation of unexplained cardiac arrest: from the CASPER (Cardiac Arrest Survivors with Preserved Ejection Fraction Registry).<br>• Year published: 2017<br>• PMID: 28600387	Aim: To assess role of genetic testing in individuals without a clinical phenotype.<br>Endpoints: Mutation carrier<br>Study type: Cross-sectional<br>Size: 174 genetic testing/ 375 cardiac arrest survivors in CASPER; genetic testing of cardiac genes at the discretion of the treating provider	Inclusion criteria: UCA with documented VT or VF requiring direct current cardioversion or defibrillation, and subsequent testing demonstrated normal left ventricular function (left ventricular ejection fraction ≥50%) and normal coronary arteries (no coronary stenosis >50%)<br>Exclusion criteria: Known causes for cardiac arrest, including an ECG diagnostic of LQTS (persistent resting QTc >460 ms for males and 480 ms for females) or BrS, HCM, marked hypokalemia, drug overdose, or commotio cordis, recognized forms of idiopathic VT	29 individuals with a pathogenic variant (17% yield); pathogenic variants identified in 18/72 (25%) of phenotype positive patients; pathogenic variants identified in 11/102 (11%) of phenotype negative patients	Mean age 39 years; 18% with ≥VUS; prior syncope (OR 4) and fam hx of SCA (OR 3.2) were associated with pathogenic variant carrier status	Nonstandardized genetic testing panels; no controls; no segregation data.<br>Prior syncope and family history of sudden death are predictors of a positive genetic test. Both arrhythmia and cardiomyopathy genes are implicated. Broad, multiphenotype testing revealed the highest frequency of pathogenic variants in phenotype-negative patients.
van der Werf et al. Diagnostic yield in sudden unexplained death and aborted cardiac arrest in	Aim: To find diagnosis in families with SUD victim (140 families) and in SCA survivors (69).<br>Study type: Cross-sectional	Inclusion criteria: SUD victim or SCA survivor aged 1-50 years	Diagnosis found in 33% of SUD families and in 61% of SCA survivors. Comprehensive cardiologic and genetic examination was performed in both populations.	ACA victims, 31 (74%) of the 42 diagnoses made were inherited cardiac diseases.	Inherited cardiac diseases are predominantly causative in both groups.

**Study name or acronym; Author; Year published; PMID**	**Aim of study; Endpoints; Study type (RCT, observational — multicenter or single center; case series or other [if RCT include intervention and comparator]); Study size (N)**	**Patient population with inclusion and exclusion criteria**	**Results (absolute event rates, *p* values; OR or RR; 95% CI)**	**Other relevant findings or adverse events**	**Limitations; Other comments; Conclusions**
the young: the experience of a tertiary referral center in the Netherlands.<br>• Year published: 2010<br>• PMID: 20646679					
**Psychological Care**					
Ingles et al. Posttraumatic stress and prolonged grief after the sudden cardiac death of a young relative.<br>• Year published: 2016<br>• PMID: 26809585	Aim: To assess psychological functioning, including PG and PTS symptoms, and to identify factors that correlate with adverse outcomes after the SCD of a young relative.<br>Size: 103 participants from 57 families	Inclusion criteria: Adults who had a relative 45 years or younger who died suddenly without a premorbid diagnosis and thorough postmortem examination either diagnostic of a GHD or resulting in an unascertained cause of death and who were first-degree relatives of the decedent	PG was reported by 19 (20.6%) participants, with a mean (SD) time since death of 5 (3) years (range, 0.5-10 years), and the proportion was higher among mothers of the decedent (10 [35.7%]) and those who were witnesses to the death (10 [41.7%]). PTS symptoms were reported by 44 (44%) participants, and the proportion was higher among mothers of the decedent (19 [59.4%]) and those who were witnesses to the death (18 [66.7%]). Participants reported more severe depression (mean [SD] score, 4.2 [4.5] vs 2.6 [3.9]; $p < .001$), anxiety (mean [SD] score, 2.9 [3.2] vs 1.7 [2.8]; $p < .001$), and stress (mean [SD] score, 5.3 [3.9] vs 4.0 [4.2]; $p = .004$) than the general population.	Witnessing the death was strongly associated with both PG and PTS symptoms after adjusting for family clustering, maternal relationship, and time since death.	Almost 1 in 2 family members reported significant psychological difficulties, particularly PG and PTS symptoms. Both PG and PTS are associated with poor health outcomes, and there is extensive evidence for the efficacy of psychological treatments for persons in need of intervention for these conditions. The study suggests that specialized input from a clinical psychologist who can triage ongoing treatment needs is critical in the multidisciplinary clinic setting.
Yeates et al. Poor psychological wellbeing particularly in mothers following	Aim: To evaluate psychological wellbeing and experiences of at-risk relatives following SCD in the young.	Inclusion criteria: Relatives who attended a specialized clinic following the SCD of a relative were invited to	The mean time since death was 4 ± 2 years (mean age at death 23 ± 10 years, 79% males). There was significant impairment in mean anxiety (8.7 ± 4.3, $p < .0001$) and depression (5.8 ± 3.6, $p <$		The SCD of a young relative has significant and long-term emotional implications for the family, particularly for the mother.

Study name or acronym; Author; Year published; PMID	Aim of study; Endpoints; Study type (RCT, observational — multicenter or single center; case series or other [if RCT include intervention and comparator]); Study size (N)	Patient population with inclusion and exclusion criteria	Results (absolute event rates, *p* values; OR or RR; 95% CI)	Other relevant findings or adverse events	Limitations; Other comments; Conclusions
sudden cardiac death in the young.<br>• Year published: 2013<br>• PMID: 23568895	Size: 50 relatives from 29 families	complete the Hospital Anxiety and Depression Scale (HADS) and a series of open-ended questions.	.0001) scores compared to the general population. Mothers showed significantly impaired anxiety (10.9 ± 4.0, *p* = .001) and depression (7.3 ± 3.3, *p* = .001) scores, with 53% having an anxiety score above 11 suggesting probable anxiety disorder.		
McDonald et al. Needs analysis of parents following sudden cardiac death in the young.<br>• Year published: 2020<br>• PMID: 32709698	Aim: To investigate the needs of parents who have experienced the SCD of their child (≤45 years).<br>Study type: A quantitative needs analysis questionnaire was developed based on semistructured interviews, including one focus group and a review of relevant literature. Eligible participants were invited to participate in this cross-sectional survey study.	There were 38 parents who completed a quantitative survey.	Parents' perceived needs for information and support spanned medical, psychosocial, spiritual, and financial domains. Of the support and information needs assessed, medical needs were identified as the most important domain, followed by psychosocial, spiritual, and financial. Importantly, psychosocial information and support needs were reported as the most unmet need, endorsed by 54% of parents. Medical information and support needs were reported as unmet by almost one-third of parents. The two most endorsed needs were "To have the option of whether or not you would pursue genetic testing for yourself or family members" and "To understand what happened."	This work demonstrates for the first time, the multifactorial needs of parents after SCD in the young.	With the greatest unmet need reported as psychosocial needs, there is clear necessity to find ways of integrating psychological support in to the care of families after SCD in the young.
Farnsworth et al. When I go in to wake them ... I wonder: parental perceptions about congenital long QT syndrome.	Aim: To describe the experiences of parents who have a child or children with LQTS.<br>Study type: Secondary analysis of a 2002 qualitative phenomenological primary study	Secondary analysis of a 2002 qualitative phenomenological primary study done to explore fear of death and quality of life for 58 patients with LQTS. The secondary study analyzed responses	Results of the study revealed that parents with young children described fear of their children dying and strategies they used to manage their fear, as well as frustrations about lack of knowledge of LQTS among health care providers. When the diagnosis of LQTS is established during adolescence, the impact on the lives	In order to support families experiencing the stress of living daily with someone with LQTS, health care providers including nurse practitioners need a better	Further studies are needed to understand the long-term psychosocial effects of children on beta-blockers, children with ICDs, and children, adolescents, and young adults who survive a sudden death event.

Study name or acronym; Author; Year published; PMID	Aim of study; Endpoints; Study type (RCT, observational — multicenter or single center; case series or other [if RCT include intervention and comparator]); Study size (N)	Patient population with inclusion and exclusion criteria	Results (absolute event rates, *p* values; OR or RR; 95% CI)	Other relevant findings or adverse events	Limitations; Other comments; Conclusions
• Year published: 2006<br>• PMID: 16719847		derived from 31 parents of children with LQTS.	of children and their families is more significant.	understanding of the symptoms, diagnosis, management, and lifestyle implications of LQTS.	
Wisten et al. Supportive needs of parents confronted with sudden cardiac death—a qualitative study.<br>• Year published: 2007<br>• PMID: 17353083	Aim: To elucidate the perceived support and the needs of bereaved parents confronted with SCD in a young son or daughter.<br>Study type: Data were derived from a qualitative contents analysis of tape-recorded, in-depth interviews with bereaved parents confronted with SCD 5-12 years post-loss.	The 20 deceased individuals were part of the Swedish forensic SCD cohort of 15-35-year-olds from 1992 to 1999.	One-third of the parents had had no contact with the ED, one-third had been disappointed after meeting caregivers at the ED who did not act with sensitivity and consistency, while one-third were more or less satisfied with the handling at the ED. A majority of the parents experienced a lack of follow-up care; they had been left mainly to themselves to find information and support.	Four factors were identified as being particularly important for the parents: evidence, reconstruction, explanation and sensitivity.	There is a need of better routines to help the suddenly bereaved.
Solomon et al. Peer support/peer provided services underlying processes, benefits, and critical ingredients.<br>• Year published: 2004<br>• PMID: 15222150	Aim: To define peer support/peer provided services; discuss the underlying psychosocial processes of these services; and delineate the benefits to peer providers, individuals receiving services, and mental health service delivery system.		Based on these theoretical processes and research, the critical ingredients of peer provided services, critical characteristics of peer providers, and mental health system principles for achieving maximum benefits are discussed, along with the level of empirical evidence for establishing these elements.		
Landers et al. An analysis of relationships among peer support,	Aim: To investigate how peer support relates to psychiatric hospitalization and crisis stabilization utilization outcomes.	The likelihood of experiencing a psychiatric hospitalization or a crisis stabilization was	Peer support was associated with an increased likelihood (odds = 1.345) of crisis stabilization, a decreased but statistically insignificant likelihood (odds = 0.871) of psychiatric		

Study name or acronym; Author; Year published; PMID	Aim of study; Endpoints; Study type (RCT, observational — multicenter or single center; case series or other [if RCT include intervention and comparator]); Study size (N)	Patient population with inclusion and exclusion criteria	Results (absolute event rates, *p* values; OR or RR; 95% CI)	Other relevant findings or adverse events	Limitations; Other comments; Conclusions
psychiatric hospitalization, and crisis stabilization.<br>• Year published: 2011<br>• PMID: 19551502		modeled for consumers using peer support services and a control group of consumers using community mental health services but not peer support with 2003 and 2004 Georgia Medicaid claims data; 2003 and 2004 Mental Health, Developmental Disability, and Addictive Diseases (MHDDAD) Community Information System data; and 2003 and 2004 MHDDAD Hospital Information System data.	hospitalization overall, and a decreased and statistically significant (odds = 0.766) likelihood of psychiatric hospitalization for those who did not have a crisis stabilization episode.		
Bartone et al. Peer support services for bereaved survivors: a systematic review.<br>• Year published: 2019<br>• PMID: 28871835	Aim: To assess the evidence regarding benefits of peer support services for bereaved survivors of sudden or unexpected death.<br>Study type: Systematic literature review<br>Size: 32 studies	Inclusion criteria: Reports that addressed peer support services for adults who experienced death of a family member, close friend, or coworker	Most studies showed evidence that peer support was helpful to bereaved survivors, reducing grief symptoms and increasing well-being and personal growth. Studies also showed benefits to providers of peer support, including increased personal growth and positive meaning in life. Several studies addressed the growing trend of Internet-based peer support programs, finding that these are beneficial in part due to their easy accessibility.	Peer support appears to be especially valuable for survivors of suicide loss, a result that may be related to stigma and lack of support from family and friends experienced by many suicide survivors.	Peer support is beneficial to bereaved survivors.

Study name or acronym; Author; Year published; PMID	Aim of study; Endpoints; Study type (RCT, observational — multicenter or single center; case series or other [if RCT include intervention and comparator]); Study size (N)	Patient population with inclusion and exclusion criteria	Results (absolute event rates, *p* values; OR or RR; 95% CI)	Other relevant findings or adverse events	Limitations; Other comments; Conclusions
**Investigation of Sudden Death: History—Personal and Family**					
Bagnall et al. A prospective study of sudden cardiac death among children and young adults.<br>• Year published: 2016<br>• PMID: 27332903	Aim: To define etiology of SCD in Australia and New Zealand 2010-2012.<br>Endpoints: Clinical/genetic diagnosis in proband and family<br>Study type: Prospective population study<br>Size: 490 SCD cases	Inclusion criteria:<br>All SCD cases aged 1-35 years	SCD incidence in Australia/New Zealand was 1.3 per 100,000 Unexplained SCD more common in females, when occurring at night, and children aged 1-5 years.<br>54/490 cases were exertional or post-exercise.	Unexplained death the most common etiology; statistically associated with female sex and nocturnal SCD	Not enough evidence to include all cases. The study did not include resuscitated cardiac arrest cases.
Risgaard et al. Sudden cardiac death: pharmacotherapy and proarrhythmic drugs: a nationwide cohort study in Denmark.<br>• Year published: 2017<br>• PMID: 29759603	Aim: To describe the use of pharmacotherapy in national survey of SCD.<br>Endpoints: Use of medication related to SCD<br>Study type: Retrospective population study of all deaths 2000-2009 and associated medication use<br>Size: 1,363 SCD cases	Inclusion criteria:<br>All SCD cases between 2000-2009 aged 1-35 years and SCD cases aged 36-49 years between 2007-2009	-58% of SCD received 1 drug in 90 days prior to SCD<br>-Use of drugs associated with BrS or LQTS associated with 2-3x increased risk of sudden arrhythmic death	Most commonly used agents with analgesic, anti-hypertensive and antibiotics	Single national population with homogeneity; issues re 90 day prior to death; observational and retrospective; assumption regarding use of prescribed drugs
Glinge et al. Symptoms before sudden arrhythmic death syndrome: a nationwide study among the young in Denmark.<br>• Year published: 2015<br>• PMID: 25807988	Aim: To identify symptoms and medical history prior to SCD.<br>Endpoints: Reported symptoms prior to death<br>Study type: Retrospective population study of all deaths 2000-2006<br>Size: 136 SCD (SADS) cases	Inclusion criteria:<br>All SCD (SADS) cases between 2000-2006 with no pathological or toxicological cause of death aged 1-35 years	-48 (35%) had symptoms prior to death<br>-34 (25%) ≥24 hours prior to death<br>-23 (17) ≤24 hours prior to death<br>-Syncope/pre-syncope the most common	-Seizures were present in 25 (18%)<br>-Prior aborted SCD in 2<br>-Only 1 in 5 reported these symptoms to a health care provider	- Retrospective<br>-Majority deaths unwitnessed therefore potential under reporting<br>-Recall bias<br>-Missing clinical information
Winkel et al. Sudden cardiac death in children	Aim: To define etiology of SCD in children in Denmark.<br>Endpoints: Postmortem	Inclusion criteria:<br>All SCD cases between	-Seizures, dyspnea and syncope most common symptoms<br>-Prodromal symptoms in 45%	-1.1 per 100,000 cardiac sudden death rate	-Retrospective<br>-No access to family history<br>-Incomplete autopsy rate

**Study name or acronym; Author; Year published; PMID**	**Aim of study; Endpoints; Study type (RCT, observational — multicenter or single center; case series or other [if RCT include intervention and comparator]); Study size (N)**	**Patient population with inclusion and exclusion criteria**	**Results (absolute event rates, *p* values; OR or RR; 95% CI)**	**Other relevant findings or adverse events**	**Limitations; Other comments; Conclusions**
(1-18 years): symptoms and causes of death in a nationwide setting.<br>• Year published: 2014<br>• PMID: 24344190	diagnosis and antemortem symptoms<br>Study type: Observational, national study<br>Size: 114 SUD cases	2000-2006 aged 1-18 years	-Antecedent symptoms in 45%<br>-43 (49%) had probable inherited cardiac etiology	-77% autopsy rate<br>-114 (7.5%) sudden death	
Lahrouchi et al. Utility of post-mortem genetic testing in cases of sudden arrhythmic death syndrome.<br>• Year published: 2017<br>• PMID: 28449774	Aim: To define use of postmortem genetic testing following SCD in negative autopsy SCD.<br>Endpoints: Genetic diagnosis in proband and family<br>Study type: Multicenter, retrospective case-control study<br>Size: 302 SCD cases	Inclusion criteria: SCD cases with available DNA and negative autopsy; negative autopsy<br>Exclusion: Structural heart disease or nonspecific pathological features at autopsy	-Death with exercise (10%) or extreme emotion (1.5%)<br>-Family history in 7.1%<br>-18% personal history of syncope or seizures<br>-21 (7%) diagnosed with epilepsy or prior history of epilepsy<br>-24 had consulted health care provider	-19 pathogenic and 15 likely pathogenic variants in RyR2, KCNH2, KCNQ1, SCN5A<br>-1 pathogenic variant in PLN, likely pathogenic versions in TTN (2), PKP2 (1), MYH7 (1)	-88% European heritage<br>-Limited family evaluation<br>-Referral bias
Behr et al. Cardiological assessment of first-degree relatives in sudden arrhythmic death syndrome.<br>• Year published: 2003<br>• PMID: 14602442	Aim: To search for evidence of inherited cardiac disease in cases of SADS.<br>Size: 147	Inclusion criteria: 147 first-degree relatives of 32 people who died of SADS	Of 147 first-degree relatives of 32 people who died of SADS, 109 (74%) underwent cardiological assessment. Seven (22%) of the 32 families were diagnosed with inherited cardiac disease: 4 with LQTS; 1 with nonstructural cardiac electrophysiological disease; 1 with myotonic dystrophy; and 1 with HCM.		Families of people who die of SADS should be offered assessment in centers with experience of inherited cardiac disease.
Behr et al. Sudden arrhythmic death syndrome: familial evaluation	Aim: To identify more susceptible SADS individuals and causes of death through comprehensive clinical	Inclusion criteria: Consecutively referred families with SADS death	First-degree relatives [184/262 (70%)] underwent evaluation, 13 (7%) reporting unexplained syncope. 17 (30%) families had a history of	1 SCN5A and 4 KCNH2 mutations (38%) were identified in 13	Over-half of SADS deaths were likely to be due to inherited heart disease; accurate identification is vital for

**Study name or acronym; Author; Year published; PMID**	**Aim of study; Endpoints; Study type (RCT, observational — multicenter or single center; case series or other [if RCT include intervention and comparator]); Study size (N)**	**Patient population with inclusion and exclusion criteria**	**Results (absolute event rates, *p* values; OR or RR; 95% CI)**	**Other relevant findings or adverse events**	**Limitations; Other comments; Conclusions**
identifies inheritable heart disease in the majority of families.<br>• Year published: 2008<br>• PMID: 18508782	investigation of SADS families.<br>Size: 57 families		additional unexplained premature sudden death(s). 30 families (53%) were diagnosed with inheritable heart disease: 13 definite LQTS, 3 possible/probable LQTS, 5 BrS, 5 ARVC, and 4 other cardiomyopathies.	definite LQTS families, 1 SCN5A mutation (20%) in five BrS families and one (25%) PKP2 (plakophyllin2) mutation in 4 ARVC families.	appropriate prophylaxis amongst relatives who should undergo comprehensive cardiological evaluation, guided and confirmed by mutation analysis.
Waddell-Smith et al. Inpatient detection of cardiac-inherited disease: the impact of improving family history taking.<br>• Year published: 2016<br>• PMID: 26925241	Aim: To evaluate adequacy of eliciting family history by adult cardiology inpatient teams.<br>Endpoints: Elicit family history of inherited cardiac disease<br>Size: 37	Inclusion criteria:<br>ACA, cardiomyopathy, or VT	37 patients (22 males) were selected: mean age 51 years (range 15-79). Admission presentations included (idiopathic) RSCD (14), dyspnea or heart failure (11), VT (2), other (10). 3 patients had already volunteered their familial diagnosis to the admitting team.	Family history was incompletely elicited in 17 (46%) and absent in 20 (54%).	Adult cardiology inpatient teams are poor at recording family history and need to be reminded of its powerful diagnostic value.
Steinberg et al. Cardiac abnormalities in first-degree relatives of unexplained cardiac arrest victims: a report from the cardiac arrest survivors with preserved ejection fraction registry.<br>• Year published: 2016<br>• PMID: 27635072	Aim: To prospectively assess first-degree relatives of UCA or SUD victims to screen for cardiac abnormalities.<br>Size: 398	Inclusion criteria:<br>Around 398 first-degree family members (186 UCA, 212 SUD victims' relatives; mean age, 44 ± 17 years) underwent extensive cardiac workup, including ECG, signal averaged ECG, exercise testing, cardiac imaging, Holter monitoring, and selective provocative drug testing with epinephrine or procainamide.	Cardiac abnormalities were detected in 120 of 398 patients (30.2%) with 67 of 398 having a definite or probable diagnosis (17%), including LQTS (13%), CPVT (4%), ARVC (4%), and BrS (3%). The detection yield was similar for family members of UCA and SUD victims (31% versus 27%; *p* = .59). Genetic testing was performed more often in family members of UCA patients (29% versus 20%; *p* = .03). Disease-causing mutations were identified in 20 of 398 relatives (5%).	The most common pathogenic mutations were RyR2 (2%), SCN5A (1%), and KNCQ1 (0.8%).	Cardiac screening revealed abnormalities in 30% of first-degree relatives of UCA or SUD victims, with a clear working diagnosis in 17%. Long-QT, ARVC, and CPVT were the most common diagnoses. Systematic cascade screening and genetic testing in asymptomatic individuals will lead to preventive lifestyle and medical interventions with potential to prevent sudden cardiac death.

Study name or acronym; Author; Year published; PMID	Aim of study; Endpoints; Study type (RCT, observational — multicenter or single center; case series or other [if RCT include intervention and comparator]); Study size (N)	Patient population with inclusion and exclusion criteria	Results (absolute event rates, *p* values; OR or RR; 95% CI)	Other relevant findings or adverse events	Limitations; Other comments; Conclusions
Raju et al. Low prevalence of risk markers in cases of sudden death due to Brugada syndrome relevance to risk stratification in Brugada syndrome.<br>• Year published: 2011<br>• PMID: 21636035	Aim: To determine the prevalence of conventional risk factors in sudden arrhythmic death syndrome (SADS) probands with BrS.<br>Size: 49 families, 202 family members in total (50 probands)	Inclusion criteria: A total of 49 consecutive families with a confirmed SADS death and a diagnosis of BrS were evaluated, comprising assessment of 202 family members in total. One family had 2 members with SADS, resulting in a total of 50 probands included.	Mean age of death of probands was 29.1 ± 10.6 years, with 41 males (82%) (*p* < .05). Antemortem ECGs were available for 5 SADS probands, 1 of which demonstrated a spontaneous type 1 pattern. In 45 probands, symptoms before death were reported reliably by family members. Of these, 9 (20%) had experienced at least 1 syncopal episode before the fatal event. 68% of probands would not have fulfilled any current criteria for consideration of ICD.		The “low-risk” asymptomatic BrS group comprises the majority of SCD in this cohort. Current risk stratification would appear to be inadequate, and new markers of risk are vital.
**Investigation of Sudden Death: Examination of Premorbid Investigations**					
Algra et al. QTc prolongation measured by standard 12-lead electrocardiography is an independent risk factor for sudden death due to cardiac arrest.<br>• Year published: 1991<br>• PMID: 2040041	Aim: QTc prolongation has been implicated as a risk factor for sudden death; however, a controversy exists over its significance.<br>Size: 6,693 patients	In the Rotterdam QT Project, 6,693 consecutive patients who underwent 24-hour ambulatory electrocardiography were followed up for 2 years; of these, 245 patients died suddenly.	In patients without evidence of cardiac dysfunction (history of symptoms of pump failure or an ejection fraction less than 40%), QTc of more than 440 ms was associated with a 2.3 times higher risk for sudden death compared with a QTc of 440 ms or less (95% confidence interval: 1.4, 3.9). In contrast, in patients with evidence of cardiac dysfunction, the relative risk of QTc prolongation was 1.0 (0.5, 1.9).	Adjustment for age, gender, history of myocardial infarction, heart rate, and the use of drugs did not alter these relative risks.	In patients without intraventricular conduction defects and cardiac dysfunction, QTc prolongation measured from the standard ECG is a risk factor for sudden death independent of age, history of myocardial infarction, heart rate, and drug use. In patients with cardiac dysfunction, QTc duration is not related to the risk for sudden death.

**Study name or acronym; Author; Year published; PMID**	**Aim of study; Endpoints; Study type (RCT, observational — multicenter or single center; case series or other [if RCT include intervention and comparator]); Study size (N)**	**Patient population with inclusion and exclusion criteria**	**Results (absolute event rates, *p* values; OR or RR; 95% CI)**	**Other relevant findings or adverse events**	**Limitations; Other comments; Conclusions**
Brugada et al. Right bundle-branch block and ST-segment elevation in leads V1 through V3: a marker for sudden death in patients without demonstrable structural heart disease.<br>• Year published: 1998<br>• PMID: 9490240	Aim: Information on long-term outcome became available due to pooled data on a large cohort of patients who are followed at 33 centers worldwide, 5 years after a specific ECG pattern of right bundle-branch block and ST-segment elevation in leads V1 through V3 associated with sudden death in patients without demonstrable structural heart disease was described.<br>Size: 63 patients	Inclusion criteria: Patients with a specific ECG pattern of right bundle-branch block and ST-segment elevation in leads V1 through V3 associated with sudden death in patients without demonstrable structural heart disease	During a mean follow-up of 34 ± 32 months, an AE occurred in 14 symptomatic patients (34%) and 6 asymptomatic patients (27%). An automatic defibrillator was implanted in 35 patients, 15 received pharmacological therapy with beta-blockers and/or amiodarone, and 13 did not receive treatment The incidence of AEs was similar in all therapy groups (log-rank 0.86); however, total mortality was 0% in the implantable defibrillator group, 26% in the pharmacological group, and 31% in the no therapy group (log-rank 0.0005).	All mortality was due to sudden death.	Patients without demonstrable structural heart disease and an ECG pattern of right bundle-branch block and ST-segment elevation in leads V1 through V3 are at risk for sudden death. Amiodarone and/or beta-blockers do not protect them against sudden death, and an implantable defibrillator seems to be the present treatment of choice.
Haissaguerre et al. Sudden cardiac arrest associated with early repolarization.<br>• Year published: 2008<br>• PMID: 18463377	Aim: Early repolarization is a common electrocardiographic finding that is generally considered to be benign. Its potential to cause cardiac arrhythmias has been hypothesized from experimental studies, but it is not known whether there is a clinical association with SCA.<br>Size: 206 case subjects	Inclusion criteria: Data from 206 case subjects at 22 centers who were resuscitated after cardiac arrest due to IVF. The control group comprised 412 subjects without heart disease who were matched for age, sex, race, and level of physical activity.	Early repolarization was more frequent in case subjects with IVF than in control subjects (31% vs. 5%, $p < .001$). Among case subjects, those with early repolarization were more likely to be male and to have a history of syncope or SCA during sleep than those without early repolarization. In eight subjects, the origin of ectopy that initiated ventricular arrhythmias was mapped to sites concordant with the localization of repolarization abnormalities.	During a mean (SD) follow-up of 61 (50) months, defibrillator monitoring showed a higher incidence of recurrent VF in case subjects with a repolarization abnormality than in those without such an abnormality (hazard ratio, 2.1; 95% confidence interval, 1.2 to 3.5; $p = .008$).	Among patients with a history of IVF, there is an increased prevalence of early repolarization.

Study name or acronym; Author; Year published; PMID	Aim of study; Endpoints; Study type (RCT, observational — multicenter or single center; case series or other [if RCT include intervention and comparator]); Study size (N)	Patient population with inclusion and exclusion criteria	Results (absolute event rates, *p* values; OR or RR; 95% CI)	Other relevant findings or adverse events	Limitations; Other comments; Conclusions
Conte et al. Out-of-hospital cardiac arrest due to IVF in patients with normal electrocardiograms: results from a multicentre long-term registry.<br>• Year published: 2019<br>• PMID: 31504477	Aim: To define the clinical characteristics and long-term clinical outcomes of a large cohort of patients with IVF and normal 12-lead ECGs.<br>Size: 245 patients	Inclusion criteria: Patients with VF as the presenting rhythm, normal baseline, and follow-up ECGs with no signs of cardiac channelopathy including early repolarization or AV conduction abnormalities, and without structural heart disease.	Over a median follow-up of 63 months (IQR 25-110 months), 12 patients died (5%); in 4 of them (1.6%) the lethal event was of cardiac origin. Patients treated with antiarrhythmic drugs only had a higher rate of cardiovascular death compared to patients who received an ICD (16% vs. 0.4%, *p* = .001). Fifty-two patients (21%) experienced an arrhythmic recurrence.	Age ≤16 years at the time of the first ventricular arrhythmia was the only predictor of arrhythmic recurrence on multivariable analysis (HR 0.41, 95% CI 0.18-0.92; *p* = .03).	Patients with IVF and persistently normal ECGs frequently have arrhythmic recurrences, but a good prognosis when treated with an ICD. Children are a category of IVF patients at higher risk of arrhythmic recurrences.
Haukilahti et al. Sudden cardiac death in women.<br>• Year published: 2019<br>• PMID: 30779638	Aim: To determine the autopsy findings and causes of death among women in a large SCD population and to classify prior ECG characteristics in male and female subjects with SCD.<br>Size: 1,101 subjects	Inclusion criteria: The Fingesture study systematically collected clinical and autopsy data from subjects with SCD in Northern Finland between 1998 and 2017. The cohort consists of 5,869 subjects with SCD. Previously recorded ECGs were available and analyzed in 1,101 subjects (18.8% of total population; and in 25.3% of women).	Female subjects with SCD were significantly older than men: 70.1 ± 13.1 years versus 63.5 ± 11.8 years (mean ± SD, *p* < .001). The most frequently identified cause of death was ischemic heart disease in both sexes: 71.7% among women versus 75.7% among men, *p* = .005. In contrast, women were more likely to have nonischemic cause of SCD than men (28.3% versus 24.3%, *p* = .005). The prevalence of primary myocardial fibrosis was higher among women (5.2%, n = 64) than in men (2.6%, n = 120; *p* < .001). Female subjects with SCD were more likely to have normal prior ECG tracings (22.2% versus 15.3% in men, *p* < .001).	A normal ECG was even more common among nonischemic female subjects with SCD (27.8% versus 16.2% in men, *p* = .009). However, ECG markers of LVH, with or without repolarization abnormalities, were more common among women (8.2%; 17.9%) than in men (4.9%; 10.6%, *p* = .036; *p* < .001, respectively).	Women were considerably older at the time of SCD and more commonly had nonischemic causes. Women were also more likely to have a prior normal ECG than men, but an increased marker for SCD risk based on ECG criteria for LVH with repolarization abnormalities was more commonly observed in women.
Abdalla et al. Relation between ventricular	Aim: To evaluate the association between VPCs detected on a rest 2-min	Inclusion criteria: Apparently healthy white men, aged 35 to	The prevalence of any VPC was 4.4% (681 of 15,637). Over an average follow-up period of 7.5 years, a total	The presence of any VPC was not associated with any	When VPC characteristics such as frequency (2 or more uniform VPCs every 2 min) and

**Study name or acronym; Author; Year published; PMID**	**Aim of study; Endpoints; Study type (RCT, observational — multicenter or single center; case series or other [if RCT include intervention and comparator]); Study size (N)**	**Patient population with inclusion and exclusion criteria**	**Results (absolute event rates, *p* values; OR or RR; 95% CI)**	**Other relevant findings or adverse events**	**Limitations; Other comments; Conclusions**
premature complexes and sudden cardiac death in apparently healthy men.<br>• Year published: 1987<br>• PMID: 3673904	lead I electrocardiographic rhythm strip and SCD, occurring within 1 h of onset of symptoms.<br>Size: 15,637	57 years, at the first screening examination (1973 to 1975) to determine eligibility for the Multiple Risk Factor Intervention Trial in Minneapolis/St. Paul, MN.	of 381 deaths occurred. Of these, 34% (131 of 381) were ascribed to CAD and 31% of the CAD deaths (41 of 131) occurred suddenly. The presence of any VPC was associated with a significantly higher risk for SCD (adjusted relative risk = 3.0; *p* < .025).	significant increase in the risk of non-SCD or of total deaths from CAD (adjusted relative risk = 1.0 and 1.6, respectively).	complexity (multiforms, pairs, runs, R-on-T) were examined, those with frequent or complex VPCs were at a significantly increased risk of SCD (adjusted relative risk = 4.2; *p* < .005), whereas for non-SCD no significant increase in risk was found (adjusted relative risk = 1.6; *p* = .28).
Ataklte et al. Meta-analysis of ventricular premature complexes and their relation to cardiac mortality in general populations.<br>• Year published: 2013<br>• PMID: 23927786	Aim: To assess the association between VPCs and risk of SCD or total cardiac death in general populations.<br>Study type: Meta-analysis<br>Size: 11 studies comprising a total of 106,195 participants	Inclusion criteria: The electronic databases MEDLINE and Embase were searched for relevant studies. Data were abstracted using standardized forms. Study-specific relative risk estimates were pooled using a random-effects meta-analysis model. Eleven studies comprising a total of 106,195 participants sampled from general populations were included.	Studies generally defined frequent VPCs as occurring ≥1 time during a standard electrocardiographic recording or ≥30 times over a 1-h recording. The prevalence of frequent VPCs in the studies ranged from 1.2% to 10.7%. The overall adjusted relative risk for SCD comparing participants with frequent VPCs versus those without frequent VPCs was 2.64 (95% confidence interval 1.93 to 3.63). The corresponding value for total cardiac death was 2.07 (95% CI 1.71 to 2.50).	Although most studies made attempts to exclude high-risk subjects, such as those with histories of cardiovascular disease, they did not test participants for underlying structural heart disease.	Frequent VPCs are associated with a substantial increase in the risk for SCD and total cardiac death. Further study is needed to determine the role of confounding and underlying structural heart disease in the observed association and its utility in cardiovascular risk prediction.
Noda et al. Malignant entity of IVF and polymorphic ventricular tachycardia initiated by	Aim: To assess the clinical characteristics and the efficacy of RFCA for IVF (VF) and/or polymorphic VT initiated by ventricular extrasystoles originating from the RVOT.	Inclusion criteria: Patients with spontaneous VF and/or polymorphic VT initiated by the ventricular extrasystoles	Among 16 patients, spontaneous episodes of VF were documented in 5 patients, and 11 patients had prior episodes of syncope. Holter recordings showed frequent isolated ventricular extrasystoles with the same morphology as that of initiating	RFCA by targeting the initiating ventricular extrasystoles eliminated episodes of syncope, VF, and cardiac arrest in all	Malignant entity of idiopathic VF and/or polymorphic VT was occasionally present in patients with idiopathic ventricular arrhythmias arising from the RVOT. RFCA was effective as a treatment option for this entity.

Study name or acronym; Author; Year published; PMID	Aim of study; Endpoints; Study type (RCT, observational — multicenter or single center; case series or other [if RCT include intervention and comparator]); Study size (N)	Patient population with inclusion and exclusion criteria	Results (absolute event rates, *p* values; OR or RR; 95% CI)	Other relevant findings or adverse events	Limitations; Other comments; Conclusions
premature extrasystoles originating from the right ventricular outflow tract.<br>• Year published: 2005<br>• PMID: 16198845	Size: 16 patients	originating from the RVOT	ventricular extrasystoles, and nonsustained polymorphic VT with short cycle length (mean of 245 ± 28 ms) in all 16 patients.	patients during follow-up periods of 54 ± 39 months.	
Aizawa et al. Dynamicity of the J-wave in IVF with a special reference to pause-dependent augmentation of the J-wave.<br>• Year published: 2012<br>• PMID: 22624834	Aim: To evaluate the pause-dependency of the J-wave to characterize this phenomenon in idiopathic VF.<br>Size: 40 patients	Inclusion criteria: Patients with J-wave-associated idiopathic VF. J waves were defined as those ≥0.1 mV above the isoelectric line and were compared with 76 non-VF patients of comparable age and sex.	The J-wave was larger in patients with idiopathic VF than in the controls: 0.360 ± 0.181 mV versus 0.192 ± 0.064 mV (p = .0011). J waves were augmented during storms of VF (n = 9 [22.5%]), which was controlled by isoproterenol; they disappeared within weeks in 5 patients. In addition, sudden prolongation of the R-R interval was observed in 27 patients induced by benign arrhythmia, and 15 patients (55.6%) demonstrated pause-dependent augmentation (from 0.391 ± 0.126 mV to 0.549 ± 0.220 mV; p < .0001).	In the other 12 experimental subjects and in the 76 control subjects, J waves remained unchanged. Pause-dependent augmentation of J waves was detected in 55.6% (sensitivity) but was specific (100%) in the patients with idiopathic VF with high positive (100%) and negative (86.4%) predictive values.	Pause-dependent augmentation of J waves was confirmed in about one-half of the patients with idiopathic VF after sudden R-R prolongation. Such dynamicity of J waves was specific to idiopathic VF and may be used for risk stratification.
Tsuda et al. Significance of automated external defibrillator in identifying lethal ventricular arrhythmias.	Aim: To conduct retrospective cohort study of patients with aborted sudden cardiac death; AED is critical in saving children who develop unexpected cardiac arrest (CA), but its	25 patients (14 males) aged 1.3 to 17.5 years who presented with CA survived with prompt CPR. 18 patients had no prior cardiac diagnosis. CA occurred in 10 patients with more	Thorough investigations revealed 6 ion channelopathies (4 catecholaminergic polymorphic VT, 1 LQTS, and 1 BrS), 5 congenital heart disease (including 2 with coronary artery obstruction), 6 cardiomyopathies, 2 myocarditis, and 2 miscellaneous. 4 patients had no identifiable heart disease. In 5	AED reliably identifies the underlying lethal ventricular arrhythmias in addition to aborting SCD.	AED is both therapeutic in aborting SCD and diagnostic in identifying the underlying lethal ventricular arrhythmias. However, the diagnostic aspect of AED is under-acknowledged by most medical providers.

Study name or acronym; Author; Year published; PMID	Aim of study; Endpoints; Study type (RCT, observational — multicenter or single center; case series or other [if RCT include intervention and comparator]); Study size (N)	Patient population with inclusion and exclusion criteria	Results (absolute event rates, *p* values; OR or RR; 95% CI)	Other relevant findings or adverse events	Limitations; Other comments; Conclusions
• Year published: 2019<br>• PMID: 31297625	diagnostic capacity is not fully acknowledged.	than moderate exercise, in 7 with light exercise, and in 8 at rest (including 1 during sleep). 22 patients were resuscitated with AED, all of which were recognized as a shockable cardiac rhythm.	patients, the downloaded AED-recorded rhythm strip delineated the underlying arrhythmias and their responses to electrical shocks. 4 patients who presented with generalized seizure at rest were initially managed for seizure disorder until AED recording identified lethal ventricular arrhythmias.		
Bayes de Luna et al. Ambulatory sudden cardiac death: mechanisms of production of fatal arrhythmia on the basis of data from 157 cases.<br>• Year published: 1989<br>• PMID: 2911968	Aim: The study of the tapes of ambulatory patients who died while wearing Holter devices allows us to know the terminal electrical events of death in these cases and which are the electrical triggering mechanisms leading to the terminal event.<br>Size: 7 published series with 10 or more cases	Inclusion criteria: Ambulatory patients who died while wearing Holter devices	The most frequent causes of sudden death are VTAs (84% of cases) and bradyarrhythmias (16%). VF was the most frequent VTA, usually secondary to VT. The rest were due to torsades de pointes in patients often without heart disease but who were taking antiarrhythmic drugs.	The VT leading to VF was often preceded by sinus tachycardia or new atrial tachyarrhythmia. Only a small percentage of patients presented ischemic ST changes.	In patients who died due to bradyarrhythmias, this was more often due to sinus depression than to AV block.
Raju et al. Low prevalence of risk markers in cases of sudden death due to Brugada syndrome relevance to risk stratification in Brugada syndrome.<br>• Year published:	Aim: To determine the prevalence of conventional risk factors in sudden arrhythmic death syndrome (SADS) probands with BrS.<br>Size: 49 families, 202 family members in total (50 probands)	Inclusion criteria: A total of 49 consecutive families with a confirmed SADS death and a diagnosis of BrS were evaluated, comprising assessment of 202 family members in total. One family had 2 members with SADS, resulting in a total of 50 probands included.	Mean age of death of probands was 29.1 ± 10.6 years, with 41 males (82%) (*p* < .05). Antemortem ECGs were available for 5 SADS probands, 1 of which demonstrated a spontaneous type 1 pattern. In 45 probands, symptoms before death were reported reliably by family members. Of these, 9 (20%) had experienced at least 1 syncopal episode before the fatal event. 68% of probands would		The "low-risk" asymptomatic BrS group comprises the majority of SCD in this cohort. Current risk stratification would appear to be inadequate, and new markers of risk are vital.

Study name or acronym; Author; Year published; PMID	Aim of study; Endpoints; Study type (RCT, observational — multicenter or single center; case series or other [if RCT include intervention and comparator]); Study size (N)	Patient population with inclusion and exclusion criteria	Results (absolute event rates, *p* values; OR or RR; 95% CI)	Other relevant findings or adverse events	Limitations; Other comments; Conclusions
2011<br>• PMID: 21636035			not have fulfilled any current criteria for consideration of ICD.		
Gladding et al. Posthumous diagnosis of long QT syndrome from neonatal screening cards.<br>• Year published: 2010<br>• PMID: 20167303	Aim: To assess the feasibility and clinical value of posthumous genetic testing for LQTS using residual material from the neonatal screening (Guthrie) card in SUDY victims.<br>Size: 21 cases	Inclusion criteria: Twenty-one cases were investigated up to 13 years after death. Deaths occurred at <1 year in one, 1-18 years in 18, and 19-35 years in two patients. Guthrie cards were 3-39 years old.	Adequate DNA was extracted in every case, although repeated purification and amplification was often required. Rare variants were detected in 6 of 19 cases undergoing diagnostic screening. Four (21%) are considered to be pathological and have been used for family screening: R243C and H455Y in KCNQ1 in 12-year-old and 13-year-old boys, respectively, and Q81H and S621R in KCNH2 in 21-month and 28-year-old females, respectively. VUS were R1047L in KCNH2 in a 2-year-old girl and S38G in KCNE1 in a 19-month-old boy.	Point mutation tests for previously identified familial LQTS mutations revealed a positive result in both cases: E146K in KCNQ1 and exon 6-4del in KCNH2.	Residual material from Guthrie cards collected for newborn metabolic screening can be used as a reliable source of DNA for the posthumous diagnosis of LQTS decades after SUDY, although purification and amplification of DNA is time intensive.
Carturan et al. Postmortem genetic testing for conventional autopsy-negative sudden unexplained death: an evaluation of different DNA extraction protocols and the feasibility of mutational analysis from archival paraffin-	Aim: To evaluate the feasibility of using PET DNA for genetic testing.<br>Size: 35 SUD cases	Inclusion criteria: Different DNA extraction procedures, involving 2 deparaffinization methods, 2 digestion methods, 4 laboratory-based purification methods, and 5 commercial kits were examined. Mutational analysis involving 25 RYR2 exons was performed on PET DNA from 35 SUD.	With the best PET-DNA extraction method, an average of only two-thirds of the region of interest could be evaluated.	Although we initially identified 5 missense mutations in 5 of 35 SUD cases, repeated analysis failed to confirm these mutations.	DNA from PET should be considered error prone and unreliable in comprehensive surveillance of SUD-associated genes. Given these shortcomings, the standard autopsy for SUD should include archiving EDTA-preserved blood or frozen tissue to facilitate postmortem genetic testing.

**Study name or acronym; Author; Year published; PMID**	**Aim of study; Endpoints; Study type (RCT, observational — multicenter or single center; case series or other [if RCT include intervention and comparator]); Study size (N)**	**Patient population with inclusion and exclusion criteria**	**Results (absolute event rates, *p* values; OR or RR; 95% CI)**	**Other relevant findings or adverse events**	**Limitations; Other comments; Conclusions**
embedded heart tissue.<br>• Year published: 2008<br>• PMID: 18285261					
Searles Nielsen et al. Newborn screening archives as a specimen source for epidemiologic studies: feasibility and potential for bias.<br>• Year published: 2008<br>• PMID: 18063239	Aim: To evaluate the feasibility of obtaining DBS from newborn screening archives for subjects in epidemiologic studies and using these specimens for genotyping, and to evaluate the potential for bias in their use.<br>Size: 230	Inclusion criteria: The study attempted to locate DBS at Washington State's archives for 230 participants in a previous case-control study of childhood cancer, who were born 1978-1990.	Specimens for 203 (88%) children were retrieved, including 199 (94%) born in months when a DBS catalog was available. Among the latter, the proportion with specimens located varied by birthplace (e.g., hospital, home), maternal education, and prenatal smoking, but did not vary significantly by race/ethnicity.	All genotyping assays were completed for all specimens, and among controls genotype distributions were in Hardy-Weinberg equilibrium and similar to previous reports.	Newborn screening archives have potential to provide specimens for epidemiologic studies conducting genotyping and perhaps other assays, but the possibility that reliance on these resources could bias risk estimates must be considered.
Mellor et al. Genetic testing in the evaluation of unexplained cardiac arrest: from the CASPER (Cardiac Arrest Survivors with Preserved Ejection Fraction Registry).<br>• Year published: 2017<br>• PMID: 28600387	Aim: To assess role of genetic testing in individuals without a clinical phenotype.<br>Endpoints: Mutation carrier<br>Study type: Cross-sectional<br>Size: 174 genetic testing/ 375 cardiac arrest survivors in CASPER; genetic testing of cardiac genes at the discretion of the treating provider	Inclusion criteria: UCA with documented VT or VF requiring direct current cardioversion or defibrillation, and subsequent testing demonstrated normal left ventricular function (left ventricular ejection fraction ≥50%) and normal coronary arteries (no coronary stenosis >50%)<br>Exclusion criteria: Known causes for cardiac arrest, including an ECG diagnostic of LQTS (persistent resting QTc	29 individuals with a pathogenic variant (17% yield); pathogenic variants identified in 18/72 (25%) of phenotype positive patients; pathogenic variants identified in 11/102 (11%) of phenotype negative patients	Mean age 39 years; 18% with ≥VUS; prior syncope (OR 4) and fam hx of SCA (OR 3.2) were associated with pathogenic variant carrier status	Nonstandardized genetic testing panels; no controls; no segregation data.<br>Prior syncope and family history of sudden death are predictors of a positive genetic test. Both arrhythmia and cardiomyopathy genes are implicated. Broad, multiphenotype testing revealed the highest frequency of pathogenic variants in phenotype-negative patients.

**Study name or acronym; Author; Year published; PMID**	**Aim of study; Endpoints; Study type (RCT, observational — multicenter or single center; case series or other [if RCT include intervention and comparator]); Study size (N)**	**Patient population with inclusion and exclusion criteria**	**Results (absolute event rates, *p* values; OR or RR; 95% CI)**	**Other relevant findings or adverse events**	**Limitations; Other comments; Conclusions**
		>460 ms for males and 480 ms for females) or BrS, HCM, marked hypokalemia, drug overdose, or commotio cordis, recognized forms of idiopathic VT			
Manolis et al. Sudden unexpected death in epilepsy: the neuro-cardio-respiratory connection.<br>• Year published: 2019<br>• PMID: 30566897	Aim: SUDEP is the major cause of epilepsy-related premature mortality which targets preferentially younger people. Its etiology remains unknown.	Neuroimaging and molecular genetic studies may provide insights into the causes of SUDEP and identify potential biomarkers for risk stratification of patients susceptible to SUDEP. These issues are reviewed with emphasis placed on the neuro-cardio-respiratory functions affected by epilepsy and their genetic control and influences.	Several risk factors have been identified with generalized tonic-clonic seizures as the most important one; seizure control remains the most effective measure of prevention. Although some cases may be attributable to cardiac causes, mainly undiagnosed cardiac channelopathies, the majority appear linked to epilepsy-related disruption of the functional connectivity of certain brain structures associated with the central autonomic control of cardio-respiratory function (neuro-cardio-respiratory connection).		Obtaining further data on its pathophysiologic mechanisms is a cardinal step toward preventing and reducing the incidence of SUDEP.
MacCormick et al. Misdiagnosis of long QT syndrome as epilepsy at first presentation.<br>• Year published: 2009<br>• PMID: 19282063	Aim: To evaluate a series of patients with genetically confirmed LQTS to establish the frequency of delayed recognition, and to examine causes and potential consequences of diagnostic delay.<br>Study type: Retrospective review<br>Size: 31 cases	Inclusion criteria: A consecutive case series of patients with LQTS was identified through the Cardiac Inherited Disease Registry in New Zealand between 2000 and 2005.	Genetic mutations in 31 probands were consistent with long QT type 1 in 18 (58%) patients, long QT type 2 in 10 (32%) and long QT type 3 in 3 (10%). Median age at diagnosis was 21 years (1 day to 54 years). Thirteen patients (39%) experienced diagnostic delay after presentation with syncope or seizure: median delay 2.4 years (2 months to 23 years). Electroencephalograms were obtained	For those labeled epileptic, diagnostic delay was significantly longer than with other misdiagnoses: estimated median difference 9.75 years (95% confidence interval 7.6 to 20.7 years). During the delay	Delayed diagnosis of LQTS is frequent. Symptoms are often attributed to alternative diagnoses, most commonly seizure disorder. Given the potentially preventable mortality of LQTS, emergency physicians investigating syncope and seizure should maintain a high index of suspicion.

**Study name or acronym; Author; Year published; PMID**	**Aim of study; Endpoints; Study type (RCT, observational — multicenter or single center; case series or other [if RCT include intervention and comparator]); Study size (N)**	**Patient population with inclusion and exclusion criteria**	**Results (absolute event rates, *p* values; OR or RR; 95% CI)**	**Other relevant findings or adverse events**	**Limitations; Other comments; Conclusions**
			in 10 patients; 5 were diagnosed with epilepsy.	period, 4 SUDs reportedly occurred in young relatives.	
Johnson et al. Identification of a possible pathogenic link between congenital long QT syndrome and epilepsy.<br>• Year published: 2009<br>• PMID: 19038855	Aim: To test the hypothesis that a "seizure phenotype" was ascribed more commonly to patients with LQT2.<br>Size: 343 patients	Charts were reviewed for 343 consecutive, unrelated patients (232 females, average age at diagnosis 27 ± 18 years, QTc 471 ± 57 ms) clinically evaluated and genetically tested for LQTS from 1998 to 2006 at two large LQTS referral centers.	A seizure phenotype was recorded in 98/343 (29%) probands. A seizure phenotype was more common in LQT2 (36/77, 47%) than LQT1 (16/72, 22%, $p < .002$) and LQT3 (7/28, 25%, $p < .05$, NS). LQT1 and LQT3 combined cohorts did not differ significantly from expected, background rates of a seizure phenotype. A personal history of seizures was more common in LQT2 (30/77, 39%) than all other subtypes of LQTS (11/106, 10%, $p < .001$).	A diagnostic consideration of epilepsy and treatment with antiepileptic drug medications was more common in patients with LQT2.	Like noncardiac organ phenotypes observed in other LQTS-susceptibility genes such as KCNQ1/deafness and SCN5A/gastrointestinal symptoms, this novel LQT2-epilepsy association raises the possibility that LQT2-causing perturbations in the KCNH2-encoded potassium channel may confer susceptibility for recurrent seizure activity.
Johnson et al. Cardiac channel molecular autopsy for sudden unexpected death in epilepsy.<br>• Year published: 2010<br>• PMID: 20395638	SUDEP is the sudden, unexplained, unexpected death of an individual with epilepsy in which postmortem examination does not reveal an anatomic or toxicologic cause of death. Patients with congenital LQTS and CPVT have been frequently initially diagnosed with epilepsy.<br>Study type: Case report<br>Size: 1 patient	A cardiac channel molecular autopsy of the common LQTS and CPVT-susceptibility genes was performed on an archived necropsy specimen from an 8-year-old victim of SUDEP.	A novel, sporadic missense mutation in exon 104 of the RYR2-encoded ryanodine receptor/calcium release channel (c. 14806G>A, p.Gly4936Arg) was discovered. This mutation was absent in >600 reference alleles including both parents, involved a highly conserved amino acid, and localized to a key structure-function domain.		First postmortem molecular diagnosis of CPVT in a patient with SUDEP.

**Study name or acronym; Author; Year published; PMID**	**Aim of study; Endpoints; Study type (RCT, observational — multicenter or single center; case series or other [if RCT include intervention and comparator]); Study size (N)**	**Patient population with inclusion and exclusion criteria**	**Results (absolute event rates, *p* values; OR or RR; 95% CI)**	**Other relevant findings or adverse events**	**Limitations; Other comments; Conclusions**
Lahrouchi et al. Utility of post-mortem genetic testing in cases of sudden arrhythmic death syndrome.<br>• Year published: 2017<br>• PMID: 28449774	Aim: To define use of postmortem genetic testing following SCD in negative autopsy SCD.<br>Endpoints: Genetic diagnosis in proband and family<br>Study type: Multicenter, retrospective case-control study<br>Size: 302 SCD cases	Inclusion criteria:<br>SCD cases with available DNA and negative autopsy; negative autopsy<br>Exclusion: Structural heart disease or nonspecific pathological features at autopsy	-Death with exercise (10%) or extreme emotion (1.5%)<br>-Family history in 7.1%<br>-18% personal history of syncope or seizures<br>-21 (7%) diagnosed with epilepsy or prior history of epilepsy<br>-24 had consulted health care provider	-19 pathogenic and 15 likely pathogenic variants in RyR2, KCNH2, KCNQ1, SCN5A<br>-1 pathogenic variant in PLN, likely pathogenic versions in TTN (2), PKP2 (1), MYH7 (1)	-88% European heritage<br>-Limited family evaluation<br>-Referral bias
Skinner et al. Use of the newborn screening card to define cause of death in a 12-year-old diagnosed with epilepsy.<br>• Year published: 2004<br>• PMID: 15469540	Aim: To report a postmortem molecular diagnosis of LQTS in a 12-year-old boy diagnosed with epilepsy who died suddenly playing sport.<br>Study type: Case report<br>Size: 1 patient	The DNA was extracted from an archived blood spot on his newborn screening (“Guthrie”) card, which had been taken from him at 6 days of age.	A missense mutation was detected in exon 5 of the KCNQ1 gene; R243C (835C > T), associated with long QT type 1.	The same mutation was found in the mother (who now takes effective preventative therapy), but not in the sibling who was reassured that she is not at risk of sudden death.	
Chahal et al. Systematic review of the genetics of sudden unexpected death in epilepsy: potential overlap with sudden cardiac death and arrhythmia-related genes.<br>• Year published: 2020	Aim: To systematically review the literature on the genetics of SUDEP.<br>Study type: Review<br>Size: 8 studies with 161 unique individuals	Inclusion criteria:<br>English language human studies analyzing SUDEP for known sudden death, ion channel and arrhythmia-related pathogenic variants, novel variant discovery, and copy number variant analyses	Mean was age 29.0 (SD 14.2) years; 61% males; ECG data were reported in 7.5% of cases; 50.7% were found prone and 58% of deaths were nocturnal. Cause included all types of epilepsy. Antemortem diagnosis of Dravet syndrome and autism (with duplication of chromosome 15) was associated with 11% and 9% of cases. The most frequently detected known pathogenic variants at postmortem were in Na+ and K+ ion channel	Overall, the majority of variants were of unknown significance. Analysis of copy number variant was insignificant.	SUDEP case adjudication and evaluation remains limited largely because of crucial missing data such as ECGs. The most frequent pathogenic/likely pathogenic variants identified by molecular autopsy are in ion channel or arrhythmia-related genes, with an ≈11% discovery rate. Comprehensive postmortem examination should include examination of the heart and brain by

Study name or acronym; Author; Year published; PMID	Aim of study; Endpoints; Study type (RCT, observational — multicenter or single center; case series or other [if RCT include intervention and comparator]); Study size (N)	Patient population with inclusion and exclusion criteria	Results (absolute event rates, *p* values; OR or RR; 95% CI)	Other relevant findings or adverse events	Limitations; Other comments; Conclusions
• PMID: 31865891			subunits, as were novel potentially pathogenic variants (11%).		specialized pathologists and blood storage.
Lacour et al. Cardiac implantable electronic device interrogation at forensic autopsy: an underestimated resource?<br>• Year published: 2018<br>• PMID: 29915100	Aim: To determine whether nonselective postmortem CIED interrogations and data analysis are useful to the forensic pathologist to determine the cause, mechanism, and time of death and to detect potential CIED-related safety issues.<br>Size: 150	Inclusion criteria: All autopsy subjects From February 2012 to April 2017 in the department of forensic medicine at the University Hospital Charité who had a CIED	In 40 cases (26.7%) time of death and in 51 cases (34.0%) cause of death could not be determined by forensic autopsy. Of these, CIED interrogation facilitated the determination of time of death in 70.0% of the cases and clarified the cause of death in 60.8%. Device concerns were identified in 9 cases (6.0%), including 3 hardware, 4 programming, and 2 algorithm issues. One CIED was submitted to the manufacturer for a detailed technical analysis.	Necessity of systematic postmortem CIED interrogation in forensic medicine to determine the cause and timing of death more accurately.	In addition, CIED analysis is an important tool to detect potential CIED-related safety issues.
Riesinger et al. Postmortem interrogation of cardiac implantable electrical devices may clarify time and cause of death.<br>• Year published: 2019<br>• PMID: 30238160	Aim: Postmortem interrogation of CIEDs in autopsy is not routinely performed. Thus, it remains unclear whether an interrogation might clarify time and cause of death.<br>Size: 70 patients	Inclusion criteria: Seventy of 4401 patients (1.6%) undergoing autopsy in 2014 and 2015 presented with a CIED. The explanted CIED were interrogated with respect to time and possible cause of death.	Twenty-five ICDs and 45 PM devices were analyzed. Death was classified as cardiac by autopsy in 17 of 70 patients. Presumably lethal ventricular arrhythmias were documented in 6 patients (8.6%; 5 ICD, 1 PM). In 2 of 30 patients with unknown cause of death after autopsy (6.7%), interrogation revealed VT as potential reason for decease (1 ICD, 1 PM). Postmortem CIED interrogation additionally allowed to make a statement regarding the day of death in 36 patients (51%; 13 ICD, 23 PM).	Interrogation of CIED revealed potentially lethal ventricular arrhythmias in 9 of 70 patients investigated and enabled valid estimation of the day of death in 15 patients.	Routinely performed postmortem CIED interrogation may clarify time and cause of death.
Sinha et al. Clinical inferences of cardiovascular implantable electronic device	Aim: To determine the utility of systematic routine CIED removal, interrogation, and analysis at autopsy.	Inclusion criteria: A total of 2,025 autopsies were performed; 84 subjects had CIEDs removed and analyzed.	Overall, 43 subjects had died suddenly, and 41 had not died suddenly. Significant clinical alerts (sustained tachyarrhythmias or an elevated fluid index value) were seen	Manufacturer analyses revealed a case of premature PM battery depletion, as well as	Postmortem CIED analysis was clinically useful in assisting with determination of the timing, mechanism, and cause of death in the majority of sudden deaths

Study name or acronym; Author; Year published; PMID	Aim of study; Endpoints; Study type (RCT, observational — multicenter or single center; case series or other [if RCT include intervention and comparator]); Study size (N)	Patient population with inclusion and exclusion criteria	Results (absolute event rates, *p* values; OR or RR; 95% CI)	Other relevant findings or adverse events	Limitations; Other comments; Conclusions
analysis at autopsy.<br>• Year published: 2016<br>• PMID: 27634115	Study type: Institutional registry analysis<br>Size: 84	These devices included 37 PMs and 47 defibrillators.	in 62.8% cases of sudden deaths. In the nonsudden death cohort, 19.5% displayed a significant clinical alert. Significant association of CIED alerts were noted when comparing sudden deaths versus nonsudden deaths ($p <$ .001), defibrillators versus PMs ($p <$ .005), and cardiac versus noncardiac causes of death ($p <$ .001).	a hard reset in a defibrillator as a result of cold exposure.	and in almost 20% of nonsudden deaths. CIED removal with analysis as an important diagnostic tool in all autopsies and to assist manufacturers in identifying potentially fatal device failures is advocated.
**Investigation of Sudden Death: The Postmortem Examination and Imaging**					
Gulino et al. Improving forensic pathologic investigation of sudden death in the young: tools, guidance, and methods of cardiovascular dissection from the sudden death in the young case registry.<br>• Year published: 2018<br>• PMID: 31240048	Aim: To describe the recommended practice by the Sudden Death in the Young Case Registry.	The SDY Case Registry autopsy guidance tools focus on medical conditions and other anatomic findings that may be associated with SDY. They include a recommended comprehensive cardiovascular examination due to the high incidence of cardiac causes of SDY in children over age one. The tools are in checklist format to enable rapid and easy use by pathologists.	To facilitate improved understanding of SDY and ultimately inform prevention efforts, the Sudden Death in the Young Case Registry created tools and guidance to encourage standardized, comprehensive autopsies and death scene investigations for SDY cases.	The SDY Case Registry tools and guidance documents promote standardization of death investigation and autopsy practices in funded jurisdictions.	After informed consent from the next of kin, the Sudden Death in the Young Case Registry permits collection of comprehensive information from investigation and autopsy as well as samples for DNA extraction that may inform the cause of death determination, guide cascade screening for families, and create a resource for research.
Risgaard et al. Risk factors and causes of sudden noncardiac death: a nationwide	Aim: To report the risk factors and causes of SNCD.<br>Study type: Retrospective<br>Size: 286	Inclusion criteria: SNDs between 2000 and 2006 of individuals aged 1-35 years and between 2007 and 2009 of individuals	The median age in the SNCD death population was 32 years. Increasing age was inversely associated with SNCD (OR 0.93, 95% CI 0.87-0.98). Female sex, in-hospital location, and the absence of cardiac comorbidities were positively	Sudden death among individuals aged <50 years was caused by noncardiac diseases in 28% of cases. Risk	Data may guide future strategies for the follow-up of family members of nonautopsied sudden death victims, improve risk

Study name or acronym; Author; Year published; PMID	Aim of study; Endpoints; Study type (RCT, observational — multicenter or single center; case series or other [if RCT include intervention and comparator]); Study size (N)	Patient population with inclusion and exclusion criteria	Results (absolute event rates, *p* values; OR or RR; 95% CI)	Other relevant findings or adverse events	Limitations; Other comments; Conclusions
cohort study in Denmark.<br>• Year published: 2015<br>• PMID: 25614248		aged 1-49 years; 1039 autopsied cases of sudden death were identified, of which 286 (28%) were classified as SNCD.	associated with SNCD (OR 1.7, 95% CI 1.3-2.3; OR 3.0, 95% CI 2.0-4.4; and OR 4.3, 95% CI 2.5-7.4, respectively). The most common cause of SNCD was pulmonary disease (n = 115 [40%]).	factors were female sex, age, and the absence of cardiac comorbidities.	stratification, and influence public health strategies.
de Noronha et al. The importance of specialist cardiac histopathological examination in the investigation of young sudden cardiac deaths.<br>• Year published: 2014<br>• PMID: 24148315	Aim: To demonstrate the improvement in diagnostic quality offered by a specialist cardiac pathology service established to investigate SCD with fast-track reporting on hearts sent by pathologists in cases of SCD.<br>Study type: Prospective observational study<br>Size: 720	Inclusion criteria: Cases of SCD referred by coroners and pathologists from 2007 to 2009	Most SCDs occurred in males (66%), with the median age being 32 years. The majority (57%) of deaths occurred at home. The main diagnoses were a morphologically normal heart (n = 321; 45%), cardiomyopathy (n = 207, 29%), and coronary artery pathology (n = 71; 10%). In 158 out of a sample of 200 consecutive cases, a cardiac examination was also performed by the referring pathologist with a disparity in diagnosis in 41% of the cases (κ = 0.48).	Referring pathologists were more inclined to diagnose cardiomyopathy than normality with only 50 out of 80 (63%) normal hearts being described correctly.	Expert cardiac pathology improves the accuracy of coronial postmortem diagnoses in young SCD. This is important as the majority of cases may be due to inherited cardiac diseases and the autopsy guides the appropriate cardiological evaluation of blood relatives for their risk of sudden death.
Finocchiaro et al. Etiology of sudden death in sports: insights from a United Kingdom regional registry.<br>• Year published: 2016<br>• PMID: 27151341	Aim: To investigate causes of SCD and their association with intensive physical activity in a large cohort of athletes.<br>Size: 357	Inclusion criteria: 357 consecutive cases of athletes who died suddenly (mean 29 ± 11 years of age, 92% males, 76% Caucasian, 69% competitive) referred to the investigators' cardiac pathology center between 1994 and 2014; all subjects underwent detailed postmortem evaluation.	SADS was the most prevalent cause of death (n = 149 [42%]). Myocardial disease was detected in 40% of cases, including idiopathic LVH and/or fibrosis (n = 59, 16%); ARVC (13%); and HCM (6%). Coronary artery anomalies occurred in 5% of cases. SADS and coronary artery anomalies affected predominantly young athletes (≤ 35 years of age), whereas myocardial disease was more common in older individuals.	SCD during intense exertion occurred in 61% of cases; ARVC and left ventricular fibrosis most strongly predicted SCD during exertion.	The strong association of ARVC and left ventricular fibrosis with exercise-induced SCD reinforces the need for early detection and abstinence from intense exercise. However, almost 40% of athletes die at rest, highlighting the need for complementary preventive strategies.

**Study name or acronym; Author; Year published; PMID**	**Aim of study; Endpoints; Study type (RCT, observational — multicenter or single center; case series or other [if RCT include intervention and comparator]); Study size (N)**	**Patient population with inclusion and exclusion criteria**	**Results (absolute event rates, *p* values; OR or RR; 95% CI)**	**Other relevant findings or adverse events**	**Limitations; Other comments; Conclusions**
Bagnall et al. A prospective study of sudden cardiac death among children and young adults.<br>• Year published: 2016<br>• PMID: 27332903	Aim: To define etiology of SCD in Australia and New Zealand 2010-2012.<br>Endpoints: Clinical/genetic diagnosis in proband and family<br>Study type: Prospective population study<br>Size: 490 SCD cases	Inclusion criteria:<br>All SCD cases aged 1-35 years	SCD incidence in Australia/New Zealand was 1.3 per 100,000<br>Unexplained SCD more common in females, when occurring at night, and children aged 1-5 years.<br>54/490 cases were exertional or post-exercise.	Unexplained death the most common etiology; statistically associated with female sex and nocturnal SCD	Not enough evidence to include all cases. The study did not include resuscitated cardiac arrest cases.
Behr et al. Sudden arrhythmic death syndrome: a national survey of sudden unexplained cardiac death.<br>• Year published: 2007<br>• PMID: 17237131	Aim: To describe the characteristics of SADS and compare its incidence with official national mortality statistics for unascertained deaths.<br>Size: 115	Inclusion criteria:<br>Consecutive cases meeting the following criteria: white Caucasian, aged 4-64 years, no history of cardiac disease, last seen alive within 12 h of death, normal coroner's autopsy, cardiac pathologist's confirmation of a normal heart and negative toxicology.	56 (49%) SADS victims were identified: mean age 32 years, range 7-64 years and 35 (63%) male. 7 of 39 cases (18%) had a family history of other premature sudden deaths (<45). The estimated mortality from SADS was 0.16/100,000 per annum (95% CI 0.12 to 0.21), compared with an official mortality of 0.10/100,000 per annum for International Classification of Diseases 798.1 (sudden death, cause unknown-instantaneous death) or 1.34/100,000 per annum for unascertained causes of death.	Deaths from SADS occur predominantly in young males.	When compared with official mortality, the incidence of SADS may be up to eight times higher than estimated: more than 500 potential SADS cases per annum in England. Families with SADS carry genetic cardiac disease, placing them at risk of further sudden deaths. SADS should therefore be a certifiable cause of death prompting specialized cardiological evaluation of families.
Bjune et al. Post-mortem toxicology in young sudden cardiac death victims: a nationwide cohort study.<br>• Year published: 2018<br>• PMID: 28339816	Aim: To investigate in detail the toxicological findings of all young SCD throughout Denmark.<br>Size: 620 medico-legal autopsied cases of SCD	Inclusion criteria:<br>Deaths in persons aged 1-49 years were included over a 10-year period. Death certificates and autopsy reports were retrieved and read to identify cases of sudden death and establish cause of death.	We identified 620 medico-legal autopsied cases of SCD, of which 77% (n = 477) were toxicologically investigated postmortem, and 57% (n = 270) had a positive toxicology profile. SCD with positive toxicology had higher rates of SADS, compared with SCD with negative toxicology (56% vs. 42%, $p < .01$). In total, 752 agents were detected, and polypharmacy (defined as the	Psychotropic drugs were the most frequent (62%, n = 467), and 82% (n = 385) were in pharmacological or subpharmacological levels.	More than half of all toxicologically investigated SCD victims had positive postmortem toxicological findings. SCD with positive toxicology had higher rate of SADS, suggesting that the compounds may play a proarrhythmic role in these cases.

Study name or acronym; Author; Year published; PMID	Aim of study; Endpoints; Study type (RCT, observational — multicenter or single center; case series or other [if RCT include intervention and comparator]); Study size (N)	Patient population with inclusion and exclusion criteria	Results (absolute event rates, *p* values; OR or RR; 95% CI)	Other relevant findings or adverse events	Limitations; Other comments; Conclusions
			presence of more than one drug) was present in 61% (n = 164), all substances combined.		
Lahrouchi et al. Utility of post-mortem genetic testing in cases of sudden arrhythmic death syndrome.<br>• Year published: 2017<br>• PMID: 28449774	Aim: To define use of postmortem genetic testing following SCD in negative autopsy SCD.<br>Endpoints: Genetic diagnosis in proband and family<br>Study type: Multicenter, retrospective case-control study<br>Size: 302 SCD cases	Inclusion criteria: SCD cases with available DNA and negative autopsy; negative autopsy<br>Exclusion: Structural heart disease or nonspecific pathological features at autopsy	-Death with exercise (10%) or extreme emotion (1.5%)<br>-Family history in 7.1%<br>-18% personal history of syncope or seizures<br>-21 (7%) diagnosed with epilepsy or prior history of epilepsy<br>-24 had consulted health care provider	-19 pathogenic and 15 likely pathogenic variants in RyR2, KCNH2, KCNQ1, SCN5A<br>-1 pathogenic variant in PLN, likely pathogenic versions in TTN (2), PKP2 (1), MYH7 (1)	-88% European heritage<br>-Limited family evaluation<br>-Referral bias
Tester et al. Cardiac channel molecular autopsy: insights from 173 consecutive cases of autopsy-negative sudden unexplained death referred for postmortem genetic testing.<br>• Year published: 2012<br>• PMID: 22677073	Aim: To perform LQTS and CPVT cardiac channel postmortem genetic testing (molecular autopsy) for a large cohort of cases of autopsy-negative SUD<br>Size: 173 cases of SUD	Inclusion criteria: From September 1, 1998, through October 31, 2010, 173 cases of SUD (106 males; mean ± SD age, 18.4 ± 12.9 years; age range, 1-69 years; 89% white) referred by medical examiners or coroners for a cardiac channel molecular autopsy.	Overall, 45 putative pathogenic mutations absent in 400 to 700 controls were identified in 45 autopsy-negative SUD cases (26.0%). Females had a higher yield (26/67 [38.8%]) than males (19/106 [17.9%]; $p < .005$). Among SUD cases with exercise-induced death, the yield trended higher among the 1- to 10-year-olds (8/12 [66.7%]) compared with the 11- to 20-year-olds (4/27 [14.8%]; $p = .002$). In contrast, for those who died during a period of sleep, the 11- to 20-year-olds had a higher yield (9/25 [36.0%]) than the 1- to 10-year-olds (1/24 [4.2%]; $p = .01$).	Cardiac channel molecular autopsy should be considered in the evaluation of autopsy-negative SUD.	Several interesting genotype-phenotype observations may provide insight into the expected yields of postmortem genetic testing for SUD and assist in selecting cases with the greatest potential for mutation discovery and directing genetic testing efforts.

Study name or acronym; Author; Year published; PMID	Aim of study; Endpoints; Study type (RCT, observational — multicenter or single center; case series or other [if RCT include intervention and comparator]); Study size (N)	Patient population with inclusion and exclusion criteria	Results (absolute event rates, *p* values; OR or RR; 95% CI)	Other relevant findings or adverse events	Limitations; Other comments; Conclusions
Carturan et al. Postmortem genetic testing for conventional autopsy-negative sudden unexplained death: an evaluation of different DNA extraction protocols and the feasibility of mutational analysis from archival paraffin-embedded heart tissue.<br>• Year published: 2008<br>• PMID: 18285261	Aim: To examine different DNA extraction procedures and to evaluate the feasibility of using PET DNA for genetic testing.<br>Size: 35 SUD cases	Different DNA extraction procedures were examined, involving 2 deparaffinization methods, 2 digestion methods, 4 laboratory-based purification methods, and 5 commercial kits. Mutational analysis involving 25 RYR2 exons was performed on PET DNA from 35 SUD cases to evaluate the feasibility of using PET DNA for genetic testing	With the best PET-DNA extraction method, an average of only two-thirds of the region of interest could be evaluated. Although 5 missense mutations were initially identified in 5 of 35 SUD cases, repeated analysis failed to confirm these mutations.	DNA from PET should be considered error prone and unreliable in comprehensive surveillance of SUD-associated genes.	Given these shortcomings, the standard autopsy for SUD should include archiving EDTA-preserved blood or frozen tissue to facilitate postmortem genetic testing.
Gollob et al. Somatic mutations in the connexin 40 gene (GJA5) in atrial fibrillation.<br>• Year published: 2006<br>• PMID: 16790700	Aim: To hypothesize that idiopathic atrial fibrillation has a genetic basis and that tissue-specific mutations in GJA5, the gene encoding connexin 40, may predispose the atria to fibrillation.<br>Size: 15 patients	Inclusion criteria: GJA5 from genomic DNA isolated from resected cardiac tissue and peripheral lymphocytes sequenced from 15 patients with idiopathic atrial fibrillation. Identified GJA5 mutations were transfected into a gap-junction-deficient cell line to assess their functional effects on	Four novel heterozygous missense mutations were identified in 4 of the 15 patients. In 3 patients, the mutations were found in the cardiac-tissue specimens but not in the lymphocytes, indicating a somatic source of the genetic defects. In the fourth patient, the sequence variant was detected in both cardiac tissue and lymphocytes, suggesting a germ-line origin.	Analysis of the expression of mutant proteins revealed impaired intracellular transport or reduced intercellular electrical coupling.	Mutations in GJA5 may predispose patients to idiopathic atrial fibrillation by impairing gap-junction assembly or electrical coupling. Data suggest that common diseases traditionally considered to be idiopathic may have a genetic basis, with mutations confined to the diseased tissue.

**Study name or acronym; Author; Year published; PMID**	**Aim of study; Endpoints; Study type (RCT, observational — multicenter or single center; case series or other [if RCT include intervention and comparator]); Study size (N)**	**Patient population with inclusion and exclusion criteria**	**Results (absolute event rates, *p* values; OR or RR; 95% CI)**	**Other relevant findings or adverse events**	**Limitations; Other comments; Conclusions**
		protein transport and intercellular electrical coupling.			
Leong et al. Splice site variants in the *KCNQ1* and *SCN5A* genes: transcript analysis as a tool in supporting pathogenicity.<br>• Year published: 2017<br>• PMID: 28725320	Aim: To report an analysis of the transcriptional consequences of two mutations, one in the KCNQ1 gene (c.781_782delinsTC) and one in the SCN5A gene (c.2437-5C>A), which are predicted to affect splicing.	RNA was isolated from lymphocytes and used a directed PCR amplification strategy of cDNA to show mis-spliced transcripts in mutation-positive patients.	The loss of an exon in each mis-spliced transcript had no deduced effect on the translational reading frame. The clinical phenotype corresponded closely with genotypic status in family members carrying the KCNQ1 splice variant, but not in family members with the SCN5A splice variant.	These results are put in the context of a literature review, where only 20% of all splice variants reported in the KCNQ1, KCNH2 and SCN5A gene entries in the HGMDPro 2015.4 database have been evaluated using transcriptional assays.	Prediction programs play a strong role in most diagnostic laboratories in classifying variants located at splice sites; however, transcriptional analysis should be considered critical to confirm mis-splicing. Genuine mis-splicing may not always imply clinical significance, and genotype/phenotype cosegregation remains important even when mis-splicing is confirmed.
Papadakis et al. Sudden cardiac death with autopsy findings of uncertain significance: potential for erroneous interpretation.<br>• Year published: 2013<br>• PMID: 23671135	Aim: The sudden death of young individuals is commonly attributed to inherited cardiac disorders, and familial evaluation is advocated. In a proportion of autopsies, structural abnormalities of uncertain significance are reported. The study objective was to explore the hypothesis that such sudden cardiac deaths represent SADS.<br>Size: 41 families	Inclusion criteria: Families in whom the deceased exhibited structural abnormalities of uncertain significance, such as ventricular hypertrophy, myocardial fibrosis, and minor CAD; 163 families in group 2 (SADS cohort) were used as controls for comparison.	Twenty-one families (51%) with autopsy findings of uncertain significance received a diagnosis based on the identification of an inherited cardiac condition phenotype in ≥1 relatives: 14 BrS; 4 LQTS; 1 CPVT; and 2 cardiomyopathy. A similar proportion of families (47.2%) received a diagnosis in the SADS cohort (p = .727). An arrhythmogenic syndrome was the predominant diagnosis in both cohorts (46% versus 45%; p = .863).	Familial evaluation after sudden cardiac deaths with autopsy findings of uncertain significance identified a similar proportion of primary arrhythmogenic syndromes to a contemporary series of SADS.	Need for accurate interpretation of autopsy findings to avoid erroneous diagnoses, with potentially devastating implications.

**Study name or acronym; Author; Year published; PMID**	**Aim of study; Endpoints; Study type (RCT, observational — multicenter or single center; case series or other [if RCT include intervention and comparator]); Study size (N)**	**Patient population with inclusion and exclusion criteria**	**Results (absolute event rates, *p* values; OR or RR; 95% CI)**	**Other relevant findings or adverse events**	**Limitations; Other comments; Conclusions**
Hofman et al. Yield of molecular and clinical testing for arrhythmia syndromes: report of 15 years' experience.<br>• Year published: 2013<br>• PMID: 23963746	Aim: To study the yield of DNA testing for inherited arrhythmia syndromes using a candidate-gene approach over 15 years of experience.<br>Size: 6,944	Among 7021 individuals who were counseled, 6,944 from 2298 different families (aged 41 ± 19 years; 49% male) were analyzed.	In 702 families (31%), a possible disease-causing mutation was detected. Most mutations were found in families with LQTS (47%) or HCM (46%). Cascade screening revealed 1,539 mutation-positive subjects. The mutation detection rate decreased over time, in part because probands with a less severe phenotype were studied, and was significantly higher in familial than in isolated cases.	372 families were counseled after SUD; in 29% of them (n = 108), an inherited arrhythmia syndrome was diagnosed.	The proportion of disease-causing mutations found decreased over time, in part because probands with a less severe phenotype were studied. Systematic screening of families identified many (often presymptomatic) mutation-positive subjects.
Behr et al. Cardiological assessment of first-degree relatives in sudden arrhythmic death syndrome.<br>• Year published: 2003<br>• PMID: 14602442	Aim: To search for evidence of inherited cardiac disease in cases of SADS.<br>Size: 147	Inclusion criteria: 147 first-degree relatives of 32 people who died of SADS	Of 147 first-degree relatives of 32 people who died of SADS, 109 (74%) underwent cardiological assessment. Seven (22%) of the 32 families were diagnosed with inherited cardiac disease: 4 with LQTS; 1 with nonstructural cardiac electrophysiological disease; 1 with myotonic dystrophy; and 1 with HCM.		Families of people who die of SADS should be offered assessment in centers with experience of inherited cardiac disease.
Behr et al. Sudden arrhythmic death syndrome: familial evaluation identifies inheritable heart disease in the majority of families.<br>• Year published: 2008<br>• PMID: 18508782	Aim: To identify more susceptible SADS individuals and causes of death through comprehensive clinical investigation of SADS families.<br>Size: 57 families	Inclusion criteria: Consecutively referred families with SADS death	First-degree relatives [184/262 (70%)] underwent evaluation, 13 (7%) reporting unexplained syncope. 17 (30%) families had a history of additional unexplained premature sudden death(s). 30 families (53%) were diagnosed with inheritable heart disease: 13 definite LQTS, 3 possible/probable LQTS, 5 BrS, 5 ARVC, and 4 other cardiomyopathies.	1 SCN5A and 4 KCNH2 mutations (38%) were identified in 13 definite LQTS families, 1 SCN5A mutation (20%) in 5 BrS families and 1 (25%) PKP2 (plakophyllin2) mutation in 4 ARVC families.	Over-half of SADS deaths were likely to be due to inherited heart disease; accurate identification is vital for appropriate prophylaxis amongst relatives who should undergo comprehensive cardiological evaluation, guided and confirmed by mutation analysis.

Study name or acronym; Author; Year published; PMID	Aim of study; Endpoints; Study type (RCT, observational — multicenter or single center; case series or other [if RCT include intervention and comparator]); Study size (N)	Patient population with inclusion and exclusion criteria	Results (absolute event rates, *p* values; OR or RR; 95% CI)	Other relevant findings or adverse events	Limitations; Other comments; Conclusions
Tan et al. Sudden unexplained death: heritability and diagnostic yield of cardiological and genetic examination in surviving relatives.<br>• Year published: 2005<br>• PMID: 15998675	Aim: Because SUDs may have heritable causes, cardiological and genetic assessment of surviving relatives of SUD victims may reveal the underlying disease and unmask presymptomatic carriers. The study aimed to establish the diagnostic yield of such assessments.<br>Size: 43 families	Inclusion criteria: 43 consecutive families with > or =1 SUD victim who died at < or =40 years of age.	An inherited disease and likely cause of death in 17 of 43 families (40%) were identified; 12 families had primary electrical disease: CPVT (5 families), LQTS (4 families), BrS (2 families), and long-QT/BrS (1 family). ARVC (3 families), HCM (1 family), and FH (1 family) were also found. Molecular genetic analysis provided confirmation in 10 families.	Finding the diagnosis was more likely when more relatives were examined and in families with > or =2 SUD victims < or =40 years of age. The resting / exercise ECG had a high diagnostic yield. These efforts unmasked 151 presymptomatic disease carriers (8.9 per family).	Examination of relatives of young SUD victims has a high diagnostic yield, with identification of the disease in 40% of families and 8.9 presymptomatic carriers per family. Simple procedures (examining many relatives) and routine tests (resting/exercise ECG) constitute excellent diagnostic strategies. Molecular genetics provide strong supportive information.
Papadakis et al. The diagnostic yield of Brugada syndrome after sudden death with normal autopsy.<br>• Year published: 2018<br>• PMID: 29544603	Aim: To assess the impact of systematic ajmaline provo cation testing using high RPLs on the diagnostic yield of BrS in a large cohort of SADS families.<br>Study type: Prospective study<br>Size: 303 SADS families, 911 relatives	303 SADS families (911 relatives) underwent evaluation with resting ECG using conventional and high RPLs, echocardiography, exercise, and 24-h ECG monitor.	An inherited cardiac disease was diagnosed in 128 (42%) families and 201 (22%) relatives. BrS was the most prevalent diagnosis (n = 85, 28% of families; n = 140, 15% of relatives). Ajmaline testing was required to unmask the BrS in 97% of diagnosed individuals.	At initial evaluation, 4 (0.4%) individuals showed a spontaneous type 1 Brugada pattern on the resting ECG, of which 2 were seen only on the high RPLs.	Systematic use of ajmaline testing with high RPLs increases substantially the yield of BrS in SADS families.
**Investigation of Sudden Death: Genetic Evaluation Where the Phenotype Is Known**					
Tan et al. Sudden unexplained death: heritability and diagnostic yield of cardiological and genetic	Aim: Because SUDs may have heritable causes, cardiological and genetic assessment of surviving relatives of SUD victims may reveal the underlying disease and unmask	Inclusion criteria: 43 consecutive families with > or =1 SUD victim who died at < or =40 years of age.	An inherited disease and likely cause of death in 17 of 43 families (40%) were identified; 12 families had primary electrical disease: CPVT (5 families), LQTS (4 families), BrS (2 families), and long-QT/BrS (1 family). ARVC (3 families), HCM (1 family), and	Finding the diagnosis was more likely when more relatives were examined and in families with > or =2 SUD victims < or	Examination of relatives of young SUD victims has a high diagnostic yield, with identification of the disease in 40% of families and 8.9 presymptomatic carriers per family. Simple procedures

**Study name or acronym; Author; Year published; PMID**	**Aim of study; Endpoints; Study type (RCT, observational — multicenter or single center; case series or other [if RCT include intervention and comparator]); Study size (N)**	**Patient population with inclusion and exclusion criteria**	**Results (absolute event rates, *p* values; OR or RR; 95% CI)**	**Other relevant findings or adverse events**	**Limitations; Other comments; Conclusions**
examination in surviving relatives.<br>• Year published: 2005<br>• PMID: 15998675	presymptomatic carriers. The study aimed to establish the diagnostic yield of such assessments.<br>Size: 43 families		FH (1 family) were also found. Molecular genetic analysis provided confirmation in 10 families.	=40 years of age. The resting/ exercise ECG had a high diagnostic yield. These efforts unmasked 151 presymptomatic disease carriers (8.9 per family).	(examining many relatives) and routine tests (resting/exercise ECG) constitute excellent diagnostic strategies. Molecular genetics provide strong supportive information.
Behr et al. Cardiological assessment of first-degree relatives in sudden arrhythmic death syndrome.<br>• Year published: 2003<br>• PMID: 14602442	Aim: To search for evidence of inherited cardiac disease in cases of SADS.<br>Size: 147	Inclusion criteria: 147 first-degree relatives of 32 people who died of SADS	Of 147 first-degree relatives of 32 people who died of SADS, 109 (74%) underwent cardiological assessment. Seven (22%) of the 32 families were diagnosed with inherited cardiac disease: 4 with LQTS; 1 with nonstructural cardiac electrophysiological disease; 1 with myotonic dystrophy; and 1 with HCM.		Families of people who die of SADS should be offered assessment in centers with experience of inherited cardiac disease.
Behr et al. Sudden arrhythmic death syndrome: familial evaluation identifies inheritable heart disease in the majority of families.<br>• Year published: 2008<br>• PMID: 18508782	Aim: To identify more susceptible SADS individuals and causes of death through comprehensive clinical investigation of SADS families.<br>Size: 57 families	Inclusion criteria: Consecutively referred families with SADS death	First-degree relatives [184/262 (70%)] underwent evaluation, 13 (7%) reporting unexplained syncope. 17 (30%) families had a history of additional unexplained premature sudden death(s). 30 families (53%) were diagnosed with inheritable heart disease: 13 definite LQTS, 3 possible/probable LQTS, 5 BrS, 5 ARVC, and 4 other cardiomyopathies.	1 SCN5A and 4 KCNH2 mutations (38%) were identified in 13 definite LQTS families, 1 SCN5A mutation (20%) in 5 BrS families and 1 (25%) PKP2 (plakophyllin2) mutation in 4 ARVC families.	Over-half of SADS deaths were likely to be due to inherited heart disease; accurate identification is vital for appropriate prophylaxis amongst relatives who should undergo comprehensive cardiological evaluation, guided and confirmed by mutation analysis.
Kumar et al. Familial cardiological and	Aim: To assess value of a clinical and genetic protocol for evaluating SCA patients	Inclusion criteria: Unexplained SCA	Clinical diagnosis made in 32 (62%); genetic testing performed in 25/32	Mean age 32	In contrast to previously published series, a comprehensive strategy of

Study name or acronym; Author; Year published; PMID	Aim of study; Endpoints; Study type (RCT, observational — multicenter or single center; case series or other [if RCT include intervention and comparator]); Study size (N)	Patient population with inclusion and exclusion criteria	Results (absolute event rates, *p* values; OR or RR; 95% CI)	Other relevant findings or adverse events	Limitations; Other comments; Conclusions
targeted genetic evaluation: low yield in sudden unexplained death and high yield in unexplained cardiac arrest syndromes.<br>• Year published: 2013<br>• PMID: 23973953	Endpoints: Diagnosis<br>Study type: Case series<br>Size: 52 probands with UCA	Exclusion criteria: An identifiable noncardiac etiology, (2) any evidence of CAD on electrocardiography (ECG) or coronary angiography, and (3) abnormal ventricular function or valvular heart disease.	families (26 genes); pathogenic variant identified in 12/25 tested (yield 48%)		cardiological evaluation and targeted genetic testing in more than 100 families with SADS was found to have a lower diagnostic yield (18%). Diagnostic yield in families with UCA was approximately 4 times higher (62%), which is consistent with the published literature.
Mellor et al. Genetic testing in the evaluation of unexplained cardiac arrest: from the CASPER (Cardiac Arrest Survivors with Preserved Ejection Fraction Registry).<br>• Year published: 2017<br>• PMID: 28600387	Aim: To assess role of genetic testing in individuals without a clinical phenotype.<br>Endpoints: Mutation carrier<br>Study type: Cross-sectional<br>Size: 174 genetic testing/ 375 cardiac arrest survivors in CASPER; genetic testing of cardiac genes at the discretion of the treating provider	Inclusion criteria: UCA with documented VT or VF requiring direct current cardioversion or defibrillation, and subsequent testing demonstrated normal left ventricular function (left ventricular ejection fraction ≥50%) and normal coronary arteries (no coronary stenosis >50%)<br>Exclusion criteria: Known causes for cardiac arrest, including an ECG diagnostic of LQTS (persistent resting QTc >460 ms for males and 480 ms for females) or BrS, HCM, marked hypokalemia, drug overdose, or commotio cordis, recognized forms of idiopathic VT	29 individuals with a pathogenic variant (17% yield); pathogenic variants identified in 18/72 (25%) of phenotype positive patients; pathogenic variants identified in 11/102 (11%) of phenotype negative patients	Mean age 39 years; 18% with ≥VUS; prior syncope (OR 4) and fam hx of SCA (OR 3.2) were associated with pathogenic variant carrier status	Nonstandardized genetic testing panels; no controls; no segregation data.<br>Prior syncope and family history of sudden death are predictors of a positive genetic test. Both arrhythmia and cardiomyopathy genes are implicated. Broad, multiphenotype testing revealed the highest frequency of pathogenic variants in phenotype-negative patients.

Study name or acronym; Author; Year published; PMID	Aim of study; Endpoints; Study type (RCT, observational — multicenter or single center; case series or other [if RCT include intervention and comparator]); Study size (N)	Patient population with inclusion and exclusion criteria	Results (absolute event rates, *p* values; OR or RR; 95% CI)	Other relevant findings or adverse events	Limitations; Other comments; Conclusions
van der Werf et al. Diagnostic yield in sudden unexplained death and aborted cardiac arrest in the young: the experience of a tertiary referral center in the Netherlands.<br>• Year published: 2010<br>• PMID: 20646679	Aim: To find diagnosis in families with SUD victim (140 families) and in SCA survivors (69).<br>Study type: Cross-sectional	Inclusion criteria: SUD victim or SCA survivor aged 1-50 years	Diagnosis found in 33% of SUD families and in 61% of SCA survivors. Comprehensive cardiologic and genetic examination was performed in both populations.	ACA victims, 31 (74%) of the 42 diagnoses made were inherited cardiac diseases.	Inherited cardiac diseases are predominantly causative in both groups.
Bagnall et al. A prospective study of sudden cardiac death among children and young adults.<br>• Year published: 2016<br>• PMID: 27332903	Aim: To define etiology of SCD in Australia and New Zealand 2010-2012.<br>Endpoints: Clinical/genetic diagnosis in proband and family<br>Study type: Prospective population study<br>Size: 490 SCD cases	Inclusion criteria:<br>All SCD cases aged 1-35 years	SCD incidence in Australia/New Zealand was 1.3 per 100,000<br>Unexplained SCD more common in females, when occurring at night, and children aged 1-5 years.<br>54/490 cases were exertional or post-exercise.	Unexplained death the most common etiology; statistically associated with female sex and nocturnal SCD	Not enough evidence to include all cases. The study did not include resuscitated cardiac arrest cases.
Lahrouchi et al. The yield of postmortem genetic testing in sudden death cases with structural findings at autopsy.<br>• Year published: 2020<br>• PMID: 31534214	Aim: To compare the diagnostic yield of postmortem genetic testing in (1) cases with structural findings of uncertain significance at autopsy to (2) cases with autopsy findings diagnostic of cardiomyopathy.<br>Size: 57 SCD cases with structural findings at cardiac autopsy	Inclusion criteria: 57 SCD cases with structural findings at cardiac autopsy were evaluated. NGS using a panel of 77 primary electrical disorder and cardiomyopathy genes was performed.	In 29 cases (51%) autopsy findings of uncertain significance were identified whereas in 28 cases (49%) a diagnosis of cardiomyopathy was established. We identified a pathogenic or likely pathogenic variant in 10 cases (18%); in 1 (3%) case with nonspecific autopsy findings compared with 9 (32%) cases with autopsy findings diagnostic of cardiomyopathy (p = .0054).	The yield of genetic testing in SCD cases with autopsy findings consistent with cardiomyopathy is comparable with the yield in cardiomyopathy patients that are alive.	Genetic testing in cases with findings of uncertain significance offers lower clinical utility than in cardiomyopathy, with lower yields than detected previously. This highlights the need for stringent evaluation of variant pathogenicity.

Study name or acronym; Author; Year published; PMID	Aim of study; Endpoints; Study type (RCT, observational — multicenter or single center; case series or other [if RCT include intervention and comparator]); Study size (N)	Patient population with inclusion and exclusion criteria	Results (absolute event rates, *p* values; OR or RR; 95% CI)	Other relevant findings or adverse events	Limitations; Other comments; Conclusions
Papadakis et al. The diagnostic yield of Brugada syndrome after sudden death with normal autopsy.<br>• Year published: 2018<br>• PMID: 29544603	Aim: To assess the impact of systematic ajmaline provo cation testing using high RPLs on the diagnostic yield of BrS in a large cohort of SADS families.<br>Study type: Prospective study<br>Size: 303 SADS families, 911 relatives	303 SADS families (911 relatives) underwent evaluation with resting ECG using conventional and high RPLs, echocardiography, exercise, and 24-h ECG monitor.	An inherited cardiac disease was diagnosed in 128 (42%) families and 201 (22%) relatives. BrS was the most prevalent diagnosis (n = 85, 28% of families; n = 140, 15% of relatives). Ajmaline testing was required to unmask the BrS in 97% of diagnosed individuals.	At initial evaluation, 4 (0.4%) individuals showed a spontaneous type 1 Brugada pattern on the resting ECG, of which 2 were seen only on the high RPLs.	Systematic use of ajmaline testing with high RPLs increases substantially the yield of BrS in SADS families.
Hofman et al. Active cascade screening in primary inherited arrhythmia syndromes: does it lead to prophylactic treatment?<br>• Year published: 2010<br>• PMID: 20513597	Aim: To investigate the follow-up and treatment of the mutation-carrying relatives of a proband with an inherited arrhythmia syndrome.<br>Size: 130 probands and 509 relatives	Inclusion criteria: From 1996 to 2008, 130 probands with a disease-causing mutation in one of the involved genes were identified, and 509 relatives tested positive for the disease-causing familial mutation. These subjects subsequently underwent cardiologic investigation.	After a mean follow-up of 69 ± 31 months (LQTS), 60 ± 19 months (CPVT), and 56 ± 21 months (BrS), treatment was initiated and ongoing in 65% (199 of 308), 71% (85 of 120), and 6% (5 of 81) of the relatives in the LQTS, CPVT, and BrS families, respectively. Eight carriers were lost to follow-up. Treatment included drug treatment (n = 249) or implantation of PMs (n = 26) or cardioverter-defibrillators (n = 14).	All mutation carriers received lifestyle instructions and a list of drugs to be avoided.	Cascade screening in families with LQTS, BrS, or CPVT, which was based on DNA mutation carrying and subsequent cardiologic investigation, resulted in immediate prophylactic treatment in a substantial proportion of carriers, although these proportions varied significantly between the different diseases.
Manrai et al. Genetic misdiagnoses and the potential for health disparities.<br>• Year published: 2016<br>• PMID: 27532831	Aim: Using publicly accessible exome data, to identify variants that have previously been considered causal in HCM and that are overrepresented in the general population; to study these variants in diverse populations and reevaluated their initial ascertainments	Inclusion criteria: Patient records at a leading genetic-testing laboratory for occurrences of these variants during the near-decade-long history of the laboratory.	Multiple patients, all of whom were of African or unspecified ancestry, received positive reports, with variants misclassified as pathogenic on the basis of the understanding at the time of testing. Subsequently, all reported variants were recategorized as benign. The mutations that were most common in the general population were significantly more	Misclassification of benign variants as pathogenic as found in the study shows the need for sequencing the genomes of diverse populations, both in asymptomatic controls and the	These results expand on current guidelines, which recommend the use of ancestry-matched controls to interpret variants. As additional populations of different ancestry backgrounds are sequenced, variant reclassifications are expected to increase, particularly for ancestry groups that have

Study name or acronym; Author; Year published; PMID	Aim of study; Endpoints; Study type (RCT, observational — multicenter or single center; case series or other [if RCT include intervention and comparator]); Study size (N)	Patient population with inclusion and exclusion criteria	Results (absolute event rates, *p* values; OR or RR; 95% CI)	Other relevant findings or adverse events	Limitations; Other comments; Conclusions
	in the medical literature.		common among black Americans than among white Americans ($p < .001$). Simulations showed that the inclusion of even small numbers of black Americans in control cohorts probably would have prevented these misclassifications. We identified methodologic shortcomings that contributed to these errors in the medical literature.	tested patient population.	historically been less well studied.
Hosseini et al. Reappraisal of reported genes for sudden arrhythmic death: evidence-based evaluation of gene validity for Brugada syndrome.<br>• Year published: 2018<br>• PMID: 29959160	Aim: To evaluate the clinical validity of genes tested by diagnostic laboratories for BrS by assembling 3 gene curation teams.<br>Size: 21 genes curated for clinical validity	Using an evidence-based semiquantitative scoring system of genetic and experimental evidence for gene-disease associations, curation teams independently classified genes as demonstrating limited, moderate, strong, or definitive evidence for disease causation in BrS.	Of 21 genes curated for clinical validity, biocurators classified only 1 gene (SCN5A) as definitive evidence, whereas all other genes were classified as limited evidence.	After comprehensive review by the clinical domain Expert panel, all 20 genes classified as limited evidence were reclassified as disputed with regard to any assertions of disease causality for BrS.	Our results contest the clinical validity of all but 1 gene clinically tested and reported to be associated with BrS. These findings warrant a systematic, evidence-based evaluation for reported gene-disease associations before use in patient care.
Das et al. Determining pathogenicity of genetic variants in hypertrophic cardiomyopathy: importance of periodic reassessment.<br>• Year published: 2014	Aim: To reassess single-nucleotide variant classification in HCM probands.<br>Size: 136 probands	Inclusion criteria: Consecutive probands with HCM with a reported pathogenic mutation or variation of uncertain significance were included. Family and medical history were obtained.	From 2000 to 2012, a total of 136 unrelated HCM probands had genetic testing, of which 63 (46%) carried at least 1 pathogenic mutation. MYBPC3 (n = 34; 47%) and MYH7 (n = 23; 32%) gene variants together accounted for 79%. Five variants in 6 probands (10%) were reclassified: 2 variation of uncertain significance were upgraded to pathogenic, 1 variation of uncertain significance and 1 pathogenic variant	None of the reclassifications had any adverse clinical consequences.	Given the rapid growth of genetic information available in both disease and normal populations, periodic reassessment of single-nucleotide variant data is essential in HCM.

**Study name or acronym; Author; Year published; PMID**	**Aim of study; Endpoints; Study type (RCT, observational — multicenter or single center; case series or other [if RCT include intervention and comparator]); Study size (N)**	**Patient population with inclusion and exclusion criteria**	**Results (absolute event rates, *p* values; OR or RR; 95% CI)**	**Other relevant findings or adverse events**	**Limitations; Other comments; Conclusions**
• PMID: 24113344			were downgraded to benign, and 1 pathogenic variant (found in 2 families) was downgraded to variation of uncertain significance.		
Adler et al. An international, multicentered, evidence-based reappraisal of genes reported to cause congenital long QT syndrome.<br>• Year published: 2020<br>• PMID: 31983240	Aim: Over the last 25 years, multiple genes have been reported to cause LQTS condition and are routinely tested in patients. Because of dramatic changes in our understanding of human genetic variation, reappraisal of reported genetic causes for LQTS is required.<br>Size: 17 genes	Inclusion criteria: Using an evidence-based framework, 3 gene curation teams blinded to each other's work scored the level of evidence for 17 genes reported to cause LQTS. A Clinical Domain Channelopathy Working Group provided a final classification of these genes for causation of LQTS after assessment of the evidence scored by the independent curation teams.	Of 17 genes reported as being causative for LQTS, 9 (AKAP9, ANK2, CAV3, KCNE1, KCNE2, KCNJ2, KCNJ5, SCN4B, SNTA1) were classified as having limited or disputed evidence as LQTS-causative genes. Only 3 genes (KCNQ1, KCNH2, SCN5A) were curated as definitive genes for typical LQTS. Another 4 genes (CALM1, CALM2, CALM3, TRDN) were found to have strong or definitive evidence for causality in LQTS with atypical features, including neonatal AV block. The remaining gene (CACNA1C) had moderate level evidence for causing LQTS.	More than half of the genes reported as causing LQTS have limited or disputed evidence to support their disease causation. Genetic variants in these genes should not be used for clinical decision making, unless accompanied by new and sufficient genetic evidence.	The findings of insufficient evidence to support gene-disease associations may extend to other disciplines of medicine and warrants a contemporary evidence-based evaluation for previously reported disease-causing genes to ensure their appropriate use in precision medicine.
Ackerman et al. Ethnic differences in cardiac potassium channel variants: implications for genetic susceptibility to sudden cardiac death and genetic testing for	Aim: To determine the spectrum, frequency, and ethnic specificity of channel variants in the potassium channel genes implicated in congenital LQTS among healthy subjects.<br>Size: 744	Inclusion criteria: Genomic DNA from 744 apparently healthy individuals (305 black, 187 white, 134 Asian, and 118 Hispanic) was subject to a comprehensive mutational analysis of the 4 LQTS-causing potassium channel genes: KCNQ1 (LQT1),	Overall, 49 distinct amino acid-altering variants (36 novel) were identified: KCNQ1 (n = 16), KCNH2 (n = 25), KCNE1 (n = 5), and KCNE2 (n = 3). More than half of these variants (26/49) were found exclusively in black subjects. The known K897T-HERG and the G38S-min K common polymorphisms were identified in all 4 ethnic groups. Excluding these 2 common polymorphisms, 25% of black subjects had at least 1	Comprehensive determination of the frequency and spectrum of cardiac channel variants found among healthy subjects from 4 major ethnic groups.	Defining the population burden of genetic variants in these critical cardiac ion channels is crucial for proper interpretation of genetic test results of individuals at risk for LQTS. This compendium provides a resource for epidemiological and functional investigation of variant effects on the repolarization properties of cardiac tissues, including

**Study name or acronym; Author; Year published; PMID**	**Aim of study; Endpoints; Study type (RCT, observational — multicenter or single center; case series or other [if RCT include intervention and comparator]); Study size (N)**	**Patient population with inclusion and exclusion criteria**	**Results (absolute event rates, *p* values; OR or RR; 95% CI)**	**Other relevant findings or adverse events**	**Limitations; Other comments; Conclusions**
congenital long QT syndrome.<br>• Year published: 2003<br>• PMID: 14661677		KCNH2 (LQT2), KCNE1 (LQT5), and KCNE2 (LQT6).	nonsynonymous potassium channel variant compared with 14% of white subjects (*p* < .01).		susceptibility to lethal cardiac arrhythmias.
Ackerman et al. Spectrum and prevalence of cardiac sodium channel variants among black, white, Asian, and Hispanic individuals: implications for arrhythmogenic susceptibility and Brugada/long QT syndrome genetic testing.<br>• Year published: 2004<br>• PMID: 15851227	Aim: To determine the prevalence and spectrum of nonsynonymous polymorphisms (amino acid variants) in the cardiac sodium channel among healthy subjects.<br>Size: 829	Inclusion criteria: Using single-stranded conformation polymorphism, denaturing high-performance liquid chromatography, and/or direct DNA sequencing, mutational analysis of the protein-encoding exons of SCN5A was performed on 829 unrelated, anonymous healthy subjects: 319 black, 295 white, 112 Asian, and 103 Hispanic.	In addition to the 4 known common polymorphisms (R34C, H558R, S1103Y, and R1193Q), four relatively ethnic-specific polymorphisms were identified: R481W, S524Y, P1090L, and V1951L. Overall, 39 distinct missense variants (28 novel) were elucidated. Nineteen variants (49%) were found only in the black cohort. Only 7 variants (18%) localized to transmembrane-spanning domains. Four variants (F1293S, R1512W, and V1951L cited previously as BrS1-causing mutations and S1787N previously published as a possible LQT3-causing mutation) were identified in this healthy cohort.	Comprehensive determination of the prevalence and spectrum of cardiac sodium channel variants in healthy subjects from 4 distinct ethnic groups.	This compendium of SCN5A variants is critical for proper interpretation of SCN5A genetic testing and provides an essential hit list of targets for future functional studies to determine whether or not any of these variants mediate genetic susceptibility for arrhythmias in the setting of either drugs or disease.
Kapa et al. Genetic testing for long-QT syndrome: distinguishing pathogenic mutations from benign variants.<br>• Year published: 2009<br>• PMID: 19841300	Aim: To quantify the value of mutation type and gene/protein region in determining the probability of pathogenicity for mutations.<br>Size: 388 unrelated “definite” cases of LQTS and and >1,300 healthy controls for each gene	Inclusion criteria: Type, frequency, and location of mutations across KCNQ1 (LQT1), KCNH2 (LQT2), and SCN5A (LQT3) were compared between 388 unrelated “definite” (clinical diagnostic score >or=4 and/or QTc >or=480 ms) cases of LQTS and >1,300 healthy controls	Mutations were 10 times more common in cases than controls (0.58 per case versus 0.06 per control). Missense mutations were the most common, accounting for 78%, 67%, and 89% of mutations in KCNQ1, KCNH2, and SCN5A in cases and >95% in controls. Nonmissense mutations have an estimated predictive value >99% regardless of location. In contrast, location appears to be critical for characterizing missense mutations.	Distinguishing pathogenic mutations from rare variants is of critical importance in the interpretation of genetic testing in LQTS. Mutation type, mutation location, and ethnic-specific background rates	Novel mutations in low-EPV regions, such as the IDL of SCN5A, should be viewed as VUS and prompt further investigation to clarify the likelihood of disease causation. However, mutations in regions such as the transmembrane, linker, and pore of KCNQ1 and KCNH2 may be defined confidently as high-probability LQTS-causing mutations. These

Study name or acronym; Author; Year published; PMID	Aim of study; Endpoints; Study type (RCT, observational — multicenter or single center; case series or other [if RCT include intervention and comparator]); Study size (N)	Patient population with inclusion and exclusion criteria	Results (absolute event rates, *p* values; OR or RR; 95% CI)	Other relevant findings or adverse events	Limitations; Other comments; Conclusions
		for each gene. From these data, estimated predictive values (percent of mutations found in definite cases that would cause LQTS) were determined according to mutation type and location.	Relative frequency of missense mutations between cases and controls ranged from approximately 1:1 in the SCN5A interdomain linker to infinity in the pore, transmembrane, and linker in KCNH2. These correspond to estimated predictive values ranging from 0% in the interdomain linker of SCN5A to 100% in the transmembrane/linker/ pore regions of KCNH2.	are critical factors in predicting pathogenicity of novel mutations.	findings will have implications for other genetic disorders involving mutational analysis.
Giudicessi et al. Classification and reporting of potentially proarrhythmic common genetic variation in long QT syndrome genetic testing.<br>• Year published: 2018<br>• PMID: 29431662	Aim: To examine the current classification and reporting (or lack thereof) of potentially proarrhythmic common genetic variants and investigate potential mechanisms to facilitate the reporting of these genetic variants without increasing the risk of diagnostic miscues.	This review utilizes LQTS as a prototype to examine the conundrum created by the failure of prevailing paradigms to provide adequate guidance regarding the classification and reporting of common genetic variants that may impact clinical decision making and investigate potential mechanisms by which current ACMG/AMP standards could be modified to better facilitate reporting of these variants.	Although the current ACMG/AMP variant classification and reporting standards provide a solid foundation, the general nature of these guidelines were not intended, nor should they be presumed, to account for the intricacies and idiosyncrasies associated with specific disorders. The application of a “one size fits all” approach to variant interpretation has likely resulted in genetic tests that suboptimally reflect a patient’s true underlying genetic risk of potentially fatal cardiac arrhythmias.	General ACMG/AMP framework needs to be adapted and tailored to fit LQTS and other genetic heart disorders through the development of expert consensus variant guidelines regarding the classification and reporting of rare and common genetic variants.	Until such documents can be thoroughly vetted, the “risk allele” and “functional risk allele” designations put forward in this review may serve as the best, albeit imperfect, stop-gap measure designed to ensure that ordering health care professionals are informed of common variants such as p.Ser1103Tyr-SCN5A and p.Asp85Asn-KCNE1 encountered during disease-specific, pan-cardiac, and clinical whole exome genetic testing and afforded every opportunity to institute the simple interventions needed to mitigate the small but increased risk for sudden death these variants confer.
Giudicessi et al. The genetic architecture of long QT syndrome:	Aim: To reappraise the genetic architecture underlying both the acquired and congenital forms of LQTS by examining	Current paradigms are detailed regarding the molecular and cellular underpinnings of LQTS; the veracity of the QT	Unexpectedly high rate of background genetic variation in the canonical and minor LQTS-susceptibility genes is beginning to reshape our understanding of the genetic	Presence of functional and potentially clinically impactful genetic variation in LQTS-	It is paramount that the field continually reappraise whether a larger than anticipated number of LQTS cases, including many currently labelled as

**Study name or acronym; Author; Year published; PMID**	**Aim of study; Endpoints; Study type (RCT, observational — multicenter or single center; case series or other [if RCT include intervention and comparator]); Study size (N)**	**Patient population with inclusion and exclusion criteria**	**Results (absolute event rates, *p* values; OR or RR; 95% CI)**	**Other relevant findings or adverse events**	**Limitations; Other comments; Conclusions**
a critical reappraisal.<br>• Year published: 2018<br>• PMID: 29661707	how the clinical phenotype associated with and background genetic variation in LQTS-susceptibility genes impacts the clinical validity of existing gene-disease associations and the variant classification and reporting strategies that serve as the foundation for diagnostic LQTS genetic testing.	phenotype associated with specific minor LQTS subtypes is reappraised; ongoing attempts are examined to classify and curate the clinical validity of existing gene-disease associations; and the limitations of current variant interpretation and reporting standards are assessed in an effort to highlight how a critical reappraisal of LQTS genetic architecture can help preserve, and hopefully enhance, the clinical validity of diagnostic LQTS genetic testing.	architecture underlying LQTS. In short order, a number of the minor LQTS-susceptibility genes, previously believed to be responsible for ~5–10% of nonsyndromic LQTS cases, could be demoted to either limited-evidence or disputed-evidence gene status and, at best, be relegated to roles as oligogenic/polygenic contributors.	susceptibility genes whose population frequencies easily exceed overall LQTS and/or LQTS subtype prevalence exposes inherent limitations in our current variant classification and reporting strategies.	minor gene-positive or genotype-negative, are actually oligogenic or polygenic in nature; previous estimates of cLQTS prevalence are indeed accurate; and existing variant classification and reporting strategies accurately reflect the full spectrum of genetic variation that may contribute to LQTS pathogenesis and therefore convey an individual's true genetic risk of disease.
Lahrouchi et al. Utility of post-mortem genetic testing in cases of sudden arrhythmic death syndrome.<br>• Year published: 2017<br>• PMID: 28449774	Aim: To define use of postmortem genetic testing following SCD in negative autopsy SCD.<br>Endpoints: Genetic diagnosis in proband and family<br>Study type: Multicenter, retrospective case-control study<br>Size: 302 SCD cases	Inclusion criteria:<br>SCD cases with available DNA and negative autopsy; negative autopsy<br>Exclusion: Structural heart disease or nonspecific pathological features at autopsy	-Death with exercise (10%) or extreme emotion (1.5%)<br>-Family history in 7.1%<br>-18% personal history of syncope or seizures<br>-21 (7%) diagnosed with epilepsy or prior history of epilepsy<br>-24 had consulted health care provider	-19 pathogenic and 15 likely pathogenic variants in RyR2, KCNH2, KCNQ1, SCN5A<br>-1 pathogenic variant in PLN, likely pathogenic versions in TTN (2), PKP2 (1), MYH7 (1)	-88% European heritage<br>-Limited family evaluation<br>-Referral bias

Study name or acronym; Author; Year published; PMID	Aim of study; Endpoints; Study type (RCT, observational — multicenter or single center; case series or other [if RCT include intervention and comparator]); Study size (N)	Patient population with inclusion and exclusion criteria	Results (absolute event rates, *p* values; OR or RR; 95% CI)	Other relevant findings or adverse events	Limitations; Other comments; Conclusions
**Investigation of Sudden Death: Genetic Evaluation Where the Phenotype Is Unknown**					
van der Werf et al. Low rate of cardiac events in first-degree relatives of diagnosis-negative young sudden unexplained death syndrome victims during follow-up.<br>• Year published: 2014<br>• PMID: 24882506	Aim: To study the prognosis of first-degree relatives of young SUDS victims, in whom the initial cardiologic and genetic examination did not lead to a diagnosis.<br>Size: 417 relatives from 83 families	Inclusion criteria: Vital status of surviving first-degree relatives from 83 diagnosis-negative families who presented to our cardiogenetics department between 1996 and 2009 because of SUDS in ≥1 relatives aged 1-50 years was retrieved.	Detailed information was obtained (median follow-up 6.6 years; IQR 4.7-9.6 years) in 340 of 417 first-degree relatives (81.5%) from 77 of 83 families (92.8%). Vital status, available in 405 relatives (97.1%), showed that 20 relatives (4.9%) died during follow-up, including 1 natural death before the age of 50. This girl belonged to a family with multiple cases of IVF and SUDS, including another successfully resuscitated sibling during follow-up. Two hundred thirty-four of 340 first-degree relatives (68.8%) underwent cardiologic examination. Of these, 76 (32.5%) were reevaluated.	Inherited cardiac disease was diagnosed in 3 families (3.6%).	In first-degree relatives of young SUDS victims with no manifest abnormalities during the initial examination, the risk of developing manifest inherited cardiac disease or cardiac events during follow-up is low. This does not apply to families with obvious familial SUDS.
Tan et al. Sudden unexplained death: heritability and diagnostic yield of cardiological and genetic examination in surviving relatives.<br>• Year published: 2005<br>• PMID: 15998675	Aim: Because SUDs may have heritable causes, cardiological and genetic assessment of surviving relatives of SUD victims may reveal the underlying disease and unmask presymptomatic carriers. The study aimed to establish the diagnostic yield of such assessments.<br>Size: 43 families	Inclusion criteria: 43 consecutive families with > or =1 SUD victim who died at < or =40 years of age.	An inherited disease and likely cause of death in 17 of 43 families (40%) were identified; 12 families had primary electrical disease: CPVT (5 families), LQTS (4 families), BrS (2 families), and long-QT/BrS (1 family). ARVC (3 families), HCM (1 family), and FH (1 family) were also found. Molecular genetic analysis provided confirmation in 10 families.	Finding the diagnosis was more likely when more relatives were examined and in families with > or =2 SUD victims < or =40 years of age. The resting / exercise ECG had a high diagnostic yield. These efforts unmasked 151 presymptomatic disease carriers (8.9 per family).	Examination of relatives of young SUD victims has a high diagnostic yield, with identification of the disease in 40% of families and 8.9 presymptomatic carriers per family. Simple procedures (examining many relatives) and routine tests (resting/exercise ECG) constitute excellent diagnostic strategies. Molecular genetics provide strong supportive information.

**Study name or acronym; Author; Year published; PMID**	**Aim of study; Endpoints; Study type (RCT, observational — multicenter or single center; case series or other [if RCT include intervention and comparator]); Study size (N)**	**Patient population with inclusion and exclusion criteria**	**Results (absolute event rates, *p* values; OR or RR; 95% CI)**	**Other relevant findings or adverse events**	**Limitations; Other comments; Conclusions**
van der Werf et al. Diagnostic yield in sudden unexplained death and aborted cardiac arrest in the young: the experience of a tertiary referral center in the Netherlands.<br>• Year published: 2010<br>• PMID: 20646679	Aim: To find diagnosis in families with SUD victim (140 families) and in SCA survivors (69)<br>Study type: Cross-sectional	Inclusion criteria: SUD victim or SCA survivor aged 1-50 years	Diagnosis found in 33% of SUD families and in 61% of SCA survivors. Comprehensive cardiologic and genetic examination was performed in both populations.	ACA victims, 31 (74%) of the 42 diagnoses made were inherited cardiac diseases.	Inherited cardiac diseases are predominantly causative in both groups.
van der Werf et al. Improving usual care after sudden death in the young with focus on inherited cardiac diseases (the CAREFUL study): a community-based intervention study.<br>• Year published: 2016<br>• PMID: 25833117	Aim: To study the usual care after sudden death in the young aimed at identifying inherited cardiac disease, and assessed the efficacy of two interventions to improve this usual care.<br>Study type: Community-based intervention study<br>Size: 390	A community-based intervention study was conducted to increase autopsy rates of young sudden death victims aged 1-44 years and referral of their relatives to cardiogenetic clinics. In the Amsterdam study region, a 24/7 central telephone number and a website were available to inform general practitioners and coroners. In the Utrecht study region, they were informed by a letter and educational meetings. In two control regions usual care was monitored.	Autopsy was performed in 169 of 390 registered sudden death cases (43.3%). Cardiogenetic evaluation of relatives was indicated in 296 of 390 cases (75.9%), but only 25 of 296 families (8.4%) attended a cardiogenetics clinic. Autopsy rates were 38.7% in the Amsterdam study region, 45.5% in the Utrecht study region, and 49.0% in the control regions. The proportion of families evaluated at cardiogenetics clinics in the Amsterdam study region, the Utrecht study region, and the control regions was 7.3, 9.9, and 8.8%, respectively.		The autopsy rate in young sudden death cases in the Netherlands is low, and few families undergo cardiogenetic evaluation to detect inherited cardiac diseases. Two different interventions did not improve this suboptimal situation substantially.

Study name or acronym; Author; Year published; PMID	Aim of study; Endpoints; Study type (RCT, observational — multicenter or single center; case series or other [if RCT include intervention and comparator]); Study size (N)	Patient population with inclusion and exclusion criteria	Results (absolute event rates, *p* values; OR or RR; 95% CI)	Other relevant findings or adverse events	Limitations; Other comments; Conclusions
Lahrouchi et al. Utility of post-mortem genetic testing in cases of sudden arrhythmic death syndrome.<br>• Year published: 2017<br>• PMID: 28449774	Aim: To define use of postmortem genetic testing following SCD in negative autopsy SCD.<br>Endpoints: Genetic diagnosis in proband and family<br>Study type: Multicenter, retrospective case-control study<br>Size: 302 SCD cases	Inclusion criteria:<br>SCD cases with available DNA and negative autopsy; negative autopsy<br>Exclusion: Structural heart disease or nonspecific pathological features at autopsy	-Death with exercise (10%) or extreme emotion (1.5%)<br>-Family history in 7.1%<br>-18% personal history of syncope or seizures<br>-21 (7%) diagnosed with epilepsy or prior history of epilepsy<br>-24 had consulted health care provider	-19 pathogenic and 15 likely pathogenic variants in RyR2, KCNH2, KCNQ1, SCN5A<br>-1 pathogenic variant in PLN, likely pathogenic versions in TTN (2), PKP2 (1), MYH7 (1)	-88% European heritage<br>-Limited family evaluation<br>-Referral bias
Bagnall et al. A prospective study of sudden cardiac death among children and young adults.<br>• Year published: 2016<br>• PMID: 27332903	Aim: To define etiology of SCD in Australia and New Zealand 2010-2012.<br>Endpoints: Clinical/genetic diagnosis in proband and family<br>Study type: Prospective population study<br>Size: 490 SCD cases	Inclusion criteria:<br>All SCD cases aged 1-35 years	SCD incidence in Australia/New Zealand was 1.3 per 100,000<br>Unexplained SCD more common in females, when occurring at night, and children aged 1-5 years.<br>54/490 cases were exertional or post-exercise.	Unexplained death the most common etiology; statistically associated with female sex and nocturnal SCD	Not enough evidence to include all cases. The study did not include resuscitated cardiac arrest cases.
Tester et al. Targeted mutational analysis of the RyR2-encoded cardiac ryanodine receptor in sudden unexplained death: a molecular autopsy of 49 medical examiner/coroner's cases.	Aim: To perform a molecular autopsy of the RyR2-encoded cardiac ryanodine receptor/calcium release channel in medical examiner/coroner's cases of SUD.<br>Size: 49 SUD cases	From September 1998 to March 2004, 49 cases of SUD were referred by medical examiners/coroners to the Sudden Death Genomics Laboratory at the Mayo Clinic in Rochester, MN, for a cardiac channel molecular autopsy. Mutational analysis of 18 exons of RyR2 implicated previously in	This cohort of 49 cases of SUD included 30 males, 13 with a family history of syncope, cardiac arrest, or SCD (mean ± SD age at death, 14.2 ± 10.9 years). Six distinct RyR2 missense mutations (3 novel) were discovered in 7 cases (14%, 6 males, mean ± SD age at death, 13.6 ± 11.2 years) of SUD. The activities at the time of SUD were exertion (3), emotion (1), and unknown (3). The mutations, R420W, S2246L, N4097S, E4146K, T4158P, and R4497C, involved nonconservative amino acid substitutions in highly	This study represents the first molecular autopsy of RyR2 in medical examiner-referred cases of SUD. A targeted analysis of only 18 of the 105 protein-encoding exons of the cardiac ryanodine receptor/calcium release channel revealed potential	These findings suggest that postmortem genetic testing of RyR2 should be considered as a part of the comprehensive medicolegal autopsy investigation of a SUD case and that this potentially heritable and often elusive arrhythmia syndrome be scrutinized carefully in family members of those who experience SUD.

Study name or acronym; Author; Year published; PMID	Aim of study; Endpoints; Study type (RCT, observational — multicenter or single center; case series or other [if RCT include intervention and comparator]); Study size (N)	Patient population with inclusion and exclusion criteria	Results (absolute event rates, *p* values; OR or RR; 95% CI)	Other relevant findings or adverse events	Limitations; Other comments; Conclusions
• Year published: 2004<br>• PMID: 15544015		the pathogenesis of CPVT was performed on genomic DNA using PCR, denaturing high-performance liquid chromatography, and direct DNA sequencing.	conserved residues across species and were not seen in 400 reference alleles.	CPVT1-causing RyR2 mutations in 1 of every 7 cases of SUD.	
Tester et al. Cardiac channel molecular autopsy: insights from 173 consecutive cases of autopsy-negative sudden unexplained death referred for postmortem genetic testing.<br>• Year published: 2012<br>• PMID: 22677073	Aim: To perform LQTS and CPVT cardiac channel postmortem genetic testing (molecular autopsy) for a large cohort of cases of autopsy-negative SUD.<br>Size: 173 SUD cases	Inclusion criteria: From September 1, 1998, through October 31, 2010, 173 cases of SUD (106 males; mean ± SD age, 18.4 ± 12.9 years; age range, 1-69 years; 89% white) were referred by medical examiners or coroners for a cardiac channel molecular autopsy.	Overall, 45 putative pathogenic mutations absent in 400 to 700 controls were identified in 45 autopsy-negative SUD cases (26.0%). Females had a higher yield (26/67 [38.8%]) than males (19/106 [17.9%]; $p < .005$). Among SUD cases with exercise-induced death, the yield trended higher among the 1- to 10-year-olds (8/12 [66.7%]) compared with the 11- to 20-year-olds (4/27 [14.8%]; $p = .002$). In contrast, for those who died during a period of sleep, the 11- to 20-year-olds had a higher yield (9/25 [36.0%]) than the 1- to 10-year-olds (1/24 [4.2%]; $p = .01$).	Cardiac channel molecular autopsy should be considered in the evaluation of autopsy-negative SUD.	Several interesting genotype-phenotype observations may provide insight into the expected yields of postmortem genetic testing for SUD and assist in selecting cases with the greatest potential for mutation discovery and directing genetic testing efforts.
Anderson et al. Whole-exome molecular autopsy after exertion-related sudden unexplained death in the young.<br>• Year published: 2016<br>• PMID: 27114410	Aim: To evaluate the role of WES in exertion-related SUDY cases<br>Size: 32	Inclusion criteria: From 1998 to 2010, 32 cases of exertion-related SUDY were referred by Medical Examiners for a cardiac channel molecular autopsy.	A mutational analysis of the major LQTS-susceptibility genes (KCNQ1, KCNH2, and SCN5A) and CPVT-susceptibility gene (RYR2) identified a putative pathogenic mutation in 11 cases. Whole-exome sequencing was performed on the remaining 21 targeted gene-negative SUDY cases. After WES, a gene-specific surveillance of all genes (N = 100) implicated in sudden death was performed to identify putative pathogenic	The overall yield of pathogenic mutations was higher among decedents aged 1 to 10 years (10/11, 91%) than those aged 11 to 19 years (4/21, 19%, $p = .0001$).	Molecular screening in this clinical scenario is appropriate with a pathogenic mutation detection rate of 44% using direct DNA sequencing followed by WES. Only 5 of the 100 interrogated sudden death genes hosted actionable pathogenic mutations for more than one-third of these exertion-related, autopsy-negative SUDY cases.

**Study name or acronym; Author; Year published; PMID**	**Aim of study; Endpoints; Study type (RCT, observational — multicenter or single center; case series or other [if RCT include intervention and comparator]); Study size (N)**	**Patient population with inclusion and exclusion criteria**	**Results (absolute event rates, *p* values; OR or RR; 95% CI)**	**Other relevant findings or adverse events**	**Limitations; Other comments; Conclusions**
			mutation(s). Three of these 21 decedents had a clinically actionable, pathogenic mutation (CALM2-F90L, CALM2-N98S, and PKP2-N634fs). Of the 18 remaining cases, 7 hosted at least 1 variant of unknown significance with a minor allele frequency <1:20,000.		
Mellor et al. Genetic testing in the evaluation of unexplained cardiac arrest: from the CASPER (Cardiac Arrest Survivors with Preserved Ejection Fraction Registry).<br>• Year published: 2017<br>• PMID: 28600387	Aim: To assess role of genetic testing in individuals without a clinical phenotype.<br>Endpoints: Mutation carrier<br>Study type: Cross-sectional<br>Size: 174 genetic testing/ 375 cardiac arrest survivors in CASPER; genetic testing of cardiac genes at the discretion of the treating provider	Inclusion criteria: UCA with documented VT or VF requiring direct current cardioversion or defibrillation, and subsequent testing demonstrated normal left ventricular function (left ventricular ejection fraction ≥50%) and normal coronary arteries (no coronary stenosis >50%)<br>Exclusion criteria: Known causes for cardiac arrest, including an ECG diagnostic of LQTS (persistent resting QTc >460 ms for males and 480 ms for females) or BrS, HCM, marked hypokalemia, drug overdose, or commotio cordis, recognized forms of idiopathic VT	29 individuals with a pathogenic variant (17% yield); pathogenic variants identified in 18/72 (25%) of phenotype positive patients; pathogenic variants identified in 11/102 (11%) of phenotype negative patients	Mean age 39 years; 18% with ≥VUS; prior syncope (OR 4) and fam hx of SCA (OR 3.2) were associated with pathogenic variant carrier status	Nonstandardized genetic testing panels; no controls; no segregation data.<br>Prior syncope and family history of sudden death are predictors of a positive genetic test. Both arrhythmia and cardiomyopathy genes are implicated. Broad, multiphenotype testing revealed the highest frequency of pathogenic variants in phenotype-negative patients.

**Study name or acronym; Author; Year published; PMID**	**Aim of study; Endpoints; Study type (RCT, observational — multicenter or single center; case series or other [if RCT include intervention and comparator]); Study size (N)**	**Patient population with inclusion and exclusion criteria**	**Results (absolute event rates, *p* values; OR or RR; 95% CI)**	**Other relevant findings or adverse events**	**Limitations; Other comments; Conclusions**
Giudicessi et al. Role of genetic heart disease in sentinel sudden cardiac arrest survivors across the age spectrum.<br>• Year published: 2018<br>• PMID: 29884292	Aim: To determine the contribution of GHDs in single-center referral cohort of nonischemic SCA survivors.<br>Size: 180	Inclusion criteria: Retrospective analysis of 3,037 patients was used to identify all individuals who experienced a sentinel event of SCA. Following exclusion of patients with ischemic or complex congenital heart disease, cases were classified by clinical diagnoses.	Overall, 180 (5.9%) referral patients experienced a sentinel SCA (average age at SCA 28 ± 15 years, 99 females). An etiology was identified in 113/180 patients (62.8%) including channelopathies in 26.7%, arrhythmogenic bileaflet mitral valve prolapse in 10.6%, cardiomyopathies in 9.4%, other etiologies in 6.7%, aLQTS in 6.7%, and multiple disorders in 2.8%. The remaining 67/180 (37.2%) cases were classified as IVF.	The contribution of GHDs declined precipitously after the first decade of life [90.0% (age 0-9; n = 20), 58.7% (age 10-19; n = 46), 28.1% (age 20-29; n = 32), 23.8% (age 30-39; n = 42), 16.7% (age 40-49; n = 24), and 12.5% (age 50+; n = 16)].	Within a referral population enriched for GHDs, the ability of a comprehensive cardiac evaluation, including genetic testing, to elucidate a root cause in nonischemic SCA survivors declined with age. Although rare, GHDs can underlie SCA into adulthood and merit consideration across the age spectrum.
Shanks et al. Importance of variant interpretation in whole-exome molecular autopsy: population-based case series.<br>• Year published: 2018<br>• PMID: 29915097	Aim: Potentially lethal cardiac channelopathies/ cardiomyopathies may underlie a substantial portion of SUDY. The whole-exome molecular autopsy represents the latest approach to postmortem genetic testing for SUDY. However, proper variant adjudication in the setting of SUDY can be challenging.<br>Size: 25 consecutive cases of SUDY	From January 2012 through December 2013, 25 consecutive cases of SUDY from 1 to 40 years of age (average age at death 27 ± 5.7 years; 13 white, 12 black) from Cook County, Illinois, were referred after a negative (n = 16) or equivocal (n = 9) conventional autopsy. A whole-exome molecular autopsy with analysis of 99 sudden death-susceptibility genes was performed.	Overall, 27 ultrarare nonsynonymous variants were seen in 16/25 (64%) victims of SUDY. Among black individuals, 9/12 (75%) had an ultrarare nonsynonymous variant compared with 7/13 (54%) white individuals. Of the 27 variants, 10 were considered pathogenic or likely pathogenic in 7/25 (28%) individuals in accordance with the ACMG guidelines.	Pathogenic/likely pathogenic variants were identified in 5/16 (31%) of autopsy-negative cases and in 2/6 (33%) victims of SUDY with equivocal findings of cardiomyopathy. Overall, 6 pathogenic/likely pathogenic variants in 4/25 (16%) cases were congruent with the phenotypic findings at autopsy and therefore considered clinically actionable.	Whole-exome molecular autopsy with gene-specific surveillance is an effective approach for the detection of potential pathogenic variants in SUDY cases. However, systematic variant adjudication is crucial to ensure accurate and proper care for surviving family members.

**Study name or acronym; Author; Year published; PMID**	**Aim of study; Endpoints; Study type (RCT, observational — multicenter or single center; case series or other [if RCT include intervention and comparator]); Study size (N)**	**Patient population with inclusion and exclusion criteria**	**Results (absolute event rates, *p* values; OR or RR; 95% CI)**	**Other relevant findings or adverse events**	**Limitations; Other comments; Conclusions**
Narula et al. Post-mortem whole exome sequencing with gene-specific analysis for autopsy-negative sudden unexplained death in the young: a case series.<br>• Year published: 2015<br>• PMID: 25500949	Aim: To determine whether postmortem WES is an efficient strategy to detect ultra-rare, potentially pathogenic variants.<br>Size: 14 consecutively referred Caucasian SUDY victims	Postmortem WES and gene-specific analysis of 117 sudden death-susceptibility genes for 14 consecutively referred Caucasian SUDY victims (average age at death 17.4 ± 8.6 years) to identify putative SUDY-associated mutations was performed.	On average, each SUDY case had 12,758 ± 2,016 nonsynonymous variants, of which 79 ± 15 localized to these 117 genes. Overall, eight ultra-rare variants (7 missense, 1 in-frame insertion) absent in 3 publicly available exome databases were identified in 6 genes (3 in TTN, and 1 each in CACNA1C, JPH2, MYH7, VCL, RYR2) in 7 of 14 cases (50 %).	Of the 7 missense alterations, two (T171M-CACNA1C, I22160T-TTN) were predicted damaging by 3 independent in silico tools.	Although WES and gene-specific surveillance is an efficient means to detect rare genetic variants that might underlie the pathogenic cause of death, accurate interpretation of each variant is challenging. Great restraint and caution must be exercised otherwise families may be informed prematurely and incorrectly that the root cause has been found.
**Investigation of Sudden Cardiac Arrest Survivors: History—Personal and Family**					
Waddell-Smith et al. Inpatient detection of cardiac-inherited disease: the impact of improving family history taking.<br>• Year published: 2016<br>• PMID: 26925241	Aim: To evaluate adequacy of eliciting family history by adult cardiology inpatient teams.<br>Endpoints: Elicit family history of inherited cardiac disease<br>Size: 37	Inclusion criteria: ACA, cardiomyopathy, or VT	37 patients (22 males) were selected: mean age 51 years (range 15-79). Admission presentations included (idiopathic) RSCD (14), dyspnea or heart failure (11), VT (2), other (10). 3 patients had already volunteered their familial diagnosis to the admitting team.	Family history was incompletely elicited in 17 (46%) and absent in 20 (54%).	Adult cardiology inpatient teams are poor at recording family history and need to be reminded of its powerful diagnostic value.
Krahn et al. Systematic assessment of patients with unexplained cardiac arrest: Cardiac Arrest Survivors with Preserved Ejection	Aim: To unmask cause of ACA with drugs and imaging.<br>Study type: Prospective analysis<br>Size: 63 patients	Inclusion criteria: UCA without evident cardiac disease	Diagnosis was obtained in 56% including LQTS, CPVT, BrS, early repolarization, ARVC, coronary spasm, myocarditis.	Systematic clinical testing, including drug provocation and advanced imaging, results in unmasking of the cause of apparently UCA in >50% of patients.	This approach assists in directing genetic testing to diagnose genetically mediated arrhythmia syndromes, which results in successful family screening.

**Study name or acronym; Author; Year published; PMID**	**Aim of study; Endpoints; Study type (RCT, observational — multicenter or single center; case series or other [if RCT include intervention and comparator]); Study size (N)**	**Patient population with inclusion and exclusion criteria**	**Results (absolute event rates, *p* values; OR or RR; 95% CI)**	**Other relevant findings or adverse events**	**Limitations; Other comments; Conclusions**
Fraction Registry (CASPER).<br>• Year published: 2009<br>• PMID: 19597050					
Marstrand et al. Clinical evaluation of unselected cardiac arrest survivors in a tertiary center over a 1-year period (the LAZARUZ study).<br>• Year published: 2016<br>• PMID: 27237785	Aim: To find cause of ACA<br>Study type: Observational<br>Size: 43 subjects	Inclusion criteria:<br>All ACA survivors ages 18-64 years	Diagnosis identified in 63% including IHD, cardiomyopathy, miscellaneous (drug, spasm), channelopathy	16/43 (37%) of unselected, prospectively included cardiac arrest survivors remained without a diagnosis despite exhaustive investigations.	Family history did not predict etiology among cardiac arrest survivors. Performing the 'brisk standing' test to induce postural sinus tachycardia and 'elevation of precordial leads V1–2' may be reasonable diagnostic tricks among cardiac arrest survivors in whom no diagnosis can be reached despite extensive evaluation.
Waldmann et al. Characteristics and clinical assessment of unexplained sudden cardiac arrest in the real-world setting: focus on idiopathic ventricular fibrillation.<br>• Year published: 2018<br>• PMID: 29566157	Aim: To find cause of ACA.<br>Study type: Prospective observational<br>Size: 717	Data were analyzed from an ongoing study, collecting all cases of OHCA in Paris area. Investigations performed during the index hospitalization or planned after discharge were gathered to evaluate the completeness of assessment of unexplained SCA. Between 2011 and 2016, among the 18,622 OHCA, 717 survivors (at hospital discharge) fulfilled the definition of cardiac SCA.	Diagnosis in 93% included CAD, cardiomyopathy, channelopathies, misc.		Complete investigations are carried out in a very low proportion of unexplained SCA. Standardized, systematic approaches need to be implemented to ensure that opportunities for specific therapies and preventive strategies (including relatives) are not missed.

**Study name or acronym; Author; Year published; PMID**	**Aim of study; Endpoints; Study type (RCT, observational — multicenter or single center; case series or other [if RCT include intervention and comparator]); Study size (N)**	**Patient population with inclusion and exclusion criteria**	**Results (absolute event rates, *p* values; OR or RR; 95% CI)**	**Other relevant findings or adverse events**	**Limitations; Other comments; Conclusions**
Allan et al. Unexpected high prevalence of cardiovascular disease risk factors and psychiatric disease among young people with sudden cardiac arrest.<br>• Year published: 2019<br>• PMID: 30661423	Aim: To find risk factors for SCD/ACA in young people.<br>Study type: Observational<br>Size: 608	Inclusion criteria: SCA (death or ACA) ages 2-45 years	2/3 had history of cardiac disease, 50% had at least 1 cardiac risk factor, 20% had psychiatric disease, over 30% had CNS active drugs detected.	Potentially heritable structural cardiac diseases accounted for only 6.9% of SCA events, with acquired cardiac diseases comprising the rest.	The underlying causes of SCA, in people aged 2 to 45 years, often occur in those with previously diagnosed cardiovascular diseases, and are associated with contributory factors including prescribed medications, recreational drugs, and a concomitant psychiatric history.
Herman et al. Outcome of apparently unexplained cardiac arrest: results from investigation and follow-up of the prospective cardiac arrest survivors with preserved ejection fraction registry.<br>• Year published: 2016<br>• PMID: 26783233	Aim: To investigate the usefulness of exercise testing, drug provocation, advanced cardiac imaging, and genetic testing.<br>Study type: Observational<br>Size: 200 survivors of UCA	Inclusion criteria: 200 survivors of UCA	Age, 48.6 ± 14.7 years, 41% female. Advanced testing determined a diagnosis in 34% of patients at baseline, with a diagnosis emerging during follow-up in 7% of patients. Of those who were diagnosed, 28 (35%) had an underlying structural condition and 53 (65%) had a primary electric disease. During a mean follow-up of 3.15 ± 2.34 years, 23% of patients had either a shock or an appropriate antitachycardia pacing from their ICD, or both.	The ICD appropriate intervention rate was 8.4% at 1 year and 18.1% at 3 years, with no clear difference between diagnosed and undiagnosed subjects, or between those diagnosed with a primary electric versus structural pathogenesis.	Obtaining a diagnosis in previously UCA patients requires systematic clinical testing and regular follow-up to unmask the cause. Nearly half of apparently UCA patients ultimately received a diagnosis, allowing for improved treatment and family screening. A substantial proportion of patients received appropriate ICD therapy during medium-term follow-up.

Study name or acronym; Author; Year published; PMID	Aim of study; Endpoints; Study type (RCT, observational — multicenter or single center; case series or other [if RCT include intervention and comparator]); Study size (N)	Patient population with inclusion and exclusion criteria	Results (absolute event rates, *p* values; OR or RR; 95% CI)	Other relevant findings or adverse events	Limitations; Other comments; Conclusions
**Investigation of Sudden Cardiac Arrest Survivors: Examination**					
Mangili et al. Achilles tendon xanthomas are associated with the presence and burden of subclinical coronary atherosclerosis in heterozygous familial hypercholesterolemia: a pilot study.<br>• Year published: 2017<br>• PMID: 28499609	Aim: To evaluate the ATX association with the presence and extent of subclinical coronary atherosclerosis in heterozygous FH patients.<br>Size: 102	Inclusion criteria: FH patients diagnosed by US-MEDPED criteria (67% with genetically proven FH), with median LDL-C 279 mg/dL (IQR 240; 313), asymptomatic for cardiovascular disease	Patients with ATX (n = 21, 21%) had higher LDL-C and lipoprotein(a) [Lp(a)] concentrations as well as greater CAC scores, SIS and SSS ($p < .05$). After adjusting for age, sex, smoking, hypertension, previous statin use, HDL-C, LDL-C and Lp(a) concentrations, there was an independent positive association of ATX presence with CAC scores (β = 1.017, $p < .001$), SSS (β = 0.809, $p < .001$) and SIS (β = 0.640, $p < .001$).		ATX are independently associated with the extension of subclinical coronary atherosclerosis quantified by tomographic scores in FH patients.
Maruthappu et al. Loss-of-function desmoplakin I and II mutations underlie dominant arrhythmogenic cardiomyopathy with a hair and skin phenotype.<br>• Year published: 2019<br>• PMID: 30382575	Aim: To systematically investigate the presence of a skin and hair phenotype in heterozygous DSP mutation carriers with ACM.<br>Size: 6 ACM pedigrees with 38 carriers	6 ACM pedigrees with 38 carriers of a dominant loss-of-function (nonsense or frameshift) mutation in DSP were evaluated by detailed clinical examination (cardiac, hair and skin) and molecular phenotyping.	All carriers with mutations affecting both major DSP isoforms (DSPI and II) were observed to have curly or wavy hair in the pedigrees examined, except for members of Family 6, where the position of the mutation only affected the cardiac-specific isoform DSPI. A mild palmoplantar keratoderma was also present in many carriers. Sanger sequencing of cDNA from nonlesional carrier skin suggested degradation of the mutant allele.	Immunohistochemistry of patient skin demonstrated mislocalization of DSP and other junctional proteins (plakoglobin, connexin 43) in the basal epidermis. However, in Family 6, DSP localization was comparable with control skin.	This study identifies a highly recognizable cutaneous phenotype associated with dominant loss-of-function DSPI/II mutations underlying ACM. Increased awareness of this phenotype among health care workers could facilitate a timely diagnosis of ACM in the absence of overt cardiac features.
Clemens et al. International Triadin Knockout	Aim: International Triadin Knockout Syndrome Registry established to include patients who have	Clinical/genetic data were collected using an online survey	Currently, the International Triadin Knockout Syndrome Registry includes 21 patients (11 males, average age of 18 years) from 16 families. 20 patients	Despite various treatment strategies, 14 (74%) patients have had	TKOS is a potentially lethal disease characterized by T-wave inversions in the precordial leads, transient QT prolongation in

Study name or acronym; Author; Year published; PMID	Aim of study; Endpoints; Study type (RCT, observational — multicenter or single center; case series or other [if RCT include intervention and comparator]); Study size (N)	Patient population with inclusion and exclusion criteria	Results (absolute event rates, *p* values; OR or RR; 95% CI)	Other relevant findings or adverse events	Limitations; Other comments; Conclusions
Syndrome Registry.<br>• Year published: 2019<br>• PMID: 30649896	genetically proven homozygous/compound heterozygous TRDN null mutations.<br>Size: 21 patients from 16 families	generated through REDCap.	(95%) presented with either cardiac arrest (15, 71%) or syncope (5, 24%) at an average age of 3 years. Mild skeletal myopathy/proximal muscle weakness was noted in 6 (29%) patients. Of the 19 surviving patients, 16 (84%) exhibit T-wave inversions, and 10 (53%) have transient QT prolongation > 480 ms. 8 of 9 patients had ventricular ectopy on EST. 13 (68%) patients have received implantable defibrillators.	recurrent breakthrough cardiac events.	some, and recurrent ventricular arrhythmias at a young age despite aggressive treatment. Patients displaying this phenotype should undergo TRDN genetic testing as TKOS may be a cause for otherwise UCA in young children. As gene therapy advances, enrollment into the International Triadin Knockout Syndrome Registry is encouraged.
Walsh et al. A multicentre study of patients with Timothy syndrome.<br>• Year published: 2018<br>• PMID: 28371864	Aim: To categorize the phenotypes and examine the outcomes of patients with TS.<br>Size: 6	Inclusion criteria: All patients diagnosed with TS in the United Kingdom over a 24-year period were reviewed. 15 centers in the British Congenital Arrhythmia Group network were contacted to partake in the study. Six patients with TS were identified over a 24-year period (4 boys and 2 girls).	Five out of the 6 patients were confirmed to have a CACNA1C mutation (p.Gly406Arg) and the other patient was diagnosed clinically. Early presentation with heart block, due to QT prolongation was frequently seen. Four are still alive, two of these have a PM and two have undergone defibrillator implantation. Five out of 6 patients have had a documented cardiac arrest with 3 occurring under general anesthesia.	Two patients suffered a cardiac arrest while in hospital and resuscitation was unsuccessful, despite immediate access to a defibrillator.	TS is a rare disorder with a high attrition rate if undiagnosed. Perioperative cardiac arrests are common and not always amenable to resuscitation. Longer-term survival is possible; however, patients invariably require PM or defibrillator implantation.
Michowitz et al. Fever-related arrhythmic events in the multicenter Survey on Arrhythmic Events in Brugada Syndrome.<br>• Year published: 2018<br>• PMID: 29649615	Aim: To describe the characteristics of fever-related AE in a large cohort of BrS patients.<br>Size: 678	Multicenter study on 678 patients with BrS with first AE documented at the time of ACA (n = 426) or after prophylactic ICD implantation (n = 252).	6% of AE in BrS were associated with fever.	The highest proportion of fever-related AE was observed in the pediatric population (age <16), with disproportionally higher event rate in the very young (0-5 years old)	In 13.3% subjects from SABRUS there was no information.<br><br>The risk of fever-related AE in BrS markedly varies according to age group, sex, and ethnicity.

Study name or acronym; Author; Year published; PMID	Aim of study; Endpoints; Study type (RCT, observational — multicenter or single center; case series or other [if RCT include intervention and comparator]); Study size (N)	Patient population with inclusion and exclusion criteria	Results (absolute event rates, *p* values; OR or RR; 95% CI)	Other relevant findings or adverse events	Limitations; Other comments; Conclusions
**Investigation of Sudden Cardiac Arrest Survivors: Baseline Investigations**					
Tseng et al. Prospective countywide surveillance and autopsy characterization of sudden cardiac death: POST SCD study.<br>• Year published: 2018<br>• PMID: 29915095	Aim: To determine the precise incidence and underlying causes of all incident SCDs in San Francisco County.<br>Study type: Retrospective + prospective	Inclusion: SCD defined by WHO—OHCA or not<br>Exclusion: Other clear cause	Leading causes were coronary disease (32%), occult overdose (13.5%), cardiomyopathy (10%), cardiac hypertrophy (8%), and neurologic (5.5%).	Cardiac arrests defined by paramedic criteria and sudden cardiac deaths defined by conventional and/or retrospective methods, as in most cohort studies or clinical trials, have limited accuracy for actual arrhythmic deaths.<br>55% of death presumed to be arrhythmic were not.	Standardized investigations and prospective premortem data collection as done in cohort studies were not possible in the population-based POST SCD study.
Avari Silva et al. Implantable loop recorder monitoring for refining management of children with inherited arrhythmia syndromes.<br>• Year published: 2016<br>• PMID: 27231019	Aim: To assess the impact of ILRs on management of pediatric patients with known or suspected inherited arrhythmia syndromes.<br>Size: 20 pediatric patients	Inclusion criteria: A retrospective chart review was undertaken of all pediatric patients with known or suspected inherited arrhythmia syndromes in whom an ILR was implanted from 2008 to 2015. A total of 20 patients met the stated inclusion criteria (LQTS, n = 8; CPVT, n = 9; BrS, n = 1; ARVC, n = 2), with 60% of patients being genotype positive.	Primary indication for implantation of ILR included ongoing monitoring +/- symptoms (n = 15, 75%), suspicion of noncompliance (n = 1, 5%), and liberalization of recommended activity restrictions (n = 4, 25%). A total of 172 transmissions were received in patients with inherited arrhythmia syndromes, with 7% yielding actionable data. The majority (52%) of symptom events were documented in the LQTS population, with only 1 tracing (5%) yielding actionable data.	Automatic transmissions were mostly seen in the CPVT cohort (81%), with 21% yielding actionable data. There was no actionable data in routine transmissions.	ILRs in patients with suspected or confirmed inherited arrhythmia syndromes may be useful for guiding management. Findings escalated therapies in 30% of subjects. As importantly, in this high-risk population, the majority of symptom events represented normal or benign rhythms, reassuring patients and physicians that no further intervention was required.

**Study name or acronym; Author; Year published; PMID**	**Aim of study; Endpoints; Study type (RCT, observational — multicenter or single center; case series or other [if RCT include intervention and comparator]); Study size (N)**	**Patient population with inclusion and exclusion criteria**	**Results (absolute event rates, *p* values; OR or RR; 95% CI)**	**Other relevant findings or adverse events**	**Limitations; Other comments; Conclusions**
Placidi et al. Miniaturized implantable loop recorder in small patients: an effective approach to the evaluation of subjects at risk of sudden death.<br>• Year published: 2016<br>• PMID: 27062386	Aim: To evaluate the efficacy of miniaturized cardiac implantable devices in the early diagnosis of arrhythmias in children ≤6 years.<br>Size: 21	Inclusion criteria: Patients (median age 5 years) who underwent implantation of miniaturized ILR after a complete cardiac workup	1 patient underwent device removal for pocket infection, and 1 needed a pocket revision. 11 (52%) patients did not show any symptom and/or arrhythmia. 8 patients experienced symptoms during ILR monitoring: 6 had no electrocardiographic abnormalities, 2 had significant sinus pauses. 2 patients had significant arrhythmias without symptoms and in 1 of these a PM was implanted. The overall diagnostic yield was 47%.	Miniaturized ILR could be very useful to make a diagnosis and to decide future management strategies in small patients with undefined symptoms or severe cardiac diseases.	Considering its characteristics, miniaturized ILR could start a new era in the diagnosis and follow-up of young patients with symptomatic and/or malignant arrhythmias.
Sweeney et al. Bradycardia pacing-induced short-long-short sequences at the onset of ventricular tachyarrhythmias: a possible mechanism of proarrhythmia?<br>• Year published: 2007<br>• PMID: 17692746	Aim: To characterize interactions between normal pacing system operation and the initiating sequence of VT/VF.	Initiating sequences of 1,356 VT/VF episodes in the PainFree Rx II (n = 634) and EnTrust Trial (n = 421) were analyzed with stored electrograms and by pacing mode (DDD/R, VVI/R, and managed ventricular pacing [MVP]). Interactions between pacing and VT/VF initiation were classified as: nonpacing associated, pacing associated, pacing permitted, and pacing facilitated.	Nonpacing associated (no pacing, no S-L-S) and pacing associated (ventricular pacing without S-L-S) onset accounted for 44.0% and 29.8% of all VT/VF, respectively. Pacing permitted (S-L-S sequences without ventricular pacing) episodes accounted for 6.4% (DDD/R), 20.0% (MVP), and 25.6% (VVI/R) of 1,356 VT/VF episodes. Pacing facilitated onset (S-L-S sequences actively facilitated by ventricular pacing including the terminal beat after a pause) accounted for 8.2% (MVP), 9.4% (VVI/R), and 14.8% (DDD/R) of 1,356 VT/VF episodes. Pacing facilitated S-L-S VT/VF occurred in 2.6% (MVP), 3.3% (VVI/R), and 5.2% (DDD/R) of patients with episodes and was the sole initiating sequence in approximately 1% of patients.	Pause durations during pacing facilitated S-L-S differed between modes (DDD/R 793 ± 172 ms vs. MVP 865 ± 278 ms vs. VVI/R 1180 ± 414 ms, *p* = .002). The majority of these episodes were monomorphic VT.	VT/VF in some ICD patients might be initiated by S-L-S sequences that are actively facilitated by bradycardia pacing operation and might constitute an important mechanism of ventricular proarrhythmia.

**Study name or acronym; Author; Year published; PMID**	**Aim of study; Endpoints; Study type (RCT, observational — multicenter or single center; case series or other [if RCT include intervention and comparator]); Study size (N)**	**Patient population with inclusion and exclusion criteria**	**Results (absolute event rates, *p* values; OR or RR; 95% CI)**	**Other relevant findings or adverse events**	**Limitations; Other comments; Conclusions**
Aizawa et al. Dynamicity of the J-wave in IVF with a special reference to pause-dependent augmentation of the J-wave.<br>• Year published: 2012<br>• PMID: 22624834	Aim: To evaluate the pause-dependency of the J-wave to characterize this phenomenon in IVF.<br>Size: 40 patients	Inclusion criteria: 40 patients with J-wave-associated IVF were studied for J waves with special reference concerning pause-dependent augmentation. J waves were defined as those ≥0.1 mV above the isoelectric line and were compared with 76 non-VF patients of comparable age and sex.	The J-wave was larger in patients with idiopathic VF than in the controls: 0.360 ± 0.181 mV versus 0.192 ± 0.064 mV (*p* = .0011). J waves were augmented during storms of VF (n = 9 [22.5%]), which was controlled by isoproterenol; they disappeared within weeks in 5 patients. Sudden prolongation of the R-R interval was observed in 27 patients induced by benign arrhythmia, and 15 patients (55.6%) demonstrated pause-dependent augmentation (from 0.391 ± 0.126 mV to 0.549 ± 0.220 mV; *p* < .0001). In the other 12 experimental subjects and in the 76 control subjects, J waves remained unchanged.	Pause-dependent augmentation of J waves was detected in 55.6% (sensitivity), but was specific (100%) in the patients with IVF with high positive (100%) and negative (86.4%) predictive values.	Pause-dependent augmentation of J waves was confirmed in about one-half of the patients with IVF after sudden R-R prolongation. Such dynamicity of J waves was specific to IVF and may be used for risk stratification.
Tsuda et al. Significance of automated external defibrillator in identifying lethal ventricular arrhythmias.<br>• Year published: 2019<br>• PMID: 31297625	Aim: To conduct retrospective cohort study of patients with aborted sudden cardiac death; AED is critical in saving children who develop unexpected cardiac arrest (CA), but its diagnostic capacity is not fully acknowledged.	25 patients (14 males) aged 1.3 to 17.5 years who presented with CA survived with prompt CPR. 18 patients had no prior cardiac diagnosis. CA occurred in 10 patients with more than moderate exercise, in 7 with light exercise, and in 8 at rest (including one during sleep). 22 patients were resuscitated with AED, all of which were recognized as a shockable cardiac rhythm.	Thorough investigations revealed 6 ion channelopathies (4 CPVT, 1 LQTS, and 1 BrS), 5 congenital heart disease (including 2 with coronary artery obstruction), 6 cardiomyopathies, 2 myocarditis, and 2 miscellaneous. 4 patients had no identifiable heart disease. In 5 patients, the downloaded AED-recorded rhythm strip delineated the underlying arrhythmias and their responses to electrical shocks. 4 patients who presented with generalized seizure at rest were initially managed for seizure disorder until AED recording identified lethal ventricular arrhythmias.	AED reliably identifies the underlying lethal ventricular arrhythmias in addition to aborting SCD.	AED is both therapeutic in aborting SCD and diagnostic in identifying the underlying lethal ventricular arrhythmias. However, the diagnostic aspect of AED is underacknowledged by most medical providers.

Study name or acronym; Author; Year published; PMID	Aim of study; Endpoints; Study type (RCT, observational — multicenter or single center; case series or other [if RCT include intervention and comparator]); Study size (N)	Patient population with inclusion and exclusion criteria	Results (absolute event rates, *p* values; OR or RR; 95% CI)	Other relevant findings or adverse events	Limitations; Other comments; Conclusions
Krahn et al. Systematic assessment of patients with unexplained cardiac arrest: Cardiac Arrest Survivors with Preserved Ejection Fraction Registry (CASPER).<br>• Year published: 2009<br>• PMID: 19597050	Aim: To unmask cause of ACA with drugs and imaging.<br>Study type: Prospective analysis<br>Size: 63 patients	Inclusion criteria: UCA without evident cardiac disease	Diagnosis was obtained in 56% including LQTS, CPVT, BrS, early repolarization, ARVC, coronary spasm, myocarditis.	Systematic clinical testing, including drug provocation and advanced imaging, results in unmasking of the cause of apparently UCA in >50% of patients.	This approach assists in directing genetic testing to diagnose genetically mediated arrhythmia syndromes, which results in successful family screening.
Jiménez-Jáimez et al. Diagnostic approach to unexplained cardiac arrest (from the FIVI-Gen Study).<br>• Year published: 2015<br>• PMID: 26189708	Aim: To assess a clinical and genetic diagnostic protocol for UCA.<br>Endpoints: Diagnosis<br>Study type: Multicenter retrospective cohort<br>Size: 35 unexplained SCA survivors; 126 heart genes tested	Inclusion criteria: UCA (VF with no diagnostic findings on the ECG, no pathologic findings on the echocardiogram, and no angiographic lesions with >50% stenosis on coronary catheterization)<br>Exclusion criteria: prolonged QT (QTc interval >460 ms in male and >480 ms in female subjects), Brugada pattern in RPLs and/or pathologic Q waves, documented monomorphic VT, structural heart dis-ease on transthoracic echocardiogram or	Diagnosis made in 51% of cases; 20% based on pharmacologic testing; 14% based on family assessment; 21% based on genetic testing	Mean age 40 years	If interpreted carefully, genetic tests can be a useful tool for diagnosing UCA without a phenotype.

**Study name or acronym; Author; Year published; PMID**	**Aim of study; Endpoints; Study type (RCT, observational — multicenter or single center; case series or other [if RCT include intervention and comparator]); Study size (N)**	**Patient population with inclusion and exclusion criteria**	**Results (absolute event rates, *p* values; OR or RR; 95% CI)**	**Other relevant findings or adverse events**	**Limitations; Other comments; Conclusions**
		CMR, probable channelopathy in the proband or first-degree relatives, or ARVC, in accordance with published criteria, evidence of drug use, severe electrolyte abnormality, ischemia, extreme bradycardia, or any other secondary cause of VF and coronary artery anomaly/vasospasm			
van der Werf et al. Diagnostic yield in sudden unexplained death and aborted cardiac arrest in the young: the experience of a tertiary referral center in the Netherlands.<br>• Year published: 2010<br>• PMID: 20646679	Aim: To find diagnosis in families with SUD victim (140 families) and in SCA survivors (69).<br>Study type: Cross-sectional	Inclusion criteria: SUD victim or SCA survivor aged 1-50 years	Diagnosis found in 33% of SUD families and in 61% of SCA survivors. Comprehensive cardiologic and genetic examination was performed in both populations.	ACA victims, 31 (74%) of the 42 diagnoses made were inherited cardiac diseases.	Inherited cardiac diseases are predominantly causative in both groups.
Haissaguerre et al. Sudden cardiac arrest associated with early repolarization.<br>• Year published: 2008	Aim: Early repolarization is a common electrocardiographic finding that is generally considered to be benign. Its potential to cause cardiac arrhythmias has been hypothesized from	Inclusion criteria: Data from 206 case subjects at 22 centers who were resuscitated after cardiac arrest due to IVF. The control group comprised 412 subjects	Early repolarization was more frequent in case subjects with IVF than in control subjects (31% vs. 5%, $p < .001$). Among case subjects, those with early repolarization were more likely to be male and to have a history of syncope or SCA during sleep than	During a mean (SD) follow-up of 61 (50) months, defibrillator monitoring showed a higher incidence of recurrent VF in	Among patients with a history of IVF, there is an increased prevalence of early repolarization.

**Study name or acronym; Author; Year published; PMID**	**Aim of study; Endpoints; Study type (RCT, observational — multicenter or single center; case series or other [if RCT include intervention and comparator]); Study size (N)**	**Patient population with inclusion and exclusion criteria**	**Results (absolute event rates, *p* values; OR or RR; 95% CI)**	**Other relevant findings or adverse events**	**Limitations; Other comments; Conclusions**
• PMID: 18463377	experimental studies, but it is not known whether there is a clinical association with SCA.<br>Size: 206 case subjects	without heart disease who were matched for age, sex, race, and level of physical activity.	those without early repolarization. In eight subjects, the origin of ectopy that initiated ventricular arrhythmias was mapped to sites concordant with the localization of repolarization abnormalities.	case subjects with a repolarization abnormality than in those without such an abnormality (hazard ratio, 2.1; 95% CI, 1.2 to 3.5; *p* = .008).	
Conte et al. Out-of-hospital cardiac arrest due to IVF in patients with normal electrocardiograms: results from a multicentre long-term registry.<br>• Year published: 2019<br>• PMID: 31504477	Aim: To define the clinical characteristics and long-term clinical outcomes of a large cohort of patients with IVF and normal 12-lead ECGs.<br>Size: 245 patients	Inclusion criteria: Patients with VF as the presenting rhythm, normal baseline, and follow-up ECGs with no signs of cardiac channelopathy including early repolarization or AV conduction abnormalities, and without structural heart disease.	Over a median follow-up of 63 months (IQR 25-110 months), 12 patients died (5%); in 4 of them (1.6%) the lethal event was of cardiac origin. Patients treated with antiarrhythmic drugs only had a higher rate of cardiovascular death compared to patients who received an ICD (16% vs. 0.4%, *p* = .001). Fifty-two patients (21%) experienced an arrhythmic recurrence.	Age ≤16 years at the time of the first ventricular arrhythmia was the only predictor of arrhythmic recurrence on multivariable analysis (HR 0.41, 95% CI 0.18-0.92; *p* = .03).	Patients with IVF and persistently normal ECGs frequently have arrhythmic recurrences, but a good prognosis when treated with an ICD. Children are a category of IVF patients at higher risk of arrhythmic recurrences.
Curcio et al. Clinical presentation and outcome of Brugada syndrome diagnosed with the new 2013 criteria.<br>• Year published: 2016<br>• PMID: 27098113	Aim: To assess the role of High-ICS in the analysis of BrS and the clinical profile of the patients diagnosed only when ECG leads are moved to upper ICS.<br>Size: 300	Inclusion criteria: 300 subjects (age 36 ± 13 years), without a diagnostic coved ST-segment elevation in conventional V1 -V3 leads, both at baseline and after provocative drug challenge.	64 subjects (21.3%, mean age at last follow-up 42 ± 11 years) were diagnosed with High-ICS. Diagnosis was possible at baseline only in 4 subjects, while in 60 it was made after drug challenge with sodium channel blockers. 3 subjects (4.7%) with spontaneous abnormal ECG experienced cardiac events with an annual event rate (0.11%) superimposable to that of the low risk category of BrS diagnosed in standard leads.	Use of new diagnostic criteria for BrS allows increasing the diagnostic yield by 20% and that the arrhythmic risk is low when BrS can be established only in High-ICS.	The prognostic value of spontaneous ECG pattern is confirmed in this subgroup.

**Study name or acronym; Author; Year published; PMID**	**Aim of study; Endpoints; Study type (RCT, observational — multicenter or single center; case series or other [if RCT include intervention and comparator]); Study size (N)**	**Patient population with inclusion and exclusion criteria**	**Results (absolute event rates, *p* values; OR or RR; 95% CI)**	**Other relevant findings or adverse events**	**Limitations; Other comments; Conclusions**
Govindan et al. Utility of high and standard right precordial leads during ajmaline testing for the diagnosis of Brugada syndrome.<br>• Year published: 2010<br>• PMID: 20962343	Aim: To assess the value of the high RPL to detect the Type I Brugada ECG pattern in patients suspected of carrying BrS.<br>Study type: Observational<br>Size: 183	Inclusion criteria: Ajmaline testing using 15-lead ECGs was performed in 183 patients suspected of carrying BrS.	Of the 183 tests, 31 (17%) were positive, and 152 were negative. In all positive studies, at least 1 high RPL became positive. In 13/31 (42%) cases, the Type I ECG pattern could be observed only in the high RPLs. Standard or high V3 were never positive before standard or high V1-V2. In 7 patients, a Type I pattern was seen in 1 standard and 1 high RPL (vertical relationship).	The high RPLs are more sensitive than the conventional 12-lead ECG alone and initial observations suggest that they remain specific for BrS, while standard and high lead V3 offer redundant data.	A vertical relationship of type 1 patterns may have a similar diagnostic value to that of a horizontal pair.
Papadakis et al. The diagnostic yield of Brugada syndrome after sudden death with normal autopsy.<br>• Year published: 2018<br>• PMID: 29544603	Aim: To assess the impact of systematic ajmaline provo cation testing using high RPLs on the diagnostic yield of BrS in a large cohort of SADS families.<br>Study type: Prospective study<br>Size: 303 SADS families, 911 relatives	303 SADS families (911 relatives) underwent evaluation with resting ECG using conventional and high RPLs, echocardiography, exercise, and 24-h ECG monitor.	An inherited cardiac disease was diagnosed in 128 (42%) families and 201 (22%) relatives. BrS was the most prevalent diagnosis (n = 85, 28% of families; n = 140, 15% of relatives). Ajmaline testing was required to unmask the BrS in 97% of diagnosed individuals.	At initial evaluation, 4 (0.4%) individuals showed a spontaneous type 1 Brugada pattern on the resting ECG, of which 2 were seen only on the high RPLs.	Systematic use of ajmaline testing with high RPLs increases substantially the yield of BrS in SADS families.
Miyamoto et al. Diagnostic and prognostic value of a type 1 Brugada electrocardiogram at higher (third or second) V1 to V2 recording in men with Brugada syndrome.<br>• Year published: 2007<br>• PMID: 17196462	Aim: To evaluate the diagnostic and prognostic value of an ECG recorded at a higher (third or second) ICS.<br>Size: 98	Inclusion criteria: 98 men (17 to 76 years of age, mean [SD] 47 [13]; with documented VF in 22 and syncope in 32) were categorized into 3 groups; 68 men had a spontaneous type 1 ECG in standard leads V(1) and V(2) (S group), 19 had a spontaneous type 1 ECG only in the higher V(1) and V(2)	There were no significant differences in baseline clinical characteristics, including VF episodes, syncope, atrial fibrillation, family history, late potentials, and inducibility of VF during electrophysiologic study across the 3 groups. During prospective follow-up periods (779 ± 525, 442 ± 282, and 573 ± 382 days, respectively), subsequent cardiac events occurred in 11 men (16%) within the S group, in 2 men (11%) in the H group, and in 0	In men with previous episodes of VF, subsequent cardiac events occurred in 7 (44%) within the S group and in 2 (50%) in the H group (*p* = NS).	Men with a spontaneous type 1 Brugada ECG recorded only at higher leads V(1) and V(2) showed a prognosis similar to that of men with a type 1 ECG in using standard leads V(1) and V(2).

**Study name or acronym; Author; Year published; PMID**	**Aim of study; Endpoints; Study type (RCT, observational — multicenter or single center; case series or other [if RCT include intervention and comparator]); Study size (N)**	**Patient population with inclusion and exclusion criteria**	**Results (absolute event rates, *p* values; OR or RR; 95% CI)**	**Other relevant findings or adverse events**	**Limitations; Other comments; Conclusions**
		leads (H group), and 11 had a type 1 ECG only after receiving class Ic sodium channel blockers (Ic group).	men (0%) in the Ic group (*p* = NS, S vs H group).		
Savastano et al. A comprehensive electrocardiographic, molecular, and echocardiographic study of Brugada syndrome: validation of the 2013 diagnostic criteria.<br>• Year published: 2014<br>• PMID: 24721456	Aim: To test the validity, and the underlying anatomy, of the new ECG diagnostic criteria using echocardiographic, molecular, and clinical evidence in 1 clinical study population with BrS.<br>Size: 114	Inclusion criteria: Patients with BrS and with a spontaneous or drug-induced type 1 ECG pattern recorded in 1 or more RPLs in fourth, third, and second ICSs	The percentage of mutation carriers and the event rate were similar regardless of the diagnostic ICS (fourth vs high ICSs: mutation carriers 23% vs 19%; event rate 22% vs 28%) and the number of diagnostic leads (1 vs ≥2: mutation carriers 20% vs 22%; event rate 22% vs 27%). The concordance between RVOT anatomical location and the diagnostic ICSs was 86%.	The percentage of the diagnostic ECG pattern recorded was significantly increased by the exploration of the ICSs showing RVOT by echocardiography (echocardiography-guided approach vs conventional approach 100% vs 43%; *p* < .001).	The high ICSs are not inferior to the standard fourth ICS for the ECG diagnosis of BrS, and the interindividual variability depends on the anatomical location of the RVOT as assessed by using echocardiography. This approach significantly increases diagnostic sensitivity without decreasing specificity and fully supports the recently published new diagnostic criteria.
Kamath et al. Value of the signal-averaged electrocardiogram in arrhythmogenic right ventricular cardiomyopathy/ dysplasia.<br>• Year published: 2011<br>• PMID: 20933608	Aim: To examine the diagnostic and clinical value of the SAECG in a large population of genotyped ARVC/D probands.<br>Size: 87	Inclusion criteria: SAECGs of 87 ARVC/D probands (age 37 ± 13 years, 47 males) diagnosed as affected or borderline by task force criteria without using the SAECG criterion were compared with 103 control subjects.	Compared with controls, all 3 components of the SAECG were highly associated with the diagnosis of ARVC/D (*p* < .001). They include the filtered QRS duration (97.8 ± 8.7 ms vs 119.6 ± 23.8 ms), low-amplitude signal (24.4 ± 9.2 ms vs 46.2 ± 23.7 ms), and root mean square amplitude of the last 40 ms of the QRS (50.4 ± 26.9 µV vs 27.9 ± 36.3 µV). The sensitivity of using SAECG for diagnosis of ARVC/D was increased from 47% using the established 2 of 3 criteria (ie, late potentials) to 69% by using a modified criterion of any 1 of 3 criteria, while maintaining a high specificity of 95%.	Abnormal SAECG as defined by this modified criterion was associated with a dilated RV volume and decreased RV ejection fraction detected by CMR (*p* < .05). SAECG abnormalities did not vary with clinical presentation or reliably predict spontaneous or inducible VT and had limited correlation with ECG findings.	Using 1 of 3 SAECG criteria contributed to increased sensitivity and specificity for the diagnosis of ARVC/D. This finding is incorporated in the recent modification of the task force criteria.

**Study name or acronym; Author; Year published; PMID**	**Aim of study; Endpoints; Study type (RCT, observational — multicenter or single center; case series or other [if RCT include intervention and comparator]); Study size (N)**	**Patient population with inclusion and exclusion criteria**	**Results (absolute event rates, *p* values; OR or RR; 95% CI)**	**Other relevant findings or adverse events**	**Limitations; Other comments; Conclusions**
White et al. Utility of cardiovascular magnetic resonance in identifying substrate for malignant ventricular arrhythmias.<br>• Year published: 2012<br>• PMID: 22038987	Aim: To evaluate the diagnostic yield of CMR-based imaging versus non-CMR-based imaging in patients with resuscitated SCD or SMVT.<br>Size: 82	Inclusion criteria: 82 patients with resuscitated SCD or SMVT underwent routine non-CMR imaging, followed by a CMR protocol with comprehensive tissue characterization.	Relevant myocardial disease was identified in 51% of patients using non-CMR-based imaging and in 74% using CMR-based imaging (p = .002). 41 patients (50%) were reassigned to a new or alternate diagnosis using CMR-based imaging, including 15 (18%) with unsuspected acute myocardial injury. 20 patients (24%) had no abnormality by non-CMR imaging but showed clinically relevant myocardial disease by CMR imaging.	CMR-based imaging provides a robust diagnostic yield in patients presenting with resuscitated SCD or SMVT and incrementally identifies clinically unsuspected acute myocardial injury.	When compared with non-CMR-based imaging, a new or alternate myocardial disease process may be identified in half of these patients.
Rodrigues et al. Diagnosis and prognosis in sudden cardiac arrest survivors without coronary artery disease: utility of a clinical approach using cardiac magnetic resonance imaging.<br>• Year published: 2017<br>• PMID: 29237609	Aim: Role of CMR in SCA pathogenesis and prognosis in survivors.<br>Endpoints: Occurrence of MACE, defined as a composite of significant nonfatal and death.<br>Study type: Retrospective<br>Size: 164	Inclusion criteria: Arrest or periarrest scenario<br>Exclusion criteria: Coronary obstruction >30%	CMR was crucial for the main final clinical diagnosis in 50 cases (30%), while it gave an important contribution to all the other cases where it was diagnostic.	Arrhythmic causes were found in 14%, while no cause was identified in 36%. MACE occurred in 31% of subjects during a median follow-up of 32 months. MACE associated with presence of a CMR diagnosis, extent of LGE, and left and right ventricular ejection fractions.	Causality could only be hypothesized; patients diagnosed with a structural or functional cardiac abnormality by CMR had a 2-fold increased risk. LGE and biventricular systolic function were associated with prognosis, with RVEF being an independent predictor.
Eckart et al. Sudden death in young adults: a 25-year review of autopsies in military recruits.<br>• Year published: 2004	Aim: To determine the causes of nontraumatic sudden death among a cohort of military recruits.<br>Size: 126	Inclusion criteria: Of 126 nontraumatic sudden deaths (rate, 13.0/100,000 recruit-years), 108 (86%) were related to exercise. The most common cause of sudden death was an	The predominant structural cardiac abnormalities were coronary artery abnormalities (39 of 64 recruits [61%]), myocarditis (13 of 64 recruits [20%]), and HCM (8 of 64 recruits [13%]). An anomalous coronary artery accounted for one-third (21 of 64 recruits) of the cases in this cohort,	This cohort underwent a pre-enlistment screening program that included history and physical examination; this	Cardiac abnormalities are the leading identifiable cause of sudden death among military recruits; however, more than one-third of sudden deaths remain unexplained after detailed medical investigation.

Study name or acronym; Author; Year published; PMID	Aim of study; Endpoints; Study type (RCT, observational — multicenter or single center; case series or other [if RCT include intervention and comparator]); Study size (N)	Patient population with inclusion and exclusion criteria	Results (absolute event rates, *p* values; OR or RR; 95% CI)	Other relevant findings or adverse events	Limitations; Other comments; Conclusions
• PMID: 15583223		identifiable cardiac abnormality (64 of 126 recruits [51%]); however, a substantial number of deaths remained unexplained (44 of 126 recruits [35%]).	and, in each, the left coronary artery arose from the right (anterior) sinus of Valsalva, coursing between the pulmonary artery and aorta.	may have altered outcomes.	
**Investigation of Sudden Cardiac Arrest Survivors: Provocative Testing**					
Perrin et al. Exercise testing in asymptomatic gene carriers exposes a latent electrical substrate of arrhythmogenic right ventricular cardiomyopathy.<br>• Year published: 2013<br>• PMID: 23810883	Aim: To determine if exercise testing could expose a latent electrical substrate of ARVC in asymptomatic gene carriers.<br>Endpoints: Exercise-induced abnormalities during ETT<br>Study type: Observational study<br>Size: 85	Inclusion criteria: 30 asymptomatic ARVC gene carriers and 30 healthy controls. 25 patients with ARVC with histories of sustained ventricular arrhythmia or cardiac arrest	Depolarization abnormalities during ETT were found to develop more frequently in asymptomatic gene carriers compared with healthy controls: epsilon waves appeared in 4 of 28 (14%) compared with 0 of 30 (0%) (*p* = .048), premature ventricular contractions in 17 of 30 (57%) compared with 3 of 30 (10%) (*p* = .0003), and new QRS terminal activation duration >/= 55 ms in 7 of 22 (32%) compared with 2 of 29 (7%) (*p* = .03). Superior axis premature ventricular contractions occurred only in gene carriers.	In the second phase of the study, the frequency of these abnormalities was found to be high in patients with symptomatic ARVC: new epsilon waves appeared in 3 of 18 (17%), superior axis premature ventricular contractions in 21 of 25 (84%), and new terminal activation duration >/= 55 ms in 8 of 12 (67%).	Exercise testing exposes a latent electrical substrate in asymptomatic ARVC gene carriers that is shared by patients with ARVC with histories of ventricular arrhythmia. ETT may be useful in guiding treatment decisions, exercise prescription, and prioritizing medical surveillance in asymptomatic ARVC gene carriers.
Amin et al. Exercise-induced ECG changes in Brugada syndrome.<br>• Year published: 2009	Aim: Experimental studies suggest that BrS-linked SCN5A mutations reduce sodium current more at fast heart rates. Yet, the effects of exercise on the BrS ECG	Inclusion criteria: 35 male control subjects, 25 BrS men without SCN5A mutation (BrS(SCN5A)(-)), and 25 BrS men with SCN5A mutation (BrS(SCN5A+).	No differences existed in clinical phenotype between BrS groups. At baseline, BrS(SCN5A)(-) and BrS(SCN5A+) patients had lower heart rates, wider QRS, shorter QT(c), and higher peak J-point amplitudes than control subjects; BrS(SCN5A+) patients	25 BrS men with SCN5A mutation (BrS(SCN5A+); 15 with missense mutation and 10 with mutation leading to	Exercise aggravated the ECG phenotype in BrS. The presence of an SCN5A mutation was associated with further conduction slowing at fast heart rates. Possible mechanisms that

Study name or acronym; Author; Year published; PMID	Aim of study; Endpoints; Study type (RCT, observational — multicenter or single center; case series or other [if RCT include intervention and comparator]); Study size (N)	Patient population with inclusion and exclusion criteria	Results (absolute event rates, *p* values; OR or RR; 95% CI)	Other relevant findings or adverse events	Limitations; Other comments; Conclusions
• PMID: 19843921	phenotype have not been studied.<br>Endpoints: Exercise-induced ECG changes<br>Study type: Observational study<br>Size: 85		also had longer PR than BrS(SCN5A)(-) and control subjects. Exercise resulted in PR shortening in all groups, more QRS widening in BrS(SCN5A+) than in BrS(SCN5A)(-) and control subjects(,) and less QT shortening in BrS(SCN5A)(-) and BrS(SCN5A+) than in control subjects. The latter resulted in QT(c) shortening in control subjects but QT(c) prolongation in BrS(SCN5A)(-) and BrS(SCN5A+). Finally, the increase in peak J-point amplitude during exercise was similar in all 3 groups but resulted in a coved-type pattern only in BrS(SCN5A)(-) and BrS(SCN5A+).	premature truncation of the protein	may explain the observed ECG changes are discussed.
Priori et al. Clinical and molecular characterization of patients with catecholaminergic polymorphic ventricular tachycardia.<br>• Year published: 2002<br>• PMID: 12093772	Aim: To investigate the proportion of patients with CPVT carrying RyR2 mutations is unknown, and the clinical features of RyR2-CPVT as compared with nongenotyped CPVT are undefined.<br>Study type: Observational<br>Size: 30 probands and of 118 family members	Inclusion criteria: Patients with documented polymorphic ventricular arrhythmias occurring during physical or emotional stress with a normal heart entered the study.	Arrhythmias documented in probands were: 14 of 30 bidirectional VT, 12 of 30 polymorphic VT, and 4 of 30 catecholaminergic IVF; RyR2 mutations were identified in 14 of 30 probands (36% bidirectional VT, 58% polymorphic VT, 50% catecholaminergic IVF) and in 9 family members (4 silent gene carriers).	Genotype-phenotype analysis showed that patients with RyR2 CPVT have events at a younger age than do patients with nongenotyped CPVT and that male sex is a risk factor for syncope in RyR2-CPVT (relative risk = 4.2).	CPVT is a clinically and genetically heterogeneous disease manifesting beyond pediatric age with a spectrum of polymorphic arrhythmias. Beta-blockers reduce arrhythmias, but in 30% of patients an implantable defibrillator may be required. These data provide a rationale for prompt evaluation and treatment of young men with RyR2 mutations.
Krahn et al. Systematic assessment of patients with unexplained cardiac arrest: Cardiac Arrest	Aim: To unmask cause of ACA with drugs and imaging.<br>Study type: Prospective analysis<br>Size: 63 patients	Inclusion criteria: UCA without evident cardiac disease	Diagnosis was obtained in 56% including LQTS, CPVT, BrS, early repolarization, ARVC, coronary spasm, myocarditis.	Systematic clinical testing, including drug provocation and advanced imaging, results in unmasking of the cause of apparently	This approach assists in directing genetic testing to diagnose genetically mediated arrhythmia syndromes, which results in successful family screening.

**Study name or acronym; Author; Year published; PMID**	**Aim of study; Endpoints; Study type (RCT, observational — multicenter or single center; case series or other [if RCT include intervention and comparator]); Study size (N)**	**Patient population with inclusion and exclusion criteria**	**Results (absolute event rates, *p* values; OR or RR; 95% CI)**	**Other relevant findings or adverse events**	**Limitations; Other comments; Conclusions**
Survivors with Preserved Ejection Fraction Registry (CASPER).<br>• Year published: 2009<br>• PMID: 19597050				UCA in >50% of patients.	
Foo et al. Unmasking latent preexcitation of a right-sided accessory pathway with intravenous adenosine after unexplained sudden cardiac arrest.<br>• Year published: 2020	Aim: To present the case of a 17-year-old male with a cardiac arrest due to VF, necessitating resuscitation with multiple shocks and adrenaline. He later reported recurrent palpitations with presyncope for a year but was otherwise well without comorbidities or family history of sudden cardiac death.	Investigations including transthoracic echocardiogram, cardiac MRI, and computed tomography showed a structurally normal heart with normal coronary origins. Initial review of ECGs in hospital were thought to be benign. There was no evidence of preexcitation or tachyarrhythmias on telemetry. Ajmaline challenge and exercise tolerance test did not show any evidence of BrS, CPVT, or LQTS.	An intravenous adenosine test (6 mg) revealed manifest preexcitation without PR shortening, suggestive of a right posterior AP with slow antegrade conduction through the pathway. In retrospect, subtle evidence of manifest preexcitation was discovered on two of more than 20 ECGs.	Intravenous adenosine is a simple test that can uncover latent preexcitation via an accessory pathway and is useful in the diagnostic workup of SCA survivors without an identifiable cause.	This case illustrates the utility of intravenous adenosine in uncovering latent preexcitation of a previously unrecognized right posterior AP in a patient resuscitated from unexplained SCA. Successful ablation of the AP negated the need for a lifelong implantable cardioverter-defibrillator. APs with latent preexcitation are typically located on the left free wall, due to the distance from the sinus node rendering the pathway “concealed” during sinus rhythm. In this patient, the AP was in the right posterior position, but there was longer local A-V time in sinus rhythm than with CS pacing, suggesting an oblique course through the AV groove. This would have contributed to the absence of manifest preexcitation in sinus rhythm despite being a right-sided AP. To the authors’ knowledge, this is the second reported case of latent

Study name or acronym; Author; Year published; PMID	Aim of study; Endpoints; Study type (RCT, observational — multicenter or single center; case series or other [if RCT include intervention and comparator]); Study size (N)	Patient population with inclusion and exclusion criteria	Results (absolute event rates, *p* values; OR or RR; 95% CI)	Other relevant findings or adverse events	Limitations; Other comments; Conclusions
					preexcitation in a typical right-sided AP (excluding nodo-ventricular APs), but the first reported case to have resulted in a cardiac arrest.
Sy et al. Derivation and validation of a simple exercise-based algorithm for prediction of genetic testing in relatives of LQTS probands.<br>• Year published: 2011<br>• PMID: 22042885	Aim: To assess the accuracy of a simple algorithm that incorporates resting and exercise ECG parameters for screening LQTS in asymptomatic relatives was evaluated, with genetic testing as the gold standard.<br>Endpoints: Accuracy of a simple algorithm<br>Study type: Prospective<br>Size: The derivation cohort consisted of 69 relatives (28 with LQT1, 20 with LQT2, and 21 noncarriers); the validation cohort (n = 152; 58 with LQT1, 61 with LQT2, and 33 noncarriers; QTc = 443 ± 47 ms).	Inclusion criteria: Asymptomatic first-degree relatives of genetically characterized probands	The derivation cohort consisted of 69 relatives (28 with LQT1, 20 with LQT2, and 21 noncarriers). Mean age was 35 ± 18 years, and resting corrected QT interval (QTc) was 466 ± 39 ms. Abnormal resting QTc (females >/=480 ms; males >/=470 ms) was 100% specific for gene carrier status, but was observed in only 48% of patients; however, mutations were observed in 68% and 42% of patients with a borderline or normal resting QTc, respectively. Among these patients, 4-min recovery QTc >/=445 ms correctly restratified 22 of 25 patients as having LQTS and 19 of 21 patients as being noncarriers. The combination of resting and 4-min recovery QTc in a screening algorithm yielded a sensitivity of 0.94 and specificity of 0.90 for detecting LQTS carriers.	When applied to the validation cohort (n = 152; 58 with LQT1, 61 with LQT2, and 33 noncarriers; QTc = 443 ± 47 ms), sensitivity was 0.92 and specificity was 0.82.	A simple algorithm that incorporates resting and exercise-recovery QTc is useful in identifying LQTS in asymptomatic relatives.
Wong et al. Utility of treadmill testing in identification and genotype prediction in long-QT syndrome.<br>• Year published: 2010<br>• PMID: 20071715	Aim: To develop a provocative testing strategy to unmask the LQTS phenotype and relate this to the results of genetic testing.<br>Study type: Retrospective<br>Size: 159 patients	Inclusion criteria: 159 consecutive patients with suspected LQTS underwent provocative testing	LQTS patients exhibited a greater prolongation in QTc with postural change than unaffected patients (LQT1: 40 ms [IQR, 42]; LQT2: 35 ms [IQR, 46]; and LQTS-negative: 21 ms [IQR, 37]; *p* = .029). During exercise, LQT1 patients had marked QTc prolongation compared with LQT2 and LQTS-negative patients (LQT1: 65 ms	QT hysteresis was more pronounced in patients with LQT2 mutations compared with LQT1 and LQT-negative patients (LQT2: 40 ms [10], LQT1: 15 ms [40];	The presence and genotype of LQTS can be predicted by a combination of postural and exercise changes in the QT/RR relationship. Beta-blockade normalized these changes. Routine exercise testing is useful in predicting and directing genetic testing in LQTS.

**Study name or acronym; Author; Year published; PMID**	**Aim of study; Endpoints; Study type (RCT, observational — multicenter or single center; case series or other [if RCT include intervention and comparator]); Study size (N)**	**Patient population with inclusion and exclusion criteria**	**Results (absolute event rates, *p* values; OR or RR; 95% CI)**	**Other relevant findings or adverse events**	**Limitations; Other comments; Conclusions**
			[60], LQT2: 3 ms [46], LQTS negative: 5 ms [41]; *p* < .0001).	LQTS-negative: 20 ms [20]; *p* < .001). Beta-blockade normalized the QTc changes seen with standing and QT hysteresis.	
Herman et al. Outcome of apparently unexplained cardiac arrest: results from investigation and follow-up of the prospective Cardiac Arrest Survivors with Preserved Ejection Fraction Registry.<br>• Year published: 2016<br>• PMID: 26783233	Aim: To investigate the usefulness of exercise testing, drug provocation, advanced cardiac imaging, and genetic testing.<br>Study type: Observational<br>Size: 200 survivors of UCA	Inclusion criteria: 200 survivors of UCA	Age, 48.6 ± 14.7 years, 41% female. Advanced testing determined a diagnosis in 34% of patients at baseline, with a diagnosis emerging during follow-up in 7% of patients. Of those who were diagnosed, 28 (35%) had an underlying structural condition and 53 (65%) had a primary electric disease. During a mean follow-up of 3.15 ± 2.34 years, 23% of patients had either a shock or an appropriate antitachycardia pacing from their ICD, or both.	The ICD appropriate intervention rate was 8.4% at 1 year and 18.1% at 3 years, with no clear difference between diagnosed and undiagnosed subjects, or between those diagnosed with a primary electric versus structural pathogenesis.	Obtaining a diagnosis in previously UCA patients requires systematic clinical testing and regular follow-up to unmask the cause. Nearly half of apparently UCA patients ultimately received a diagnosis, allowing for improved treatment and family screening. A substantial proportion of patients received appropriate ICD therapy during medium-term follow-up.
Horner et al. The diagnostic utility of recovery phase QTc during treadmill exercise stress testing in the evaluation of long QT syndrome.<br>• Year published: 2011<br>• PMID: 21699858	Aim: Nearly 40% of patients with LQTS can have a nondiagnostic QTc at rest. Treadmill and cycle EST are used in the diagnostic evaluation of LQTS.<br>Endpoints: The purpose of this study was to determine the diagnostic significance of peak exercise and recovery phase QTc values during	Inclusion criteria: 243 patients including 82 LQT1, 55 LQT2, 18 LQT3, and 88 genotype-negative patients dismissed as normal	Compared with those dismissed as normal, the average QTc was greater at all scored stages in LQT1 and LQT3 patients and at all stages in LQT2 patients except peak exercise and 1 minute of recovery (*p* < .01). Either an absolute QTc >/= 460 ms during the recovery phase or a maladaptive, paradoxical increase in QTc, defined as QTc recovery–QTc baseline >/= 30 ms (DeltaQTc), distinguished patients with	The presence of beta-blockers did not blunt these abnormal repolarization profiles.	Treadmill stress testing can unmask patients with concealed LQTS, particularly LQT1, with good diagnostic accuracy.

Study name or acronym; Author; Year published; PMID	Aim of study; Endpoints; Study type (RCT, observational — multicenter or single center; case series or other [if RCT include intervention and comparator]); Study size (N)	Patient population with inclusion and exclusion criteria	Results (absolute event rates, *p* values; OR or RR; 95% CI)	Other relevant findings or adverse events	Limitations; Other comments; Conclusions
	treadmill stress testing in LQTS<br>Study type: Observational<br>Size: 243		either manifest or concealed LQT1 from all other subsets (*p* < .0001).		
Giudicessi et al. Exercise testing oversights underlie missed and delayed diagnosis of catecholaminergic polymorphic ventricular tachycardia in young sudden cardiac arrest survivors.<br>• Year published: 2019<br>• PMID: 30763784	Aim: To determine the number of missed or delayed CPVT diagnoses attributable to exercise testing oversights in a cohort of young SCA survivors.<br>Study type: Observational study<br>Size: 101	Inclusion criteria: 101 young SCA survivors (younger than 35 years at the time of SCA) with otherwise structurally normal hearts was used to identify those with a missed or delayed CPVT diagnosis because of overlooked evidence or lack of an EST or CPT post-SCA	Of the 101 young SCA survivors, 41 (41%) had exertion/emotion-associated SCA (EEA-SCA). After primary post-SCA investigations, a probable root cause was established in 20 of 41 EEA-SCA survivors (49%; CPVT in 8) and in 30 of 60 non-EEA-SCA survivors (50%; CPVT in 2) (*p* = 1). Only 14 of 21 unexplained EEA-SCA survivors (67%) had an EST/CPT performed before their referral evaluation. Secondary review of these prior ESTs/CPTs provided evidence of CPVT in 3 of 14 (21%). Of the 7 remaining unexplained cases of EAA-SCA who had never undergone an EST/CPT, 2 (29%) underwent their first EST at our institution that led to CPVT diagnosis.	Of the 15 SCA survivors diagnosed ultimately with CPVT, one-third had a delay in diagnosis because an EST was either never performed or performed but misinterpreted.	EST/CPT must become the standard of care after SCA in the young, especially if the SCA occurred during either exertion or emotion.
Adler et al. The phenomenon of “QT stunning”: the abnormal QT prolongation provoked by standing persists even as the heart rate returns to normal in patients	Aim: To validate original observations in a cohort twice as large and to describe that the abnormal QT-interval response persists as the heart rate acceleration returns to baseline.<br>Study type: Observational<br>Size: 108 patients with LQTS and 112 healthy subjects	Inclusion criteria: 108 patients with LQTS and 112 healthy subjects	QTc(stretch) lengthened significantly more in patients with LQTS (103 ± 80 ms vs 66 ± 40 ms in controls; *p* < .001) and so did QTc(return) (28 ± 48 ms for patients with LQTS vs -3 ± 32 ms for controls; *p* < .001). Using a sensitivity cutoff of 90%, the specificity for diagnosing LQTS was 74% for QTc(base), 84% for QTc(return), and 87% for QTc(stretch).	The present study extends previous findings on the abnormal response of the QT interval in response to standing in patients with LQTS.	The study also shows that this abnormal response persists even after the heart rate slows back to baseline.

**Study name or acronym; Author; Year published; PMID**	**Aim of study; Endpoints; Study type (RCT, observational — multicenter or single center; case series or other [if RCT include intervention and comparator]); Study size (N)**	**Patient population with inclusion and exclusion criteria**	**Results (absolute event rates, *p* values; OR or RR; 95% CI)**	**Other relevant findings or adverse events**	**Limitations; Other comments; Conclusions**
with long QT syndrome.<br>• Year published: 2012<br>• PMID: 22300664					
Dionne et al. Dynamic QT interval changes from supine to standing in healthy children.<br>• Year published: 2018<br>• PMID: 29275885	Aim: A quick standing challenge has been proposed as an alternative provocative test in adults, with no pediatric data yet available.<br>Study type: Observational<br>Size: 100	Inclusion criteria: 100 healthy children	100 healthy children (mean age, 9.7 ± 3.1 years). On standing, the heart rate increased by 29 ± 10 bpm. The QT interval was similar at baseline and on standing (394 ± 34 ms vs 394 ± 34 ms; *p* = 1.0). However, QTc increased from 426 ± 21 to 509 ± 41 ms (*p* < .001). The 95th percentile for QTc at baseline and maximal heart rate was 457 ms and 563 ms, respectively. At 1 min of recovery, the QT interval was shorter (375 ± 31 ms) compared with baseline (394 ± 34 ms; *p* < .001) and standing (394 ± 34 ms; *p* < .001). QTc reached baseline values after 1 minute of recovery and remained stable thereafter (423 ± 23 ms at 1 min; 426 ± 22 ms at 5 min; *p* = 1.0).	This first characterization of QTc changes on standing in children shows substantial alterations, which are greater than those seen in adults.	Two-thirds of the children would have been misclassified as having LQTS by adult criteria, indicating the need to create child-specific standards.
Viskin et al. The response of the QT interval to the brief tachycardia provoked by standing: a bedside test for diagnosing long QT syndrome.<br>• Year published: 2010<br>• PMID: 20116193	Aim: To assess standing test for diagnosing LQTS.<br>Endpoints: QTc<br>Study type:<br>Size: 68 LQTS, 82 controls	Inclusion criteria:<br>68 with LQTS [LQT1 46%, LQT2 41%, LQT3 4%, not genotyped 9%] and 82 control subjects	On average, the QT interval of controls shortened by 21 ± 19 ms whereas the QT interval of LQTS patients increased by 4 ± 34 ms (*p* < .001). Since the RR interval shortened more than the QT interval, during maximal tachycardia the corrected QT interval increased by 50 ± 30 ms in the control group and by 89 ± 47 ms in the LQTS group (*p* < .001).	The response of the QT interval to brisk standing was particularly impaired in patients with LQT2.	Evaluation of the response of the QT interval to the brisk tachycardia induced by standing provides important information that aids in the diagnosis of LQTS.

Study name or acronym; Author; Year published; PMID	Aim of study; Endpoints; Study type (RCT, observational — multicenter or single center; case series or other [if RCT include intervention and comparator]); Study size (N)	Patient population with inclusion and exclusion criteria	Results (absolute event rates, *p* values; OR or RR; 95% CI)	Other relevant findings or adverse events	Limitations; Other comments; Conclusions
Filippini et al. The brisk-standing-test for long QT syndrome in prepubertal school children: defining normal.<br>• Year published: 2018<br>• PMID: 29036559	Aim: To present reference values for prepubertal children.<br>Study type: Observational<br>Size: 57	Inclusion criteria: Healthy, prepubertal children, aged 7-13 years underwent a standard supine resting ECG and during continuous ECG recording performed a brisk-standing test.	57 children, 29 boys (10.2 ± 1.1 years) and 28 girls (9.9 ± 1.1 years) were included. Baseline characteristics and response to standing were not statistically different for boys and girls: mean supine pre-standing heart rate 74 ± 9 vs. 77 ± 9 bpm, supine pre-standing QTc 406 ± 27 vs. 407 ± 17 ms, maximal heart rate upon standing 109 ± 11 vs. 112 ± 11 bpm, and QTc at maximal heart rate 484 ± 29 vs. 487 ± 35 ms.	The QT interval corrected for heart rate-prolongation at maximal tachycardia after standing was 79 ± 26 (19-144) ms, which is significantly longer than previously published values in adults (50 ± 30 ms).	The QT interval corrected for heart rate prolongation after brisk standing in healthy prepubertal children is more pronounced than in healthy adults. This finding advocates distinct prepubertal cut-off values because using adult values for prepubertal children would yield false positive results with the risk of incorrect LQTS-diagnosis and overtreatment.
Shimizu et al. Diagnostic value of epinephrine test for genotyping LQT1, LQT2, and LQT3 forms of congenital long QT syndrome.<br>• Year published: 2004<br>• PMID: 15851169	Aim: To test the hypothesis that epinephrine test may have diagnostic value for genotyping LQT1, LQT2, and LQT3 forms of congenital LQTS.<br>Study type: Retrospective and prospective, in blinded fashion to prospectively separate a different group<br>Size: Retrospective: 15 LQT1, 10 LQT2, 8 LQT3, and 10 healthy volunteers; prospective: 42 probands, 67 family members	Inclusion criteria: Retrospective: 15 LQT1, 10 LQT2, 8 LQT3, and 10 healthy volunteers; prospective: 42 probands, 67 family members, and 10 new volunteers	The sensitivity (penetrance) by ECG diagnostic criteria was lower in LQT1 (68%) than in LQT2 (83%) or LQT3 (83%) before epinephrine and was improved with steady-state epinephrine in LQT1 (87%) and LQT2 (91%) but not in LQT3 (83%), without the expense of specificity (100%). The sensitivity and specificity to differentiate LQT1 from LQT2 were 97% and 96%, those from LQT3 were 97% and 100%, and those from Control were 97% and 100%, respectively, when Delta mean corrected Q-Tend >/=35 ms at steady state was used. The sensitivity and specificity to differentiate LQT2 from LQT3 or Control were 100% and 100%, respectively, when Delta mean corrected Q-Tend >/=80 ms at peak was used.		Epinephrine infusion is a powerful test to predict the genotype of LQT1, LQT2, and LQT3 syndromes as well as to improve the clinical diagnosis of genotype-positive patients, especially those with LQT1 syndrome.

**Study name or acronym; Author; Year published; PMID**	**Aim of study; Endpoints; Study type (RCT, observational — multicenter or single center; case series or other [if RCT include intervention and comparator]); Study size (N)**	**Patient population with inclusion and exclusion criteria**	**Results (absolute event rates, *p* values; OR or RR; 95% CI)**	**Other relevant findings or adverse events**	**Limitations; Other comments; Conclusions**
Ackerman et al. Epinephrine-induced QT interval prolongation: a gene-specific paradoxical response in congenital long QT syndrome.<br>• Year published: 2002<br>• PMID: 12004990	Aim: To determine the effect of epinephrine on the QT interval in patients with genotyped LQTS.<br>Study type: Retrospective<br>Size: 37	Inclusion criteria:<br>37 patients (24 females) with genotyped LQTS (19 LQT1, 15 LQT2, 3 LQT3, mean age, 27 years; range, 10-53 years) from 21 different kindreds and 27 (16 females) controls (mean age, 31 years; range, 13-45 years)	The mean ± SD baseline QTc was greater in LQTS patients (500 ± 68 ms) than in controls (436 ± 19 ms, *p* < .001). However, 9 (47%) of 19 KVLQT1-genotyped LQT1 patients had a nondiagnostic resting QTc (<460 ms), whereas 11 (41%) of 27 controls had a resting QTc higher than 440 ms. During epinephrine infusion, every LQT1 patient manifested prolongation of the QT interval (paradoxical response), whereas healthy controls and patients with either LQT2 or LQT3 tended to have shortened QT intervals (*p* < .001). The maximum mean ± SD change in QT (AQT [epinephrine QT minus baseline QT]) was -5 ± 47 ms (controls), +94 ± 31 ms (LQT1), and -87 ± 67 ms (LQT2 and LQT3 patients).	Of 27 controls, 6 had lengthening of their QT intervals (AQT >30 milliseconds) during high-dose epinephrine. Low-dose epinephrine (0.05 microg x kg(-1) x min(-1)) completely discriminated LQT1 patients (AQT, +82 ± 34 ms) from controls (AQT, -7 ± 13 ms; *p* < .001).	Epinephrine-induced prolongation of the QT interval appears pathognomonic for LQT1. Low-dose epinephrine infusion distinguishes controls from patients with concealed LQT1 manifesting an equivocal QTc at rest. Thus, epinephrine provocation may help unmask some patients with concealed LQTS and strategically direct molecular genetic testing.
Krahn et al. Epinephrine infusion in the evaluation of unexplained cardiac arrest and familial sudden death: from the Cardiac Arrest Survivors with Preserved Ejection Fraction Registry.<br>• Year published: 2012<br>• PMID: 22944906	Aim: To assess epinephrine infusion may unmask latent genetic conditions associated with cardiac arrest, including LQTS and CPVT.<br>Study type: Prospective observational<br>Size: 170	Inclusion criteria:<br>Patients with UCA (normal left ventricular function and QT interval) and selected family members from the Cardiac Arrest Survivors with Preserved Ejection Fraction Registry	Epinephrine infusion was performed in 170 patients (age, 42 ± 16 years; 49% men), including 98 patients with UCA. Testing was positive for LQTS in 31 patients (18%) and borderline in 24 patients (14%). Exercise testing provoked an abnormal QT response in 42% of tested patients with a positive epinephrine response. Testing for CPVT was positive in 7% and borderline in 5%.	Targeted genetic testing of abnormal patients was positive in 17% of LQTS patients and 13% of CPVT patients.	Epinephrine challenge provoked abnormalities in a substantial proportion of patients, most commonly a prolonged QT interval. Exercise and genetic testing replicated the diagnosis suggested by the epinephrine response in a small proportion of patients. Epinephrine infusion combined with exercise testing and targeted genetic testing is recommended in the workup of suspected familial sudden death syndromes.

**Study name or acronym; Author; Year published; PMID**	**Aim of study; Endpoints; Study type (RCT, observational — multicenter or single center; case series or other [if RCT include intervention and comparator]); Study size (N)**	**Patient population with inclusion and exclusion criteria**	**Results (absolute event rates, *p* values; OR or RR; 95% CI)**	**Other relevant findings or adverse events**	**Limitations; Other comments; Conclusions**
Marjamaa et al. Intravenous epinephrine infusion test in diagnosis of catecholaminergic polymorphic ventricular tachycardia.<br>• Year published: 2012<br>• PMID: 21954897	Aim: To estimate the predictive value of intravenous epinephrine administration in CPVT patients with frequent exercise-induced ventricular ectopy.<br>Study type: Observational<br>Size: 81	Inclusion criteria: 81 subjects, including 25 CPVT-linked ryanodine receptor 2 (RYR2) mutation carriers, 11 genetically undefined CPVT patients, and 45 unaffected family members	All subjects underwent a maximal EST and an intravenous epinephrine infusion test. EST was positive in 25 (31%) patients including 14 of 25 (56%) established RYR2-mutation carriers and all 11 (100%) genetically undefined CPVT patients. Epinephrine infusion induced arrhythmias in 3 (12%) RYR2-mutation carriers, 4 (36%) genetically undefined CPVT patients, and 1 (2%) unaffected family member. A total of 18 EST positive patients did not respond to intravenous epinephrine administration, whereas only 1 epinephrine test responder had a normal EST.	If EST is used as a standard, the sensitivity of the epinephrine infusion test is 28% and specificity is 98%.	Intravenous epinephrine infusion has low sensitivity and may not be considered as an alternative method for a maximal EST in diagnosis of CPVT.
Vyas et al. Epinephrine QT stress testing in the evaluation of congenital long-QT syndrome: diagnostic accuracy of the paradoxical QT response.<br>• Year published: 2006<br>• PMID: 16534005	Aim: A paradoxical increase in the uncorrected QT interval during infusion of low-dose epinephrine appears pathognomonic for type 1 LQTS (LQT1). To determine the diagnostic accuracy of this response among patients referred for clinical evaluation of congenital LQTS.<br>Study type: Observational<br>Size: 147	Inclusion criteria: The 125 untreated patients (44 genotype negative, 40 LQT1, 30 LQT2, and 11 LQT3) constituted the primary analysis.	The median baseline corrected QT intervals (QTc) were 444 ms (gene negative), 456 ms (LQT1), 486 ms (LQT2), and 473 ms (LQT3). The median change in QT interval during low-dose epinephrine infusion was -23 ms in the gene-negative group, 78 ms in LQT1, -4 ms in LQT2, and -58 ms in LQT3. The paradoxical QT response was observed in 37 (92%) of 40 patients with LQT1 compared with 18% (gene-negative), 13% (LQT2), and 0% (LQT3; $p < .0001$) of the remaining patients.	Overall, the paradoxical QT response had a sensitivity of 92.5%, specificity of 86%, positive predictive value of 76%, and negative predictive value of 96% for LQT1 status.	The epinephrine QT stress test can unmask concealed type 1 LQTS with a high level of accuracy.
Govindan et al. Utility of high and standard right precordial leads during ajmaline	Aim: To assess the value of the high RPL to detect the Type I Brugada ECG pattern in patients suspected of carrying BrS.	Inclusion criteria: Ajmaline testing using 15-lead ECGs was performed in 183	Of the 183 tests, 31 (17%) were positive, and 152 were negative. In all positive studies, at least 1 high RPL became positive. In 13/31 (42%) cases, the Type I ECG pattern could be	The high RPLs are more sensitive than the conventional 12-lead ECG alone and initial	A vertical relationship of type 1 patterns may have a similar diagnostic value to that of a horizontal pair.

**Study name or acronym; Author; Year published; PMID**	**Aim of study; Endpoints; Study type (RCT, observational — multicenter or single center; case series or other [if RCT include intervention and comparator]); Study size (N)**	**Patient population with inclusion and exclusion criteria**	**Results (absolute event rates, *p* values; OR or RR; 95% CI)**	**Other relevant findings or adverse events**	**Limitations; Other comments; Conclusions**
testing for the diagnosis of Brugada syndrome.<br>• Year published: 2010<br>• PMID: 20962343	Study type: Observational<br>Size: 183	patients suspected of carrying BrS.	observed only in the high RPLs. Standard or high V3 were never positive before standard or high V1-V2. In 7 patients, a Type I pattern was seen in 1 standard and 1 high RPL (vertical relationship).	observations suggest that they remain specific for BrS, while standard and high lead V3 offer redundant data.	
Hasdemir et al. High prevalence of concealed Brugada syndrome in patients with atrioventricular nodal reentrant tachycardia.<br>• Year published: 2015<br>• PMID: 25998140	Aim: To determine the prevalence of drug-induced type 1 Brugada ECG pattern (concealed BrS) in patients presenting with clinical spontaneous AVNRT and to investigate their electrocardiographic, electrophysiological, and genetic characteristics.<br>Study type: Observational<br>Size: 96 patients, 66 controls	Inclusion criteria: 96 consecutive patients without any sign of BrS on baseline ECG undergoing electrophysiological study and ablation for symptomatic, drug-resistant AVNRT and 66 control subjects underwent an ajmaline challenge to unmask BrS.	A concealed BrS ECG was uncovered in 26 of 96 patients with AVNRT (27.1%) and in 3 of 66 control subjects (4.5%). Patients with concealed BrS were predominantly female patients (n = 23 [88.5%] vs n = 44 [62.9%], *p* = .015), had higher prevalence of chest pain (n = 10 [38.5%] vs n = 13 [18.6%], *p* = .042), migraine headaches (n = 10 [38.5%] vs n = 10 [14.2%], *p* = .008), and drug-induced initiation and/or worsening of duration and/or frequency of AVNRT (n = 4 [15.4%] vs n = 1 [1.4%], *p* = .006) as compared to patients with AVNRT without BrS.	Genetic screening identified 19 mutations or rare variants in 13 genes in 13 of 17 patients with both AVNRT and BrS (yield = 76.5%). Ten of these 13 genotype-positive patients (76.9%) harbored genetic variants known or suspected to cause a loss of function of cardiac sodium channel current (SCN5A, SCN10A, SCN1B, GPD1L, PKP2, and HEY2).	Spontaneous AVNRT and concealed BrS co-occur, particularly in female patients, and genetic variants that reduce sodium channel current may provide a mechanistic link between AVNRT and BrS and predispose to expression of both phenotypes.
Meregalli et al. Diagnostic value of flecainide testing in unmasking SCN5A-related Brugada syndrome.	Aim: To establish sensitivity, specificity, and safety of flecainide testing.<br>Endpoints: Sensitivity, specificity, and safety of flecainide testing<br>Study type: Prospective	Inclusion criteria: Subjects determined to be at risk for BrS	The sensitivity and specificity, calculated in SCN5A-positive probands and their family members, were 77% and 80%, respectively.	No malignant arrhythmias were observed.<br>An attempt to predict the outcome of flecainide testing from these baseline	Flecainide testing is a valid and safe tool to identify SCN5A-related BrS patients. Baseline ECGs do not predict test outcomes, but point to conduction slowing as a core mechanism in BrS.

**Study name or acronym; Author; Year published; PMID**	**Aim of study; Endpoints; Study type (RCT, observational — multicenter or single center; case series or other [if RCT include intervention and comparator]); Study size (N)**	**Patient population with inclusion and exclusion criteria**	**Results (absolute event rates, *p* values; OR or RR; 95% CI)**	**Other relevant findings or adverse events**	**Limitations; Other comments; Conclusions**
• Year published: 2006<br>• PMID: 16764707	analysis<br>Size: 160 tests			ECG parameters failed.	
Hong et al. Value of electrocardiographic parameters and ajmaline test in the diagnosis of Brugada syndrome caused by SCN5A mutations.<br>• Year published: 2004<br>• PMID: 15520322	Aim: Sodium channel blockers are effective in unmasking carriers of the BrS. However, the value of the test remains controversial.<br>Size: 147	Inclusion criteria: 147 individuals representing 4 large families with SCN5A mutations were studied. Of these, 104 were determined to be at possible risk for BrS and underwent both electrocardiographic and genetic evaluation. SCN5A mutations	24 individuals displayed an ECG diagnostic of BrS at baseline. Of the remaining, 71 received intravenous ajmaline. Of the 35 genetic carriers who received ajmaline, 28 had a positive test and 7 a negative ajmaline test. The sensitivity, specificity, and positive and negative predictive values of the drug challenge were 80% (28:35), 94.4% (34:36), 93.3% (28:30), and 82.9% (34:41), respectively.	Penetrance of the disease phenotype increased from 32.7% to 78.6% with the use of sodium channel blockers. In the absence of ST-segment elevation under baseline conditions, a prolonged P-R interval, but not incomplete right bundle-branch block or early repolarization patterns, indicates a high probability of an SCN5A mutation carrier.	In families with BrS, the data suggest that ajmaline testing is valuable in the diagnosis of SCN5A carriers. In the absence of ST-segment elevation at baseline, family members with first-degree AV block should be suspected of carrying the mutation. An ajmaline test is often the key to making the proper diagnosis in these patients.
Brugada et al. Sodium channel blockers identify risk for sudden death in patients with ST-segment elevation and right bundle branch block but structurally normal hearts.	Aim: To examine arrhythmic risk in patients with overt and concealed forms of the disease and the effectiveness of sodium channel blockers to unmask the syndrome and, thus, identify patients at risk.<br>Size: 34	Inclusion criteria:<br>34 patients with the syndrome and transient normalization of the ECG (group A), 11 members of 3 families in whom a SCN5A mutation was associated with the syndrome and 8 members in whom it was not (group B), and	Ajmaline, procainamide, or flecainide administration resulted in ST-segment elevation and right bundle branch block in all patients in group A and in all 11 patients with the mutation in group B. A similar pattern could not be elicited in the 8 patients in group B who lacked the mutation or in any person in group C.	The follow-up period (37 ± 33 months) revealed no differences in the incidence of arrhythmia between the 34 patients in whom the phenotypic manifestation of the syndrome was transient and the 24	The data demonstrated a similar incidence of potentially lethal arrhythmias in patients displaying transient versus persistent ST-segment elevation and right bundle branch block, as well as the effectiveness of sodium channel blockers to unmask the syndrome and, thus, identify patients at risk.

**Study name or acronym; Author; Year published; PMID**	**Aim of study; Endpoints; Study type (RCT, observational — multicenter or single center; case series or other [if RCT include intervention and comparator]); Study size (N)**	**Patient population with inclusion and exclusion criteria**	**Results (absolute event rates, *p* values; OR or RR; 95% CI)**	**Other relevant findings or adverse events**	**Limitations; Other comments; Conclusions**
• Year published: 2000<br>• PMID: 10662748		53 control subjects (group C).		patients in whom it was persistent (log-rank, 0.639).	
Krahn et al. Diagnosis of unexplained cardiac arrest: role of adrenaline and procainamide infusion.<br>• Year published: 2005<br>• PMID: 16203906	Aim: To unmask latent primary electrical disease.<br>Study type: Prospective observational<br>Size: 18 probands with SCD; 55 family members	Inclusion criteria:<br>18 patients (mean ± SD age, 41 ± 17 years; 11 female) with UCA were assessed.	The final diagnosis was CPVT in 10 patients (56%), BrS in 2 patients (11%), and unexplained (IVF) in 6 patients (33%). Of 55 family members (mean ± SD age, 27 ± 17 years; 33 female), 9 additional affected family members were detected from 2 families, with a single BrS patient and 8 CPVT patients.	Provocative testing with adrenaline and procainamide infusions is useful in unmasking the etiology of apparent UCA.	This approach helps to diagnose primary electrical disease, such as CPVT and BrS, and provides the opportunity for therapeutic intervention in identified, asymptomatic family members who harbor the same disease.
Waldmann et al. Coronary vasospasm-related sudden cardiac arrest in the community.<br>• Year published: 2018<br>• PMID: 30092958	Aim: To assess the extent to which CVS-related SCA is investigated and managed in the real-world setting.<br>Study type: Multicenter, prospectively observational<br>Size: 3,028 SCA patients	Inclusion criteria: 1,557 patients with a definite cardiac etiology, 31 SCA were diagnosed as related CVS	Among the 1,557 patients with a definite cardiac etiology, 31 SCA were diagnosed as related to CVS, representing 2.0% of cardiac causes. The diagnosis of CVS was made by typical major spontaneous spasm during initial coronary angiography in 16 (51.6%) cases, by provocative test with ergonovine in 12 (38.7%), and ST segment elevation accompanied by chest pain during ECG monitoring with initial normal angiography in 3 (9.7%) patients.	CVS provocative test was performed in only 63.9% of apparently unexplained SCA patients, with a diagnostic yield of 30.8%.	CVS as a cause of SCA is under-investigated in the general population and ICD therapy remains poorly utilized.
Nakao et al. Hyperventilation as a specific test for diagnosis of coronary artery spasm.<br>• Year published: 1997	Aim: To establish the sensitivity and specificity of the hyperventilation test and to clarify the characteristics of hyperventilation test-positive patients.<br>Study type: Observational	Inclusion criteria: 206 patients in whom coronary spasm was documented by angiography (spasm group), and 183 patients without angina at rest in whom	Of the spasm group patients, 127 showed positive responses to the test, including ST elevation (n = 111), ST depression (n = 15) and negative U wave (n = 1). None in the nonspasm group showed any ischemic electrocardiographic change. Thus, the sensitivity and specificity of this test		The findings imply that hyperventilation is a highly specific test for the diagnosis of coronary artery spasm, and that hyperventilation test-positive patients are likely to have life-threatening arrhythmias during attacks and multivessel spasm.

Study name or acronym; Author; Year published; PMID	Aim of study; Endpoints; Study type (RCT, observational — multicenter or single center; case series or other [if RCT include intervention and comparator]); Study size (N)	Patient population with inclusion and exclusion criteria	Results (absolute event rates, *p* values; OR or RR; 95% CI)	Other relevant findings or adverse events	Limitations; Other comments; Conclusions
• PMID: 9294979	Size: 206	acetylcholine failed to induce spasm (nonspasm group)	for diagnosis of coronary spasm were 62% and 100%, respectively.		
Wang et al. Patients with supraventricular tachycardia presenting with aborted sudden death: incidence, mechanism and long-term follow-up.<br>• Year published: 1991<br>• PMID: 1960318	Aim: To investigate the incidence of supraventricular arrhythmia responsible for SCD and the results of long-term follow-up of surviving patients.<br>Endpoints: Incidence of supraventricular arrhythmia responsible for SCD and the results of long-term follow-up of surviving patients<br>Study type: Observational<br>Size: 290	Inclusion criteria: 13 (4.5%) of 290 patients with aborted sudden death	A total of 13 (4.5%) of 290 patients with aborted sudden death had either documented (7; 54%) or strong presumptive evidence of supraventricular tachycardia that deteriorated into VF. Six (46%) of the 13 had an accessory conduction pathway and either atrial fibrillation (5 patients) or paroxysmal AV reentrant tachycardia (1 patient) that deteriorated into VF. Three patients with AV node reentrant tachycardia and 4 with atrial fibrillation and enhanced AV node conduction presented with supraventricular arrhythmias that deteriorated into VF.	Over a follow-up period of 41.6 ± 33.6 months, 12 patients are alive without symptomatic arrhythmias.	Treatment directed at prevention of supraventricular tachycardia was associated with an excellent prognosis. Current treatment techniques appear to obviate the need for automatic defibrillator therapy in these patients.
Roberts et al. Bundle branch re-entrant ventricular tachycardia: novel genetic mechanisms in a life-threatening arrhythmia.<br>• Year published: 2017<br>• PMID: 29759522	Aim: To investigate for an underlying genetic etiology in cases of apparent idiopathic BBRVT.<br>Size: 6	Inclusion criteria: Enrollment required a clinically documented wide complex tachycardia and BBRVT proven during invasive electrophysiology study.	Among 6 cases of idiopathic BBRVT, each presented with hemodynamic compromise and 2 suffered cardiac arrests requiring resuscitation. Putative culprit mutations were identified in 3 of 6 cases, including 2 in SCN5A (Ala1905Gly [novel] and c.4719C>T [splice site mutation]) and 1 in LMNA (Leu327Val [novel]). Following catheter ablation, BBRVT was noninducible in all cases and none experienced a clinical recurrence during follow-up.	Small sample size	Our investigation into apparent idiopathic BBRVT has identified the first genetic culprits for this life-threatening arrhythmia, providing further insight into its underlying pathophysiology and emphasizing a potential role for genetic testing in this condition. Our findings also highlight BBRVT as a novel genetic etiology of unexplained SCD that can be cured with catheter ablation.

Study name or acronym; Author; Year published; PMID	Aim of study; Endpoints; Study type (RCT, observational — multicenter or single center; case series or other [if RCT include intervention and comparator]); Study size (N)	Patient population with inclusion and exclusion criteria	Results (absolute event rates, *p* values; OR or RR; 95% CI)	Other relevant findings or adverse events	Limitations; Other comments; Conclusions
Santangeli et al. Imaging of scar in patients with ventricular arrhythmias of right ventricular origin: cardiac magnetic resonance versus electroanatomic mapping.<br>• Year published: 2011<br>• PMID: 21736658	Aim: To compare LGE with EAM for the detection of scar in patients with arrhythmias of RV origin.<br>Size: 31	Inclusion criteria: Arrhythmias of RV origin and a biopsy-proven diagnosis of structural heart disease (18 ARVC, 13 myocarditis)	EAM scars were present in 23 (64%) patients (all with structural heart disease), whereas LGE was present only in 12 (33%). In 2 cases, EAM provided a false-positive diagnosis of a small scar in the basal perivalvular area. LGE correctly diagnosed EAM scar in 48% of patients, resulting in high positive (92%) but low negative (50%) predictive values.	Absence of LGE in these patients does not reliably rule out abnormal myocardial substrates and EAM with biopsy should be considered to increase the diagnostic yield when the clinical suspicion is high.	LGE is significantly less sensitive than EAM in identifying RV cardiomyopathic substrates. Absence of LGE does not rule out the presence of small scars, and EAM with biopsy should be considered to increase the diagnostic yield.
Haissaguerre et al. Localized structural alterations underlying a subset of unexplained sudden cardiac death.<br>• Year published: 2018<br>• PMID: 30002064	Aim: SCD because of VF is commonly unexplained in younger victims. Detailed electrophysiological mapping in such patients has not been report.<br>Endpoints: Identify origins of VF<br>Study type: Case series<br>Size: 24 patients with IVF	Inclusion criteria: 24 patients (29 ± 13 years) who survived IVF	VF mapping demonstrated reentrant and focal activities (87% versus 13%, respectively) in all. The activities were dominant in one ventricle in 9 patients, whereas they had biventricular distribution in others. During sinus rhythm areas of abnormal electrograms were identified in 15/24 patients (62.5%) revealing localized structural alterations: in the RV in 11, the left ventricle in 1, and both in 3. In the 9 patients without structural alteration, a high incidence of Purkinje triggers (7/9 versus 4/15, $p = .033$) was observed. Catheter ablation resulted in arrhythmia-free outcome in 15/18 patients at 17 ± 11 months follow-up.	Small sample size	This study shows that localized structural alterations underlie a significant subset of previously unexplained sudden cardiac death. In the other subset, Purkinje electrical pathology seems as a dominant mechanism.
**Investigation of Sudden Cardiac Arrest Survivors: Genetic Evaluation**					
Moss et al. Effectiveness and limitations of beta-	Aim: Beta-blockers are routinely prescribed in congenital LQTS, but the	Inclusion criteria: The study population comprised 869 LQTS	After initiation of beta-blockers, there was a significant ($p < .001$) reduction in the rate of cardiac events in	Patients with a history of ACA before starting	Beta-blockers are associated with a significant reduction in cardiac events in LQTS patients.

**Study name or acronym; Author; Year published; PMID**	**Aim of study; Endpoints; Study type (RCT, observational — multicenter or single center; case series or other [if RCT include intervention and comparator]); Study size (N)**	**Patient population with inclusion and exclusion criteria**	**Results (absolute event rates, *p* values; OR or RR; 95% CI)**	**Other relevant findings or adverse events**	**Limitations; Other comments; Conclusions**
blocker therapy in congenital long-QT syndrome.<br>• Year published: 2000<br>• PMID: 10673253	effectiveness and limitations of beta-blockers in this disorder have not been evaluated.<br>Size: 869	patients treated with beta-blockers. Effectiveness of beta-blockers was analyzed during matched periods before and after starting beta-blocker therapy, and by survivorship methods to determine factors associated with cardiac events while on prescribed beta-blockers.	probands (0.97 ± 1.42 to 0.31 ± 0.86 events per year) and in affected family members (0.26 ± 0.84 to 0.15 ± 0.69 events per year) during 5-year matched periods. On-therapy survivorship analyses revealed that patients with cardiac symptoms before beta-blockers (n = 598) had a hazard ratio of 5.8 (95% CI, 3.7 to 9.1) for recurrent cardiac events (syncope, ACA, or death) during beta-blocker therapy compared with asymptomatic patients; 32% of these symptomatic patients will have another cardiac event within 5 years while on prescribed beta-blockers.	Beta-blockers (n = 113) had a hazard ratio of 12.9 (95% CI, 4.7 to 35.5) for ACA or death while on prescribed beta-blockers compared with asymptomatic patients; 14% of these patients will have another arrest (aborted or fatal) within 5 years on beta-blockers.	However, syncope, ACA, and LQTS-related death continue to occur while patients are on prescribed beta-blockers, particularly in those who were symptomatic before starting this therapy.
Schwartz et al. Long QT syndrome patients with mutations of the SCN5A and HERG genes have differential responses to Na+ channel blockade and to increases in heart rate. Implications for gene-specific therapy.<br>• Year published: 1995<br>• PMID: 8521555	Aim: To test the hypothesis that the QT interval would shorten more in LQT3 than in LQT2 patients in response to mexiletine and also in response to increases in heart rate.<br>Size: 15	Inclusion criteria: 15 LQTS patients were studied. 6 LQT3 and 7 LQT2 patients were treated with mexiletine, and its effects on QT and QTc were measured.	Mexiletine significantly shortened the QT interval among LQT3 patients (QTc from 535 ± 32 to 445 ± 31 ms, $p < .005$) but not among LQT2 patients (QTc from 530 ± 79 to 503 ± 60 ms, p = NS). LQT3 patients (n = 7) shortened their QT interval in response to increases in heart rate much more than LQT2 patients (n = 4) and also more than 18 healthy control subjects (9.45 ± 3.3 vs 3.95 ± 1.97 and 2.83 ± 1.33, $p < .05$; data expressed as percent reduction in QT per 100-ms shortening in RR). Among these patients, there is also a trend for LQT2 patients to have syncope or cardiac arrest under emotional or physical stress and for LQT3 patients to have cardiac events either at rest or during sleep.	The study demonstrates differential responses of LQTS patients to interventions targeted to their specific genetic defect.	The findings also suggest that LQT3 patients may be more likely to benefit from Na+ channel blockers and from cardiac pacing because they would be at higher risk of arrhythmia at slow heart rates. Conversely, LQT2 patients may be at higher risk to develop syncope under stressful conditions because of the combined arrhythmogenic effect of catecholamines with the insufficient adaptation of their QT interval when heart rate increases.

**Study name or acronym; Author; Year published; PMID**	**Aim of study; Endpoints; Study type (RCT, observational — multicenter or single center; case series or other [if RCT include intervention and comparator]); Study size (N)**	**Patient population with inclusion and exclusion criteria**	**Results (absolute event rates, *p* values; OR or RR; 95% CI)**	**Other relevant findings or adverse events**	**Limitations; Other comments; Conclusions**
Moss et al. Safety and efficacy of flecainide in subjects with long QT-3 syndrome (DeltaKPQ mutation): a randomized, double-blind, placebo-controlled clinical trial.<br>• Year published: 2005<br>• PMID: 16274417	Aim: To evaluate the safety and efficacy of flecainide in LQT-3.<br>Study type: Randomized controlled trial<br>Size: 6 male LQT-3 subjects with the DeltaKPQ deletion	Inclusion criteria: A study of chronic therapy with flecainide versus placebo was conducted in a small group of LQT-3 patients with the DeltaKPQ deletion.	The lowest possible dose of flecainide associated with at least a 40 ms reduction in the QTc interval was determined in an initial open-label, dose-ranging investigation using one-fourth or half of the recommended maximal antiarrhythmic flecainide dose. QTc reduction was achieved with a flecainide dose of 1.5 mg/kg per day in 4 subjects and with 3.0 mg/kg per day in 2 subjects. Subjects were randomized to 4 6-month alternating periods of flecainide and placebo therapy based on the open-label dose findings. Average QTc values during placebo and flecainide therapies were 534 ms and 503 ms, respectively, with an adjusted reduction in QTc of -27.1 ms (95% confidence interval: -36.8 ms to -17.4 ms; $p < .001$) at a mean flecainide blood level of 0.11 ± 0.05 microg/ml.	Minimal prolongation in QRS occurred (mean: +2.5 ms), and there were no major adverse cardiac effects.	Chronic low-dose flecainide significantly shortens the QTc interval in LQT-3 subjects with the DeltaKPQ mutation. No major adverse drug effects were observed with flecainide during this trial, but the sample size is not large enough to evaluate the safety of flecainide therapy in patients with this mutation.
Moss et al. Ranolazine shortens repolarization in patients with sustained inward sodium current due to type-3 long-QT syndrome.<br>• Year published: 2008<br>• PMID: 18662191	Aim: To hypothesize that ranolazine would have beneficial effects on electrical and mechanical cardiac function in LQT3 patients with the SCN5A-DeltaKPQ mutation.<br>Size: 5	The effects of 8-h intravenous ranolazine infusions (45 mg/h for 3 h followed by 90 mg/h for 5 h) on ventricular repolarization and myocardial relaxation in 5 LQT3 patients with the SCN5A-Delta KPQ mutation were assessed.	Ranolazine shortened QTc by 26 ± 3 ms ($p < .0001$) in a concentration-dependent manner. At peak ranolazine infusion, there was a significant 13% shortening in left ventricular isovolumic relaxation time, a significant 25% increase in mitral E-wave velocity, and a meaningful 22% decrease in mitral E-wave deceleration time compared with the baseline.	No adverse effects of ranolazine were observed in the study patients.	Ranolazine at therapeutic concentrations shortened a prolonged QTc interval and improved diastolic relaxation in patients with the LQT3-Delta KPQ mutation, a genetic disorder that is known to cause an increase in late sodium current.

**Study name or acronym; Author; Year published; PMID**	**Aim of study; Endpoints; Study type (RCT, observational — multicenter or single center; case series or other [if RCT include intervention and comparator]); Study size (N)**	**Patient population with inclusion and exclusion criteria**	**Results (absolute event rates, *p* values; OR or RR; 95% CI)**	**Other relevant findings or adverse events**	**Limitations; Other comments; Conclusions**
Kannankeril et al. Efficacy of flecainide in the treatment of catecholaminergic polymorphic ventricular tachycardia: a randomized clinical trial.<br>• Year published: 2017<br>• PMID: 28492868	Aim: To determine whether flecainide dosed to therapeutic levels and added to beta-blocker therapy is superior to beta-blocker therapy alone for the prevention of exercise-induced arrhythmias in CPVT.<br>Study type: Multicenter, single-blind, placebo-controlled crossover clinical trial<br>Size: 14	Inclusion criteria: Patients with a clinical diagnosis of CPVT and an ICD underwent a baseline exercise test while receiving maximally tolerated beta-blocker therapy that was continued throughout the trial. Patients were then randomized to treatment A (flecainide or placebo) for 3 months, followed by exercise testing. After a 1-week washout period, patients crossed over to treatment B (placebo or flecainide) for 3 months, followed by exercise testing.	Of 14 patients (7 males and 7 females; median age, 16 years [interquartile range, 15.0-22.5 years]) randomized, 13 completed the study. The median baseline exercise test score was 3.0 (range, 0-4), with no difference noted between the baseline and placebo (median, 2.5; range, 0-4) exercise scores. The median ventricular arrhythmia score during exercise was significantly reduced by flecainide (0 [range, 0-2] vs 2.5 [range, 0-4] for placebo; $p < .01$), with complete suppression observed in 11 of 13 patients (85%).	Overall and serious adverse events did not differ between the flecainide and placebo arms.	Flecainide plus beta-blocker significantly reduced ventricular ectopy during exercise compared with placebo plus beta-blocker and beta-blocker alone.
James et al. Exercise increases age-related penetrance and arrhythmic risk in arrhythmogenic right ventricular dysplasia/ cardiomyopathy-associated desmosomal mutation carriers.<br>• Year published: 2013<br>• PMID: 23871885	Aim: To determine how exercise influences penetrance of ARVD/C among patients with desmosomal mutations.<br>Size: 87	87 carriers (46 male; mean age, 44 ± 18 years) were interviewed about regular physical activity from 10 years of age. The relationship of exercise with sustained ventricular arrhythmia (VT/VF), stage C heart failure, and meeting diagnostic criteria for ARVD/C (2010 Revised Task Force Criteria) was studied.	Symptoms developed in endurance athletes (N = 56) at a younger age (30.1 ± 13.0 years vs. 40.6 ± 21.1 years, $p = .05$); they were more likely to meet Task Force Criteria at last follow-up (82% vs. 35%, $p < .001$) and have a lower lifetime survival free of VT/VF ($p = .013$) and heart failure ($p = .004$). Compared with those who did the least exercise per year (lowest quartile) before presentation, those in the second (odds ratio [OR]: 6.64, $p = .013$), third (OR: 16.7, $p = .001$), and top (OR: 25.3, $p < .0001$) quartiles were increasingly likely to meet Task Force	Survival from a first VT/VF event was lowest among those who exercised most (top quartile) both before ($p = .036$) and after ($p = .005$) clinical presentation. Among individuals in the top quartile, a reduction in exercise decreased VT/VF risk ($p = .04$).	Endurance exercise and frequent exercise increase the risk of VT/VF, heart failure, and ARVD/C in desmosomal mutation carriers. These findings support exercise restriction for these patients.

**Study name or acronym; Author; Year published; PMID**	**Aim of study; Endpoints; Study type (RCT, observational — multicenter or single center; case series or other [if RCT include intervention and comparator]); Study size (N)**	**Patient population with inclusion and exclusion criteria**	**Results (absolute event rates, *p* values; OR or RR; 95% CI)**	**Other relevant findings or adverse events**	**Limitations; Other comments; Conclusions**
			Criteria. Among 61 individuals who did not present with VT/VF, the 13 subjects experiencing a first VT/VF event over a mean follow-up of 8.4 ± 6.7 years were all endurance athletes (*p* = .002).		
Ruwald et al. Association of competitive and recreational sport participation with cardiac events in patients with arrhythmogenic right ventricular cardiomyopathy: results from the North American multidisciplinary study of arrhythmogenic right ventricular cardiomyopathy.<br>• Year published: 2015<br>• PMID: 25896080	Aim: It has been proposed that competitive sport increases the risk of VTA and death in patients with ARVC. However, it is unknown whether this only applies to competitive sport or if recreational sports activity also increases the risk of VTA/death.<br>Size: 108	Inclusion criteria: Probands diagnosed with ARVC according to the 2010 Task Force Criteria for ARVC	Competitive sport was associated with a significantly higher risk of VTA/death when compared with both recreational sport [HR = 1.99 (1.21-3.28), *p* = .007] and inactive patients [HR = 2.05 (1.07-3.91), *p* = .030]. No increased risk of VTA/death was associated with recreational sport when compared with patients who were inactive [HR = 1.03 (0.54-1.97), *p* = .930]. Symptoms developed at an earlier age in patients who participated in competitive sport (30 ± 12 years), when compared with patients who participated in recreational sport (38 ± 17 years) (*p* = .015) and inactive patients (41 ± 11 years) (*p* = .002).	No difference in age at first symptom was seen between patients who participated in recreational sport and inactive patients (*p* = .651).	Competitive sport was associated with a two-fold increased risk of VTA/death, and earlier presentation of symptoms, when compared with inactive patients, and to patients who participated in recreational sport. When compared with inactive patients, recreational sport was not associated with earlier onset of symptoms or increased risk of VTA/death.
van Rijsingen et al. Risk factors for malignant ventricular arrhythmias in Lamin A/C mutation carriers a European cohort study.	Aim: To determine risk factors that predict MVA in Lamin A/C (LMNA) mutation carriers.<br>Size: 269	Inclusion criteria: In this multicenter cohort of 269 LMNA mutation carriers, we evaluated risk factors for MVA, defined as sudden cardiac death, resuscitation, and appropriate ICD treatment.	In a median follow-up period of 43 months (IQR: 17 to 101 months), 48 (18%) persons experienced a first episode of MVA: 11 persons received successful CPR, 25 received appropriate ICD treatment, and 12 persons died suddenly. Independent risk factors for MVA were nonsustained VT, left ventricular ejection fraction <45% at the first	MVA occurred only in persons with at least 2 of these risk factors. There was a cumulative risk for MVA per additional risk factor.	Carriers of LMNA mutations with a high risk of MVA can be identified using these risk factors. This facilitates selection of LMNA mutation carriers who are most likely to benefit from an ICD.

**Study name or acronym; Author; Year published; PMID**	**Aim of study; Endpoints; Study type (RCT, observational — multicenter or single center; case series or other [if RCT include intervention and comparator]); Study size (N)**	**Patient population with inclusion and exclusion criteria**	**Results (absolute event rates, *p* values; OR or RR; 95% CI)**	**Other relevant findings or adverse events**	**Limitations; Other comments; Conclusions**
• Year published: 2012<br>• PMID: 22281253			clinical contact, male sex, and nonmissense mutations (ins-del/truncating or mutations affecting splicing).		
van Rijsingen et al. Outcome in phospholamban R14del carriers: results of a large multicentre cohort study.<br>• Year published: 2014<br>• PMID: 24909667	Aim: To evaluate mortality, cardiac disease outcome, and risk factors for MVA in a cohort of PLN R14del mutation carriers.<br>Size: 403	Inclusion criteria: Using the family tree mortality ratio method in a cohort of 403 PLN R14del mutation carriers, a standardized mortality ratio of 1.7 (95% CI, 1.4-2.0) with significant excess mortality starting from the age of 25 years was found. Cardiological data were available for 295 carriers.	In a median follow-up period of 42 months, 55 (19%) individuals had a first episode of MVA and 33 (11%) had an end-stage heart failure event. The youngest age at which MVA occurred was 20 years, whereas for an end-stage heart failure event this was 31 years.	Independent risk factors for MVA were left ventricular ejection fraction <45% and sustained or nonsustained VT with hazard ratios of 4.0 (95% CI, 1.9-8.1) and 2.6 (95% CI, 1.5-4.5), respectively.	PLN R14del mutation carriers are at high risk for MVA and end-stage heart failure, with left ventricular ejection fraction <45% and sustained or nonsustained VT as independent risk factors. High mortality and a poor prognosis are present from late adolescence. Genetic and cardiac screening is, therefore, advised from adolescence onwards.
Mellor et al. Genetic testing in the evaluation of unexplained cardiac arrest: from the CASPER (Cardiac Arrest Survivors with Preserved Ejection Fraction Registry).<br>• Year published: 2017<br>• PMID: 28600387	Aim: To assess role of genetic testing in individuals without a clinical phenotype.<br>Endpoints: Mutation carrier<br>Size: 174 genetic testing / 375 cardiac arrest survivors in CASPER; genetic testing of cardiac genes at the discretion of the treating provider	Inclusion criteria: UCA with documented VT or VF requiring direct current cardioversion or defibrillation, and subsequent testing demonstrated normal left ventricular function (left ventricular ejection fraction ≥50%) and normal coronary arteries (no coronary stenosis >50%)<br>Exclusion criteria: Known causes for cardiac arrest, including an ECG diagnostic of	29 individuals with a pathogenic variant (17% yield); pathogenic variants identified in 18/72 (25%) of phenotype positive patients; pathogenic variants identified in 11/102 (11%) of phenotype negative patients	Mean age 39 years; 18% with ≥VUS; prior syncope (OR 4) and fam hx of SCA (OR 3.2) were associated with pathogenic variant carrier status	Nonstandardized genetic testing panels; no controls; no segregation data.<br>Prior syncope and family history of sudden death are predictors of a positive genetic test. Both arrhythmia and cardiomyopathy genes are implicated. Broad, multiphenotype testing revealed the highest frequency of pathogenic variants in phenotype-negative patients.

Study name or acronym; Author; Year published; PMID	Aim of study; Endpoints; Study type (RCT, observational — multicenter or single center; case series or other [if RCT include intervention and comparator]); Study size (N)	Patient population with inclusion and exclusion criteria	Results (absolute event rates, *p* values; OR or RR; 95% CI)	Other relevant findings or adverse events	Limitations; Other comments; Conclusions
		LQTS (persistent resting QTc >460 ms for males and 480 ms for females) or BrS, HCM, marked hypokalemia, drug overdose, or commotio cordis, recognized forms of idiopathic VT			
De Ferrari et al. Clinical management of catecholaminergic polymorphic ventricular tachycardia: the role of left cardiac sympathetic denervation.<br>• Year published: 2015<br>• PMID: 26019152	Aim: To propose LCSD as useful additional therapy, but evidence remains anecdotal.<br>Study type: Multicenter<br>Size: 63	Inclusion criteria: Patients with CPVT who underwent LCSD as secondary (n = 54) or primary (n = 9) prevention	The median post-LCSD follow-up was 37 months. The 9 asymptomatic patients remained free of major cardiac events. Of the 54 patients with prior major cardiac events either on (n = 38) or off (n = 16) optimal medical therapy, 13 (24%) had at least 1 recurrence: 0 patients had an ACA, 2 patients had syncope only, 10 patients had ≥1 appropriate ICD discharges, and 1 patient died suddenly. The 1- and 2-year cumulative event-free survival rates were 87% and 81%. The percentage of patients with major cardiac events despite optimal medical therapy (n = 38) was reduced from 100% to 32% ($p < .001$) after LCSD, and among 29 patients with a presurgical ICD, the rate of shocks dropped by 93% from 3.6 to 0.6 shocks per person per year ($p < .001$).	Patients with an incomplete LCSD (n = 7) were more likely to experience major cardiac events after LCSD (71% versus 17%; $p < .01$) than those with a complete LCSD.	LCSD is an effective antifibrillatory intervention for patients with CPVT. Whenever syncope occurs despite optimal medical therapy, LCSD could be considered the next step rather than an ICD and could complement ICDs in patients with recurrent shocks.
Coleman et al. Videoscopic left cardiac sympathetic denervation for	Aim: Although LCSD is well established in LQTS, its role in non-LQTS arrhythmogenic channelopathies and cardiomyopathies is less	Inclusion criteria: Patients who were denervated for an underlying diagnosis other than autosomal	5 patients had LCSD because of high-risk assessment and beta-blocker intolerance, none of whom had a sentinel breakthrough cardiac event at early follow-up. Among the remaining	LCSD may represent a substrate-independent antifibrillatory treatment option	The early follow-up seems promising, with a marked reduction in the frequency of cardiac events postdenervation.

Study name or acronym; Author; Year published; PMID	Aim of study; Endpoints; Study type (RCT, observational — multicenter or single center; case series or other [if RCT include intervention and comparator]); Study size (N)	Patient population with inclusion and exclusion criteria	Results (absolute event rates, *p* values; OR or RR; 95% CI)	Other relevant findings or adverse events	Limitations; Other comments; Conclusions
patients with recurrent ventricular fibrillation/malignant ventricular arrhythmia syndromes besides congenital long-QT syndrome.<br>• Year published: 2012<br>• PMID: 22787014	clear. The aim is to report the single-center experience in performing LCSD in this setting.<br>Size: 27	dominant or sporadic LQTS	22 previously symptomatic patients who had LCSD as secondary prevention, all had an attenuation in cardiac events, with 18 having no breakthrough cardiac events so far and 4 having experienced ≥1 post-LCSD breakthrough cardiac event.	for patients with life-threatening ventricular arrhythmia syndromes other than LQTS.	
Bos et al. Mexiletine shortens the QT interval in patients with potassium channel-mediated type 2 long QT syndrome.<br>• Year published: 2019<br>• PMID: 31006312	Aim: The potential role of sodium channel blockers in patients with potassium channel-mediated LQTS (ie, LQT1 and LQT2) has not been investigated in detail.<br>Size: 12	Inclusion criteria: Patients with genetically established LQT2 or a combination of LQT1/LQT2 or LQT2/LQT3 who received mexiletine	Before diagnosis, 6 patients were symptomatic and, before initiation of mexiletine, 4 patients experienced ≥1 breakthrough cardiac event on beta-blocker. Median age at first mexiletine dose was 24.3 years (IQR, 14-32.4). After mexiletine, the median QTc decreased by 65±45 ms from 547 ms (IQR, 488-558) premexiletine to 470 ms (IQR, 409-529) postmexiletine (*p* = .0005) for all patients. In 8 patients (67%), the QTc decreased by ≥ 40 ms with a mean decrease in QTc of 91 ms (*p* < .008).	For the 11 patients maintained on mexiletine therapy, there have been no breakthrough cardiac events during follow-up.	Although commonly prescribed in patients with LQT3, mexiletine also shortens the QTc significantly in two-thirds of a small subset of patients with potassium channel-mediated LQT2. In patients with LQT2, pharmacological targeting of the physiological late sodium current may provide added therapeutic efficacy to beta-blocker therapy.
Burns et al. Multiple gene variants in hypertrophic cardiomyopathy in the era of next-generation sequencing.	Aim: To re-examine the significance of multiple rare variants in patients with HCM in the setting of comprehensive and targeted panels.<br>Size: 382	Inclusion criteria: Of 758 HCM probands, we included 382 with ≥45 cardiomyopathy genes screened. There were 224 (59%) with ≥1 rare variant (allele frequency ≤0.02%). Variants were analyzed	Based on a 45-gene panel, 127 (33%) had a LP/P variant, 139 (36%) had VUS, and 66 (17%) had multiple rare variants. A targeted 8-gene panel yielded 125 (32%) LP/P variants, 52 (14%) VUS, and 14 (4%) had multiple rare variants. No proband had 2 LP/P variants. Including affected family members (total n = 412), cluster-	Those with multiple variants had worse event-free survival from all-cause death, cardiac death, cardiac transplantation, and cardiac arrest (log-rank *p* = .008).	No proband had multiple LP/P variants in contrast to previous reports. However, multiple rare variants regardless of classification were seen in 4% and contributed to earlier disease onset and cardiac events. Our findings support a

Study name or acronym; Author; Year published; PMID	Aim of study; Endpoints; Study type (RCT, observational — multicenter or single center; case series or other [if RCT include intervention and comparator]); Study size (N)	Patient population with inclusion and exclusion criteria	Results (absolute event rates, *p* values; OR or RR; 95% CI)	Other relevant findings or adverse events	Limitations; Other comments; Conclusions
• Year published: 2017<br>• PMID: 28790153		using varying sized gene panels to represent comprehensive or targeted testing.	adjusted analyses identified a phenotype effect, with younger age (odds ratio, 0.95; 95% confidence interval, 0.92-0.98; *p* = .004) and family history of SCD (odds ratio, 3.5; 95% confidence interval, 1.3-9.9; *p* = .02) significantly more likely in multiple versus single variant patients when considering an 8-gene panel but not larger panels.		cumulative variant hypothesis in HCM.
Kumar et al. Familial cardiological and targeted genetic evaluation: low yield in sudden unexplained death and high yield in unexplained cardiac arrest syndromes.<br>• Year published: 2013<br>• PMID: 23973953	Aim: To assess value of a clinical and genetic protocol for evaluating SCA patients<br>Endpoints: Diagnosis<br>Study type: Case series<br>Size: 52 probands with UCA	Inclusion criteria: Unexplained SCA<br>Exclusion criteria: An identifiable noncardiac etiology, any evidence of CAD on ECG or coronary angiography, and abnormal ventricular function or valvular heart disease	Clinical diagnosis made in 32 (62%); genetic testing performed in 25/32 families (26 genes); pathogenic variant identified in 12/25 tested (yield 48%)	Mean age 32 years	In contrast to previously published series, a comprehensive strategy of cardiological evaluation and targeted genetic testing in more than 100 families with SADS was found to have a lower diagnostic yield (18%). Diagnostic yield in families with UCA was approximately 4 times higher (62%), which is consistent with the published literature.
Jiménez-Jáimez et al. Diagnostic approach to unexplained cardiac arrest (from the FIVI-Gen Study).<br>• Year published: 2015<br>• PMID: 26189708	Aim: To assess a clinical and genetic diagnostic protocol for UCA.<br>Endpoints: Diagnosis<br>Study type: Multicenter retrospective cohort<br>Size: 35 unexplained SCA survivors; 126 heart genes tested	Inclusion criteria: UCA (VF with no diagnostic findings on the ECG, no pathologic findings on the echocardiogram, and no angiographic lesions with >50% stenosis on coronary catheterization)<br>Exclusion criteria: prolonged QT (QTc	Diagnosis made in 51% of cases; 20% based on pharmacologic testing; 14% based on family assessment; 21% based on genetic testing	Mean age 40 years	If interpreted carefully, genetic tests can be a useful tool for diagnosing UCA without a phenotype.

**Study name or acronym; Author; Year published; PMID**	**Aim of study; Endpoints; Study type (RCT, observational — multicenter or single center; case series or other [if RCT include intervention and comparator]); Study size (N)**	**Patient population with inclusion and exclusion criteria**	**Results (absolute event rates, *p* values; OR or RR; 95% CI)**	**Other relevant findings or adverse events**	**Limitations; Other comments; Conclusions**
		interval >460 ms in male and >480 ms in female subjects), Brugada pattern in RPLs and/or pathologic Q waves, documented monomorphic VT, structural heart disease on transthoracic echocardiogram or CMR, probable channelopathy in the proband or first-degree relatives, or ARVC, in accordance with published criteria, evidence of drug use, severe electrolyte abnormality, ischemia, extreme bradycardia, or any other secondary cause of VF and coronary artery anomaly/vasospasm			
Asatryan et al. Usefulness of genetic testing in sudden cardiac arrest survivors with or without previous clinical evidence of heart disease.<br>• Year published: 2019<br>• PMID: 30975432	Aim: To assess utility of genetic testing in phenotype + and – SCA survivors.<br>Endpoints: Positive genetic test<br>Study type: Prospective/ retrospective cohort<br>Size: 60	Inclusion criteria: SCA patients referred to the Bern University Hospital for genetic testing between January 2014 and May 2018	N = 24 phenotype “positive” and 36 phenotype “negative” individuals;<br>185 clinically relevant cardiac genes;<br>17/24 (71%) genotype + individuals among phenotype +;<br>10/36 (28%) genotype + individuals among phenotype -	Mix of prospective and retrospective collection;<br>mix of exome and panel testing	The test was useful in both groups to identify or confirm an inherited heart disease, with an important impact on the patient care and first-degree relatives at risk.

Study name or acronym; Author; Year published; PMID	Aim of study; Endpoints; Study type (RCT, observational — multicenter or single center; case series or other [if RCT include intervention and comparator]); Study size (N)	Patient population with inclusion and exclusion criteria	Results (absolute event rates, *p* values; OR or RR; 95% CI)	Other relevant findings or adverse events	Limitations; Other comments; Conclusions
Ashar et al. A comprehensive evaluation of the genetic architecture of sudden cardiac arrest.<br>• Year published: 2018<br>• PMID: 30169657	Aim: To identify potential loci associated with SCA and to identify risk factors causally associated with SCA.<br>Size: 3,939 cases, 25,989 noncases	A large GWAS was carried out for SCA (n = 3,939 cases, 25,989 noncases) to examine common variation genome-wide and in candidate arrhythmia genes. MR methods were exploited using cross-trait multivariant GRSA to assess causal relationships of 18 risk factors with SCA.	No variants were associated with SCA at genome-wide significance, nor were common variants in candidate arrhythmia genes associated with SCA at nominal significance. Using cross-trait GRSA, we established genetic correlation between SCA and CAD and traditional CAD risk factors (blood pressure, lipids, and diabetes); height and BMI; and electrical instability traits (QT and atrial fibrillation), suggesting etiologic roles for these traits in SCA risk.	Comprehensive approach to the genetic architecture of SCA can shed light on the determinants of a complex life-threatening condition with multiple influencing factors in the general population.	The results of this genetic analysis, both positive and negative findings, have implications for evaluating the genetic architecture of patients with a family history of SCA, and for efforts to prevent SCA in high-risk populations and the general community.
Milano et al. Sudden cardiac arrest and rare genetic variants in the community.<br>• Year published: 2016<br>• PMID: 26800703	Aim: To assess yield of genetic testing for select rare founder variants in a Dutch SCA cohort (PLN-p.Arg14del, MYBPC3-p.Trp792fsX17, MYBPC3-p.Arg943X, MYBPC3-p.Pro955fsX95, PKP2-p.Arg79X, and the Chr7q36 IVF risk haplotype).<br>Endpoints: Carrier status<br>Study type: Case-control<br>Size: 1,440 cases, 1,379 geographically matched controls/500 GoNL nationwide controls with WGS/8,261 PREVEND controls (Groningen)	Inclusion criteria: ARREST study/OHCA occurring in North Holland province of the Netherlands<br>Exclusion criteria: Noncardiac cause of VF, only asystole	1.1% prevalence of any of the 6 variants in cases vs. 0.4% in ethnically/ geographically matched controls ($p < .05$)	Proof-of-concept for the notion that rare genetic variants contribute to some extent to SCA risk in the community	No ancestry adjustment<br>Founder mutations<br>2 of the variants (MYBPC3-p.Pro955fsX95, Chr7q36 IVF risk haplotype) not observed

**Study name or acronym; Author; Year published; PMID**	**Aim of study; Endpoints; Study type (RCT, observational — multicenter or single center; case series or other [if RCT include intervention and comparator]); Study size (N)**	**Patient population with inclusion and exclusion criteria**	**Results (absolute event rates, *p* values; OR or RR; 95% CI)**	**Other relevant findings or adverse events**	**Limitations; Other comments; Conclusions**
Visser et al. Next-generation sequencing of a large gene panel in patients initially diagnosed with IVF.<br>• Year published: 2017<br>• PMID: 28087426	Aim: To assess the diagnostic yield of NGS in patients with IVF.<br>Size: 33	Inclusion criteria: Patients initially diagnosed with IVF	In 1 of 33 patients, a likely pathogenic mutation was detected. The added yield of genetic testing with NGS of 179 additional genes is 3% in patients with IVF. In 15% of patients, 1 or multiple variants of uncertain clinical significance were detected.	The added yield of genetic screening of extended NGS panels in patients initially diagnosed with IVF is minimal.	Routine analysis of large diagnostic NGS panels is therefore not recommended.
Visser et al. Long-term outcome of patients initially diagnosed with IVF: a descriptive study.<br>• Year published: 2016<br>• PMID: 27733492	Aim: IVF is a rare cause of SCA. Limited data are available on the long-term outcome of IVF patients.<br>Study type: Retrospective cohort<br>Size: 107	Inclusion criteria: Initial diagnosis of IVF; missing diagnostic data were acquired during follow-up, including genetic testing, to exclude underlying disease.	A specific diagnosis was revealed in 22 of 107 patients (21%) during a median follow-up of 10.2 years. Mortality rate was 9% in IVF patients (8/85). Appropriate ICD therapy was delivered in 23 patients (29%) of 78 IVF patients with an ICD, with a median of 3 appropriate shocks per patient.	One-fifth of the patients initially diagnosed with IVF reveal a specific diagnosis during long-term follow-up.	Additional diagnostic testing, including genetic testing, contributes to the detection of specific diseases. The recurrence rate of ventricular arrhythmias in IVF patients is high. Our data show the importance of thorough follow-up and reassessment of diagnosis in IVF patients.
Ingles et al. Concealed arrhythmogenic right ventricular cardiomyopathy in sudden unexplained cardiac death events.<br>• Year published: 2018<br>• PMID: 30571190	Aim: To report a unique case series of sudden cardiac death events in the young with structurally normal hearts and causative PKP2 variants.<br>Study type: Case report<br>Size: 4	Inclusion criteria: Probands who presented because of an unexplained SCA with overt structurally normal hearts in early adulthood with causative loss of function variants in the PKP2 gene subsequently identified	All variants led to loss of function of PKP2 (and severe clinical presentations, despite a previous study suggesting nonsense variants are associated with later onset disease.	Although the arrhythmic risk is not reported before structural disease, most knowledge about PKP2 carriers comes from observational studies of ARVC cohorts.	This has important implications and presents a clinical conundrum in terms of management of asymptomatic ARVC gene carriers who do not have overt structural disease.

Study name or acronym; Author; Year published; PMID	Aim of study; Endpoints; Study type (RCT, observational — multicenter or single center; case series or other [if RCT include intervention and comparator]); Study size (N)	Patient population with inclusion and exclusion criteria	Results (absolute event rates, *p* values; OR or RR; 95% CI)	Other relevant findings or adverse events	Limitations; Other comments; Conclusions
Yang et al. Allelic variants in long-QT disease genes in patients with drug-associated torsades de pointes.<br>• Year published: 2002<br>• PMID: 11997281	Aim: DNA variants appearing to predispose to drug-associated aLQTS have been reported in congenital long-QT disease genes. However, the incidence of these genetic risk factors has not been systematically evaluated in a large set of patients with aLQTS.	Inclusion criteria: The coding regions of the genes encoding the pore-forming channel proteins KvLQT1, HERG, and SCN5A were screened in the same aLQTS cohort (n = 92) and controls, drawn from patients tolerating QT-prolonging drugs (n = 67) and cross sections of the Middle Tennessee (n = 71) and US populations (n = 90)	The frequency of 3 common nonsynonymous coding region polymorphisms was no different between aLQTS and control subjects, as follows: 24% versus 19% for H558R (SCN5A), 3% versus 3% for R34C (SCN5A), and 14% versus 14% for K897T (HERG). Missense mutations (absent in controls) were identified in 5 of 92 patients. KvLQT1 and HERG mutations (one each) reduced K+ currents in vitro, consistent with the idea that they augment risk for aLQTS.	Three SCN5A variants did not alter I(Na), which argues that they played no role in the aLQTS phenotype.	DNA variants in the coding regions of congenital long-QT disease genes predisposing to aLQTS can be identified in approximately 10% to 15% of affected subjects, predominantly in genes encoding ancillary subunits.
Giudicessi et al. Role of genetic heart disease in sentinel sudden cardiac arrest survivors across the age spectrum.<br>• Year published: 2018<br>• PMID: 29884292	Aim: To determine the contribution of GHDs in single-center referral cohort of nonischemic SCA survivors.<br>Size: 180	Inclusion criteria: Retrospective analysis of 3,037 patients was used to identify all individuals who experienced a sentinel event of SCA. Following exclusion of patients with ischemic or complex congenital heart disease, cases were classified by clinical diagnoses.	Overall, 180 (5.9%) referral patients experienced a sentinel SCA (average age at SCA 28 ± 15 years, 99 females). An etiology was identified in 113/180 patients (62.8%) including channelopathies in 26.7%, arrhythmogenic bileaflet mitral valve prolapse in 10.6%, cardiomyopathies in 9.4%, other etiologies in 6.7%, aLQTS in 6.7%, and multiple disorders in 2.8%. The remaining 67/180 (37.2%) cases were classified as IVF.	The contribution of GHDs declined precipitously after the first decade of life [90.0% (age 0-9; n = 20), 58.7% (age 10-19; n = 46), 28.1% (age 20-29; n = 32), 23.8% (age 30-39; n = 42), 16.7% (age 40-49; n = 24), and 12.5% (age 50+; n = 16)].	Within a referral population enriched for GHDs, the ability of a comprehensive cardiac evaluation, including genetic testing, to elucidate a root cause in nonischemic SCA survivors declined with age. Although rare, GHDs can underlie SCA into adulthood and merit consideration across the age spectrum.
Tester et al. Plakophilin-2 truncation variants in patients clinically diagnosed with catecholaminergic polymorphic	Aim: To determine if radical plakophilin-2 (PKP2) variants might underlie some cases of clinically diagnosed CPVT and exercise-associated, autopsy-negative SUDY.	Inclusion criteria: 18 unrelated patients clinically diagnosed with CPVT but who were RYR2-, CASQ2-, KCNJ2-, and TRDN-negative, and 19	Radical PKP2 variants were identified in 5 of 18 (27.7%) CPVT patients and 1 of 19 (5.3%) exercise-related SUDY cases compared with 96 of 138,632 (0.069%) individuals in gnomAD (p = 3.1×10^{-13}).	Cardiac imaging or autopsy demonstrated a structurally normal heart in all patients at the time of their	Progression of the PKP2-dependent electropathy can be independent of structural perturbations and can precipitate exercise-associated SCA or SCD before the presence of overt cardiomyopathy, which

Study name or acronym; Author; Year published; PMID	Aim of study; Endpoints; Study type (RCT, observational — multicenter or single center; case series or other [if RCT include intervention and comparator]); Study size (N)	Patient population with inclusion and exclusion criteria	Results (absolute event rates, *p* values; OR or RR; 95% CI)	Other relevant findings or adverse events	Limitations; Other comments; Conclusions
ventricular tachycardia and decedents with exercise-associated autopsy negative sudden unexplained death in the young.<br>• Year published: 2019<br>• PMID: 30678776	Size: 18 patients and 19 decedents with SUDY	decedents with SUDY during exercise		CPVT diagnosis or sudden death.	clinically mimics CPVT, similar to the PKP2 knockout mouse model. Thus, CPVT and SUDY genetic test panels should now include PKP2.
**Investigation of the Family: Cause Identified—Cascade Testing, Clinical and Genetic Investigations**					
Knight et al. Genetic testing and cascade screening in pediatric long QT syndrome and hypertrophic cardiomyopathy.<br>• Year published: 2020<br>• PMID: 31229680	Aim: To examine the use of genetic testing and yield of cascade screening across diverse regions in the United States and to evaluate obstacles to screening in multipayer systems.<br>Endpoints: Percentage of index patients undergoing genetic testing, #patients with pathogenic mutation, proportion of families participating in cascade testing, #positive results per family<br>Study type: Observational, multicenter<br>Size: 315 index patients	Inclusion criteria:<br>Age <21 years and a clinical diagnosis of and/or gene-positive status for LQTS or HCM established from 2008–2014; from specialty cardiology clinics at 6 centers in the USA	Genetic testing was performed in 250 of the 315 index patients (79%). 182 received a positive test result, 125 for LQTS (81%) and 57 for HCM (60%).<br>(2) Of the 315 families studied, 234 (74%) participated in cascade screening. A total of 553 relatives were screened, for a rate of 2.4 per participating family.<br>Cascade screening identified 221 affected individuals (40%) among the 553 relatives screened (0.94 relatives detected per index family participating in screening). The LQTS screen-positive rate was 42% and HCM 37%.	Contrary to a widespread perception, insurance was not a significant barrier to cascade screening, nor was it the primary barrier to index patient genetic testing.	The 74% participation in this report demonstrates that participation is not complete even in tertiary centers with comprehensive arrhythmia programs including genetic counselors.<br>Cascade screening detected a positive result in 39% of relatives with a yield of ~1 person per family.
Catchpool et al. A cost-effectiveness model of genetic testing and periodical clinical	Aim: To assess the relative cost-effectiveness of cascade genetic testing in asymptomatic relatives of patients with DCM	A decision-analytic model, combining a decision tree and a Markov model, was used to determine the	The incremental cost per additional QALY of cascade genetic testing prior to periodical clinical surveillance of first-degree relatives compared with periodical clinical surveillance alone	Extensive sensitivity analyses, including the addition of second-degree relatives, did not	Using cascade genetic testing to guide clinical surveillance of asymptomatic relatives of patients with DCM is very likely to be cost-effective. As the DCM

Study name or acronym; Author; Year published; PMID	Aim of study; Endpoints; Study type (RCT, observational — multicenter or single center; case series or other [if RCT include intervention and comparator]); Study size (N)	Patient population with inclusion and exclusion criteria	Results (absolute event rates, *p* values; OR or RR; 95% CI)	Other relevant findings or adverse events	Limitations; Other comments; Conclusions
screening for the evaluation of families with dilated cardiomyopathy.<br>• Year published: 2019<br>• PMID: 31222143	compared with periodical clinical surveillance.	lifetime costs and QALYs for the two strategies. Deterministic and probabilistic sensitivity analyses were undertaken to assess the robustness of findings and to explore decision uncertainty.	was estimated at approximately AUD $6,100. At established thresholds of cost-effectiveness, there is a 90% probability that cascade genetic testing is cost-effective.	alter the conclusions drawn from the main analysis.	pathogenic variant detection rate rises and new evidence for personalized treatment of at-risk individuals becomes available, the cost-effectiveness of cascade testing will further increase.
Mak et al. Sudden arrhythmia death syndrome in young victims: a five-year retrospective review and two-year prospective molecular autopsy study by next-generation sequencing and clinical evaluation of their first-degree relatives.<br>• Year published: 2019<br>• PMID: 30670673	Aim: To investigate the value of molecular autopsy techniques for detecting Sudden arrhythmia death syndrome in an East Asian population.<br>Study type: (1) retrospective; (2) prospective study<br>Size: 289 retrospective victims, 21 prospective victims, 11 first-degree relatives	Inclusion criteria: First, a retrospective 5-year review of autopsies performed in public mortuaries on young SCD victims was conducted. Second, a prospective 2-year study combining conventional autopsy investigations, molecular autopsy, and cardiac evaluation of the first-degree relatives of SCD victims was conducted. A panel of 35 genes implicated in SADS was analyzed by NGS.	There were 289 SCD victims included in the 5-year review. CAD was the major cause of death (35%); 40% were structural heart diseases and 25% were unexplained. These unexplained cases could include SADS-related conditions. In the 2-year prospective study, 21 SCD victims were examined: 10% had ARVC, 5% had HCM, and 85% had negative autopsy.	Genetic analysis showed 29% with positive heterozygous genetic variants; 6 variants were novel. One-third of victims had history of syncope, and 14% had family history of SCD. More than half of the 11 first-degree relatives who underwent genetic testing carried related genetic variants, and 10% had SADS-related clinical features.	This pilot feasibility study shows the value of incorporating cardiac evaluation of surviving relatives and NGS molecular autopsy into conventional forensic investigations in diagnosing young SCD victims in East Asian populations.
Mellor et al. Genetic testing in the evaluation of unexplained cardiac arrest:	Aim: To assess role of genetic testing in individuals without a clinical phenotype.<br>Endpoints: Mutation carrier<br>Study type: Cross-sectional	Inclusion criteria: UCA with documented VT or VF requiring direct current cardioversion or defibrillation, and	29 individuals with a pathogenic variant (17% yield); pathogenic variants identified in 18/72 (25%) of phenotype positive patients; pathogenic variants identified in	Mean age 39 years; 18% with ≥VUS; prior syncope (OR 4) and fam hx of SCA (OR 3.2) were	Nonstandardized genetic testing panels; no controls; no segregation data.<br>Prior syncope and family history of sudden death are predictors

**Study name or acronym; Author; Year published; PMID**	**Aim of study; Endpoints; Study type (RCT, observational — multicenter or single center; case series or other [if RCT include intervention and comparator]); Study size (N)**	**Patient population with inclusion and exclusion criteria**	**Results (absolute event rates, *p* values; OR or RR; 95% CI)**	**Other relevant findings or adverse events**	**Limitations; Other comments; Conclusions**
from the CASPER (Cardiac Arrest Survivors with Preserved Ejection Fraction Registry).<br>• Year published: 2017<br>• PMID: 28600387	Size: 174 genetic testing/ 375 cardiac arrest survivors in CASPER; genetic testing of cardiac genes at the discretion of the treating provider	subsequent testing demonstrated normal left ventricular function (left ventricular ejection fraction ≥50%) and normal coronary arteries (no coronary stenosis >50%)<br>Exclusion criteria: Known causes for cardiac arrest, including an ECG diagnostic of LQTS (persistent resting QTc >460 ms for males and 480 ms for females) or BrS, HCM, marked hypokalemia, drug overdose, or commotio cordis, recognized forms of idiopathic VT	11/102 (11%) of phenotype negative patients	associated with pathogenic variant carrier status	of a positive genetic test. Both arrhythmia and cardiomyopathy genes are implicated. Broad, multiphenotype testing revealed the highest frequency of pathogenic variants in phenotype-negative patients.
Lahrouchi et al. Utility of post-mortem genetic testing in cases of sudden arrhythmic death syndrome.<br>• Year published: 2017<br>• PMID: 28449774	Aim: To define use of postmortem genetic testing following SCD in negative autopsy SCD.<br>Endpoints: Genetic diagnosis in proband and family<br>Study type: Multicenter, retrospective case-control study<br>Size: 302 SCD cases	Inclusion criteria: SCD cases with available DNA and negative autopsy; negative autopsy<br>Exclusion: Structural heart disease or nonspecific pathological features at autopsy	-Death with exercise (10%) or extreme emotion (1.5%)<br>-Family history in 7.1%<br>-18% personal history of syncope or seizures<br>-21 (7%) diagnosed with epilepsy or prior history of epilepsy<br>-24 had consulted health care provider	-19 pathogenic and 15 likely pathogenic variants in RyR2, KCNH2, KCNQ1, SCN5A<br>-1 pathogenic variant in PLN, likely pathogenic versions in TTN (2), PKP2 (1), MYH7 (1)	-88% European heritage<br>-Limited family evaluation<br>-Referral bias
Fox et al. Returning results to family	Aim: To critically appraise the ethical and legal duties to disclose findings to the	Family members stand to benefit in important ways from discoveries	Careful appreciation of how medical research differs from clinical practice and of the uncertainties at stake in	Genomic autopsy studies for sudden death satisfy these	These are among the rare instances in which offering results to family members is not

**Study name or acronym; Author; Year published; PMID**	**Aim of study; Endpoints; Study type (RCT, observational — multicenter or single center; case series or other [if RCT include intervention and comparator]); Study size (N)**	**Patient population with inclusion and exclusion criteria**	**Results (absolute event rates, *p* values; OR or RR; 95% CI)**	**Other relevant findings or adverse events**	**Limitations; Other comments; Conclusions**
members: professional duties in genomics research in the United States.<br>• Year published: 2018<br>• PMID: 30289737	family members of research participants.	that can inform their own health and reproductive risks.	genomic research complicates any warning to relatives. Research laboratories should generally be immune from liability for failing to diagnose or disclose a genetic disorder in time to prevent adverse outcomes for a participant's family members or to return properly interpreted test results for even direct findings under investigation, let alone incidental ones. The only exception is where warning relatives of medical risks is very likely to prevent imminent harm and would not override known participant wishes.	conditions of life-saving potential for relatives without disrespect to subjects.	just permissible but obligatory, not just as a moral matter but as a legal one.
van den Heuvel et al. Informing relatives at risk of inherited cardiac conditions: experiences and attitudes of healthcare professionals and counselees.<br>• Year published: 2019<br>• PMID: 31053782	Aim: To explore how relatives at risk of inherited cardiac conditions can be informed with a family-mediated approach.<br>Study type: Focus groups<br>Size: 28 health care professionals; 25 counselees	Inclusion criteria: Cardiologists, clinical geneticists, genetic counselors and psychosocial workers specialized in cardiogenetics. Probands who had received cardiogenetic counseling in the year prior to the study, and their relatives.	Both health care professionals and counselees believed that relatives should be informed about their risk of inherited cardiac conditions. Generally, counselees felt responsible for informing their relatives themselves. Health care professionals directly contacting relatives was considered a reasonable alternative.	Counselees preferred to receive additional information from health care professionals for relatives (letter, either on paper or via e-mail).	Counselees preferred an approach tailored to patient and family characteristics. Health care professionals preferred uniformity in procedures due to high workload.
Asatryan et al. Molecular and genetic insights into progressive cardiac conduction disease.	Aim: To provide insight into genetics and molecular mechanisms of PCCD and related diseases and also highlight the phenotypic overlaps of PCCD with other inherited cardiac and	PCCD is often a primarily genetic disorder, with clinical and genetic overlaps with other inherited cardiac and metabolic diseases. A number of genes have	Precise genetic diagnosis contributes to risk stratification, better selection of specific therapy and allows familiar cascade screening.		Insight into genetics and molecular mechanisms of PCCD and related diseases

Study name or acronym; Author; Year published; PMID	Aim of study; Endpoints; Study type (RCT, observational — multicenter or single center; case series or other [if RCT include intervention and comparator]); Study size (N)	Patient population with inclusion and exclusion criteria	Results (absolute event rates, *p* values; OR or RR; 95% CI)	Other relevant findings or adverse events	Limitations; Other comments; Conclusions
• Year published: 2019<br>• PMID: 31087102	metabolic diseases, present unmet challenges in clinical practice, and summarize the available therapeutic options for affected patients.	been implicated in PCCD pathogenesis with or without structural heart disease or systemic manifestations			
Broekhuizen et al. No significant improvement of cardiovascular disease risk indicators by a lifestyle intervention in people with familial hypercholesterolemia compared to usual care: results of a randomised controlled trial.<br>• Year published: 2012<br>• PMID: 22490761	Aim: To evaluate the efficacy of an individualized tailored lifestyle intervention on lipids (LDL-C, HDL-C, TC, and triglycerides), systolic blood pressure, glucose, BMI, and waist circumference in people with FH.<br>Study type: Randomized controlled trial<br>Size: 340	Adults with FH (n = 340), recruited from a Dutch cascade screening program, were randomly assigned to either a control group or an intervention group. The personalized intervention consisted of web-based tailored lifestyle advice and personal counseling. The control group received care as usual.	After 12 months, no significant between-group differences of cardiovascular disease risk indicators were observed. LDL-C levels had decreased in both the intervention and control group. This difference between intervention and control group was not statistically significant.	This project suggests that an individually tailored lifestyle intervention did not have an additional effect in improving cardiovascular disease risk indicators among people with FH.	The cumulative effect of many small improvements in all indicators on long-term cardiovascular disease risk remains to be assessed in future studies.
Charron et al. Prenatal molecular diagnosis in hypertrophic cardiomyopathy: report of the first case.<br>• Year published: 2004<br>• PMID: 15386449	Aim: To report the case of a couple who requested prenatal molecular testing after detailed information had been given through a multidisciplinary consultation.<br>Study type: Case report	Prenatal diagnosis in HCM is associated with complex medical and psychological implications, in addition to general ethical considerations, as the potential value of the diagnosis is counterbalanced by the highly variable expression of the disease and the difficulty in predicting its evolution.	The R403L mutation in the MYH7 gene had been previously identified in this family, characterized by a malignant form of HCM. In the specific context of this case, the request of the parents and performed the prenatal diagnosis was agreed to.		This is the first report of a prenatal molecular diagnosis performed in the context of HCM.

Study name or acronym; Author; Year published; PMID	Aim of study; Endpoints; Study type (RCT, observational — multicenter or single center; case series or other [if RCT include intervention and comparator]); Study size (N)	Patient population with inclusion and exclusion criteria	Results (absolute event rates, *p* values; OR or RR; 95% CI)	Other relevant findings or adverse events	Limitations; Other comments; Conclusions
Harper et al. The ESHRE PGD Consortium: 10 years of data collection.<br>• Year published: 2012<br>• PMID: 22343781	Aim: To present an overview of the first 10 years of PGD data, highlighting trends.<br>Endpoints: Pregnancy outcome and follow-up of deliveries	Since it was established in 1997, the ESHRE PGD Consortium has been collecting data from international PGD centers. Ten papers have been published, including data from January 1997 to December 2007.	In data collection I, 16 centers contributed data, which increased to 57 centers by data X (average of 39 centers per data collection). These centers contributed data on over 27,000 cycles that reached oocyte retrieval. Of these cycles, 61% were for aneuploidy screening, 17% for single gene disorders, 16% for chromosomal abnormalities, 4% for sexing of X-linked disease and 2% for social sexing.	Cumulatively, 5,187 clinical pregnancies gave rise to 4,140 deliveries and 5,135 newborns (singletons: 3,182, twins: 921, triplets: 37).	Overview of the first 10 years of PGD data, highlighting trends, including the introduction of laser-assisted biopsy, an increase in polar body and trophectoderm biopsy, new strategies, methodologies and technologies for diagnosis, including recently arrays, and the more frequent use of freezing biopsied embryos. The Consortium data reports represent a valuable resource for information about the practice of PGD.
Kuliev et al. PGD for inherited cardiac diseases.<br>• Year published: 2012<br>• PMID: 22386593	Aim: To present cumulative experience of PGD for inherited cardiac diseases, including familial HCM and DCM, cardioencephalomyopathy and Emery-Dreifuss muscular dystrophy.	A total of 18 PGD cycles were performed, resulting in transfer in 15 of them, which yielded 9 unaffected pregnancies and the births of 7 disease- or disease predisposition-free children.			The data open the prospect of PGD for inherited cardiac diseases, allowing couples carrying cardiac disease predisposing genes to reproduce without much fear of having offspring with these genes, which are at risk for premature or sudden death.
**Investigation of the Family: Cause Not Identified—Clinical and Genetic Investigations**					
Quenin et al. Clinical yield of familial screening after sudden death in young subjects: the French experience.	Aim: To investigate the value of clinical screening in relatives of all subjects who died suddenly before 45 years of age.<br>Study type: Prospective cohort, multicenter	Inclusion criteria: Unexplained SCD with negative autopsy before 45 years of age were included from May 2009 to December 2014.	In 16/64 (25%) families a diagnosis was established by clinical screening.	More diagnoses were established when both parents were investigated. The diagnostic yield also increased when more relatives were screened.	Even without autopsy, familial screening after sudden death in young patients is effective. Broad screening of relatives and systematic tests, including pharmacological challenges, greatly increases the likelihood of diagnosis in families.

**Study name or acronym; Author; Year published; PMID**	**Aim of study; Endpoints; Study type (RCT, observational — multicenter or single center; case series or other [if RCT include intervention and comparator]); Study size (N)**	**Patient population with inclusion and exclusion criteria**	**Results (absolute event rates, *p* values; OR or RR; 95% CI)**	**Other relevant findings or adverse events**	**Limitations; Other comments; Conclusions**
• Year published: 2017<br>• PMID: 28912206	Size: 103 families, out of whom 64 participated				
Tan et al. Sudden unexplained death: heritability and diagnostic yield of cardiological and genetic examination in surviving relatives.<br>• Year published: 2005<br>• PMID: 15998675	Aim: Because SUDs may have heritable causes, cardiological and genetic assessment of surviving relatives of SUD victims may reveal the underlying disease and unmask presymptomatic carriers. The study aimed to establish the diagnostic yield of such assessments.<br>Size: 43 families	Inclusion criteria: 43 consecutive families with > or =1 SUD victim who died at < or =40 years of age.	An inherited disease and likely cause of death in 17 of 43 families (40%) were identified; 12 families had primary electrical disease: CPVT (5 families), LQTS (4 families), BrS (2 families), and long-QT/BrS (1 family). ARVC (3 families), HCM (1 family), and FH (1 family) were also found. Molecular genetic analysis provided confirmation in 10 families.	Finding the diagnosis was more likely when more relatives were examined and in families with > or =2 SUD victims < or =40 years of age. The resting/ exercise ECG had a high diagnostic yield. These efforts unmasked 151 presymptomatic disease carriers (8.9 per family).	Examination of relatives of young SUD victims has a high diagnostic yield, with identification of the disease in 40% of families and 8.9 presymptomatic carriers per family. Simple procedures (examining many relatives) and routine tests (resting/exercise ECG) constitute excellent diagnostic strategies. Molecular genetics provide strong supportive information.
Behr et al. Sudden arrhythmic death syndrome: familial evaluation identifies inheritable heart disease in the majority of families.<br>• Year published: 2008<br>• PMID: 18508782	Aim: To identify more susceptible SADS individuals and causes of death through comprehensive clinical investigation of SADS families.<br>Size: 57 families	Inclusion criteria: Consecutively referred families with SADS death	First-degree relatives [184/262 (70%)] underwent evaluation, 13 (7%) reporting unexplained syncope. 17 (30%) families had a history of additional unexplained premature sudden death(s). 30 families (53%) were diagnosed with inheritable heart disease: 13 definite LQTS, 3 possible/probable LQTS, 5 BrS, 5 ARVC, and 4 other cardiomyopathies.	1 SCN5A and 4 KCNH2 mutations (38%) were identified in 13 definite LQTS families, 1 SCN5A mutation (20%) in 5 BrS families and 1 (25%) PKP2 (plakophyllin2) mutation in 4 ARVC families.	Over-half of SADS deaths were likely to be due to inherited heart disease; accurate identification is vital for appropriate prophylaxis amongst relatives who should undergo comprehensive cardiological evaluation, guided and confirmed by mutation analysis.

**Study name or acronym; Author; Year published; PMID**	**Aim of study; Endpoints; Study type (RCT, observational — multicenter or single center; case series or other [if RCT include intervention and comparator]); Study size (N)**	**Patient population with inclusion and exclusion criteria**	**Results (absolute event rates, *p* values; OR or RR; 95% CI)**	**Other relevant findings or adverse events**	**Limitations; Other comments; Conclusions**
Steinberg et al. Cardiac abnormalities in first-degree relatives of unexplained cardiac arrest victims: a report from the Cardiac Arrest Survivors with Preserved Ejection Fraction Registry.<br>• Year published: 2016<br>• PMID: 27635072	Aim: To prospectively assess first-degree relatives of UCA or SUD victims to screen for cardiac abnormalities.<br>Size: 398	Inclusion criteria: Around 398 first-degree family members (186 UCA, 212 SUD victims' relatives; mean age, 44 ± 17 years) underwent extensive cardiac workup, including ECG, signal averaged ECG, exercise testing, cardiac imaging, Holter monitoring, and selective provocative drug testing with epinephrine or procainamide.	Cardiac abnormalities were detected in 120 of 398 patients (30.2%) with 67 of 398 having a definite or probable diagnosis (17%), including LQTS (13%), CPVT (4%), ARVC (4%), and BrS (3%). The detection yield was similar for family members of UCA and SUD victims (31% vs 27%; *p* = .59). Genetic testing was performed more often in family members of UCA patients (29% vs 20%; *p* = .03). Disease-causing mutations were identified in 20 of 398 relatives (5%).	The most common pathogenic mutations were RyR2 (2%), SCN5A (1%), and KNCQ1 (0.8%).	Cardiac screening revealed abnormalities in 30% of first-degree relatives of UCA or SUD victims, with a clear working diagnosis in 17%. Long-QT, ARVC, and CPVT were the most common diagnoses. Systematic cascade screening and genetic testing in asymptomatic individuals will lead to preventive lifestyle and medical interventions with potential to prevent sudden cardiac death.
Behr et al. Cardiological assessment of first-degree relatives in sudden arrhythmic death syndrome.<br>• Year published: 2003<br>• PMID: 14602442	Aim: To search for evidence of inherited cardiac disease in cases of SADS.<br>Size: 147	Inclusion criteria: 147 first-degree relatives of 32 people who died of SADS	Of 147 first-degree relatives of 32 people who died of SADS, 109 (74%) underwent cardiological assessment. Seven (22%) of the 32 families were diagnosed with inherited cardiac disease: 4 with LQTS; 1 with nonstructural cardiac electrophysiological disease; 1 with myotonic dystrophy; and 1 with HCM.		Families of people who die of SADS should be offered assessment in centers with experience of inherited cardiac disease.
Mellor et al. Genetic testing in the evaluation of unexplained cardiac arrest: from the CASPER (Cardiac Arrest Survivors with Preserved Ejection	Aim: To assess role of genetic testing in individuals without a clinical phenotype<br>Endpoints: Mutation carrier<br>Study type: Cross-sectional<br>Size: 174 genetic testing/ 375 cardiac arrest survivors in CASPER; genetic testing of cardiac genes at the	Inclusion criteria: UCA with documented VT or VF requiring direct current cardioversion or defibrillation, and subsequent testing demonstrated normal left ventricular function (left ventricular ejection	29 individuals with a pathogenic variant (17% yield); pathogenic variants identified in 18/72 (25%) of phenotype positive patients; pathogenic variants identified in 11/102 (11%) of phenotype negative patients	Mean age 39 years; 18% with ≥VUS; prior syncope (OR 4) and fam hx of SCA (OR 3.2) were associated with pathogenic variant carrier status	Nonstandardized genetic testing panels; no controls; no segregation data.<br>Prior syncope and family history of sudden death are predictors of a positive genetic test. Both arrhythmia and cardiomyopathy genes are implicated. Broad, multiphenotype testing revealed

Study name or acronym; Author; Year published; PMID	Aim of study; Endpoints; Study type (RCT, observational — multicenter or single center; case series or other [if RCT include intervention and comparator]); Study size (N)	Patient population with inclusion and exclusion criteria	Results (absolute event rates, *p* values; OR or RR; 95% CI)	Other relevant findings or adverse events	Limitations; Other comments; Conclusions
Fraction Registry).<br>• Year published: 2017<br>• PMID: 28600387	discretion of the treating provider	fraction ≥50%) and normal coronary arteries (no coronary stenosis >50%)<br>Exclusion criteria: Known causes for cardiac arrest, including an ECG diagnostic of LQTS (persistent resting QTc >460 ms for males and 480 ms for females) or BrS, HCM, marked hypokalemia, drug overdose, or commotio cordis, recognized forms of idiopathic VT			the highest frequency of pathogenic variants in phenotype-negative patients.
Papadakis et al. The diagnostic yield of Brugada syndrome after sudden death with normal autopsy.<br>• Year published: 2018<br>• PMID: 29544603	Aim: To assess the impact of systematic ajmaline provo cation testing using high RPLs on the diagnostic yield of BrS in a large cohort of SADS families.<br>Study type: Prospective study<br>Size: 303 SADS families, 911 relatives	303 SADS families (911 relatives) underwent evaluation with resting ECG using conventional and high RPLs, echocardiography, exercise, and 24-h ECG monitor.	An inherited cardiac disease was diagnosed in 128 (42%) families and 201 (22%) relatives. BrS was the most prevalent diagnosis (n = 85, 28% of families; n = 140, 15% of relatives). Ajmaline testing was required to unmask the BrS in 97% of diagnosed individuals.	At initial evaluation, 4 (0.4%) individuals showed a spontaneous type 1 Brugada pattern on the resting ECG, of which 2 were seen only on the high RPLs.	Systematic use of ajmaline testing with high RPLs increases substantially the yield of BrS in SADS families.
Lahrouchi et al. Utility of post-mortem genetic testing in cases of sudden arrhythmic death syndrome.	Aim: To define use of postmortem genetic testing following SCD in negative autopsy SCD.<br>Endpoints: Genetic diagnosis in proband and family<br>Study type: Multicenter, retrospective case-control	Inclusion criteria: SCD cases with available DNA and negative autopsy; negative autopsy<br>Exclusion: Structural heart disease or nonspecific	-Death with exercise (10%) or extreme emotion (1.5%)<br>-Family history in 7.1%<br>-18% personal history of syncope or seizures<br>-21 (7%) diagnosed with epilepsy or prior history of epilepsy	-19 pathogenic and 15 likely pathogenic variants in RyR2, KCNH2, KCNQ1, SCN5A<br>-1 pathogenic variant in PLN,	-88% European heritage<br>-Limited family evaluation<br>-Referral bias

Study name or acronym; Author; Year published; PMID	Aim of study; Endpoints; Study type (RCT, observational — multicenter or single center; case series or other [if RCT include intervention and comparator]); Study size (N)	Patient population with inclusion and exclusion criteria	Results (absolute event rates, *p* values; OR or RR; 95% CI)	Other relevant findings or adverse events	Limitations; Other comments; Conclusions
• Year published: 2017<br>• PMID: 28449774	study<br>Size: 302 SCD cases	pathological features at autopsy	-24 had consulted health care provider	likely pathogenic versions in TTN (2), PKP2 (1), MYH7 (1)	
te Riele et al. Yield of serial evaluation in at-risk family members of patients with ARVD/C.<br>• Year published: 2014<br>• PMID: 25034067	Aim: To determine the optimal approach to longitudinal follow-up regarding screening interval and testing strategy in at-risk relatives of ARVD/C patients.<br>Size: 117 relatives from 64 families	Inclusion criteria: 117 relatives (45% male, age 33.3 ± 16.3 years) were included from 64 families who were at risk of developing ARVD/C by virtue of their familial predisposition (72% mutation carriers [92% plakophilin-2]; 28% first-degree relatives of a mutation-negative proband). Subjects were evaluated by ECG, Holter monitoring, signal-averaged ECG, and CMR.	At first evaluation, 43 subjects (37%) fulfilled an ARVD/C diagnosis according to the 2010 Task Force Criteria. Among the remaining 74 subjects (63%), 11 of 37 (30%) with complete re-evaluation experienced disease progression during 4.1 ± 2.3 years of follow-up. Electrical progression (n = 10 [27%], including by ECG [14%], Holter monitoring [11%], or signal-averaged ECG [14%]) was more frequently observed than structural progression (n = 1 [3%] on CMR).	All 5 patients (14%) with clinical ARVD/C diagnosis at last follow-up had an abnormal ECG or Holter monitor recording, and the only patient with an abnormal CMR already had an abnormal ECG at enrollment.	Over a mean follow-up of 4 years, our study showed that almost one-third of at-risk relatives have electrical progression; structural progression is rare; and electrical abnormalities precede detectable structural changes. This information could be valuable in determining family screening protocols.
Krahn et al. Epinephrine infusion in the evaluation of unexplained cardiac arrest and familial sudden death: from the Cardiac Arrest Survivors with Preserved Ejection Fraction Registry.	Aim: To assess epinephrine infusion may unmask latent genetic conditions associated with cardiac arrest, including LQTS and CPVT.<br>Study type: Prospective observational<br>Size: 170	Inclusion criteria: Patients with UCA (normal left ventricular function and QT interval) and selected family members from the Cardiac Arrest Survivors with Preserved Ejection Fraction Registry	Epinephrine infusion was performed in 170 patients (age, 42 ± 16 years; 49% men), including 98 patients with UCA. Testing was positive for LQTS in 31 patients (18%) and borderline in 24 patients (14%). Exercise testing provoked an abnormal QT response in 42% of tested patients with a positive epinephrine response. Testing for CPVT was positive in 7% and borderline in 5%.	Targeted genetic testing of abnormal patients was positive in 17% of LQTS patients and 13% of CPVT patients.	Epinephrine challenge provoked abnormalities in a substantial proportion of patients, most commonly a prolonged QT interval. Exercise and genetic testing replicated the diagnosis suggested by the epinephrine response in a small proportion of patients. Epinephrine infusion combined with exercise testing and targeted genetic testing is recommended in the workup of

Study name or acronym; Author; Year published; PMID	Aim of study; Endpoints; Study type (RCT, observational — multicenter or single center; case series or other [if RCT include intervention and comparator]); Study size (N)	Patient population with inclusion and exclusion criteria	Results (absolute event rates, *p* values; OR or RR; 95% CI)	Other relevant findings or adverse events	Limitations; Other comments; Conclusions
• Year published: 2012<br>• PMID: 22944906					suspected familial sudden death syndromes.
Huchet et al. Familial catecholamine-induced QT prolongation in unexplained sudden cardiac death.<br>• Year published: 2017<br>• PMID: 28335847	Aim: To describe the use of MST to identify CIQTP in SCD familial screening.<br>Size: 65 families	MST was performed in the screening of 65 consecutives families affected by unexplained SCD referred to the National Referral Centre for Inherited Cardiac Arrhythmias of Nantes. Conventional screening included echocardiography, exercise test, and epinephrine and ajmaline tests.	Patients were considered affected for CIQTP when presenting prolonged QTc interval >480 ms or if >30 ms QT prolongation with the QTc interval >460 ms during the different tests.	Even if CIQTP may be identified by both MST and epinephrine test, MST has the advantage of being safer, easier, and quicker to perform. On the other hand, the complex use of epinephrine test can lead to contraindication or misinterpretation. The use of MST in the context of unexplained SCD and LQTS deserves further investigations and comparative studies.	Preliminary findings have identified CIQTP as a new specific familial phenotype characterized by normal QT duration at rest but major QT lengthening during mental stress. CIQTP seems to play an important role in unexplained VF and MST appears to be a simple and efficient test to identify this pathology.
van der Werf et al. Low rate of cardiac events in first-degree relatives of diagnosis-negative young sudden unexplained death syndrome victims during follow-up.	Aim: To study the prognosis of first-degree relatives of young SUDS victims, in whom the initial cardiologic and genetic examination did not lead to a diagnosis.<br>Size: 417 relatives from 83 families	Inclusion criteria: Vital status of surviving first-degree relatives from 83 diagnosis-negative families who presented to our cardiogenetics department between 1996 and 2009 because of SUDS in ≥1 relatives	Detailed information was obtained (median follow-up 6.6 years; IQR 4.7-9.6 years) in 340 of 417 first-degree relatives (81.5%) from 77 of 83 families (92.8%). Vital status, available in 405 relatives (97.1%), showed that 20 relatives (4.9%) died during follow-up, including 1 natural death before the age of 50. This girl belonged to a family with multiple cases of IVF and	Inherited cardiac disease was diagnosed in 3 families (3.6%).	In first-degree relatives of young SUDS victims with no manifest abnormalities during the initial examination, the risk of developing manifest inherited cardiac disease or cardiac events during follow-up is low. This does not apply to families with obvious familial SUDS.

Study name or acronym; Author; Year published; PMID	Aim of study; Endpoints; Study type (RCT, observational — multicenter or single center; case series or other [if RCT include intervention and comparator]); Study size (N)	Patient population with inclusion and exclusion criteria	Results (absolute event rates, *p* values; OR or RR; 95% CI)	Other relevant findings or adverse events	Limitations; Other comments; Conclusions
• Year published: 2014<br>• PMID: 24882506		aged 1-50 years was retrieved.	SUDS, including another successfully resuscitated sibling during follow-up. Two hundred thirty-four of 340 first-degree relatives (68.8%) underwent cardiologic examination. Of these, 76 (32.5%) were reevaluated.		

ACA = aborted cardiac arrest; ACM = arrhythmogenic cardiomyopathy; ACMG/AMP = American College of Medical Genetics and Genomics/Association for Molecular Pathology; AE = arrhythmic event; AED = automated external defibrillator; aLQTS = “acquired” long-QT syndrome; ARVC = arrhythmogenic right ventricular cardiomyopathy; ARVD/C = arrhythmogenic right ventricular dysplasia/cardiomyopathy; ATX = Achilles tendon xanthomas; AV = atrioventricular; AVNRT = atrioventricular nodal reentrant tachycardia; BBRVT = bundle branch re-entrant ventricular tachycardia; BMI = body mass index; BrS = Brugada syndrome; CAC = coronary artery calcium; CAD = coronary artery disease; CIED = cardiovascular implantable electronic device; CIQTP = catecholamine-induced QT prolongation; CMR = cardiac magnetic resonance imaging; CPR = cardiopulmonary resuscitation; CPT = catecholamine provocation test; CPVT = catecholaminergic polymorphic ventricular tachycardia; CVGC = cardiovascular genetic counseling; CVS = coronary vasospasm; DBS = dried blood spots; DCM = dilated cardiomyopathy; DSP = desmoplakin; EAM = electroanatomic mapping; ECG = electrocardiogram; ED = emergency department; EMS = emergency medical services; ESHRE = European Society of Human Reproduction and Embryology; EST = exercise stress test; ETT = exercise treadmill testing; FH = familial hypercholesterolemia; GC = genetic counseling; GHD = genetic heart disease; GRSA = genetic risk score associations; GWAS = genome-wide association study; HCM = hypertrophic cardiomyopathy; HDL-C = high-density lipoprotein cholesterol; ICD = implantable cardioverter-defibrillator; ICS = intercostal space; ILR = implantable loop recorder; IVF = idiopathic ventricular fibrillation; LCSD = left cardiac sympathetic denervation; LDL-C = low-density lipoprotein cholesterol; LGE = late gadolinium enhancement; LP/P = likely pathogenic/pathogenic; LQTS = long QT syndrome; LVH = left ventricular hypertrophy; MACE = major adverse cardiac events; ME/C = medical examiner and coroner; MR = Mendelian randomization; MST = mental stress test; MVA = malignant ventricular arrhythmias; NAME = National Association of Medical Examiners; NGS = next-generation sequencing; OHCA = out-of-hospital cardiac arrest; PCCD = progressive cardiac conduction disease; PCR = polymerase chain reaction; PET = paraffin-embedded tissue; PG = prolonged grief; PGD = preimplantation genetic diagnosis; PLN = phospholamban; PM = pacemaker; PTS = posttraumatic stress; QALY = quality-adjusted life years; RFCA = radiofrequency catheter ablation; RPL = right precordial lead; RSCD = resuscitated sudden cardiac death; RV = right ventricle; RVOT = right ventricular outflow tract; SADS = sudden arrhythmic death syndrome; SAECG = signal-averaged electrocardiogram; SCA = sudden cardiac arrest; SCD = sudden cardiac death; SMVT = sustained monomorphic ventricular tachycardia; SNCD = sudden noncardiac death; SUD = sudden unexplained death; SUDEP = sudden unexpected death in epilepsy; SUDS = sudden unexplained death syndrome; SUDY = sudden unexplained death in the young; TC = total cholesterol; TKOS = triadin knockout syndrome; TS = Timothy syndrome; UCA = unexplained cardiac arrest; VF = ventricular fibrillation; VPC = ventricular premature complex; VT = ventricular tachycardia; VTA = ventricular tachyarrhythmia; VUS = variants of uncertain significance; WES = whole-exome sequencing; WHO = World Health Organization.
